# Elucidation of the Biosynthetic Pathway of Vitamin B Groups and Potential Secondary Metabolite Gene Clusters Via Genome Analysis of a Marine Bacterium *Pseudoruegeria *sp. M32A2M

**DOI:** 10.4014/jmb.1911.11006

**Published:** 2020-01-17

**Authors:** Sang-Hyeok Cho, Eunju Lee, So-Ra Ko, Sangrak Jin, Yoseb Song, Chi-Yong Ahn, Hee-Mock Oh, Byung-Kwan Cho, Suhyung Cho

**Affiliations:** 1 Department of Biological Sciences, Korea Advanced Institute of Science and Technology, Daejeon 34141, Republic of Korea; 2 KI for the BioCentury, Korea Advanced Institute of Science and Technology, Daejeon 34141, Republic of Korea; 3 Biological Resource Center, Korea Research Institute of Bioscience and Biotechnology, Daejeon 34141, Republic of Korea

**Keywords:** *Pseudoruegeria* sp. M32A2M, whole-genome sequencing, Vitamin B, secondary metabolite

## Abstract

The symbiotic nature of the relationship between algae and marine bacteria is well-studied among the complex microbial interactions. The mutual profit between algae and bacteria occurs via nutrient and vitamin exchange. It is necessary to analyze the genome sequence of a bacterium to predict its symbiotic relationships. In this study, the genome of a marine bacterium, *Pseudoruegeria* sp. M32A2M*,* isolated from the south-eastern isles (GeoJe-Do) of South Korea, was sequenced and analyzed. A draft genome (91 scaffolds) of 5.5 Mb with a DNA G+C content of 62.4% was obtained. In total, 5,101 features were identified from gene annotation, and 4,927 genes were assigned to functional proteins. We also identified transcription core proteins, RNA polymerase subunits, and sigma factors. In addition, full flagella-related gene clusters involving the flagellar body, motor, regulator, and other accessory compartments were detected even though the genus *Pseudoruegeria* is known to comprise non-motile bacteria. Examination of annotated KEGG pathways revealed that *Pseudoruegeria* sp. M32A2M has the metabolic pathways for all seven vitamin Bs, including thiamin (vitamin B1), biotin (vitamin B7), and cobalamin (vitamin B12), which are necessary for symbiosis with vitamin B auxotroph algae. We also identified gene clusters for seven secondary metabolites including ectoine, homoserine lactone, beta-lactone, terpene, lasso peptide, bacteriocin, and non-ribosomal proteins.

## Introduction

According to the World Register of Marine Species (WoRMS) database, 535,681 biotas have been registered with marine taxonomic names among which 447,097 species are reported to inhabit the oceanic ecosystem [1]. Moreover, ~70% of all marine biomass has been estimated to be composed of microorganisms [2]. These abundant microorganisms compose complex marine networks, where the flux of dissolved organic carbon is described as the microbial loop [3]. However, there are more diverse interactions in the network between algae and bacteria beyond the predator-prey relationship [4, 5]. Algae form the phycosphere in their surroundings to exchange diverse metabolites with environmental bacteria [6]. Some bacteria such as *Phaeobacter, Kordia,* and *Marinobacter* are known to exert an algicidal effect through secretion of antibiotics, proteases, or siderophores [7-9], whereas other algae-bacteria interactions are mutual to commensal relationships. Algae can produce a range of dissolved organic matter (DOM), oxygen, and sulfur sources in the phycosphere. In return, algae can be supplied with vitamins B1, B7, and B12 from *Ruegeria*, ferric ions from *Marinobacter*, and there are reports of interactions through bacterial signaling molecules [10-12]. Among diverse marine bacteria, the genus *Roseobacter* is considered a significant group interacting with algae [13]. *Roseobacter*, which accounts for 25% of marine bacteria, is a bacterial genus belonging to the *Rhodobacteraceae* of *alpha-proteobacteria*. *Roseobacter* species are expected to be actively involved in this interaction as they can produce a variety of secondary metabolites [14]. However, other species from diverse phylogenetic groups such as *Flavobacterium* from phylum *Bacteriodetes* also play a critical role in the algal-bacterial bioeconomy. The relationship between vitamin auxotroph algae surviving by trading nutrients with marine bacteria that contain vitamin biosynthesis pathways has been reported previously [15].

*Psuedoruegeria* is a member of the *Rhodobacteraceae* family, and is named after its closely related cousin, *Ruegeria. Pseudoruegeria* are marine bacteria frequently found in South Korea, China, and Japan. In South Korea, *P. insulae* sp. nov. and *P. litorisediminis* sp. nov.were isolated from a tidal flat sediment and *P. limi* sp. Nov. was isolated from mudflats in the Yellow Sea [16-18]. Further, *P. aestuaril* sp. nov. was isolated from a tidal flat at Muuido, *P. sabulilitoris* was isolated from seashore sand on GeoJe Island, *P. haliotis* sp. nov. was isolated from the gut of the abalone *Haliotis discus hannai*, *P. lutimaris* sp. nov. was isolated from the tidal flat sediment of Hwang-do, and *P. aquimaris* gen. nov.*,* was isolated from seawater in Hwajinpo of the East Sea [19-24]. Most *Pseudoruegeria* found in Korea were gram-stain-negative, aerobic, non-motile, and rod-shaped bacteria. Moreover, these were generally found in tidal flat sediments or seashore sand, and sometimes in seawater. However, *P. marinistellae* sp. nov.*,* isolated from an unidentified starfish in Sanya, China, was closely related to *P. sabulilitoris* sp. GJMS-35 isolated in Korea, with a similarity of 98.42% based on 16S rRNA analysis. However, *P. marinistellae* sp. nov. was a facultatively anaerobic bacterial strain, unlike most *Pseudoruegeria* species reported as aerobic strains [25]. *Pseudoruegeria* sp. SK021, isolated from the North Sea sediment was disclosed to have a relatively small draft genome size of 3.95 Mb and 3,747 protein-coding sequences upon genome sequencing [26]. Interestingly, while most *Pseudoruegeria* showed a non-motile property in laboratory conditions, the genome of *Pseudoruegeria* sp. M32A2M possessed a gene cluster for a flagellar apparatus, indicating the potential for flagellar expression under some conditions.

With the introduction of NGS technologies and bioinformatics tools, large amounts of nucleic acid sequence data can be obtained. These techniques and tools are also used in ecological studies [27]. A comparative genomics study of various algal species suggests that several algal groups need to obtain vitamins such as thiamin, cobalamin, and biotin from the environment [28]. Based on sequence data, the genetic background of the relationship between each bacteria-alga pair can be analyzed. In this study, we performed genome sequencing of *Pseudoruegeria* sp. M32A2M isolated from seawater at Geojedo in South Korea. Primary genome analysis based on genome assembly and annotation was conducted. Since *Ruegeria* can supply vitamins to algae in its symbiotic interaction, we searched for biosynthetic pathways possessed by *Pseudoruegeria* sp. M32A2M, which may be required or necessary for symbiosis with other organisms. Furthermore, secondary metabolite gene clusters in *Pseudoruegeria* sp. M32A2M were investigated to determine the potential secondary metabolites for communication or defensive purposes with other marine microorganisms.

## Materials and Methods

### Isolation and Scanning Electron Microscopy

*Pseudoruegeria* sp. M32A2M isolated from the coastal region of GeoJe island was cultured in marine broth 2216 (BD Difco, USA) or marine agar at 30°C. For scanning electron microscopy (SEM), *Pseudoruegeria* sp. M32A2M were cultured in marine broth and centrifuged at 4,000 rpm at 4°C. The bacterial cell pellet was resuspended in a 2.5% paraformaldehyde-glutaraldehyde mixture buffered with 0.1 M phosphate (pH 7.2). The sample was fixed in the solution for 2 h, post-fixed in 1% osmium tetroxide in the same buffer for 1 h, dehydrated in graded ethanol, and substituted with isoamyl acetate. They were then dried at the critical point in CO_2_. Finally, the samples were sputtered with gold in a sputter coater (SC502, Polaron) and were observed using a scanning electron microscope and FEI Quanta 250 FEG (FEI, USA). The isolated bacterium *Pseudoruegeria* sp. M32A2M was deposited in the KCTC (Korea Collection for Type Culture), with the accession number KCTC 18616P.

### Genome Sequencing and Genome *de novo* Assembly

Genomic DNA of *Pseudoruegeria* sp. M32A2M was extracted using the Wizard Genomic DNA Purification Kit (Promega, USA) following the manufacturer’s protocol. The quality of extracted genomic DNA was checked by NanoDrop 2000 (ThermoFisher Scientific, USA) for a UV absorbance ratio (260:280) at ~2 and inspection by 1% agarose gel electrophoresis. A genome sequencing library with an insert size of 550 bp was prepared using the TruSeq Nano DNA Library Prep Kit (Illumina, USA) following the manufacturer’s protocol. The prepared genome sequencing library was sequenced using a 250-cycle paired-end reaction on the Illumina Miseq platform. Raw sequencing reads were subjected to PlasmidSeeker for the detection of a native plasmid [29]. Sequencing data were processed on a CLC Genomics Workbench 6.5.1. (CLC bio, Denmark). PhiX, adapters, and quality trimmed reads were used for *de novo* genome assembly (word size = 24, bubble size = automatic, and mapping option = map reads back to contigs (slow)). This Whole Genome Shotgun project has been deposited at DDBJ/ENA/GenBank under the accession VNKR00000000. The version described in this paper is version VNKR01000000.

### Phylogenetic Analysis

Genome sequences of each strain used for phylogenetic analysis were downloaded from the NCBI genome portal. The phylogenetic tree was reconstructed by utilizing the Up-to-date Bacterial Core Gene (UBCG) analysis pipeline [30]. Randomized Axelerated Maximum Likelihood (RAxML) was used to generate the phylogenetic tree from calculated distance data [31].

### Gene Annotation and Secondary Metabolite Biosynthesis Gene Prediction

*De novo* assembled genome sequence of *Pseudoruegeria* sp. M32A2M was annotated using the NCBI Prokaryotic Genome Annotation Pipeline (PGAP) [32]. Subsequently, the amino acid sequences were extracted from the annotated coding genes and searched against KEGG Orthology (KO) ID [33], Clusters of Orthologous Groups (COG) [34], and Gene Ontology (GO) [35]. Secondary metabolite biosynthesis gene clusters were predicted via AntiSMASH 5.0 [36].

### Prediction of Genome Structure

To analyze the start and stop codon usage, the first three and last three nucleotides (TGA, TAG, or TAA) were extracted based on the predicted coding sequences (CDSs), and in pseudogenes, the start codons except the reinitiating position were used. To detect the Shine-Dalgarno sequence motif, the nucleotide sequences between 20 to 1 nt upstream from the start codon were subjected to MEME (Multiple Em for Motif Elicitation) [37]. The promoter motif was searched from nucleotides between the 100 to 1 nt upstream of RNA genes.

## Results and Discussion

### Morphology of *Pseudoruegeria* sp. M32A2M

**Fig. 1:**
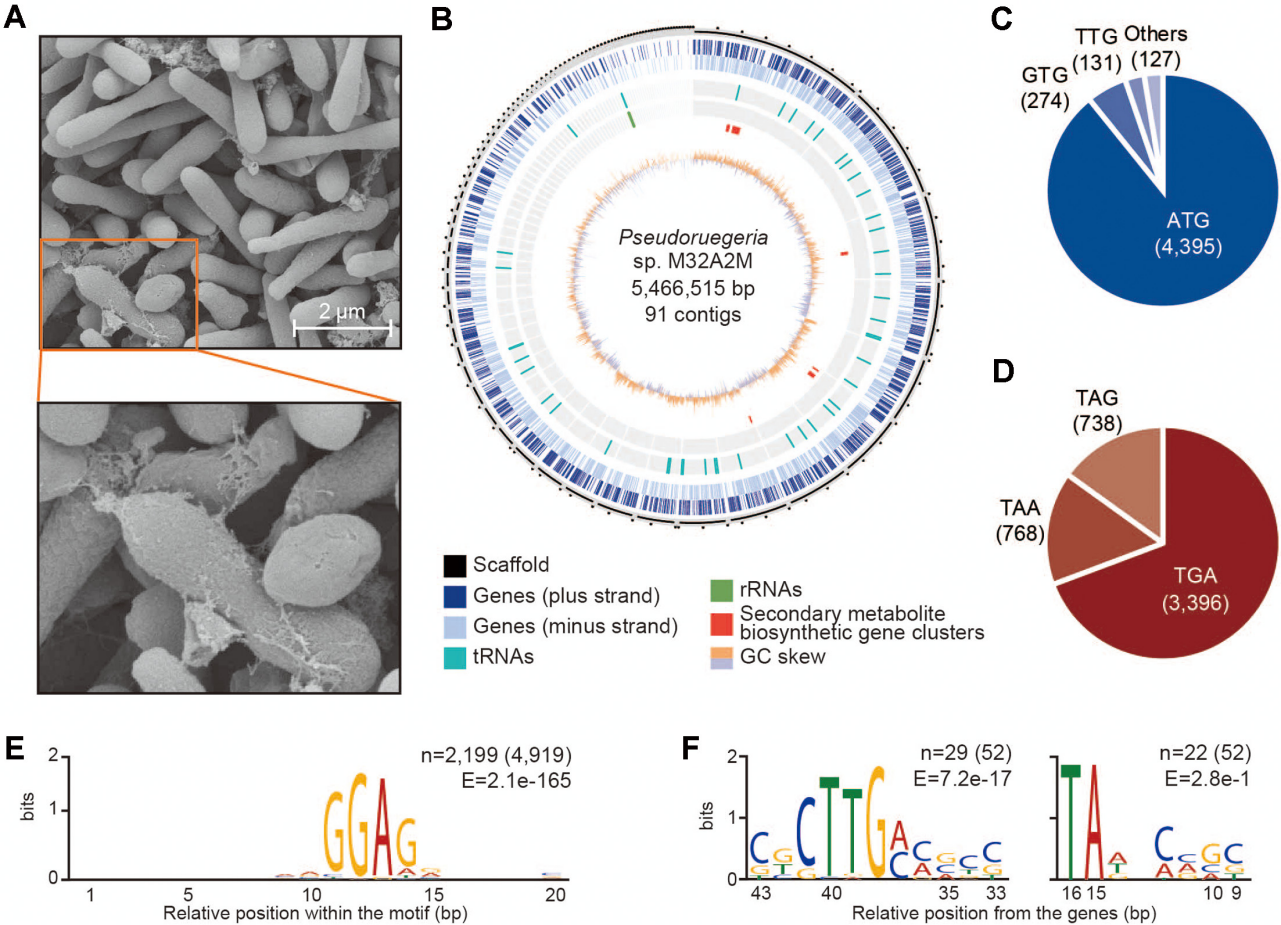
General features of *Pseudoruegeria* sp. M32A2M. (**A**) Scanning electron microscopy of *Pseudoruegeria* sp. M32A2M. (**B**) Circular representation of the draft genome of *Pseudoruegeria* sp. M32A2M . From the outside to the center: scaffolds in the order of length (black, ticks every 100 Kb), genes on the plus strand (blue), genes on the minus strand (light blue), tRNA (teal), rRNA (green), secondary metabolite biosynthetic gene clusters (red), and GC skew (orange and light purple). (**C**) Start codon usage. (**D**) Stop codon usage. (**E-F**) Conserved motifs from nucleotides between 20 to 1 nt upstream of the CDSs (**E**) and between 100 to 1 nt upstream of RNA genes (**F**), were searched using MEME.

We observed the morphology of *Pseudoruegeria* sp. M32A2M using scanning electron microscopy (SEM) (Fig. 1A). As previously reported, the morphology of *Pseudoruegeria* sp. M32A2M is ovoid to rod-shaped with the size of the bacterium at 1–3 μm in length and 0.5 μm in breadth. *Pseudoruegeria* sp. M32A2M seems to divide by binary fission similar to *E. coli*. *Pseudoruegeria* sp. M32A2M is always observed in aggregates both in liquid media and solid media. Together, as shown in SEM, a glue-like substance from cells was secreted near the cell surface or the end of the cell. There are reports that some biomolecules such as proteins, extracellular DNA, and polysaccharides are emitted from bacteria for biofilm matrix formation [38]. These biomolecules emitted from *Pseudoruegeria* sp. M32A2M seem to help the association between cells.

### Genome Sequencing and Gene Annotation

We then performed genome sequencing of *Pseudoruegeria* sp. M32A2M and genome assembly (Fig. 1B). As a result, *Pseudoruegeria* sp. M32A2M has a draft genome of 5.5 Mb from 91 scaffolds and a DNA G+C content of 62.4% (Table S1). The maximum length of the scaffold was 733,566 bp, and the minimum length of the scaffold was 1,015 bp. No evidence of native plasmids was found when searched with PlasmidSeeker [29]. Further, through *in silico* prediction and annotation, we identified 5,101 total features. Among them, the CDSs of 5,049 genes containing 4,927 functional genes and 122 pseudogenes were annotated (Tables 1 and S2). In addition, a total of 52 RNA genes, one copy each of the complete rRNA genes (5S, 16S, 23S), 46 tRNAs, and three non-coding genes were annotated. Besides, Cas1 and Cas2 coding regions, which are known to function as a complex, were found downstream of the CRISPR array [39].

From gene annotation results, we investigated the RNA polymerase subunits and sigma factors, which are core proteins for transcription. There were four kinds of RNA polymerase subunits: alpha (RpoA), beta (RpoB), beta’ (Rp°), and omega (RpoZ) along with several sigma factors, RpoD, and RpoH, and no assigned sigma factors were found together (Table 2). Sigma factors that are not searched with stringent e-value cut-offs from BLASTp seem to have degenerated sequences from known minor sigma factors such as *rpoE*, *rpoF*, *rpoN*, *rpoS*, and *fecI* found in conventional bacteria such as *E. coli*. However, there are four genes annotated as sigma-54-dependent Fis family transcriptional regulators; FPS10_00340, FPS10_02200, FPS10_17975, and FPS10_25320. These genes indicate the possible existence of non-annotated RpoN.

Interestingly, we found flagella-related gene clusters in *Pseudoruegeria* sp. M32A2M*,* which is known as a non-motile bacterium. Interestingly, *Pseudoruegeria* sp. M32A2M has full gene clusters for flagellar formation including flagellar biosynthesis proteins (FlbT, FlaF, FlgJ, FliQ, FlhB, and FlhA), flagellar basal body related proteins (FliL, FliI, FlgK, FlgE, FliF, FliL, FlgH, FlgA, FlgG, FlgF, FliE, and FlgC), motor proteins (FliG, MotA, and FliM/FliN), and the secretion system pore protein (FliP) (Table S3). Although motility of *Pseudoruegeria* sp. M32A2M was not observed in the cultured condition, its expression may be activated by habitat, environmental stresses such as symbiosis with other marine microorganisms, and changes in nutrients or temperature. Since *Pseudoruegeria* is known to inhabit seawater as well as tidal flat sediments or seashore sand as mentioned earlier, their expression may change according to their circumstances.

### Genome Structure Analysis

With the assigned CDSs, the usage of the start and stop codon in *Pseudoruegeria* sp. M32A2M was inspected. The overall accuracy of the start and stop codon prediction is assumed 89.9% according to the report of NCBI Prokaryotic Genome Annotation Pipeline [32]. The most commonly used codon, ATG, was represented in the CDS of 4,395 among 4,927 (89.2%) and alternatively GTG, TTG, and other codons were used together (Fig. 1C). The order of start codon usage follows the previously reported order of start codon translation strength [40]. The most frequently used codon was TGA (3,396 among 4,902, 69.3%), followed by TAA and TAG (Fig. 1D and Table S4).

Like other bacterial species, the Shine-Dalgarno sequence of *Pseudoruegeria* sp. M32A2M was found upstream of the CDSs. Enriched motifs in the sequences within 20 nt upstream of the CDSs are shown (Fig. 1E). Among the total of 4,919 query sequences, the motif enriched in 2,199 sequences was aaGGAGg. This enriched motif is similar to the poly-purine sequence of *E. coli* (UAGGAGGU). Further, the promoter sequence motif was predicted from RNA genes because the transcription start site is equivalent to the gene start position.

From 52 RNA query sequences (3 rRNAs, 46 tRNAs, and 3 ncRNAs), DNA sequences were extracted from the 50 to 21 upstream and 20 to 1 upstream, respectively, to search promoter motifs. As a result, CTTG(a/c)(c/a) and TA motifs were found as -35 element and -10 element with E-values of E=7.2e^-17^ and E=2.8e^-1^, respectively (Fig. 1F). The distance between two elements was ~17 nt, which is in the normal spacing range of 16-19 nt. A high E-value at -10 element may be a result of variance due to a small number of query sequences. Further information such as transcription initiation sites is required to accurately determine the promoter sequence of an given organism [41]. The *rpoD* subunit of RNA polymerase plays a role in recognizing the promoter. In particular, the 2.4 subregion and the helix-turn-helix (HTH) motif of the 4.2 subregion into rpoD are known to recognize -10 and -35 elements respectively and to be well conserved regardless of species. The 2.4 and 4.2 HTH motifs of *Pseudoruegeria* sp. M32A2M were perfectly matched with the ones of the same *Rhodobacteraceae* family *Ruegeria pomeroyi*, and were also considerabley conserved for the ones of *Enterobacteriaceae* family *E. coli* excluding K578Q and D581S substitution into HTH motif (Fig. S1). Such little substitutions may reflect the phylogenetical distance among three species and result in the subtly different promoter specificity.

### Genome-Scale Phylogenetic Analysis of *Pseudoruegeria* sp. M32A2M

**Fig. 2:**
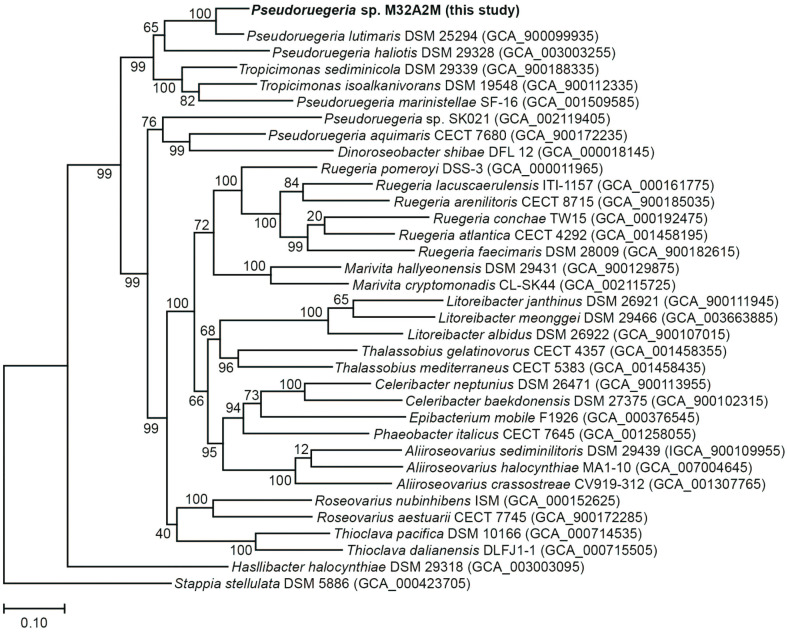
Genome level phylogenetic analysis. Phylogenetic analysis from *Pseudoruegeria* sp. M32A2M and 34 closely related taxa was performed based on their core genes. *Stappia stellulata* was selected as the outgroup. The tree is drawn to scale, with branch lengths in the same units as those of the evolutionary distances used to infer the phylogenetic tree. The evolutionary distances were provided by UBCG and plotted by RAxML.

In the previous report, the phylogenetic analysis of another Pseudoruegeria species isolated from GeoJe island based on 16S rRNA sequences was performed [24]. The 16s rRNA sequence has been widely used for conventional phylogenetic analysis. However, advances in NGS technology have made it easier to obtain genome sequences, and comparison methods for multiple highly conserved genes have emerged [30]. The Up-to-date Bacterial Core Gene (UBCG) analysis pipeline was used to reconstruct the phylogenetic tree of *Pseudoruegeria* sp. M32A2M. Based on the genome sequence of *Pseudoruegeria* sp. M32A2M and other related marine bacteria, phylogenetic analysis was performed (Fig. 2). Thirty-four available marine bacterial genome sequences were obtained from the NCBI genome portal. The input data included all sequence data from species reported as *Pseudoruegeria* and the newly assembled genome of *Pseudoruegeria* sp. M32A2M. *Stappia stellulata* was selected as the outgroup. The distance values calculated from the UBCG pipeline were plotted to a phylogenetic tree using RAxML [31]. The phylogenetic tree analysis result was similar to the result of the 16S rRNA sequence comparison. However, *Pseudoruegeria* sp. SK021 and *Pseudoruegeria aquamaris* were found to be farther away than other *Pseudoruegeria* species and were found closely related to *Dinoroseobacter shibae* species. Besides, two species reported as *Tropicimonas* were found to be bound with *Pseudoruegeria* species.

### Functional Categorization

**Fig. 3:**
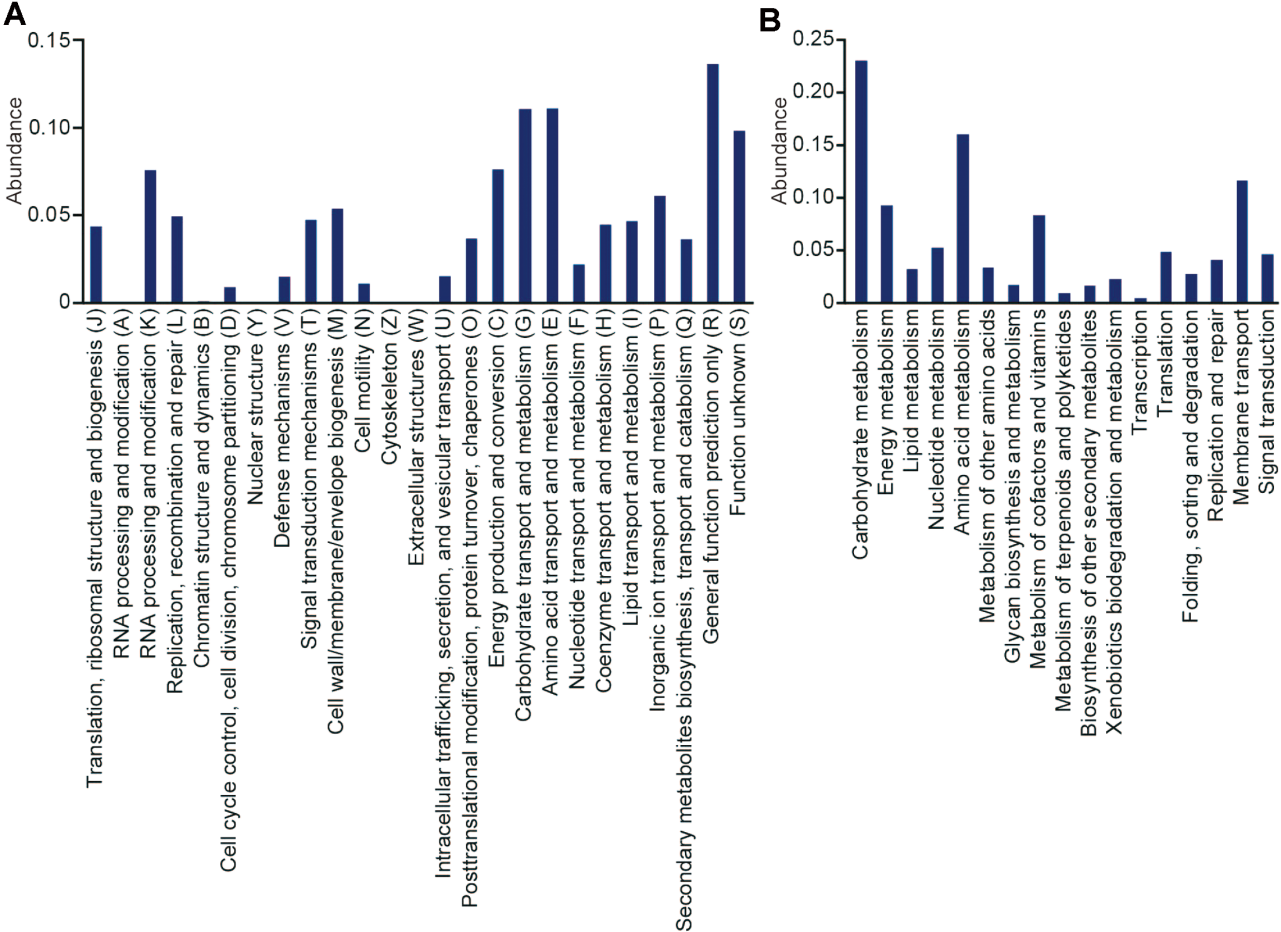
KEGG pathway analysis and COG analysis. Among 4,927 coding genes, 3,870 genes were categorized by COG function (**A**) and 2,443 genes were categorized by KEGG Orthology (**B**).

Further, functional annotation of CDS was performed using three pipelines, KEGG Orthology (KO), Clusters of Orthologous Groups of proteins (COG), and Gene Ontology (GO) (Table S2). In results, among 4,927 protein-coding genes, a total of 3,870 COG functions, 2,443 KO IDs, and 2,140 GO terms were annotated. A total of 4,025 genes were assigned to at least one functional category of three. In COG category assignment, except for poorly characterized categories (R and S), carbohydrate metabolism (G) and amino acid metabolism (E) showed high abundance (Fig. 3A). In KEGG analysis, carbohydrate metabolism, amino acid metabolism, metabolism of cofactors and vitamins, and membrane transporter categories had a high abundance of genes assigned consistent with COG analysis (Fig. 3B). In general, carbohydrate and amino acid metabolism related genes are abundant in most cells. Notably, the high abundance of cofactors and vitamins metabolism-related genes in *Pseudoruegeria* sp. M32A2M is noticeable in the points that several algal strains are known to be vitamin B auxotrophs and *Ruegeria* are known to exchange metabolites such as the vitamin B group with algae [28].

### Vitamin B Biosynthesis Pathways in *Pseudoruegeria* sp. M32A2M

**Fig. 4:**
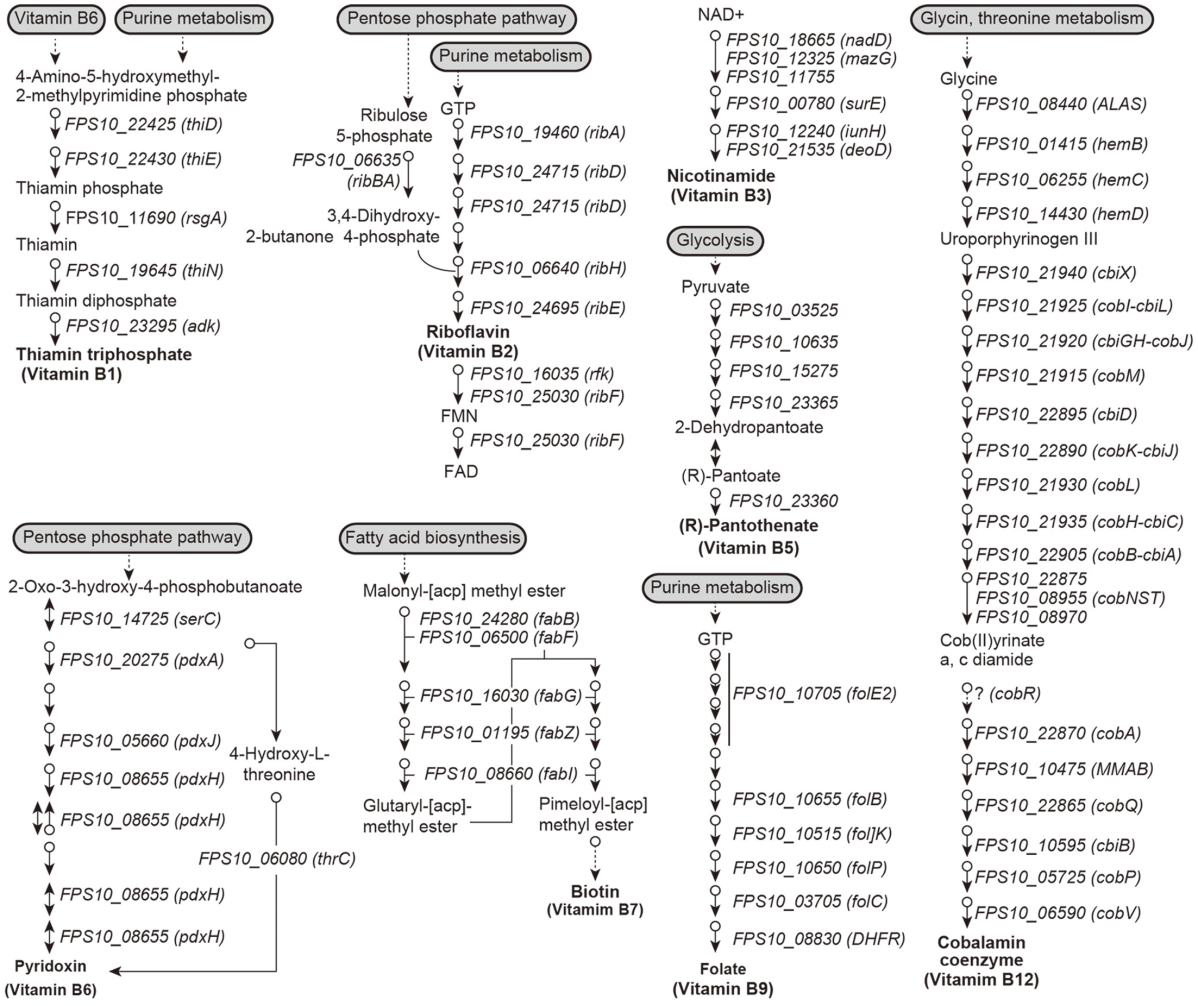
Vitamin B group biosynthesis metabolic pathways in *Pseudoruegeria* sp. M32A2M. In total, seven vitamin B (B1, B2, B3, B6, B7, B9, and B12) pathways were discovered based on the KEGG pathway database and the related enzymes were identified. Individual reactions are represented as arrows with the corresponding gene id (gene name).

Based on the inspection of KEGG functional annotation of *Pseudoruegeria* sp. M32A2M, we investigated the vitamin B biosynthesis-related metabolic pathways. We identified the related enzymes from biosynthesis pathways for the entire vitamin B group, thiamin (vitamin B1), Riboflavin (vitamin B2), Nicotinamide (vitamin B3), pantothenate (vitamin B5), Pyridoxin (vitamin B6), Biotin (vitamin B7), Folate (vitamin B9), and cobalamin (vitamin B12) (Fig. 4). Among the vitamin B group, vitamin B1, B7, and B12 that are produced by bacteria are essential to vitamin-auxotroph algae, and in return, the bacteria uptake nutrients from algae [15, 42-44]. Vitamin B1, B7, and B12 are also known to be required for harmful algal bloom (HAB), by promoting algal growth and regulating the dynamics of HABs [45]. For vitamin B1, there are five known natural thiamin (or vitamin B1) phosphate derivatives: thiamin monophosphate (ThMP), thiamin pyrophosphate (TPP), thiamin triphosphate (ThTP), adenosine thiamin diphosphate (AThDP), and adenosine thiamin triphosphate (AThTP). TPP participates in carbohydrate and amino acid catabolic pathways as a coenzyme [46]. Biotin is also known as vitamin B7, which is known for its activity as a cofactor in fatty acid biosynthesis, amino acid metabolism, and gluconeogenesis [47]. Cobalamin or vitamin B12 participates in enzymatic reactions as a coenzyme. Such cobalamin-requiring enzymes include isomerase, methyltransferase, and dehalogenase. Cobalamin also is known as an essential cofactor for controlling DNA biosynthesis and fatty acid/amino acid related metabolism [48].

### Secondary Metabolites Biosynthesis Clusters in *Pseudoruegeria* sp*. M32A2M*

**Fig. 5:**
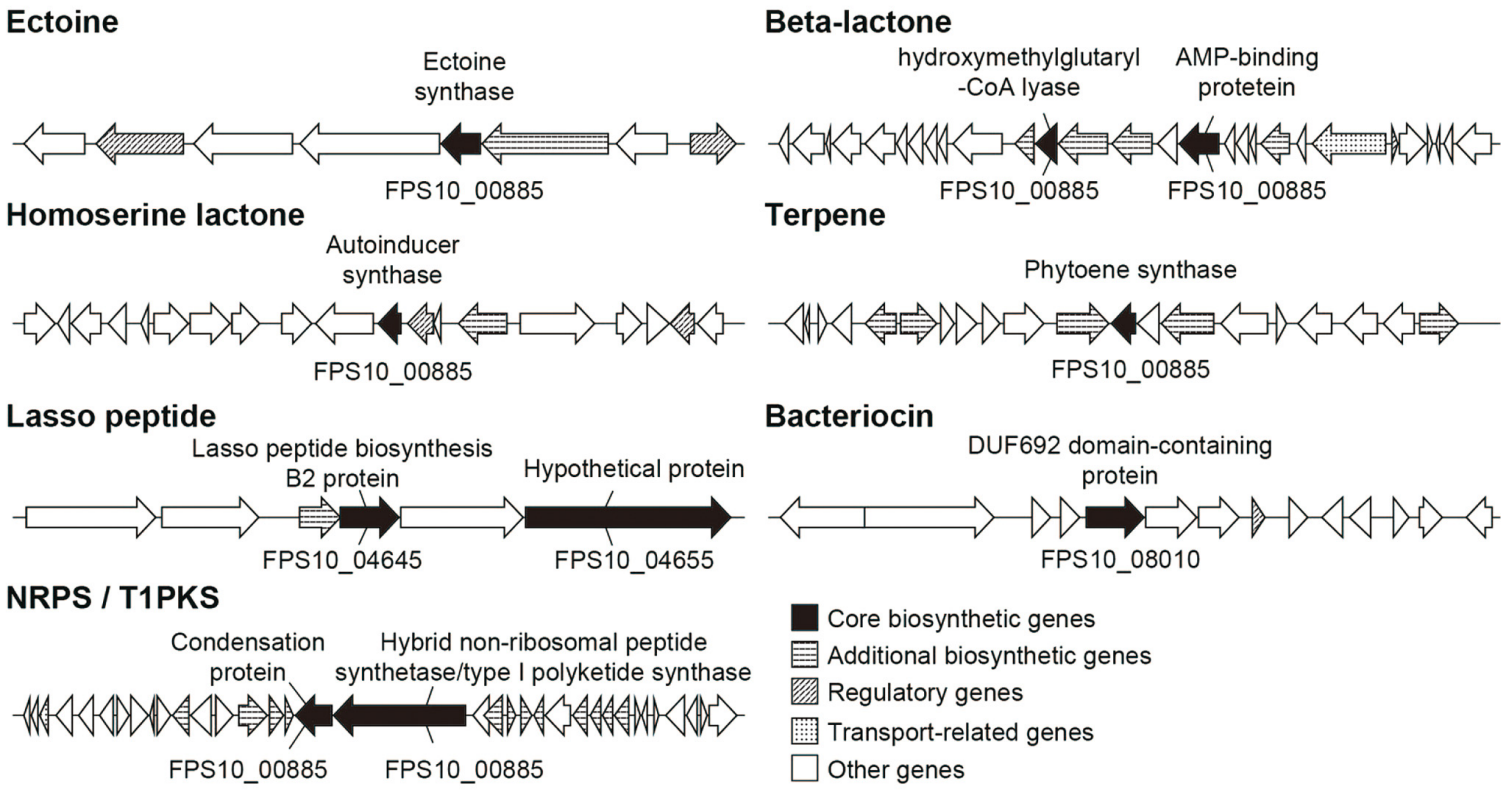
Predicted secondary metabolite biosynthetic gene clusters. Secondary metabolite biosynthetic pathways are predicted based on the genome sequence of *Pseudoruegeria* sp. M32A2M by AntiSMASH. In total, seven clusters were predicted; ectoine, homoserine lactone, beta-lactone, terpene, lasso peptide, bacteriocin, and NRPS/T1PKS. Each gene category is marked by a gray-scale contrast.

Secondary metabolites are not directly related to the cell but play a crucial role in defense against other microorganisms such as viruses or microbes, and against harmful stresses such as toxins or UV exposure. In addition, these metabolites are essential in a symbiotic relationship or in competition with other organisms. We thus examined the potential secondary metabolite-producing clusters in *Pseudoruegeria* sp. M32A2M using AntiSMASH (Fig. 5). As a result, seven secondary metabolite production clusters were predicted as follows: terpene, lasso peptide, bacteriocin, beta-lactone, ectoine, homoserine lactone, and NRPS/T1PKS. Except for ectoine and homoserine lactone whose primary function is to maintain osmotic homeostasis and quorum sensing, other compounds are likely to be used in defense mechanisms. Ectoine is one of the two non-offensive compounds of the seven predicted secondary metabolites. Ectoine serves as protection from osmotic stress, and it is found in a wide range of gram-negative and gram-positive bacteria [49]. The best-characterized homoserine lactone is the bacterial quorum-sensing signal molecule, N-acyl-L-homoserine lactone (AHL). AHLs enable bacterial cells to communicate with each other to control the behavior of the population by regulating gene expression [50]. Studies regarding AHL also reveal that bacterial AHL might be used as a signaling molecule to alter the gene expression of some plant species where the response of chloroplasts to AHLs is explained by their bacterial origin [51]. Beta-lactones are cyclic carboxylic esters formed by intramolecular esterification. There are as many as 30 families of naturally produced beta-lactone derivatives, many with bioactivity against bacterial species [52]. Some beta-lactones exhibit anti-microbial effects, whereas some are used as building blocks of more complex bio-compounds, including flavor or fragrances, antibiotics, and anti-cancer drugs. Terpene is a large group of diverse organic compounds produced mainly by plants or fungi for defense purposes [53, 54]. However, it was recently discovered in bacterial species such as *Streptomyces albidoflavus, Myxobacteria,* and even in some cyanobacterial species. Additionally, we found that *Pseudoruegeria* sp. M32A2M has a well-conserved pathway of terpenoid backbone biosynthesis, suggesting its ability to produce diverse bioactive compounds [55]. Lasso peptides are ribosomally synthesized peptides with a typical length of around 20 amino acids [56, 57]. Lasso peptides are post-translationally modified into a cyclic peptide, where its C-terminal tail is protected by its ring structure (“lasso” structure). Lasso peptides can have diverse functions, including anti-microbial effects. The thermal and chemical resistance derived from its lasso structure makes lasso-peptide a potential pharmaceutical peptide. Bacteriocins are another group of ribosomally synthesized and post-translationally modified proteins that inhibit or usually kill a narrow spectrum of microorganisms [58, 59]. Bacteriocins are produced in an inactive form and gain bioactivity through post-translational modification. There are a variety of bacteriocins, resulting in diverse modes of action. While some assault peptidoglycan biosynthesis and inhibit cell wall biosynthesis, others function with pore neutralizing membrane potential, and inhibit gene or protein expression. Non-ribosomal peptide synthetases polymerize peptides (non-ribosomal peptides, NRP) independent of mRNA [60, 61]. NRPS genes are typically found in a cluster of genes coding large multifunctional enzyme complexes. Each cluster works to synthesize a particular NRP product in a modular manner. NRPS genes are mostly found in bacterial or fungal species and are sources of new antibacterial agents. Diverse NRPs have been discovered and studied for their antibiotic, antiviral, anti-inflammatory, and anti-cancer effects.

Taken together, *Pseudoruegeria* sp. M32A2M has the possibility of occupying an important niche in the marine ecosystem, and can respond to various stimulants and organisms including algae. As the genetic elements responsible for the production of several secondary metabolites were identified, further research is required to determine their regulatory mechanisms. As *Pseudoruegeria* sp. M32A2M was isolated from the coastal region where harmful red tides occur frequently [62, 63], further understanding of its interaction with algal species might provide insights into controlling harmful algal bloom.

## Supplementary material

Supplementary data for this paper are available on-line only at http://jmb.or.kr.



## Figures and Tables

**Table 1:** Gene annotation statistics.

Features annotated	*Pseudoruegeria* sp. M32A2M
**Coding sequences (CDSs)**	**5,049**
Functional CDSs	4,927
Pseudogenes	122
**RNA genes**	**52**
rRNAs	1, 1, 1 (5S, 16S, 23S)
tRNAs	46
ncRNAs	3
**Total annotated features**	**5,101**

**Table 2:** RNA polymerase subunit and sigma factors identified in Pseudoruegeria sp. M32A2M.

Gene ID	Gene	Function	Num. of Amino acid	Size (Da)
FPS10_11085	*rpoA*	DNA-directed RNA polymerase subunit alpha	348	36,833
FPS10_15795	*rpoB*	DNA-directed RNA polymerase subunit beta	1,390	153,105
FPS10_15790	*rpoC*	DNA-directed RNA polymerase subunit beta'	1,427	157,458
FPS10_10510	*rpoZ*	DNA-directed RNA polymerase subunit omega	126	13,252
FPS10_24745	*rpoD*	RNA polymerase sigma factor RpoD	675	75,645
FPS10_02115	*rpoH*	RNA polymerase sigma factor RpoH	312	33,691
FPS10_24655	-	RNA polymerase factor sigma-32	300	33,358
FPS10_22820	-	RNA polymerase sigma factor	203	21,954
FPS10_22950	-	sigma-70 family RNA polymerase sigma factor	175	19,707
FPS10_23250	-	RNA polymerase sigma factor	183	19,447
