# Supplementary material for: Elucidation of the Biosynthetic Pathway of Vitamin B Groups and Potential Secondary Metabolite Gene Clusters Via Genome Analysis of a Marine Bacterium Pseudoruegeria sp. M32A2M
Source: J Microbiol Biotechnol. 2020 Jan 17;30(4):505–14. doi: 10.4014/jmb.1911.11006 (PMC9728324; doi:10.4014/jmb.1911.11006)
Supplement: Supplementary file 1 [file JMB-30-4-505-supple.pdf]

J. Microbiol. Biotechnol.
https://doi.org/10.4014/jmb.1911.11006

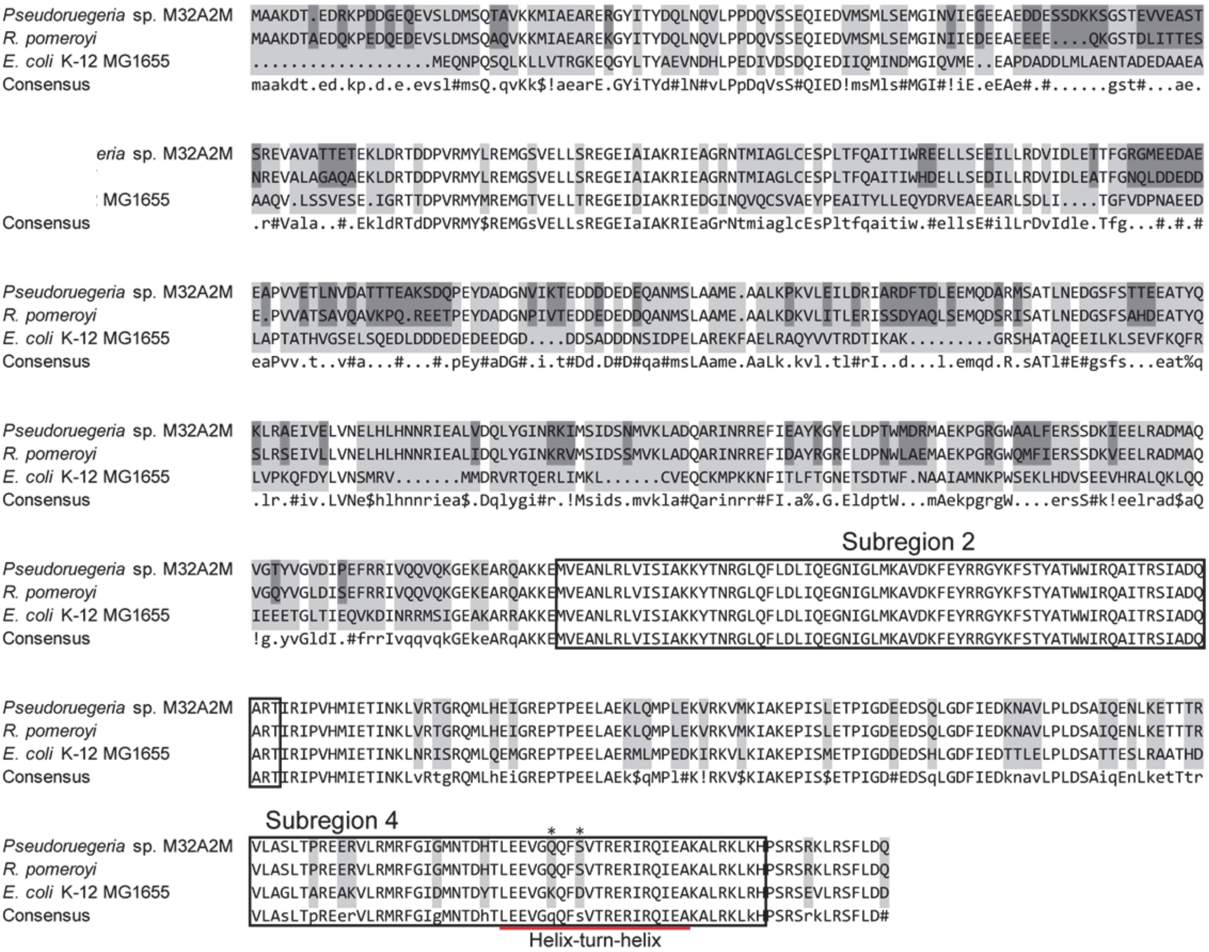

Supplementary Figure 1. RpoD sequence alignment. Protein sequence alignment was performed for RpoD from three species, *Pseudoruegeria* sp. M32A2M (FPS10_24745, this study), *Ruegeria pomeroyi* (NCBI RefSeq: WP_011047484.1), and *Escherichia coli* (NCBI RefSeq: NP_417539.1). Multalin version 5.4.1 was used for the analysis. Subregion 2 and 4 are represented in a rectangle, and the helix-turn-helix motif in subregion 4 is highlighted in red. The amino acid degeneration from any of the three was highlighted in light gray and the sequence degeneration between *Pseudoruegeria* sp. M32A2M and *R. pomeroyi* is highlighted in dark gray. The substituted two amino acids into HTH motif (K578Q and D581S) against *E. coli* were marked as asterisk.

Supplementary Table 1. Genome assembly statistics

Categories	*Pseudoruegeria* sp. M32A2M
Number of scaffolds less than 1,000 bp	0
Number of scaffolds between 1,000 bp –10,000 bp	39
Number of scaffolds between 10,000 bp – 100,000 bp	38
Number of scaffolds larger than 100,000 bp	14
Number of scaffolds	91
Total assembled length (bp)	5,466,515
G+C contents (%)	62.4
N50 (bp)	249,384
Minimum length of scaffold (bp)	1,015
Maximum length of scaffold (bp)	733,566
Total Ns included in the draft genome	2,158

Supplementary Table 2. The list of gene annotation and its functional categorization in *Pseudoruegeria* sp. M32A2M

Gene id	Prokaryotic Genome Annotation Pipeline	Functional categorization		
	Annotation	GO	KEGG	COG
FPS10_00005	IS481 family transposase			
FPS10_00010	EAL domain-containing protein			T
FPS10_00015	HAMP domain-containing protein		K03406	NT
FPS10_00020	DUF2254 domain-containing protein			S
FPS10_00025	glucose 1-dehydrogenase	GO:0004316,GO:0008152,GO:0016491,GO:0055114	K00059	IQR
FPS10_00030	FadR family transcriptional regulator			K
FPS10_00035	TRAP transporter substrate-binding protein	GO:0006810,GO:0030288		G
FPS10_00040	TRAP transporter small permease			G
FPS10_00045	TRAP transporter large permease	GO:0016020,GO:0016021		G
FPS10_00050	GNAT family N-acetyltransferase	GO:0008080,GO:0008152,GO:0016740		J
FPS10_00055	hypothetical protein			
FPS10_00060	50S ribosomal protein L27		K02899	J
FPS10_00065	50S ribosomal protein L21		K02888	J
FPS10_00070	DUF2059 domain-containing protein			
FPS10_00075				
FPS10_00080	GTPase ObgE	GO:0000166,GO:0000287,GO:0003924,GO:0005525,GO:0005737,GO:0008152,GO:0016787,GO:0042254,GO:0046872	K03979	R
FPS10_00085	glutamate 5-kinase		K00931	E
FPS10_00090	glutamate-5-semialdehyde dehydrogenase	GO:0004350,GO:0005737,GO:0006561,GO:0008152,GO:0008652,GO:0016491,GO:0016620,GO:0050661,GO:0055114,GO:0055129	K00147	E
FPS10_00095	histidine phosphotransferase	GO:0008152,GO:0016740	K13588	S
FPS10_00100	DUF3553 domain-containing protein			
FPS10_00105	GNAT family N-acetyltransferase	GO:0008152,GO:0016740,GO:0016746		R
FPS10_00110	1-acyl-sn-glycerol-3-phosphate acyltransferase	GO:0008152,GO:0016020,GO:0016021,GO:0016740,GO:0016746	K22617	I
FPS10_00115	thiamine phosphate synthase	GO:0003824,GO:0004789,GO:0009228	K00788	H
FPS10_00120	RNA methyltransferase		K02533	J
FPS10_00125	heme A synthase	GO:0005886,GO:0006783,GO:0006784,GO:0016020,GO:0016021,GO:0016491,GO:0016627,GO:0055114	K02259	O
FPS10_00130	carboxypeptidase M32	GO:0004180,GO:0004181,GO:0006508,GO:0016787	K01299	E
FPS10_00135	OsmC family protein	GO:0004601,GO:0006979,GO:0098869	K04063	O
FPS10_00140	branched-chain amino acid ABC transporter permease	GO:0005215,GO:0005886,GO:0006810,GO:0016020,GO:0016021	K01998	E
FPS10_00145	branched-chain amino acid ABC transporter permease	GO:0005215,GO:0005886,GO:0006810,GO:0016020,GO:0016021	K01997	E
FPS10_00150	ABC transporter ATP-binding protein		K01996	E
FPS10_00155	hypothetical protein			
FPS10_00160	ABC transporter ATP-binding protein	GO:0000166,GO:0005524,GO:0008152,GO:0016887	K01995	E
FPS10_00165	ABC transporter substrate-binding protein		K01999	E
FPS10_00170	PQQ-dependent sugar dehydrogenase	GO:0003824,GO:0005975,GO:0016901,GO:0048038,GO:0055114	K21430	G
FPS10_00175	GlxA family transcriptional regulator			K
FPS10_00180	3-deoxy-7-phosphoheptulonate synthase class II	GO:0003849,GO:0009073,GO:0016740	K01626	E
FPS10_00185	PAS domain-containing protein			S
FPS10_00190	YicC family protein			S
FPS10_00195	guanylate kinase	GO:0000166,GO:0004385,GO:0005524,GO:0005737,GO:0006163,GO:0016301,GO:0016310,GO:0016740,GO:0046037,GO:0046710	K00942	F
FPS10_00200	gamma carbonic anhydrase family protein			R
FPS10_00205	two-component sensor histidine kinase		K07636	T
FPS10_00210	phosphonate ABC transporter substrate-binding protein		K02040	P
FPS10_00215	phosphate ABC transporter permease subunit PstC	GO:0005315,GO:0005886,GO:0006810,GO:0006817,GO:0016020,GO:0016021,GO:0055085	K02037	P
FPS10_00220	phosphate ABC transporter permease PstA		K02038	P
FPS10_00225	phosphate ABC transporter ATP-binding protein	GO:0000166,GO:0005315,GO:0005524,GO:0005886,GO:0006810,GO:0006817,GO:0008152,GO:0015415,GO:0016020,GO:0016787,GO:0016887,GO:0035435,GO:0043190	K02036	P
FPS10_00230	phosphate signaling complex protein PhoU		K02039	P
FPS10_00235	phosphate regulon transcriptional regulatory protein PhoB	GO:0000156,GO:0000160,GO:0003677,GO:0005622,GO:0006351,GO:0006355,GO:0006817	K07657	TK
FPS10_00240	DksA/TraR family C4-type zinc finger protein	GO:0008270		T
FPS10_00245	class I SAM-dependent methyltransferase			H
FPS10_00250	FAD-binding oxidoreductase	GO:0016020,GO:0016021,GO:0016491,GO:0055114		E
FPS10_00255	type II 3-dehydroquinate dehydratase	GO:0003855,GO:0008652,GO:0009073,GO:0009423,GO:0016829	K03786	E
FPS10_00260	LuxR family transcriptional regulator	GO:0003677,GO:0006351,GO:0006355		TK
FPS10_00265	elongation factor Ts	GO:0003746,GO:0005622,GO:0005737,GO:0006412,GO:0006414	K02357	J
FPS10_00270	30S ribosomal protein S2	GO:0003735,GO:0005622,GO:0005840,GO:0006412,GO:0015935,GO:0030529	K02967	J
FPS10_00275	cupin domain-containing protein	GO:0008152,GO:0016853		G
FPS10_00280	kinase	GO:0016301,GO:0016310,GO:0016740,GO:0016773		G

FPS10_00285	pseudouridine-5'-phosphate glycosidase		K16329	Q
FPS10_00290	DUF502 domain-containing protein	GO:0016020,GO:0016021		S
FPS10_00295	patatin-like phospholipase family protein	GO:0006629,GO:0008152	K07001	R
FPS10_00300	3-hydroxybutyrate dehydrogenase	GO:0003858,GO:0008152,GO:0016491,GO:0055114	K00019	IQR
FPS10_00305	ABC transporter substrate-binding protein	GO:0043190,GO:0055085	K02035	E
FPS10_00310	hypothetical protein			
FPS10_00315	rhodanese-like domain-containing protein	GO:0008152,GO:0016740		P
FPS10_00320	30S ribosomal protein S9	GO:0003735,GO:0005840,GO:0006412,GO:0030529	K02996	J
FPS10_00325	50S ribosomal protein L13	GO:0003735,GO:0005840,GO:0006412,GO:0030529	K02871	J
FPS10_00330	Rne/Rng family ribonuclease	GO:0000287,GO:0003676,GO:0003723,GO:0004518,GO:0004519,GO:0004521,GO:0004540,GO:0005737,GO:0005886,GO:0006364,GO:0006396,GO:0006402,GO:0008033,GO:0008270,GO:0008995,GO:0009898,GO:0016020,GO:0016787,GO:0046872,GO:0090305,GO:0090501,GO:0090502	K08300	J
FPS10_00335	HAD family phosphatase			R
FPS10_00340	sigma-54-dependent Fis family transcriptional regulator	GO:0000160,GO:0000166,GO:0003677,GO:0005524,GO:0005622,GO:0006351,GO:0006355,GO:0008134,GO:0043565		T
FPS10_00345	sensor histidine kinase		K10125	T
FPS10_00350	phosphoribosylformylglycinamidine synthase subunit PurQ		K23265	F
FPS10_00355	zinc-dependent alcohol dehydrogenase family protein	GO:0004022,GO:0008270,GO:0016491,GO:0046872,GO:00551[illegible]4		R
FPS10_00360	phosphoribosylformylglycinamidine synthase subunit PurS	GO:0000166,GO:0004642,GO:0005524,GO:0005737,GO:0006164,GO:0006189,GO:0016874	K23264	F
FPS10_00365	phosphoribosylaminoimidazolesuccinocarboxamide synthase	GO:0000166,GO:0004639,GO:0005524,GO:0006164,GO:0006189,GO:0016874	K01923	F
FPS10_00370	hypothetical protein			
FPS10_00375	DUF1476 domain-containing protein	GO:0003674,GO:0005575,GO:0008150		S
FPS10_00380	betaine--homocysteine S-methyltransferase	GO:0008168,GO:0016740,GO:0032259	K00548	E
FPS10_00385	PA0069 family radical SAM protein	GO:0003824,GO:0008152,GO:0051536		L
FPS10_00390	cobalamin-binding protein	GO:0008168,GO:0016740,GO:0031419,GO:0032259,GO:00468[illegible]2		R
FPS10_00395	hypothetical protein			
FPS10_00400	DUF1638 domain-containing protein			
FPS10_00405	GNAT family N-acetyltransferase		K03830	R
FPS10_00410	SufE family protein		K02426	R
FPS10_00415	hypothetical protein			
FPS10_00420	ribonuclease D	GO:0000166,GO:0003676,GO:0003824,GO:0004518,GO:0004527,GO:0005622,GO:0005737,GO:0006139,GO:0008033,GO:0008408,GO:0016787,GO:0033890,GO:0042780,GO:0044237,GO:0090305,GO:0090503	K03684	J
FPS10_00425	phosphoribosylglycinamide formyltransferase	GO:0004644,GO:0006164,GO:0006189,GO:0009058,GO:0016740,GO:0016742	K11175	F
FPS10_00430	phosphoribosylformylglycinamidine cyclo-ligase	GO:0000166,GO:0004641,GO:0005524,GO:0005737,GO:0006164,GO:0006189,GO:0016874	K01933	F
FPS10_00435				
FPS10_00440	hypothetical protein			
FPS10_00445	isoleucine--tRNA ligase	GO:0000166,GO:0002161,GO:0004812,GO:0004822,GO:0005524,GO:0005737,GO:0006412,GO:0006418,GO:0006428,GO:0006450,GO:0008270,GO:0016874,GO:0046872	K01870	J
FPS10_00450	hypothetical protein			
FPS10_00455	competence protein TfoX			
FPS10_00460	AbrB family transcriptional regulator	GO:0004497,GO:0010468,GO:0016020,GO:0016021,GO:00551[illegible]4	K07120	R
FPS10_00465	phosphoribosyl-AMP cyclohydrolase	GO:0000105,GO:0000287,GO:0004635,GO:0005737,GO:0008270,GO:0008652,GO:0016787,GO:0046872	K01496	E
FPS10_00470	iron-sulfur cluster assembly scaffold protein	GO:0005506,GO:0016226,GO:0051536		C
FPS10_00475	sterol-binding protein			I
FPS10_00480	U32 family peptidase	GO:0006508,GO:0008233	K08303	O
FPS10_00485	U32 family peptidase	GO:0006508,GO:0008233		O
FPS10_00490	cation-transporting P-type ATPase	GO:0000166,GO:0005388,GO:0005524,GO:0008152,GO:0016020,GO:0016021,GO:0016787,GO:0046872,GO:0070588	K01537	P
FPS10_00495	acyl carrier protein			IQ
FPS10_00500	2-oxo acid dehydrogenase subunit E2	GO:0008152,GO:0016740,GO:0016746	K00627	C
FPS10_00505	pyruvate dehydrogenase (acetyl-transferring) E1 component subunit alpha		K11381	C
FPS10_00510	acetate--CoA ligase	GO:0003824,GO:0003987,GO:0008152,GO:0016020,GO:0016021,GO:0016874	K01895	I
FPS10_00515	hypothetical protein	GO:0051536		C
FPS10_00520	cyclic nucleotide-binding domain-containing protein			T
FPS10_00525	Ni/Fe hydrogenase subunit gamma			HC
FPS10_00530	oxidoreductase			C
FPS10_00535	Ni/Fe hydrogenase subunit alpha	GO:0008901,GO:0016151,GO:0016491,GO:0046872,GO:00551[illegible]4		C

FPS10_00540	hydrogenase maturation protease		K03605	C
FPS10_00545	phosphoenolpyruvate synthase		K01007	G
FPS10_00550	cadmium-translocating P-type ATPase			P
FPS10_00555	SDR family oxidoreductase			IQR
FPS10_00560	multidrug efflux RND transporter permease subunit	GO:0005215,GO:0006810,GO:0016020,GO:0016021		V
FPS10_00565	efflux RND transporter periplasmic adaptor subunit		K03585	M
FPS10_00570	hypothetical protein			
FPS10_00575	RlmE family RNA methyltransferase	GO:0001510,GO:0005737,GO:0006364,GO:0008168,GO:0008650,GO:0016740,GO:0031167,GO:0032259	K02427	J
FPS10_00580	Ppx/GppA family phosphatase		K01524	FP
FPS10_00585	hypothetical protein			
FPS10_00590	methylenetetrahydrofolate reductase		K00297	E
FPS10_00595	HAD-IA family hydrolase	GO:0008152,GO:0016787		R
FPS10_00600	methyltetrahydrofolate cobalamin methyltransferase	GO:0042558,GO:0044237	K00548	E
FPS10_00605	serine--tRNA ligase	GO:0000166,GO:0004812,GO:0004828,GO:0005524,GO:0005737,GO:0006412,GO:0006418,GO:0006434,GO:0016260,GO:0016874,GO:0097056	K01875	J
FPS10_00610	EF-hand domain-containing protein	GO:0005509		
FPS10_00615	ribosome biogenesis GTPase Der	GO:0000166,GO:0005525,GO:0042254	K03977	R
FPS10_00620	PQQ-binding-like beta-propeller repeat protein			S
FPS10_00625	hypothetical protein			S
FPS10_00630	efflux RND transporter periplasmic adaptor subunit	GO:0016020,GO:0055085	K18990	M
FPS10_00635	efflux RND transporter permease subunit	GO:0005215,GO:0006810,GO:0016020,GO:0016021	K18989	V
FPS10_00640	porin family protein		K16079	M
FPS10_00645	amidohydrolase			R
FPS10_00650	winged helix-turn-helix transcriptional regulator			K
FPS10_00655	DUF3422 domain-containing protein	GO:0016020,GO:0016021		S
FPS10_00660	NAD(P)-dependent oxidoreductase	GO:0004616,GO:0016491,GO:0051287,GO:0055114	K00020	I
FPS10_00665	LPS export ABC transporter permease LptF	GO:0016020,GO:0016021,GO:0043190,GO:0055085	K07091	R
FPS10_00670	LPS export ABC transporter permease LptG	GO:0016020,GO:0016021,GO:0043190,GO:0055085	K11720	R
FPS10_00675	LPS-assembly protein LptD		K04744	M
FPS10_00680	peptidylprolyl isomerase		K03771	O
FPS10_00685	4-hydroxythreonine-4-phosphate dehydrogenase PdxA		K00097	H
FPS10_00690	16S rRNA (adenine(1518)-N(6)/adenine(1519)-N(6))-dimethyltransferase RsmA	GO:0000154,GO:0000179,GO:0003723,GO:0005737,GO:0006364,GO:0008168,GO:0008649,GO:0016433,GO:0016740,GO:0031167,GO:0032259,GO:0052908	K02528	J
FPS10_00695	DUF4188 domain-containing protein			
FPS10_00700	DUF808 domain-containing protein		K09781	S
FPS10_00705	pantetheine-phosphate adenylyltransferase	GO:0000166,GO:0003824,GO:0004595,GO:0005524,GO:0005737,GO:0009058,GO:0015937,GO:0016740,GO:0016779	K00954	H
FPS10_00710	CBS domain-containing protein			R
FPS10_00715	LysR family transcriptional regulator	GO:0003677,GO:0003700,GO:0006351,GO:0006355		K
FPS10_00720	CoA-acylating methylmalonate-semialdehyde dehydrogenase	GO:0004491,GO:0008152,GO:0016491,GO:0016620,GO:0055114	K00140	C
FPS10_00725	acyl-CoA dehydrogenase			I
FPS10_00730	enoyl-CoA hydratase/isomerase family protein	GO:0008152,GO:0016836,GO:0016853		I
FPS10_00735	3-hydroxyisobutyrate dehydrogenase		K00020	I
FPS10_00740	AI-2E family transporter			R
FPS10_00745	Sec-independent protein translocase TatA	GO:0005886,GO:0005887,GO:0006810,GO:0008320,GO:0008565,GO:0009306,GO:0015031,GO:0016020,GO:0016021,GO:0033281,GO:0043953	K03116	U
FPS10_00750	twin-arginine translocase subunit TatB	GO:0005886,GO:0005887,GO:0006810,GO:0008320,GO:0008565,GO:0009306,GO:0015031,GO:0016020,GO:0016021,GO:0033281,GO:0043953	K03117	U
FPS10_00755	twin-arginine translocase subunit TatC	GO:0005886,GO:0005887,GO:0006810,GO:0008320,GO:0015031,GO:0016020,GO:0016021,GO:0033281,GO:0043953	K03118	U
FPS10_00760	ATP-binding protein		K06923	R
FPS10_00765	hypothetical protein			
FPS10_00770	LysM peptidoglycan-binding domain-containing M23 family metallopeptidase			M
FPS10_00775	protein-L-isoaspartate(D-aspartate) O-methyltransferase		K00573	O
FPS10_00780	5'/3'-nucleotidase SurE	GO:0000166,GO:0005737,GO:0008152,GO:0008252,GO:0008253,GO:0016311,GO:0016787,GO:0046872	K03787	R
FPS10_00785	SDR family oxidoreductase	GO:0008152,GO:0016491,GO:0055114		R
FPS10_00790	pilus assembly protein PilP			
FPS10_00795	SLC13 family permease			P

FPS10_00800	amidophosphoribosyltransferase	GO:0000287,GO:0004044,GO:0006164,GO:0006189,GO:0006541,GO:0009113,GO:0016740,GO:0016757,GO:0046872,GO:0051536,GO:0051539	K00764	F
FPS10_00805	CvpA family protein		K03558	R
FPS10_00810	DNA repair protein RadA	GO:0000166,GO:0003677,GO:0003684,GO:0005524,GO:0006281,GO:0006974,GO:0008094,GO:0046872	K04485	O
FPS10_00815	paraquat-inducible protein A	GO:0016020,GO:0016021		S
FPS10_00820	SDR family oxidoreductase	GO:0008152,GO:0016491,GO:0055114	K00059	IQR
FPS10_00825	hypothetical protein			
FPS10_00830	ParA family protein		K03496	D
FPS10_00835	hypothetical protein			
FPS10_00840	hypothetical protein			
FPS10_00845	L%2CD-transpeptidase	GO:0008152,GO:0016740		S
FPS10_00850	glycosyltransferase family 2 protein			R
FPS10_00855	carboxylate-amine ligase	GO:0000166,GO:0004357,GO:0005524,GO:0016874,GO:0016879,GO:0042398	K06048	S
FPS10_00860	hypothetical protein			
FPS10_00865	hypothetical protein			S
FPS10_00870	L%2CD-transpeptidase			S
FPS10_00875	Hsp70 family protein		K04046	O
FPS10_00880	peptide chain release factor 3	GO:0000166,GO:0003747,GO:0003924,GO:0005525,GO:0005737,GO:0006412,GO:0006415,GO:0006449,GO:0016149	K02837	J
FPS10_00885	phytoene synthase	GO:0009058,GO:0016740		I
FPS10_00890	class I SAM-dependent methyltransferase	GO:0008168,GO:0032259		R
FPS10_00895	citramalate synthase	GO:0003824,GO:0003852,GO:0008652,GO:0009082,GO:0009098,GO:0016740,GO:0019752,GO:0046912	K01649	E
FPS10_00900	cysteine--tRNA ligase		K01883	J
FPS10_00905	MmcQ/YjbR family DNA-binding protein			S
FPS10_00910	selenide%2C water dikinase SelD		K01008	E
FPS10_00915	Gfo/Idh/MocA family oxidoreductase	GO:0008152,GO:0016491,GO:0055114		R
FPS10_00920	threonine dehydratase		K01754	E
FPS10_00925	pyridoxal phosphate-dependent aminotransferase	GO:0003824,GO:0004069,GO:0008483,GO:0009058,GO:0016740,GO:0030170,GO:0080130	K12252	E
FPS10_00930	hypothetical protein	GO:0016020,GO:0016021	K07034	S
FPS10_00935	porin family protein			M
FPS10_00940	ATP-binding cassette domain-containing protein		K15738	R
FPS10_00945	GNAT family N-acetyltransferase	GO:0008152,GO:0016740,GO:0016746		R
FPS10_00950	peptide-methionine (R)-S-oxide reductase MsrB	GO:0006979,GO:0016491,GO:0016671,GO:0030091,GO:0033743,GO:0055114	K07305	O
FPS10_00955	outer membrane protein assembly factor BamE	GO:0019867		J
FPS10_00960	DUF177 domain-containing protein			R
FPS10_00965	50S ribosomal protein L32	GO:0003735,GO:0005840,GO:0006412,GO:0015934,GO:0030529	K02911	J
FPS10_00970	phosphate acyltransferase PlsX	GO:0003824,GO:0005737,GO:0006629,GO:0006633,GO:0006644,GO:0008654,GO:0016616,GO:0016740,GO:0016747,GO:0055114	K03621	I
FPS10_00975	ketoacyl-ACP synthase III	GO:0003824,GO:0004315,GO:0005737,GO:0006629,GO:0006631,GO:0006633,GO:0008152,GO:0016740,GO:0016746,GO:0033818	K00648	I
FPS10_00980	integration host factor subunit alpha	GO:0003677,GO:0006310,GO:0006351,GO:0006355,GO:0006417	K04764	L
FPS10_00985	MerR family transcriptional regulator	GO:0003677,GO:0006355		K
FPS10_00990				
FPS10_00995	2'-deoxycytidine 5'-triphosphate deaminase		K01494	F
FPS10_01000	cytochrome B			C
FPS10_01005	tetrathionate reductase family octaheme c-type cytochrome	GO:0016020,GO:0016021		
FPS10_01010	carbohydrate porin		K02024	G
FPS10_01015	SMC-Scp complex subunit ScpB	GO:0005737,GO:0007049,GO:0007059,GO:0051301,GO:0051304	K06024	K
FPS10_01020	segregation/condensation protein A		K05896	S
FPS10_01025	beta-N-acetylhexosaminidase	GO:0004553,GO:0004563,GO:0005975,GO:0008152,GO:0016787,GO:0016798	K01207	G
FPS10_01030	SPOR domain-containing protein			S
FPS10_01035	arginine--tRNA ligase	GO:0000166,GO:0004812,GO:0004814,GO:0005524,GO:0005737,GO:0006412,GO:0006418,GO:0006420,GO:0016874	K01887	J
FPS10_01040	deoxyguanosinetriphosphate triphosphohydrolase	GO:0000287,GO:0008832,GO:0016787,GO:0046039	K01129	F
FPS10_01045	iron-sulfur cluster assembly accessory protein			S
FPS10_01050	two pore domain potassium channel family protein			
FPS10_01055	peptidylprolyl isomerase			O
FPS10_01060	exodeoxyribonuclease III		K01142	L
FPS10_01065	SLC13 family permease	GO:0006813,GO:0008324,GO:0016020,GO:0016021,GO:0055085,GO:0098655		P
FPS10_01070	hypothetical protein			O

FPS10_01075	vitamin B12-dependent ribonucleotide reductase	GO:0000166,GO:0004748,GO:0006260,GO:0016491,GO:0031419,GO:0050897,GO:0055114	K00525	F
FPS10_01080	choline dehydrogenase	GO:0008812,GO:0016491,GO:0016614,GO:0019285,GO:0050660,GO:0055114	K00108	E
FPS10_01085	betaine-aldehyde dehydrogenase		K00130	C
FPS10_01090	transcriptional regulator BetI	GO:0003677,GO:0003700,GO:0006351,GO:0006355,GO:0019285,GO:0045892	K02167	K
FPS10_01095	choline ABC transporter substrate-binding protein		K02002	E
FPS10_01100	choline ABC transporter permease subunit		K02001	E
FPS10_01105	choline ABC transporter ATP-binding protein	GO:0000166,GO:0005524,GO:0008152,GO:0015220,GO:0015871,GO:0016887,GO:0055052	K02000	E
FPS10_01110	NADH-quinone oxidoreductase subunit F	GO:0008137,GO:0010181,GO:0046872,GO:0051536,GO:0051539,GO:0055114	K22515	C
FPS10_01115	periplasmic heavy metal sensor			
FPS10_01120	formate dehydrogenase subunit alpha	GO:0008863,GO:0009055,GO:0015942,GO:0016491,GO:0030151,GO:0046872,GO:0051536,GO:0051539,GO:0055114	K00123	R
FPS10_01125	hypothetical protein			R
FPS10_01130	NADP-dependent isocitrate dehydrogenase		K00031	C
FPS10_01135	UDP-glucose--hexose-1-phosphate uridylyltransferase	GO:0003824,GO:0005975,GO:0006012,GO:0008108,GO:0008270,GO:0016740,GO:0016779,GO:0046872	K00965	C
FPS10_01140	galactokinase		K00849	G
FPS10_01145	SDR family NAD(P)-dependent oxidoreductase			R
FPS10_01150	ATP-dependent DNA helicase RecG	GO:0000166,GO:0003676,GO:0004003,GO:0004386,GO:0005524,GO:0006281,GO:0006310,GO:0006974,GO:0016787,GO:0032508	K03655	LK
FPS10_01155	NAD-dependent DNA ligase LigA		K01972	L
FPS10_01160	response regulator transcription factor	GO:0000160,GO:0003677,GO:0005622,GO:0006351,GO:0006355	K13584	TK
FPS10_01165	DUF1153 domain-containing protein			
FPS10_01170	tRNA 2-thiouridine(34) synthase MnmA	GO:0000049,GO:0000166,GO:0003723,GO:0005524,GO:0005737,GO:0006400,GO:0008033,GO:0008168,GO:0016740,GO:0016783,GO:0032259	K00566	J
FPS10_01175	N-acyl homoserine lactone synthase		K22745	C
FPS10_01180	lipid-A-disaccharide synthase	GO:0008915,GO:0009245,GO:0016740,GO:0016757	K00748	M
FPS10_01185	LpxI family protein		K09949	S
FPS10_01190	acyl-ACP--UDP-N-acetylglucosamine O-acyltransferase	GO:0005737,GO:0006629,GO:0008610,GO:0008780,GO:0009245,GO:0016740,GO:0016746	K00677	M
FPS10_01195	3-hydroxyacyl-ACP dehydratase FabZ	GO:0005737,GO:0006629,GO:0006633,GO:0009245,GO:0016829,GO:0016836,GO:0019171,GO:0047451	K02372	I
FPS10_01200	OmpH family outer membrane protein			
FPS10_01205	outer membrane protein assembly factor BamA	GO:0009279,GO:0016020,GO:0016021,GO:0019867,GO:0043165,GO:0051205,GO:0071709	K07277	M
FPS10_01210	RIP metalloprotease RseP	GO:0004222,GO:0005886,GO:0006508,GO:0008233,GO:0008237,GO:0016020,GO:0016021,GO:0016787,GO:0046872	K11749	M
FPS10_01215	1-deoxy-D-xylulose-5-phosphate reductoisomerase	GO:0008299,GO:0016114,GO:0016491,GO:0016853,GO:0019288,GO:0030604,GO:0046872,GO:0055114,GO:0070402	K00099	I
FPS10_01220	phosphatidate cytidylyltransferase	GO:0004605,GO:0016020,GO:0016021,GO:0016024,GO:0016740,GO:0016772,GO:0016779	K00981	I
FPS10_01225	isoprenyl transferase		K00806	I
FPS10_01230	ribosome recycling factor		K02838	J
FPS10_01235	PEP-CTERM sorting domain-containing protein			
FPS10_01240	UMP kinase		K09903	F
FPS10_01245	tRNA (adenosine(37)-N6)-dimethylallyltransferase MiaA	GO:0000166,GO:0005524,GO:0008033,GO:0016740,GO:0052381	K00791	J
FPS10_01250	helix-turn-helix transcriptional regulator	GO:0003677,GO:0003700,GO:0006351,GO:0006355,GO:0043565	K18954	K
FPS10_01265	ABC transporter permease	GO:0005886,GO:0006810,GO:0016020,GO:0016021	K02034	EP
FPS10_01270	hypothetical protein			
FPS10_01280	hypothetical protein			
FPS10_01285	acyl carrier protein	GO:0000036,GO:0005737,GO:0006629,GO:0006631,GO:0006633	K02078	IQ
FPS10_01290	3-oxoacyl-[acyl-carrier-protein] reductase		K00059	IQR
FPS10_01295	Hly-III family protein	GO:0016020,GO:0016021,GO:0019835	K11068	R
FPS10_01300	long-chain fatty acid--CoA ligase		K01897	I
FPS10_01305	hypothetical protein			
FPS10_01310	crossover junction endodeoxyribonuclease RuvC	GO:0000287,GO:0003676,GO:0004518,GO:0004520,GO:0006281,GO:0006310,GO:0006974,GO:0008821,GO:0016787,GO:0046872,GO:0090305	K01159	L
FPS10_01315	Holliday junction branch migration protein RuvA	GO:0000166,GO:0003677,GO:0003678,GO:0004386,GO:0005524,GO:0006281,GO:0006310,GO:0006974,GO:0009378,GO:0009379,GO:0009432,GO:0016787,GO:0032508	K03550	L
FPS10_01320	Holliday junction branch migration DNA helicase RuvB	GO:0000166,GO:0003677,GO:0004386,GO:0005524,GO:0006281,GO:0006310,GO:0006974,GO:0009378,GO:0009432,GO:0016787,GO:0032508	K03551	L
FPS10_01325	hypothetical protein			

FPS10_01330	50S ribosomal protein L11 methyltransferase	GO:0005737,GO:0005840,GO:0006479,GO:0008168,GO:0008276,GO:0016740,GO:0032259	K02687	J
FPS10_01335	AAA family ATPase			F
FPS10_01340	peptide-methionine (S)-S-oxide reductase MsrA	GO:0006464,GO:0006979,GO:0008113,GO:0016491,GO:0016671,GO:0030091,GO:0055114	K07304	O
FPS10_01345	MFS transporter	GO:0016020,GO:0016021,GO:0055085		
FPS10_01350	YeeE/YedE family protein	GO:0016020,GO:0016021		
FPS10_01355	NAD(P)/FAD-dependent oxidoreductase		K11816	P
FPS10_01360	primosomal protein N'	GO:0000166,GO:0003676,GO:0003677,GO:0004003,GO:0004386,GO:0005524,GO:0006260,GO:0006268,GO:0006269,GO:0008270,GO:0016787,GO:0032508,GO:0046872,GO:1990077	K04066	L
FPS10_01370	DUF484 family protein		K09921	S
FPS10_01375	tyrosine recombinase XerC		K03733	L
FPS10_01380	hypothetical protein			
FPS10_01385	phosphatidylcholine synthase	GO:0005886,GO:0006629,GO:0008654,GO:0016020,GO:0016021,GO:0016740,GO:0016780,GO:0050520	K01004	I
FPS10_01390	tRNA pseudouridine(38-40) synthase TruA	GO:0001522,GO:0003723,GO:0008033,GO:0009451,GO:0009982,GO:0016853,GO:0031119	K06173	J
FPS10_01395	YcjX family protein		K06918	R
FPS10_01400	TIGR01620 family protein	GO:0005886,GO:0016020,GO:0016021	K08990	S
FPS10_01405	PaaI family thioesterase			Q
FPS10_01410	component of SufBCD complex	GO:0016020,GO:0016021		
FPS10_01415	porphobilinogen synthase	GO:0003824,GO:0004655,GO:0006779,GO:0016829,GO:0033014,GO:0046872	K01698	H
FPS10_01420	twin-arginine translocation pathway signal			S
FPS10_01425	DUF3179 domain-containing protein			
FPS10_01430	FAD-binding oxidoreductase	GO:0016491,GO:0055114		E
FPS10_01435	peptidylprolyl isomerase	GO:0000413,GO:0003755,GO:0006457,GO:0016853		O
FPS10_01440	peptidylprolyl isomerase	GO:0000413,GO:0003755,GO:0006457,GO:0016853		O
FPS10_01445	phosphoglycerate kinase	GO:0000166,GO:0004618,GO:0005524,GO:0005737,GO:0006096,GO:0016301,GO:0016310,GO:0016740	K00927	G
FPS10_01450	cation transporter	GO:0005886,GO:0006810,GO:0006812,GO:0008324,GO:0016020,GO:0016021,GO:0055085,GO:0098655		P
FPS10_01455	class I fructose-bisphosphate aldolase	GO:0003824,GO:0004332,GO:0008152,GO:0016829	K11645	G
FPS10_01460	acetolactate synthase small subunit	GO:0003984,GO:0008152,GO:0009082,GO:0016597,GO:0016740	K01653	E
FPS10_01465	fumarylacetoacetate hydrolase family protein	GO:0003824,GO:0008152,GO:0016787	K16165	Q
FPS10_01470	DsbE family thiol:disulfide interchange protein	GO:0015036,GO:0016020,GO:0016021,GO:0016491,GO:0017004,GO:0030288,GO:0045454,GO:0055114	K02199	OC
FPS10_01475	heme exporter protein CcmD	GO:0015886,GO:0016020,GO:0016021,GO:0017004	K02196	
FPS10_01480	heme ABC transporter permease		K02195	O
FPS10_01485	heme exporter protein CcmB		K02194	O
FPS10_01490	heme ABC exporter ATP-binding protein CcmA		K02193	O
FPS10_01495	hypothetical protein			S
FPS10_01500	sulfite exporter TauE/SafE family protein		K07090	R
FPS10_01505	protein translocase subunit SecF	GO:0005622,GO:0005886,GO:0006605,GO:0006810,GO:0006886,GO:0015031,GO:0015450,GO:0016020,GO:0016021,GO:0043952,GO:0065002	K03074	U
FPS10_01510	protein translocase subunit SecD	GO:0005622,GO:0005886,GO:0006605,GO:0006810,GO:0006886,GO:0015031,GO:0015450,GO:0016020,GO:0016021,GO:0043952,GO:0065002	K03072	U
FPS10_01515	preprotein translocase subunit YajC	GO:0016020,GO:0016021	K03210	U
FPS10_01520	DUF4445 domain-containing protein	GO:0009055,GO:0051536		R
FPS10_01525				
FPS10_01530	TetR/AcrR family transcriptional regulator			K
FPS10_01535	hypothetical protein		K02051	P
FPS10_01540	ABC transporter permease	GO:0005886,GO:0006810,GO:0016020,GO:0016021	K02050	P
FPS10_01545	ABC transporter ATP-binding protein	GO:0000166,GO:0005524,GO:0008152,GO:0016887	K02049	P
FPS10_01550	creatininase	GO:0006601,GO:0006602,GO:0047789	K01470	R
FPS10_01555	SDR family oxidoreductase			IQR
FPS10_01560	3-oxoacyl-ACP reductase FabG	GO:0004316,GO:0006633,GO:0008152,GO:0016491,GO:0051287,GO:0055114	K00059	IQR
FPS10_01565	agmatinase	GO:0008152,GO:0016787,GO:0016813,GO:0046872	K18459	E
FPS10_01570	GntR family transcriptional regulator			K
FPS10_01575	sulfite exporter TauE/SafE family protein	GO:0016020,GO:0016021		R
FPS10_01580	gamma-glutamyltransferase		K00681	E
FPS10_01585	TRAP transporter large permease subunit	GO:0016020,GO:0016021		Q
FPS10_01590	TRAP transporter small permease subunit			Q
FPS10_01595	hypothetical protein	GO:0006810,GO:0030288,GO:0042597,GO:0046872		Q

FPS10_01600	cytochrome c3 family protein			
FPS10_01605	hypothetical protein			
FPS10_01610	cytochrome B			C
FPS10_01615	hypothetical protein			
FPS10_01625	hypothetical protein			K
FPS10_01630	hypothetical protein			
FPS10_01635	cold-shock protein	GO:0003676,GO:0003677,GO:0005737,GO:0006351,GO:00063[illegible]5	K03704	K
FPS10_01640	hypothetical protein			
FPS10_01645	chromosome partitioning protein ParB			K
FPS10_01650	transglutaminase family protein			E
FPS10_01655	DUF4102 domain-containing protein	GO:0003677,GO:0006310,GO:0015074		L
FPS10_01660	sel1 repeat family protein			R
FPS10_01670	IS110 family transposase	GO:0003677,GO:0004803,GO:0006313		
FPS10_01680	hypothetical protein			
FPS10_01685	mechanosensitive ion channel			T
FPS10_01690	DUF2493 domain-containing protein			
FPS10_01695	response regulator			T
FPS10_01700	response regulator			T
FPS10_01705	ammonium transporter		K03320	P
FPS10_01710	hypothetical protein			
FPS10_01715	response regulator			T
FPS10_01720	hypothetical protein			S
FPS10_01730	IS3 family transposase		K07497	L
FPS10_01735	plasmid pRiA4b ORF-3 family protein			
FPS10_01740	plasmid maintenance protein CcdB		K19163	
FPS10_01745	hypothetical protein		K19164	R
FPS10_01750	VanZ family protein			
FPS10_01755	IS21 family transposase			L
FPS10_01760	ATP-binding protein	GO:0005524		L
FPS10_01765	inositol 2-dehydrogenase	GO:0008152,GO:0016491,GO:0055114	K00010	R
FPS10_01770	class II fructose-bisphosphate aldolase	GO:0003824,GO:0004332,GO:0005975,GO:0008270,GO:0016829,GO:0016832,GO:0046872	K01624	G
FPS10_01775	5-deoxy-glucuronate isomerase	GO:0008880,GO:0016853,GO:0016861,GO:0019310	K03337	G
FPS10_01780	5-dehydro-2-deoxygluconokinase	GO:0016301,GO:0016310,GO:0016740,GO:0016773	K03338	G
FPS10_01785	3D-(3%2C5/4)-trihydroxycyclohexane-1%2C2-dione acylhydrolase (decyclizing)	GO:0000287,GO:0003824,GO:0016823,GO:0019310,GO:0030976	K03336	E
FPS10_01795	TIM barrel protein		K03335	G
FPS10_01800	Gfo/Idh/MocA family oxidoreductase	GO:0008152,GO:0016491,GO:0055114		R
FPS10_01805	LacI family transcriptional regulator	GO:0003677,GO:0003700,GO:0006351,GO:0006355		K
FPS10_01810	sugar ABC transporter substrate-binding protein	GO:0008643	K02058	G
FPS10_01815	ABC transporter permease		K02057	G
FPS10_01820	sugar ABC transporter ATP-binding protein	GO:0005524,GO:0008152,GO:0016887		G
FPS10_01830	sensor domain-containing diguanylate cyclase			T
FPS10_01835	TonB-dependent receptor	GO:0004872,GO:0006810,GO:0009279,GO:0016020	K02014	P
FPS10_01840	PepSY domain-containing protein			S
FPS10_01845	hypothetical protein			
FPS10_01850	TniQ family protein			
FPS10_01855	AAA family ATPase			
FPS10_01860	DDE-type integrase/transposase/recombinase		K07497	
FPS10_01865	EAL domain-containing protein			T
FPS10_01870	extracellular solute-binding protein		K02040	P
FPS10_01880	transposase			
FPS10_01885	PilZ domain-containing protein			
FPS10_01890	hypothetical protein			
FPS10_01895	NADH ubiquinone oxidoreductase			
FPS10_01900	zinc metalloprotease HtpX	GO:0004222,GO:0005886,GO:0006508,GO:0008233,GO:00082[illegible]7,GO:0008270,GO:0016020,GO:0016021,GO:0016787,GO:0046872	K03799	O
FPS10_01905	peptidase U32		K08303	O
FPS10_01910	phosphate acetyltransferase	GO:0005737,GO:0006085,GO:0008152,GO:0008959,GO:0016407,GO:0016740,GO:0016746	K13788	C
FPS10_01920	cytochrome c			C
FPS10_01925	N-acetylglucosamine-6-phosphate deacetylase		K01443	G
FPS10_01930	SIS domain-containing protein	GO:0005975,GO:0030246	K00820	M
FPS10_01935	GntR family transcriptional regulator	GO:0003677,GO:0003700,GO:0006351,GO:0006355	K03710	K
FPS10_01940	hypothetical protein		K18676	G
FPS10_01945	N-acetylmuramic acid 6-phosphate etherase		K07106	R
FPS10_01950	carbohydrate ABC transporter substrate-binding protein	GO:0005215,GO:0006810	K10117	G

FPS10_01955	sugar ABC transporter permease		K10118	G
FPS10_01960	carbohydrate ABC transporter permease	GO:0005886,GO:0006810,GO:0016020,GO:0016021	K10119	G
FPS10_01965	Gfo/Idh/MocA family oxidoreductase	GO:0016491,GO:0055114		R
FPS10_01970	ABC transporter ATP-binding protein	GO:0000166,GO:0005215,GO:0005524,GO:0006810,GO:0008152,GO:0016820,GO:0016887,GO:0043190,GO:0055085	K10111	G
FPS10_01975	transglutaminase family protein	GO:0006508,GO:0008233		E
FPS10_01980	hypothetical protein			S
FPS10_01985	transglutaminase family protein			E
FPS10_01990	formyltetrahydrofolate deformylase	GO:0006164,GO:0006189,GO:0006730,GO:0008152,GO:0008864,GO:0009058,GO:0016597,GO:0016742,GO:0016787	K01433	F
FPS10_01995	HlyD family efflux transporter periplasmic adaptor subunit			V
FPS10_02000	efflux RND transporter permease subunit			V
FPS10_02005	hypothetical protein			
FPS10_02010	twin-arginine translocation pathway signal protein			
FPS10_02015	nitrous oxide reductase accessory protein NosL			C
FPS10_02025	FtsX-like permease family protein	GO:0016020,GO:0016021		M
FPS10_02030	ABC transporter ATP-binding protein	GO:0005524,GO:0008152,GO:0016887	K02003	V
FPS10_02035	hypothetical protein	GO:0016020,GO:0016021		
FPS10_02040	response regulator transcription factor	GO:0000160,GO:0003677,GO:0005622,GO:0006351,GO:00063[illegible]5	K02483	TK
FPS10_02045	response regulator	GO:0000155,GO:0000160,GO:0000166,GO:0004673,GO:0004871,GO:0005524,GO:0005622,GO:0007165,GO:0016020,GO:0016021,GO:0016301,GO:0016310,GO:0016740,GO:0016772,GO:0018106,GO:0023014		T
FPS10_02050	extracellular solute-binding protein	GO:0005215,GO:0006810	K02027	G
FPS10_02055	phosphoenolpyruvate--protein phosphotransferase	GO:0003824,GO:0005215,GO:0005737,GO:0006810,GO:0009401,GO:0016301,GO:0016310,GO:0016740,GO:0016772	K02768	G
FPS10_02060	1-phosphofructokinase	GO:0000166,GO:0005524,GO:0005975,GO:0008662,GO:0016301,GO:0016310,GO:0016740,GO:0016773,GO:0046835	K00882	G
FPS10_02065	PTS fructose-like transporter subunit IIB	GO:0005351,GO:0005886,GO:0006810,GO:0008152,GO:0008643,GO:0008982,GO:0009401,GO:0015755,GO:0015992,GO:0016020,GO:0016021,GO:0016740,GO:0022877,GO:0034219	K02769	G
FPS10_02070				
FPS10_02075				
FPS10_02080	hypothetical protein	GO:0016020,GO:0016021		
FPS10_02085	hypothetical protein			
FPS10_02090	M20/M25/M40 family metallo-hydrolase	GO:0008152,GO:0016787		E
FPS10_02095	histidine phosphatase family protein			G
FPS10_02100	polyphosphate kinase 2	GO:0006793,GO:0008976,GO:0016301,GO:0016310	K22468	S
FPS10_02105	hypothetical protein			
FPS10_02110	RluA family pseudouridine synthase	GO:0001522,GO:0003723,GO:0009451,GO:0009982,GO:00168[illegible]3	K06180	J
FPS10_02115	RNA polymerase sigma factor RpoH	GO:0001123,GO:0003677,GO:0003700,GO:0005737,GO:0006351,GO:0006352,GO:0006355,GO:0006950,GO:0008270,GO:0009408,GO:0010468,GO:0016987,GO:0030435	K03089	K
FPS10_02120	hypothetical protein			
FPS10_02125				
FPS10_02130	endopeptidase La		K01338	O
FPS10_02135	tRNA guanosine(34) transglycosylase Tgt		K00773	J
FPS10_02140	EAL domain-containing protein			T
FPS10_02145	SUF system Fe-S cluster assembly protein			R
FPS10_02150	iron-sulfur cluster assembly accessory protein	GO:0005198,GO:0016226,GO:0051536	K13628	S
FPS10_02155	LysR family transcriptional regulator	GO:0003677,GO:0003700,GO:0006351,GO:0006355		K
FPS10_02160	triose-phosphate isomerase	GO:0003824,GO:0004807,GO:0005737,GO:0006094,GO:0006096,GO:0006098,GO:0008152,GO:0016853	K01803	G
FPS10_02165	ankyrin repeat domain-containing protein			R
FPS10_02170	iron ABC transporter permease	GO:0005886,GO:0006810,GO:0016020,GO:0016021	K02011	P
FPS10_02175	sulfotransferase			
FPS10_02180	extracellular solute-binding protein	GO:0046872	K02012	P
FPS10_02185	TRAP transporter large permease		K11690	G
FPS10_02190	TRAP transporter small permease	GO:0016020,GO:0016021	K11689	G
FPS10_02195	TRAP transporter substrate-binding protein	GO:0006810,GO:0030288	K11688	G
FPS10_02200	sigma-54-dependent Fis family transcriptional regulator	GO:0000160,GO:0000166,GO:0003677,GO:0005524,GO:0005622,GO:0006351,GO:0006355,GO:0008134,GO:0043565	K10126	T
FPS10_02205	sensor histidine kinase	GO:0000155,GO:0000160,GO:0000166,GO:0005524,GO:0005622,GO:0007165,GO:0015740,GO:0016020,GO:0016021,GO:0016301,GO:0016310,GO:0016740,GO:0016772,GO:0023014	K10125	T
FPS10_02210	cytochrome P450	GO:0004497,GO:0005506,GO:0016491,GO:0016705,GO:0020037,GO:0046872,GO:0055114		Q
FPS10_02215	Lrp/AsnC family transcriptional regulator	GO:0003677,GO:0003700,GO:0005622,GO:0006351,GO:0006355,GO:0043565	K05800	K

FPS10_02220	uroporphyrinogen-III C-methyltransferase	GO:0003824,GO:0004851,GO:0006779,GO:0008152,GO:000816 8,GO:0009236,GO:0016491,GO:0016740,GO:0016829,GO:00193 54,GO:0032259,GO:0043115,GO:0051266,GO:0051287,GO:0055 114	K02302	H
FPS10_02225	DUF2849 domain-containing protein			
FPS10_02230	nitrite/sulfite reductase		K00381	P
FPS10_02235	phosphoadenylyl-sulfate reductase		K00390	EH
FPS10_02240	DUF934 domain-containing protein			S
FPS10_02245	ferredoxin--NADP reductase	GO:0016491,GO:0055114	K00528	C
FPS10_02250	amylosucrase	GO:0003824,GO:0005975,GO:0016740,GO:0016757,GO:004776 9	K05341	G
FPS10_02255	hypothetical protein			
FPS10_02260	translation initiation factor IF-3	GO:0003743,GO:0005737,GO:0006412,GO:0006413	K02520	J
FPS10_02265	DUF938 domain-containing protein			
FPS10_02270	arginase		K01476	E
FPS10_02280	hypothetical protein			
FPS10_02285	phosphoadenosine phosphosulfate reductase			
FPS10_02290	urease accessory protein UreG	GO:0000166,GO:0003924,GO:0005525,GO:0005737,GO:000680 7,GO:0016151	K03189	OK
FPS10_02295	urease accessory protein UreF	GO:0005737,GO:0006807,GO:0016151	K03188	O
FPS10_02300	urease accessory protein UreE	GO:0005737,GO:0006457,GO:0006461,GO:0016151,GO:001830 7,GO:0019627,GO:0051082	K03187	O
FPS10_02305	hypothetical protein			
FPS10_02310	DUF1127 domain-containing protein			
FPS10_02315	urease subunit alpha	GO:0005737,GO:0006807,GO:0009039,GO:0016151,GO:001678 7,GO:0016810,GO:0019627,GO:0043419,GO:0046872	K01428	E
FPS10_02320	hypothetical protein			
FPS10_02325	urease subunit beta	GO:0005737,GO:0009039,GO:0016787,GO:0043419	K01429	E
FPS10_02330	hypothetical protein			
FPS10_02335	urease subunit gamma	GO:0005737,GO:0009039,GO:0016151,GO:0016787,GO:001962 7,GO:0043419	K01430	E
FPS10_02340	urease accessory protein UreD	GO:0005737,GO:0006807,GO:0016151	K03190	O
FPS10_02345	dihydroorotase	GO:0004151,GO:0006221,GO:0008270,GO:0016787,GO:001681 2,GO:0019856,GO:0044205,GO:0046872	K01465	F
FPS10_02350	orotate phosphoribosyltransferase	GO:0000287,GO:0004588,GO:0006221,GO:0009116,GO:001674 0,GO:0016757,GO:0044205	K00762	F
FPS10_02355	replicative DNA helicase	GO:0000166,GO:0003677,GO:0003678,GO:0004386,GO:000552 4,GO:0006260,GO:0006269,GO:0016787,GO:0032508,GO:19900 77	K02314	L
FPS10_02360	antibiotic biosynthesis monooxygenase			S
FPS10_02365	alanine racemase	GO:0003824,GO:0006522,GO:0008784,GO:0016853,GO:003017 0,GO:0030632	K01775	M
FPS10_02370	ABC transporter permease	GO:0005886,GO:0006810,GO:0016020,GO:0016021,GO:004319 0	K02066	Q
FPS10_02375	ABC transporter ATP-binding protein	GO:0000166,GO:0005524,GO:0008152,GO:0016887	K02065	Q
FPS10_02380	LysM peptidoglycan-binding domain-containing protein			S
FPS10_02385	TIGR00730 family Rossman fold protein	GO:0009691,GO:0016787,GO:0016799		R
FPS10_02390	EamA family transporter RarD	GO:0005215,GO:0005887,GO:0006810,GO:0016020,GO:001602 1	K05786	R
FPS10_02395	superoxide dismutase	GO:0004784,GO:0006801,GO:0016491,GO:0019430,GO:004687 2,GO:0055114	K04564	P
FPS10_02400	tetratricopeptide repeat protein			
FPS10_02405	hypothetical protein			
FPS10_02410	sarcosine oxidase subunit gamma		K00305	E
FPS10_02415	sarcosine oxidase subunit alpha family protein	GO:0008115,GO:0016491,GO:0046653,GO:0055114	K00302	E
FPS10_02420	sarcosine oxidase subunit delta	GO:0008115,GO:0016491,GO:0046653,GO:0055114	K00304	E
FPS10_02425	TfoX/Sxy family protein			
FPS10_02430	hypothetical protein			
FPS10_02435	sarcosine oxidase subunit beta family protein	GO:0008115,GO:0016491,GO:0046653,GO:0055114	K00303	E
FPS10_02440	c-type cytochrome biogenesis protein CcmI		K02200	O
FPS10_02445	Holliday junction resolvase RuvX	GO:0000967,GO:0003676,GO:0004518,GO:0005737,GO:000613 9,GO:0006364,GO:0016787,GO:0016788,GO:0042254,GO:00903 05	K07447	L
FPS10_02450	DUF1289 domain-containing protein		K06938	R
FPS10_02455	tRNA dihydrouridine(20/20a) synthase DusA		K05539	J
FPS10_02460	site-specific integrase			L
FPS10_02465	hypothetical protein			
FPS10_02470	hypothetical protein			
FPS10_02475	hypothetical protein			
FPS10_02480	hypothetical protein			
FPS10_02485	P27 family phage terminase small subunit			
FPS10_02490	hypothetical protein			
FPS10_02500	DUF4268 domain-containing protein			L
FPS10_02505	hypothetical protein			

FPS10_02510	META domain-containing protein			O
FPS10_02515	hypothetical protein			
FPS10_02520	ABC transporter ATP-binding protein			E
FPS10_02525	ABC transporter ATP-binding protein		K02031	EP
FPS10_02535	alanine:cation symporter family protein		K03310	E
FPS10_02540	universal stress protein	GO:0006950		T
FPS10_02545	IS630 family transposase			L
FPS10_02550	IS110 family transposase			L
FPS10_02555	hypothetical protein	GO:0016020,GO:0016021		Q
FPS10_02560	hypothetical protein			
FPS10_02565	IS21 family transposase			L
FPS10_02570	ATPase	GO:0005524		L
FPS10_02575	hypothetical protein	GO:0003824,GO:0008152		
FPS10_02580	iron ABC transporter permease	GO:0005886,GO:0006810,GO:0016020,GO:0016021	K02011	P
FPS10_02585	ABC transporter ATP-binding protein	GO:0000166,GO:0005215,GO:0005524,GO:0006810,GO:0008152,GO:0015408,GO:0015682,GO:0016787,GO:0016820,GO:0016887,GO:0043190,GO:0055085	K02010	E
FPS10_02590	extracellular solute-binding protein		K02012	P
FPS10_02595	histidine phosphatase family protein			G
FPS10_02600	ROK family protein		K00884	KG
FPS10_02605	SIS domain-containing protein	GO:0005975,GO:0016853,GO:0030246	K02082	M
FPS10_02610	DUF2189 domain-containing protein	GO:0016020,GO:0016021		S
FPS10_02615	hypothetical protein		K18480	R
FPS10_02620	MCE family protein		K02067	Q
FPS10_02625	ABC transporter ATP-binding protein		K02065	Q
FPS10_02630	MlaE family lipid ABC transporter permease subunit		K02066	Q
FPS10_02635	arylsulfatase		K01130	P
FPS10_02640	AraC family transcriptional regulator			K
FPS10_02655	IS110 family transposase			L
FPS10_02675	carbohydrate porin		K07267	M
FPS10_02680	AraC family transcriptional regulator			K
FPS10_02685	amidohydrolase family protein			Q
FPS10_02690	amidohydrolase family protein			Q
FPS10_02700	hypothetical protein			
FPS10_02705	ABC transporter ATP-binding protein		K02003	V
FPS10_02710	ABC transporter permease	GO:0005886,GO:0016020,GO:0016021	K02004	V
FPS10_02715	DUF3299 domain-containing protein		K09950	S
FPS10_02720	transposase		K07497	
FPS10_02725	recombinase family protein			
FPS10_02730	c-type cytochrome	GO:0009055,GO:0020037		C
FPS10_02740	hypothetical protein			
FPS10_02750	IS481 family transposase	GO:0003676,GO:0003677,GO:0015074		
FPS10_02755	hypothetical protein			
FPS10_02760	hypothetical protein	GO:0003677,GO:0004803,GO:0006313		
FPS10_02765	hypothetical protein			
FPS10_02770	tyrosine-type recombinase/integrase	GO:0003677,GO:0006310,GO:0015074		L
FPS10_02775	DUF3883 domain-containing protein			KL
FPS10_02780	hypothetical protein			
FPS10_02785	DUF1156 domain-containing protein		K07445	L
FPS10_02790	DUF499 domain-containing protein			R
FPS10_02795	hypothetical protein			
FPS10_02800	hypothetical protein			
FPS10_02805	hypothetical protein			
FPS10_02810	IS3 family transposase			L
FPS10_02815	AAA family ATPase			L
FPS10_02820	hypothetical protein			
FPS10_02825	IS66 family insertion sequence hypothetical protein	GO:0003677,GO:0004803,GO:0006313,GO:0043565	K07483	L
FPS10_02830	IS66 family insertion sequence element accessory protein TnpB		K07484	L
FPS10_02835	IS66 family transposase			L
FPS10_02840	plasmid pRiA4b ORF-3 family protein			
FPS10_02845	hypothetical protein			S
FPS10_02850	DUF1887 family protein			
FPS10_02855	hypothetical protein			
FPS10_02860	hypothetical protein			L
FPS10_02865	CRISPR-associated endonuclease Cas1		K15342	L

FPS10_02870	CRISPR-associated endonuclease Cas2	GO:0004518,GO:0004519,GO:0004521,GO:0016787,GO:0043571,GO:0046872,GO:0051607,GO:0090305,GO:0090502	K09951	L
FPS10_02875	hypothetical protein			
FPS10_02880	hypothetical protein			
FPS10_02885	hypothetical protein			
FPS10_02890	hypothetical protein			
FPS10_02895	hypothetical protein			
FPS10_02900	hypothetical protein			
FPS10_02905	hypothetical protein			
FPS10_02910	hypothetical protein			
FPS10_02915	DUF4011 domain-containing protein			L
FPS10_02920	hypothetical protein			
FPS10_02925	hypothetical protein			R
FPS10_02930	hypothetical protein			
FPS10_02935	hypothetical protein			
FPS10_02940	AlpA family phage regulatory protein		K07733	K
FPS10_02945	hypothetical protein			
FPS10_02950	hypothetical protein			
FPS10_02955	tyrosine-type recombinase/integrase			
FPS10_02960	hypothetical protein			
FPS10_02965	hypothetical protein			
FPS10_02970	glutathionylspermidine synthase family protein			E
FPS10_02975	DUF1190 domain-containing protein			S
FPS10_02980	hypothetical protein			
FPS10_02985	AraC family transcriptional regulator			K
FPS10_03005	transposase			
FPS10_03015	transposase		K07483	L
FPS10_03020	peptidoglycan-binding protein	GO:0008745,GO:0009253		M
FPS10_03025	M23 family metallopeptidase			M
FPS10_03030	peptidoglycan-binding protein			M
FPS10_03035	hypothetical protein			
FPS10_03040	hypothetical protein			
FPS10_03045	hypothetical protein			M
FPS10_03050	caspase family protein			R
FPS10_03060				
FPS10_03065	agmatine deiminase	GO:0004668,GO:0009446,GO:0016787,GO:0047632	K10536	E
FPS10_03070	alanine:cation symporter family protein		K03310	E
FPS10_03075	asparaginase		K01424	EJ
FPS10_03080	hypothetical protein			M
FPS10_03085	zinc-binding dehydrogenase	GO:0008270,GO:0016491,GO:0055114		CR
FPS10_03090	DUF3604 domain-containing protein			
FPS10_03095	HupE/UreJ family protein	GO:0016020,GO:0016021		
FPS10_03100	peptidyl-prolyl cis-trans isomerase			O
FPS10_03105	pyrroline-5-carboxylate reductase		K00286	E
FPS10_03110	PAS domain-containing protein	GO:0000155,GO:0000160,GO:0000166,GO:0005524,GO:0005622,GO:0007165,GO:0016020,GO:0016021,GO:0016301,GO:0016310,GO:0016740,GO:0016772,GO:0023014		T
FPS10_03115	response regulator transcription factor	GO:0000160,GO:0003677,GO:0005622,GO:0006351,GO:00063[illegible] 5		T
FPS10_03120	lipolytic enzyme%2C G-D-S-L			E
FPS10_03125	YhcH/YjgK/YiaL family protein			G
FPS10_03130	LacI family DNA-binding transcriptional regulator		K06145	K
FPS10_03135	hypothetical protein	GO:0004497,GO:0055114		
FPS10_03140	NAD(P)-dependent oxidoreductase	GO:0004616,GO:0016491,GO:0051287,GO:0055114	K00042	I
FPS10_03145	SDR family oxidoreductase	GO:0008152,GO:0016491,GO:0055114	K00046	IQR
FPS10_03150	2-hydroxyacid dehydrogenase	GO:0008152,GO:0016616,GO:0051287,GO:0055114		CHR
FPS10_03155	L-idonate 5-dehydrogenase	GO:0008270,GO:0016491,GO:0050572,GO:0055114	K00098	ER
FPS10_03160	murein biosynthesis integral membrane protein MurJ		K03980	R
FPS10_03165	SLC13 family permease	GO:0006813,GO:0008324,GO:0016020,GO:0016021,GO:0055085,GO:0098655		P
FPS10_03170	hypothetical protein			
FPS10_03175	response regulator			T
FPS10_03180	transposase			L
FPS10_03185	response regulator			T
FPS10_03190	hypothetical protein			
FPS10_03195	sugar ABC transporter ATP-binding protein		K10441	G
FPS10_03200	ABC transporter permease		K10440	G
FPS10_03205	sugar ABC transporter substrate-binding protein		K10543	G

FPS10_03210	ABC transporter permease		K10440	G
FPS10_03215	SDR family oxidoreductase	GO:0008152,GO:0016491,GO:0055114		IQR
FPS10_03220	dihydroxyacetone kinase subunit DhaK	GO:0004371,GO:0006071,GO:0016310	K05878	G
FPS10_03225	IS1182 family transposase			L
FPS10_03230	hypothetical protein			
FPS10_03235	recombinase family protein			L
FPS10_03240	chromosome partitioning protein ParB		K03497	K
FPS10_03245	AAA family ATPase			D
FPS10_03250	hypothetical protein		K18996	
FPS10_03255	hypothetical protein			S
FPS10_03260	IS3 family transposase			L
FPS10_03270	hypothetical protein			K
FPS10_03275	hypothetical protein			
FPS10_03280	type II toxin-antitoxin system Phd/YefM family antitoxin			
FPS10_03285	single-stranded DNA-binding protein	GO:0003677,GO:0003697,GO:0006260	K03111	L
FPS10_03290	hypothetical protein			
FPS10_03295	DUF2493 domain-containing protein			
FPS10_03300	hypothetical protein			
FPS10_03305	hypothetical protein			
FPS10_03310	hypothetical protein			
FPS10_03315	hypothetical protein			
FPS10_03320	hypothetical protein			
FPS10_03325	ribbon-helix-helix protein%2C CopG family			
FPS10_03330	conjugal transfer protein TraD	GO:0016020,GO:0016021		D
FPS10_03335	conjugative relaxase			L
FPS10_03340	hypothetical protein			
FPS10_03345	DUF2726 domain-containing protein			
FPS10_03350	hypothetical protein			
FPS10_03355	hypothetical protein			
FPS10_03360	hypothetical protein			
FPS10_03365	hypothetical protein			
FPS10_03370	hypothetical protein			
FPS10_03375	hypothetical protein			
FPS10_03380	transcriptional regulator	GO:0003677,GO:0006355,GO:0008270		K
FPS10_03385	translesion error-prone DNA polymerase V autoproteolytic subunit		K03503	KT
FPS10_03390	hypothetical protein		K03502	L
FPS10_03395	RES domain-containing protein			
FPS10_03400	DUF2384 domain-containing protein			
FPS10_03405	DUF2235 domain-containing protein	GO:0016020,GO:0016021		S
FPS10_03410	hypothetical protein			
FPS10_03415	cobalamin-binding protein		K02016	P
FPS10_03420	hypothetical protein			
FPS10_03425	TonB-dependent receptor	GO:0004872,GO:0006810,GO:0009279,GO:0016020	K16092	H
FPS10_03430	IS110 family transposase	GO:0003677,GO:0004803,GO:0006281,GO:0006313		L
FPS10_03435	IS3 family transposase			L
FPS10_03440	hypothetical protein			
FPS10_03445	hypothetical protein			
FPS10_03450	site-specific integrase	GO:0003677,GO:0006310,GO:0015074		L
FPS10_03455	helix-turn-helix transcriptional regulator			
FPS10_03460	RraA family protein	GO:0008168,GO:0016740,GO:0032259		H
FPS10_03465	PhoX family phosphatase		K07093	R
FPS10_03470	cytochrome c			C
FPS10_03475	IS3 family transposase		K07497	L
FPS10_03485	TetR family transcriptional regulator	GO:0003677,GO:0006351,GO:0006355		K
FPS10_03490	shikimate dehydrogenase		K00014	E
FPS10_03495	DctP family TRAP transporter solute-binding subunit			G
FPS10_03500	TRAP transporter small permease	GO:0016020,GO:0016021		G
FPS10_03505	TRAP transporter large permease			G
FPS10_03510	hydroxymethylglutaryl-CoA lyase		K01640	E
FPS10_03515	sugar phosphate isomerase/epimerase	GO:0008152,GO:0016853		G
FPS10_03520	aldehyde dehydrogenase family protein	GO:0008152,GO:0016491,GO:0016620,GO:0055114	K15515	C
FPS10_03525	thiamine pyrophosphate-binding protein		K01652	EH
FPS10_03530	sugar phosphate isomerase/epimerase and 4-hydroxyphenylpyruvate domain-containing protein	GO:0051213,GO:0055114	K00457	G
FPS10_03535	lipid kinase		K02051	P

FPS10_03540	ABC transporter permease subunit	GO:0005886,GO:0006810,GO:0016020,GO:0016021	K02050	P
FPS10_03545	ABC transporter ATP-binding protein	GO:0000166,GO:0005524,GO:0008152,GO:0016887	K02049	P
FPS10_03550	agmatinase	GO:0008152,GO:0016787,GO:0046872	K01480	E
FPS10_03555	hypothetical protein			
FPS10_03560	YaaA family protein		K09861	S
FPS10_03565	DNA helicase RecQ	GO:0000166,GO:0003676,GO:0003824,GO:0004003,GO:0004386,GO:0005524,GO:0005622,GO:0006260,GO:0006281,GO:0006310,GO:0008026,GO:0009432,GO:0016787,GO:0032508,GO:0043140,GO:0044237	K03654	J
FPS10_03570	YggT family protein		K02221	
FPS10_03575	acyl-CoA thioesterase			R
FPS10_03580	succinyl-diaminopimelate desuccinylase	GO:0006508,GO:0008152,GO:0008237,GO:0008270,GO:0008652,GO:0009014,GO:0009085,GO:0009089,GO:0016787,GO:0019877,GO:0046872,GO:0050897	K01439	E
FPS10_03585	glycosyltransferase	GO:0003674,GO:0005575,GO:0008150	K09931	S
FPS10_03590	ribonuclease R		K12573	K
FPS10_03595	lytic murein transglycosylase			M
FPS10_03600	amphi-Trp domain-containing protein			
FPS10_03605	HprK-related kinase B	GO:0016301,GO:0016310		
FPS10_03610	GAK system ATP-grasp enzyme	GO:0005524,GO:0008152,GO:0016740,GO:0046872	K05844	HJ
FPS10_03615	phosphotransferase			P
FPS10_03620	NUDIX domain-containing protein			F
FPS10_03625	GAK system CofD-like protein			S
FPS10_03630	NUDIX domain-containing protein			T
FPS10_03635	thiamine pyrophosphate-binding protein	GO:0000287,GO:0003824,GO:0003984,GO:0008152,GO:0016740,GO:0030976	K01652	EH
FPS10_03640	hypothetical protein			
FPS10_03645	S-(hydroxymethyl)glutathione dehydrogenase/class III alcohol dehydrogenase	GO:0006069,GO:0008270,GO:0016491,GO:0046292,GO:0046872,GO:0051903,GO:0055114	K00121	C
FPS10_03650	NYN domain-containing protein			S
FPS10_03655	lactoylglutathione lyase family protein	GO:0004462,GO:0008152,GO:0046872,GO:0051213,GO:00551[illegible]4		E
FPS10_03660	LysR family transcriptional regulator			K
FPS10_03665	TetR/AcrR family transcriptional regulator	GO:0003677,GO:0006351,GO:0006355		K
FPS10_03670	AEC family transporter	GO:0016020,GO:0016021,GO:0055085	K07088	R
FPS10_03675	S-formylglutathione hydrolase	GO:0016787,GO:0018738,GO:0046294	K01070	R
FPS10_03680	YaiI/YqxD family protein		K09768	S
FPS10_03685	HAD family phosphatase	GO:0008152,GO:0016787	K01560	R
FPS10_03690	ornithine cyclodeaminase			E
FPS10_03695	MFS transporter	GO:0016020,GO:0016021,GO:0055085		G
FPS10_03700	AFG1 family ATPase	GO:0005524	K06916	R
FPS10_03705	bifunctional folylpolyglutamate synthase/dihydrofolate synthase	GO:0000166,GO:0004326,GO:0005524,GO:0009058,GO:0009396,GO:0016874,GO:0046901	K11754	H
FPS10_03710	acetyl-CoA carboxylase carboxyltransferase subunit beta	GO:0000166,GO:0003989,GO:0005524,GO:0005737,GO:0006629,GO:0006631,GO:0006633,GO:0008270,GO:0009317,GO:0016740,GO:0016874,GO:0046872,GO:2001295	K01963	I
FPS10_03715	CPBP family intramembrane metalloprotease		K07052	R
FPS10_03720	CPBP family intramembrane metalloprotease		K07052	R
FPS10_03725	hypothetical protein			
FPS10_03730	hypothetical protein			
FPS10_03735	hypothetical protein			
FPS10_03740	FAD-dependent oxidoreductase	GO:0003824,GO:0010181,GO:0016491,GO:0055114	K00219	R
FPS10_03745	LysR family transcriptional regulator	GO:0003677,GO:0003700,GO:0006351,GO:0006355		K
FPS10_03750	OmpA family protein			M
FPS10_03755	peroxidase-related enzyme	GO:0004601,GO:0016209,GO:0016491,GO:0051920,GO:0055114,GO:0098869		S
FPS10_03760	GNAT family N-acetyltransferase	GO:0008080,GO:0008152,GO:0016740		
FPS10_03765	competence/damage-inducible protein A			R
FPS10_03770	acetoacetyl-CoA reductase	GO:0005737,GO:0008152,GO:0016491,GO:0018454,GO:0042619,GO:0055114	K00023	IQR
FPS10_03775	acetyl-CoA C-acetyltransferase	GO:0003824,GO:0003985,GO:0008152,GO:0016740,GO:0016746,GO:0016747	K00626	I
FPS10_03780	EAL domain-containing protein			T
FPS10_03785	DNA-3-methyladenine glycosylase I	GO:0003824,GO:0006281,GO:0006284,GO:0008152,GO:0008725,GO:0016787,GO:0016798	K01246	L
FPS10_03790	hypothetical protein			
FPS10_03795	DNA polymerase III subunit alpha	GO:0003677,GO:0003824,GO:0003887,GO:0005737,GO:0006260,GO:0006281,GO:0006974,GO:0008408,GO:0016740,GO:0016779,GO:0071897,GO:0090305	K14162	L

FPS10_03800	hypothetical protein			
FPS10_03805	hypothetical protein			
FPS10_03810	hypothetical protein			
FPS10_03815	hypothetical protein			R
FPS10_03820	hypothetical protein			
FPS10_03825	serine/threonine-protein phosphatase		K20074	T
FPS10_03830	FHA domain-containing protein	GO:0003674,GO:0005575,GO:0008150		T
FPS10_03835	serine/threonine protein kinase	GO:0004672,GO:0004674,GO:0005524,GO:0006468,GO:0016301,GO:0016310		RTKL
FPS10_03840	glycosyltransferase			M
FPS10_03845	hypothetical protein			R
FPS10_03850	hypothetical protein			
FPS10_03855	ABC transporter ATP-binding protein		K06147	V
FPS10_03860	dTDP-4-dehydrorhamnose 3%2C5-epimerase	GO:0008152,GO:0008830	K01790	M
FPS10_03865	dTDP-glucose 4%2C6-dehydratase		K01710	M
FPS10_03870	dTDP-4-dehydrorhamnose reductase	GO:0008831,GO:0016491,GO:0055114	K00067	M
FPS10_03875	glucose-1-phosphate thymidylyltransferase RfbA	GO:0008879,GO:0009058,GO:0016740,GO:0016779,GO:0045226,GO:0046872	K00973	M
FPS10_03880	methyltransferase domain-containing protein			H
FPS10_03885	UDP-galactopyranose mutase	GO:0008152,GO:0008767,GO:0016853	K01854	M
FPS10_03890	hypothetical protein			
FPS10_03895	hypothetical protein			
FPS10_03900	BMP family ABC transporter substrate-binding protein	GO:0005886	K07335	R
FPS10_03905	ABC transporter ATP-binding protein	GO:0000166,GO:0005524,GO:0008152,GO:0008643,GO:0015407,GO:0015749,GO:0016887	K23537	R
FPS10_03910	ABC transporter permease	GO:0005215,GO:0005886,GO:0006810,GO:0016020,GO:0016021	K23535	R
FPS10_03915	ABC transporter permease	GO:0005215,GO:0005886,GO:0006810,GO:0016020,GO:0016021	K23536	R
FPS10_03920	amidohydrolase family protein		K01485	FR
FPS10_03925	acyl-CoA dehydrogenase		K09709	S
FPS10_03930	catalase/peroxidase HPI	GO:0004096,GO:0004601,GO:0006979,GO:0016491,GO:0020037,GO:0042744,GO:0046872,GO:0055114,GO:0098869	K03782	P
FPS10_03935	LysR family transcriptional regulator	GO:0003677,GO:0003700,GO:0006351,GO:0006355	K04761	K
FPS10_03940	GntR family transcriptional regulator			K
FPS10_03945	type I methionyl aminopeptidase		K01265	J
FPS10_03950	16S rRNA (guanine(966)-N(2))-methyltransferase RsmD	GO:0008168,GO:0016740,GO:0031167,GO:0032259	K08316	L
FPS10_03955	pyridine nucleotide-disulfide oxidoreductase	GO:0005623,GO:0016491,GO:0045454,GO:0050660,GO:0055114	K00529	R
FPS10_03960	mechanosensitive ion channel	GO:0016020,GO:0016021,GO:0055085	K03442	M
FPS10_03965	4a-hydroxytetrahydrobiopterin dehydratase	GO:0006729,GO:0008124,GO:0016829	K01724	H
FPS10_03970	N-acetyltransferase		K09919	S
FPS10_03975	phosphodiesterase	GO:0006629,GO:0008081,GO:0008889		C
FPS10_03980	RidA family protein			J
FPS10_03985	HlyD family type I secretion periplasmic adaptor subunit	GO:0005623,GO:0009306,GO:0015031,GO:0016020,GO:0016021		M
FPS10_03990	type I secretion system permease/ATPase			R
FPS10_03995	VacJ family lipoprotein	GO:0016020	K04754	M
FPS10_04000	ABC transporter substrate-binding protein		K07323	Q
FPS10_04005	glycosyl transferase			M
FPS10_04010	P-II family nitrogen regulator	GO:0006351,GO:0006355,GO:0006808,GO:0030234,GO:0050790	K04751	E
FPS10_04015	ammonium transporter	GO:0006810,GO:0008519,GO:0015696,GO:0016020,GO:0016021,GO:0072488	K03320	P
FPS10_04020	rhodanese-like domain-containing protein	GO:0008152,GO:0016740		P
FPS10_04025	aspartate/tyrosine/aromatic aminotransferase	GO:0003824,GO:0006520,GO:0008483,GO:0008793,GO:0009058,GO:0016740,GO:0030170,GO:0080130	K00832	E
FPS10_04030	GNAT family N-acetyltransferase			
FPS10_04035	3-mercaptopyruvate sulfurtransferase	GO:0004792,GO:0008152,GO:0016740	K01011	P
FPS10_04040	SsrA-binding protein SmpB	GO:0003723,GO:0005737,GO:0070929	K03664	O
FPS10_04045	glutathione S-transferase family protein		K00799	O
FPS10_04050	4-hydroxy-tetrahydrodipicolinate synthase	GO:0003824,GO:0005737,GO:0008152,GO:0008652,GO:0008840,GO:0009085,GO:0009089,GO:0016829,GO:0019877	K01714	EM
FPS10_04055	lytic transglycosylase domain-containing protein		K08309	M
FPS10_04060	DMT family transporter	GO:0016020,GO:0016021		GER
FPS10_04065	tRNA (5-methylaminomethyl-2-thiouridine)(34)-methyltransferase MnmD			S
FPS10_04070	FAD-binding oxidoreductase			E
FPS10_04075	DNA polymerase Y family protein	GO:0006281	K14161	L
FPS10_04080	FkbM family methyltransferase			
FPS10_04085	hypothetical protein			S
FPS10_04090	30S ribosome-binding factor RbfA	GO:0005737,GO:0006364	K02834	J

FPS10_04095	4-hydroxy-tetrahydrodipicolinate reductase	GO:0005737,GO:0008652,GO:0008839,GO:0009085,GO:0009089,GO:0016491,GO:0016726,GO:0019877,GO:0050661,GO:0051287,GO:0055114	K00215	E
FPS10_04100	GNAT family N-acetyltransferase	GO:0008080,GO:0008152,GO:0016740		
FPS10_04105	DUF1674 domain-containing protein			S
FPS10_04110	methyltransferase domain-containing protein		K03500	J
FPS10_04115	heparinase			S
FPS10_04120	bifunctional phosphoribosylaminoimidazolecarboxamide formyltransferase/IMP cyclohydrolase	GO:0003824,GO:0003937,GO:0004643,GO:0006164,GO:0006189,GO:0008152,GO:0016740,GO:0016787	K00602	F
FPS10_04125	signal peptidase II		K03101	MU
FPS10_04130	DUF3035 domain-containing protein			
FPS10_04135	insulinase family protein		K07263	R
FPS10_04140	insulinase family protein		K07263	R
FPS10_04145	saccharopine dehydrogenase		K00290	E
FPS10_04150	saccharopine dehydrogenase	GO:0016491,GO:0055114		E
FPS10_04155	cold-shock protein	GO:0003676,GO:0003677,GO:0005737,GO:0006351,GO:00063[illegible]5	K03704	K
FPS10_04160	DUF3775 domain-containing protein			
FPS10_04165	DNA recombination protein RmuC	GO:0016020,GO:0016021	K09760	S
FPS10_04170	DNA mismatch repair endonuclease MutL	GO:0005524,GO:0006281,GO:0006298,GO:0006974,GO:00309[illegible]3	K03572	L
FPS10_04175	30S ribosomal protein S15	GO:0003723,GO:0003735,GO:0005622,GO:0005840,GO:0006412,GO:0019843,GO:0030529	K02956	J
FPS10_04180	DUF1643 domain-containing protein			S
FPS10_04185	tRNA pseudouridine(55) synthase TruB	GO:0001522,GO:0003723,GO:0006396,GO:0008033,GO:0009451,GO:0009982,GO:0016853,GO:0031119	K03177	J
FPS10_04190	DUF2794 domain-containing protein			
FPS10_04195	NLP/P60 hydrolase			M
FPS10_04200	leucyl aminopeptidase family protein	GO:0004177,GO:0005622,GO:0005737,GO:0006508,GO:00082[illegible]3,GO:0008235,GO:0016787,GO:0019538,GO:0030145,GO:0046872	K01255	E
FPS10_04205	hypothetical protein	GO:0000160,GO:0004871,GO:0005622		
FPS10_04210	carbonic anhydrase	GO:0004089,GO:0008152,GO:0008270,GO:0015976,GO:00168[illegible]9	K01673	P
FPS10_04215	aspartate-semialdehyde dehydrogenase	GO:0003942,GO:0004073,GO:0005737,GO:0008652,GO:0009085,GO:0009086,GO:0009088,GO:0009089,GO:0009097,GO:0016491,GO:0016620,GO:0019877,GO:0046983,GO:0050661,GO:0051287,GO:0055114,GO:0071266	K00133	E
FPS10_04220	IS481 family transposase	GO:0003676,GO:0003677,GO:0015074		L
FPS10_04225	glucans biosynthesis glucosyltransferase MdoH	GO:0008152,GO:0016020,GO:0016021,GO:0016740	K03669	M
FPS10_04230	glucan biosynthesis protein G		K03670	P
FPS10_04235	glycosyltransferase family 4 protein	GO:0008152,GO:0016740		M
FPS10_04240	ABC transporter permease subunit	GO:0005886,GO:0006810,GO:0016020,GO:0016021		P
FPS10_04245	ABC transporter ATP-binding protein	GO:0000166,GO:0005524,GO:0008152,GO:0016887	K02049	P
FPS10_04250	ABC transporter substrate-binding protein	GO:0009228		P
FPS10_04255	flippase-like domain-containing protein	GO:0016020,GO:0016021		S
FPS10_04260	LTA synthase family protein	GO:0003824,GO:0008152,GO:0008484,GO:0016020,GO:00160[illegible]1		
FPS10_04265	CDP-alcohol phosphatidyltransferase family protein	GO:0008654,GO:0016020,GO:0016021,GO:0016740,GO:00167[illegible]0		I
FPS10_04270	cytochrome b	GO:0016020,GO:0016021,GO:0022904	K12262	C
FPS10_04275	IS6 family transposase	GO:0003676	K07498	L
FPS10_04280	transposase			L
FPS10_04285	IS110 family transposase	GO:0003677,GO:0004803,GO:0006313		L
FPS10_04290	TRAP transporter fused permease subunit	GO:0016020,GO:0016021		R
FPS10_04295	TAXI family TRAP transporter solute-binding subunit			R
FPS10_04300	DUF3299 domain-containing protein		K09950	S
FPS10_04310	hypothetical protein			
FPS10_04315	carbohydrate porin		K07267	M
FPS10_04320	amidohydrolase family protein			Q
FPS10_04325	AraC family transcriptional regulator			K
FPS10_04330	SMP-30/gluconolactonase/LRE family protein			G
FPS10_04335	hypothetical protein			
FPS10_04340	two pore domain potassium channel family protein	GO:0005249,GO:0006813,GO:0016020,GO:0016021,GO:00718[illegible]5		
FPS10_04350	BCCT family transporter		K05020	M
FPS10_04355	hypothetical protein			
FPS10_04360	DUF302 domain-containing protein			S
FPS10_04365	DUF4345 domain-containing protein	GO:0016020,GO:0016021		
FPS10_04370	arylsulfatase		K01130	P
FPS10_04375	hypothetical protein			
FPS10_04380	hypothetical protein			
FPS10_04385	helix-turn-helix domain-containing protein			K
FPS10_04390	tyrosine-type recombinase/integrase			L

FPS10_04395	site-specific integrase			L
FPS10_04400	recombinase family protein			L
FPS10_04405	MFS transporter			
FPS10_04410	BamA/TamA family outer membrane protein			U
FPS10_04415	nuclear transport factor 2 family protein			S
FPS10_04420	autotransporter outer membrane beta-barrel domain-containing protein			
FPS10_04425	efflux RND transporter permease subunit	GO:0005215,GO:0006810,GO:0016020,GO:0016021		V
FPS10_04430	efflux RND transporter periplasmic adaptor subunit			M
FPS10_04435	sulfatase-like hydrolase/transferase	GO:0003824,GO:0008152,GO:0008484	K01130	P
FPS10_04440	hypothetical protein			
FPS10_04445	formate/nitrite transporter family protein		K21993	P
FPS10_04450	DUF3179 domain-containing protein			
FPS10_04455	hypothetical protein			
FPS10_04460	hypothetical protein			
FPS10_04465	hypothetical protein			
FPS10_04470	IS110 family transposase	GO:0003677,GO:0004803,GO:0006313		L
FPS10_04475	helix-turn-helix domain-containing protein			L
FPS10_04480	site-specific integrase			L
FPS10_04485	site-specific integrase			
FPS10_04490	recombinase family protein	GO:0000150,GO:0003677,GO:0006310		L
FPS10_04495	site-specific integrase			L
FPS10_04500	type I restriction-modification system subunit M	GO:0003676,GO:0003677,GO:0006306,GO:0008168,GO:0008170,GO:0009007,GO:0009307,GO:0032259,GO:0032775	K03427	V
FPS10_04505	AAA family ATPase			
FPS10_04510	restriction endonuclease subunit S		K01154	V
FPS10_04515	type I restriction endonuclease subunit R	GO:0003677,GO:0004519,GO:0005524,GO:0006304,GO:0009035,GO:0009307,GO:0016787,GO:0090305	K01153	V
FPS10_04520	type II toxin-antitoxin system Phd/YefM family antitoxin			D
FPS10_04525	hypothetical protein			
FPS10_04530	transcriptional regulator			
FPS10_04535	hypothetical protein			
FPS10_04540	VRR-NUC domain-containing protein			
FPS10_04545	helix-turn-helix domain-containing protein			
FPS10_04550	hypothetical protein			
FPS10_04555	hypothetical protein			
FPS10_04560	hypothetical protein			
FPS10_04570	gamma-aminobutyraldehyde dehydrogenase	GO:0008152,GO:0016491,GO:0016620,GO:0055114	K00137	C
FPS10_04575	ABC transporter permease	GO:0005886,GO:0006810,GO:0016020,GO:0016021	K02053	E
FPS10_04580	ABC transporter permease		K02054	E
FPS10_04585	ABC transporter ATP-binding protein		K02052	E
FPS10_04590	ABC transporter substrate-binding protein	GO:0015846,GO:0019808,GO:0042597	K02055	E
FPS10_04595	LysR family transcriptional regulator	GO:0003677,GO:0003700,GO:0006351,GO:0006355		K
FPS10_04600	carboxymuconolactone decarboxylase family protein	GO:0051920,GO:0055114,GO:0098869		S
FPS10_04605	GTP 3'%2C8-cyclase MoaA	GO:0000166,GO:0003824,GO:0005525,GO:0006777,GO:001682 9,GO:0019008,GO:0046872,GO:0051536,GO:0051539,GO:0061597	K03639	H
FPS10_04610	molybdopterin-dependent oxidoreductase	GO:0009055,GO:0016491,GO:0020037,GO:0030151,GO:0046872,GO:0055114	K07812	C
FPS10_04615	response regulator		K07772	TK
FPS10_04620	response regulator		K07647	T
FPS10_04625	TMAO reductase system periplasmic protein TorT		K11930	G
FPS10_04630	nucleotidyltransferase family protein			
FPS10_04635	hypothetical protein			T
FPS10_04640	PqqD family protein			
FPS10_04645	lasso peptide biosynthesis B2 protein			
FPS10_04650	hypothetical protein			
FPS10_04655	hypothetical protein		K01953	E
FPS10_04660	hypothetical protein		K14160	S
FPS10_04665	AI-2E family transporter	GO:0016020,GO:0016021		R
FPS10_04670	CDP-diacylglycerol--serine O-phosphatidyltransferase		K17103	I
FPS10_04675	phosphatidylserine decarboxylase		K01613	I
FPS10_04680	hypothetical protein		K01051	G
FPS10_04685	di-trans%2Cpoly-cis-decaprenylcistransferase	GO:0003824,GO:0008152,GO:0016740	K15532	R
FPS10_04690	glycoside hydrolase family 28 protein			M
FPS10_04695	carbohydrate ABC transporter substrate-binding protein	GO:0005215,GO:0006810	K10192	G
FPS10_04700	sn-glycerol-3-phosphate ABC transporter ATP-binding protein UgpC	GO:0000166,GO:0005215,GO:0005524,GO:0006810,GO:0008152,GO:0016820,GO:0016887,GO:0043190,GO:0055085	K10195	G

FPS10_04705	carbohydrate ABC transporter permease		K10194	G
FPS10_04710	sugar ABC transporter permease	GO:0005886,GO:0006810,GO:0016020,GO:0016021	K10193	G
FPS10_04715	alpha-galactosidase	GO:0003824,GO:0004553,GO:0005975,GO:0008152,GO:0016616,GO:0016787,GO:0016798,GO:0055114	K22933	G
FPS10_04720	helix-turn-helix domain-containing protein			F
FPS10_04725	amidohydrolase family protein			Q
FPS10_04730	xylose isomerase	GO:0000287,GO:0005737,GO:0005975,GO:0006098,GO:0009045,GO:0016853,GO:0042732,GO:0046872	K01805	G
FPS10_04735	xylulokinase	GO:0004856,GO:0005975,GO:0005997,GO:0016301,GO:0016310,GO:0016740,GO:0016773,GO:0046835	K00854	G
FPS10_04740	sugar ABC transporter ATP-binding protein		K10545	G
FPS10_04745	sugar ABC transporter permease	GO:0005215,GO:0005886,GO:0006810,GO:0016020,GO:0016021	K10544	G
FPS10_04750	D-xylose ABC transporter substrate-binding protein	GO:0008152,GO:0015407,GO:0015753,GO:0016787,GO:0048029	K10543	G
FPS10_04755	ROK family transcriptional regulator	GO:0003677		KG
FPS10_04760	CRTAC1 family protein			
FPS10_04765	cytochrome-c peroxidase		K00428	P
FPS10_04770	LacI family transcriptional regulator	GO:0003677,GO:0003700,GO:0006351,GO:0006355	K05499	K
FPS10_04775	sugar phosphate isomerase/epimerase	GO:0004519,GO:0090305		G
FPS10_04780	Gfo/Idh/MocA family oxidoreductase	GO:0008152,GO:0016491,GO:0055114		R
FPS10_04785	sugar ABC transporter ATP-binding protein	GO:0000166,GO:0005524,GO:0008152,GO:0015407,GO:0015749,GO:0016787,GO:0016887	K10441	G
FPS10_04790	ABC transporter permease		K10440	G
FPS10_04795	substrate-binding domain-containing protein		K10439	G
FPS10_04800	1-acyl-sn-glycerol-3-phosphate acyltransferase		K00655	I
FPS10_04805	Lrp/AsnC family transcriptional regulator			K
FPS10_04810	acyl-CoA dehydrogenase		K00252	I
FPS10_04815	aldo/keto reductase	GO:0016491,GO:0055114		C
FPS10_04820	Na+/H+ antiporter subunit G	GO:0005451,GO:0015672,GO:0015992,GO:0016020,GO:0016021,GO:1902600	K05564	P
FPS10_04825	K+/H+ antiporter subunit F	GO:0015075,GO:0016020,GO:0016021,GO:0034220	K05563	P
FPS10_04830	Na+/H+ antiporter subunit E	GO:0006812,GO:0008324,GO:0016020,GO:0016021,GO:00986[illegible]5	K05562	P
FPS10_04835	monovalent cation/H+ antiporter subunit D	GO:0008137,GO:0016020,GO:0016021,GO:0042773,GO:0055114	K05561	CP
FPS10_04840	Na+/H+ antiporter subunit C	GO:0016020,GO:0016021,GO:0016651,GO:0042773,GO:0055114	K05560	P
FPS10_04845	monovalent cation/H+ antiporter subunit A		K05559	CP
FPS10_04850	TAXI family TRAP transporter solute-binding subunit		K07080	R
FPS10_04855	TRAP transporter fused permease subunit	GO:0016020,GO:0016021		R
FPS10_04860	universal stress protein			T
FPS10_04865	HlyD family efflux transporter periplasmic adaptor subunit		K02005	M
FPS10_04870	FtsX-like permease family protein	GO:0016020,GO:0016021	K02004	M
FPS10_04875	ABC transporter ATP-binding protein	GO:0000166,GO:0005524,GO:0008152,GO:0016887	K02003	V
FPS10_04880	hypothetical protein			
FPS10_04885	hypothetical protein			
FPS10_04890	ATP-dependent Clp protease proteolytic subunit	GO:0004252,GO:0005737,GO:0006508,GO:0008233,GO:0008236,GO:0016301,GO:0016310,GO:0016740,GO:0016787	K01358	OU
FPS10_04895	ATP-dependent Clp protease ATP-binding subunit ClpX	GO:0000166,GO:0005524,GO:0006457,GO:0006508,GO:0008233,GO:0008270,GO:0046872,GO:0046983,GO:0051082	K03544	O
FPS10_04900	aromatic hydrocarbon degradation protein			I
FPS10_04905	RidA family protein			J
FPS10_04910	NADH:ubiquinone oxidoreductase subunit NDUFA12			C
FPS10_04915	outer membrane lipid asymmetry maintenance protein MlaD		K02067	Q
FPS10_04920	DUF2155 domain-containing protein			S
FPS10_04925	leucyl/phenylalanyl-tRNA--protein transferase		K00684	O
FPS10_04930	acetyl-CoA carboxylase biotin carboxylase subunit	GO:0003824,GO:0003989,GO:0004075,GO:0005524,GO:0008152,GO:0016874,GO:0046872	K01961	I
FPS10_04935	acetyl-CoA carboxylase biotin carboxyl carrier protein		K02160	I
FPS10_04940	NAD(P)-dependent oxidoreductase	GO:0016491,GO:0051536,GO:0055114	K17722	ER
FPS10_04945	NAD-dependent dihydropyrimidine dehydrogenase subunit PreA		K17723	F
FPS10_04950	TetR family transcriptional regulator		K09017	K
FPS10_04955	aspartate aminotransferase family protein	GO:0003824,GO:0008152,GO:0008483,GO:0016223,GO:0016740,GO:0030170	K00822	H
FPS10_04960	Zn-dependent hydrolase	GO:0008152,GO:0016787,GO:0016813	K06016	E
FPS10_04965	dihydropyrimidinase	GO:0004157,GO:0005737,GO:0008152,GO:0016787,GO:0016810	K01464	F
FPS10_04970	ABC transporter ATP-binding protein	GO:0000166,GO:0005524,GO:0008152,GO:0015411,GO:0015837,GO:0016787,GO:0016887	K02049	P
FPS10_04975	ABC transporter permease	GO:0005886,GO:0006810,GO:0016020,GO:0016021	K02050	P
FPS10_04980	ABC transporter permease	GO:0006810,GO:0016020,GO:0016021	K02050	P
FPS10_04985	ABC transporter substrate-binding protein	GO:0009228	K02051	P

FPS10_04990	hypothetical protein			
FPS10_04995	hypothetical protein			
FPS10_05000	NAD(P)-dependent oxidoreductase	GO:0004616,GO:0008442,GO:0008679,GO:0016491,GO:0051287,GO:0055114		I
FPS10_05005	MFS transporter	GO:0016020,GO:0016021,GO:0055085		G
FPS10_05010	DNA alkylation repair protein			L
FPS10_05015	glutamine--fructose-6-phosphate transaminase (isomerizing)	GO:0004360,GO:0005737,GO:0005975,GO:0006541,GO:0008483,GO:0016740,GO:0030246,GO:1901137	K00820	M
FPS10_05020	bifunctional UDP-N-acetylglucosamine diphosphorylase/glucosamine-1-phosphate N-acetyltransferase GlmU	GO:0000287,GO:0000902,GO:0003824,GO:0003977,GO:0005737,GO:0006048,GO:0008152,GO:0008360,GO:0009103,GO:0009245,GO:0009252,GO:0016740,GO:0016746,GO:0016779,GO:0019134,GO:0046872,GO:0071555	K04042	M
FPS10_05025	HAD-IA family hydrolase	GO:0008152,GO:0016787	K01091	R
FPS10_05030	aminotransferase			M
FPS10_05035	hypothetical protein			
FPS10_05040	ABC transporter ATP-binding protein		K02010	E
FPS10_05045	ABC transporter ATP-binding protein/permease		K06147	O
FPS10_05050	prolyl oligopeptidase family serine peptidase	GO:0008152,GO:0016787	K06999	R
FPS10_05055	hypothetical protein			
FPS10_05060	HNH endonuclease	GO:0003676,GO:0004519,GO:0090305		V
FPS10_05065	QacE family quaternary ammonium compound efflux SMR transporter		K03297	P
FPS10_05070	YitT family protein	GO:0016020,GO:0016021		S
FPS10_05075	class II glutamine amidotransferase	GO:0006541,GO:0016740		R
FPS10_05080	ABC transporter substrate-binding protein	GO:0043190,GO:0055085	K02035	E
FPS10_05085	LysR family transcriptional regulator		K03566	K
FPS10_05090	serine hydroxymethyltransferase	GO:0003824,GO:0004372,GO:0008168,GO:0016740,GO:00322[illegible]	K00600	E
FPS10_05095	NADP-dependent isocitrate dehydrogenase	GO:0000287,GO:0004450,GO:0006099,GO:0006102,GO:0016491,GO:0016616,GO:0046872,GO:0051287,GO:0055114	K00031	C
FPS10_05100	HlyD family secretion protein			V
FPS10_05105	DUF2955 domain-containing protein			
FPS10_05110	HD domain-containing protein		K07023	R
FPS10_05115	hypothetical protein	GO:0004455,GO:0016491,GO:0016853,GO:0055114		
FPS10_05120	class I SAM-dependent methyltransferase			QR
FPS10_05125	hypothetical protein			
FPS10_05130	alpha/beta fold hydrolase		K07018	R
FPS10_05135	Rrf2 family transcriptional regulator		K13643	K
FPS10_05140	cysteine desulfurase	GO:0003824,GO:0008152,GO:0008483,GO:0016740	K04487	E
FPS10_05145	Fe-S cluster assembly protein SufB		K09014	O
FPS10_05150	hypothetical protein			
FPS10_05155	cupin domain-containing protein			S
FPS10_05160	heavy metal-binding domain-containing protein			S
FPS10_05165	hypothetical protein			
FPS10_05170	Fe-S cluster assembly ATPase SufC	GO:0000166,GO:0005524,GO:0006810,GO:0008152,GO:00168[illegible]7	K09013	O
FPS10_05175	SufD family Fe-S cluster assembly protein	GO:0016226	K09015	O
FPS10_05180	hypothetical protein			
FPS10_05185	YIP1 family protein			
FPS10_05190	cysteine desulfurase		K11717	E
FPS10_05195	hypothetical protein			
FPS10_05200	hypothetical protein		K05190	
FPS10_05205				
FPS10_05210	hypothetical protein		K09800	S
FPS10_05215	MarC family protein	GO:0005886,GO:0016020,GO:0016021	K05595	U
FPS10_05220	efflux RND transporter periplasmic adaptor subunit		K03585	M
FPS10_05225	AraC family transcriptional regulator			K
FPS10_05230	MFS transporter			
FPS10_05235	alpha/beta hydrolase			I
FPS10_05240	hypothetical protein	GO:0016020,GO:0016021		S
FPS10_05245	tyrosine-type recombinase/integrase	GO:0003677,GO:0006310,GO:0015074		L
FPS10_05255	hypothetical protein			
FPS10_05260	helix-turn-helix transcriptional regulator			
FPS10_05265	hypothetical protein			
FPS10_05270	DUF4892 domain-containing protein			M
FPS10_05275	helix-turn-helix transcriptional regulator	GO:0003677		K
FPS10_05280	hypothetical protein			
FPS10_05285	hypothetical protein			R
FPS10_05290	hypothetical protein			

FPS10_05295	TCR/Tet family MFS transporter	GO:0005215,GO:0016020,GO:0016021,GO:0055085	K08151	G
FPS10_05300	IS5/IS1182 family transposase			
FPS10_05310	AraC family transcriptional regulator			K
FPS10_05315	DUF1254 domain-containing protein			S
FPS10_05320	DUF1254 domain-containing protein			S
FPS10_05325	DUF1254 domain-containing protein			S
FPS10_05330	helix-turn-helix transcriptional regulator			
FPS10_05335	tyrosine-type recombinase/integrase	GO:0003677,GO:0006310,GO:0015074		L
FPS10_05340	hypothetical protein	GO:0016020,GO:0016021		S
FPS10_05345	dienelactone hydrolase			Q
FPS10_05355	lysine decarboxylase	GO:0003824,GO:0005737,GO:0006520,GO:0016831	K01584	E
FPS10_05360	response regulator			GER
FPS10_05365	Hpt domain-containing protein			T
FPS10_05370	response regulator			T
FPS10_05375	ABC transporter substrate-binding protein			Q
FPS10_05380	AraC family transcriptional regulator			K
FPS10_05385	amidohydrolase family protein			Q
FPS10_05390	hypothetical protein			
FPS10_05395	DUF3299 domain-containing protein			S
FPS10_05400	carbohydrate porin		K07267	
FPS10_05405	hypothetical protein			
FPS10_05415	redoxin domain-containing protein	GO:0016209,GO:0016491,GO:0055114,GO:0098869		
FPS10_05420	rhamnulose-1-phosphate aldolase		K01629	G
FPS10_05425	L-rhamnose isomerase	GO:0005737,GO:0008740,GO:0016853,GO:0019299,GO:001930 1,GO:0030145,GO:0046872	K01813	G
FPS10_05430	carbohydrate kinase	GO:0005975,GO:0016773		G
FPS10_05435	L-rhamnose mutarotase		K03534	S
FPS10_05440	ABC transporter permease	GO:0005215,GO:0005886,GO:0006810,GO:0016020,GO:001602 1	K10561	G
FPS10_05445	ABC transporter permease	GO:0005215,GO:0005886,GO:0006810,GO:0016020,GO:001602 1	K10560	G
FPS10_05450	sugar ABC transporter ATP-binding protein	GO:0000166,GO:0005524,GO:0005886,GO:0006810,GO:000815 2,GO:0008643,GO:0015407,GO:0015591,GO:0015749,GO:00157 52,GO:0016020,GO:0016787,GO:0016887,GO:0034219,GO:0043 190,GO:0043211	K10562	G
FPS10_05455	rhamnose ABC transporter substrate-binding protein	GO:0015762,GO:0043190	K10559	G
FPS10_05460	DeoR/GlpR transcriptional regulator		K03477	KG
FPS10_05465	bifunctional rhamnulose-1-phosphate aldolase/short-chain dehydrogenase	GO:0008152,GO:0016491,GO:0055114		IQR
FPS10_05470	hypothetical protein			
FPS10_05475	sodium-dependent transporter	GO:0005328,GO:0006810,GO:0006836,GO:0015293,GO:001602 0,GO:0016021,GO:0055085	K03308	R
FPS10_05485	hypothetical protein			
FPS10_05490	sulfurtransferase			P
FPS10_05500	exopolysaccharide biosynthesis protein			R
FPS10_05510	transposase			L
FPS10_05515	hypothetical protein		K07290	M
FPS10_05520	hypothetical protein			
FPS10_05525	hypothetical protein			
FPS10_05530	DUF3604 domain-containing protein			
FPS10_05535	AraC family transcriptional regulator			K
FPS10_05540	arylsulfatase	GO:0003824,GO:0008152,GO:0008484	K01130	P
FPS10_05545	two pore domain potassium channel family protein			
FPS10_05550	AI-2E family transporter			R
FPS10_05555	HlyD family secretion protein			V
FPS10_05560	DUF2955 domain-containing protein			
FPS10_05570	hypothetical protein			TZDR
FPS10_05575	aggregation factor core			
FPS10_05580	fasciclin domain-containing protein			M
FPS10_05585	PAS domain-containing protein			T
FPS10_05590	DUF427 domain-containing protein			S
FPS10_05595	DUF4149 domain-containing protein	GO:0008168,GO:0008689,GO:0016020,GO:0016021,GO:001674 0,GO:0032259		
FPS10_05600	DUF393 domain-containing protein			S
FPS10_05605	deoxyribodipyrimidine photolyase			L
FPS10_05610	FAD-binding protein		K06955	R
FPS10_05615	deoxyribodipyrimidine photolyase	GO:0008152,GO:0016829	K01669	L
FPS10_05620	DUF2256 domain-containing protein			S
FPS10_05625	AarF/ABC1/UbiB kinase family protein			R

FPS10_05630	IS6 family transposase	GO:0003676	K07498	L
FPS10_05640	asparagine synthase (glutamine-hydrolyzing)		K01953	E
FPS10_05650	IS110 family transposase	GO:0003677,GO:0004803,GO:0006313		L
FPS10_05655	hypothetical protein			
FPS10_05660	pyridoxine 5'-phosphate synthase	GO:0003824,GO:0005737,GO:0008615,GO:0016740,GO:0016769,GO:0033856	K03474	H
FPS10_05665	Dabb family protein			
FPS10_05670	holo-ACP synthase	GO:0000287,GO:0005737,GO:0006629,GO:0006631,GO:0006633,GO:0008897,GO:0016740,GO:0046872	K00997	I
FPS10_05675	signal peptidase I	GO:0006508,GO:0008233,GO:0008236,GO:0016020,GO:0016021,GO:0016787	K03100	U
FPS10_05680	ribonuclease III		K03685	K
FPS10_05685	pilus assembly protein			U
FPS10_05690	hypothetical protein			
FPS10_05695	GTPase Era	GO:0000166,GO:0003723,GO:0003924,GO:0005525,GO:0005622,GO:0005737,GO:0005886,GO:0008152,GO:0016020,GO:0019843,GO:0042254,GO:0042274,GO:0070181	K03595	R
FPS10_05700	DUF1491 family protein			S
FPS10_05705	DNA repair protein RecO	GO:0006281,GO:0006310,GO:0006974	K03584	L
FPS10_05710	3-methyladenine DNA glycosylase			L
FPS10_05715	NUDIX domain-containing protein			F
FPS10_05720	ChaN family lipoprotein			S
FPS10_05725	bifunctional adenosylcobinamide kinase/adenosylcobinamide-phosphate guanylyltransferase	GO:0000166,GO:0005525,GO:0008820,GO:0009236,GO:0016310,GO:0016740,GO:0016779,GO:0043752,GO:0051188	K02231	H
FPS10_05730	histidine phosphatase family protein			G
FPS10_05735	glutathione S-transferase	GO:0008152,GO:0016740	K00799	O
FPS10_05740	sugar transferase	GO:0008152,GO:0016020,GO:0016021,GO:0016740		M
FPS10_05745	YifB family Mg chelatase-like AAA ATPase	GO:0003677,GO:0005524,GO:0006260	K07391	O
FPS10_05750	alpha/beta hydrolase	GO:0008152,GO:0016787	K14731	I
FPS10_05755	glutathione synthase	GO:0000166,GO:0000287,GO:0003824,GO:0004363,GO:0005524,GO:0005829,GO:0006750,GO:0016874,GO:0030145,GO:0046872	K01920	HJ
FPS10_05760	hypothetical protein	GO:0003676,GO:0004518,GO:0090305	K07460	L
FPS10_05765	16S rRNA (cytidine(1402)-2'-O)-methyltransferase	GO:0000453,GO:0005737,GO:0006364,GO:0008152,GO:0008168,GO:0016740,GO:0032259,GO:0070677	K07056	R
FPS10_05770	penicillin-binding protein activator			E
FPS10_05775	[protein-PII] uridylyltransferase		K00990	O
FPS10_05780	murein biosynthesis integral membrane protein MurJ	GO:0005886,GO:0006810,GO:0008360,GO:0009252,GO:0016020,GO:0016021,GO:0071555	K03980	R
FPS10_05785	tryptophan--tRNA ligase		K01867	J
FPS10_05790	rhomboid family intramembrane serine protease	GO:0004252,GO:0006508,GO:0016020,GO:0016021		R
FPS10_05795	OsmC family peroxiredoxin			O
FPS10_05800	branched-chain amino acid aminotransferase	GO:0003824,GO:0008152,GO:0008483,GO:0016740	K00826	EH
FPS10_05805	universal stress protein			
FPS10_05810	NifU family protein			O
FPS10_05815	tRNA (adenosine(37)-N6)-threonylcarbamoyltransferase complex dimerization subunit type 1 TsaB			O
FPS10_05820	ribosomal-protein-alanine N-acetyltransferase	GO:0008080,GO:0008152,GO:0008999,GO:0016740,GO:0016746	K03789	R
FPS10_05825	BMP family ABC transporter substrate-binding protein	GO:0005886	K07335	R
FPS10_05830	ABC transporter ATP-binding protein	GO:0000166,GO:0005524,GO:0008152,GO:0016887	K23537	R
FPS10_05835	ABC transporter permease	GO:0005215,GO:0005886,GO:0006810,GO:0016020,GO:0016021	K23535	R
FPS10_05840	ABC transporter permease	GO:0005215,GO:0005886,GO:0006810,GO:0016020,GO:0016021	K23536	R
FPS10_05845	sulfite exporter TauE/SafE family protein	GO:0016020,GO:0016021	K07090	R
FPS10_05850	sulfite exporter TauE/SafE family protein	GO:0016020,GO:0016021	K07090	R
FPS10_05855	hypothetical protein	GO:0016020,GO:0016021		
FPS10_05860	YqgE/AlgH family protein		K07735	K
FPS10_05865	hypothetical protein			OC
FPS10_05870	efflux RND transporter permease subunit	GO:0005215,GO:0006810,GO:0016020,GO:0016021		V
FPS10_05875	HlyD family efflux transporter periplasmic adaptor subunit	GO:0016020,GO:0055085		V
FPS10_05880	uracil-DNA glycosylase		K21929	L
FPS10_05885	LysE family translocator	GO:0005886,GO:0006865,GO:0016020,GO:0016021		E
FPS10_05890	molybdenum cofactor biosynthesis protein B	GO:0006777	K03638	H
FPS10_05895	tetratricopeptide repeat protein			U
FPS10_05900	tetratricopeptide repeat protein			U
FPS10_05905	hypothetical protein	GO:0004190,GO:0006508,GO:0008233,GO:0016020,GO:0016021,GO:0016787	K02278	OU
FPS10_05910	hypothetical protein			
FPS10_05915	ATPase			O
FPS10_05920	NAD(P)-dependent oxidoreductase		K00020	I

FPS10_05925	DUF389 domain-containing protein			S
FPS10_05930	ligase-associated DNA damage response DEXH box helicase	GO:0000166,GO:0003676,GO:0004386,GO:0005524,GO:0008152,GO:0016787,GO:0016818	K03724	R
FPS10_05935	ligase-associated DNA damage response endonuclease PdeM		K06953	R
FPS10_05940	PaaI family thioesterase			Q
FPS10_05945	bifunctional methylenetetrahydrofolate dehydrogenase/methenyltetrahydrofolate cyclohydrolase FolD		K01491	H
FPS10_05950	formate--tetrahydrofolate ligase		K01938	F
FPS10_05955	methyltransferase domain-containing protein		K00598	R
FPS10_05960	ATP-dependent metallopeptidase FtsH/Yme1/Tma family protein	GO:0000166,GO:0004222,GO:0005524,GO:0005886,GO:0006508,GO:0008233,GO:0008237,GO:0008270,GO:0016020,GO:0016021,GO:0016787,GO:0016887,GO:0030163,GO:0046872	K03798	O
FPS10_05965	tRNA lysidine(34) synthetase TilS		K04075	D
FPS10_05970	tol-pal system protein YbgF			S
FPS10_05975	peptidoglycan-associated lipoprotein Pal		K03640	M
FPS10_05980	hypothetical protein			
FPS10_05985	Tol-Pal system protein TolB		K03641	U
FPS10_05990	pilus assembly protein	GO:0016020,GO:0016021		
FPS10_05995	lytic transglycosylase domain-containing protein			M
FPS10_06000	SDR family NAD(P)-dependent oxidoreductase	GO:0008152,GO:0016491,GO:0055114		R
FPS10_06005	FAD-binding oxidoreductase	GO:0003824,GO:0003885,GO:0016020,GO:0016491,GO:0016614,GO:0050660,GO:0055114		C
FPS10_06010	CoA transferase subunit B	GO:0008152,GO:0008260,GO:0008410,GO:0016740	K01029	I
FPS10_06015	CoA transferase subunit A	GO:0006725,GO:0008152,GO:0008410,GO:0016740,GO:00475[illegible]9	K01028	I
FPS10_06020	PLP-dependent aminotransferase family protein	GO:0003677,GO:0003700,GO:0003824,GO:0006351,GO:0006355,GO:0009058,GO:0030170		KE
FPS10_06025	DUF1127 domain-containing protein			S
FPS10_06030	type I DNA topoisomerase	GO:0000287,GO:0003677,GO:0003916,GO:0003917,GO:0005694,GO:0006265,GO:0016853,GO:0046872	K03168	L
FPS10_06035	FadR family transcriptional regulator			K
FPS10_06040	2-hydroxy-3-oxopropionate reductase		K00042	I
FPS10_06045	DNA-protecting protein DprA	GO:0009294	K04096	LU
FPS10_06050	metalloprotease TldD	GO:0006508,GO:0008237	K03568	R
FPS10_06055	cytochrome c oxidase subunit II		K02275	C
FPS10_06060	protoheme IX farnesyltransferase	GO:0004659,GO:0005886,GO:0006783,GO:0008495,GO:0016020,GO:0016021,GO:0016740,GO:0048034	K02257	O
FPS10_06065	cytochrome c oxidase assembly protein		K02258	O
FPS10_06070	cytochrome c oxidase subunit 3		K02276	C
FPS10_06075	SURF1 family protein	GO:0016020,GO:0016021	K14998	S
FPS10_06080	threonine synthase	GO:0004795,GO:0006520,GO:0016829,GO:0030170	K01733	E
FPS10_06085	insulinase family protein	GO:0003824,GO:0004222,GO:0006508,GO:0016787,GO:00468[illegible]2		R
FPS10_06090	GNAT family N-acetyltransferase	GO:0008080,GO:0008152,GO:0016740,GO:0016746	K03790	J
FPS10_06095	MBL fold metallo-hydrolase			R
FPS10_06100	FAD-binding oxidoreductase	GO:0003824,GO:0004458,GO:0016491,GO:0016614,GO:0050660,GO:0055114		C
FPS10_06105	adenine phosphoribosyltransferase	GO:0003999,GO:0005737,GO:0006166,GO:0006168,GO:0009116,GO:0016740,GO:0016757,GO:0044209	K00759	F
FPS10_06110	flavin reductase family protein			R
FPS10_06115	N-acetyltransferase	GO:0008080,GO:0008152,GO:0016740		R
FPS10_06120	hypothetical protein			
FPS10_06125	phosphoenolpyruvate--protein phosphotransferase		K08484	T
FPS10_06130	aspartate kinase	GO:0004072,GO:0008152,GO:0008652,GO:0009089,GO:0016301,GO:0016310,GO:0016597,GO:0016740	K00928	E
FPS10_06135	Hint domain-containing protein			
FPS10_06140	thermonuclease family protein			L
FPS10_06145	ImmA/IrrE family metallo-endopeptidase	GO:0003677,GO:0006355,GO:0043565	K21686	K
FPS10_06150	multidrug effflux MFS transporter	GO:0005215,GO:0006810,GO:0016020,GO:0016021,GO:00550[illegible]5	K07552	G
FPS10_06155	acyl-CoA carboxylase subunit beta	GO:0003989,GO:0004658,GO:0006633,GO:0009317,GO:0016874,GO:0019541	K01966	I
FPS10_06160	hypothetical protein			
FPS10_06165	hypothetical protein			
FPS10_06170	hypothetical protein			
FPS10_06175	acetyl/propionyl/methylcrotonyl-CoA carboxylase subunit alpha	GO:0003824,GO:0004075,GO:0005524,GO:0008152,GO:0046872	K01965	I
FPS10_06180	DUF4174 domain-containing protein			
FPS10_06185	hypothetical protein			

FPS10_06190	methylmalonyl-CoA mutase	GO:0003824,GO:0004494,GO:0008152,GO:0016853,GO:0016866,GO:0031419,GO:0046872	K01847	I
FPS10_06195	VOC family protein			
FPS10_06200	aldo/keto reductase	GO:0016491,GO:0055114		R
FPS10_06205	molecular chaperone DjiA		K05801	O
FPS10_06210	DUF1992 domain-containing protein			
FPS10_06215	endonuclease	GO:0004519,GO:0004527,GO:0090305		R
FPS10_06220	hypothetical protein			
FPS10_06225	class I SAM-dependent methyltransferase		K00564	J
FPS10_06230	SDR family oxidoreductase	GO:0008152,GO:0016491,GO:0055114	K00076	IQR
FPS10_06235	hypothetical protein			
FPS10_06240	oxygen-dependent coproporphyrinogen oxidase	GO:0004109,GO:0006779,GO:0006782,GO:0016491,GO:0055114	K00228	H
FPS10_06245	trimeric intracellular cation channel family protein	GO:0016020,GO:0016021		S
FPS10_06250	uroporphyrinogen decarboxylase	GO:0004853,GO:0005737,GO:0006779,GO:0006782,GO:0016829,GO:0016831	K01599	H
FPS10_06255	hydroxymethylbilane synthase	GO:0004418,GO:0006779,GO:0006782,GO:0016740,GO:0018160,GO:0033014	K01749	H
FPS10_06260	Ppx/GppA family phosphatase		K01524	FP
FPS10_06265	RNA degradosome polyphosphate kinase	GO:0000166,GO:0005524,GO:0006799,GO:0008976,GO:0009358,GO:0016301,GO:0016310,GO:0016740	K00937	P
FPS10_06270	chromosomal replication initiator DnaA			L
FPS10_06275	AI-2E family transporter	GO:0016020,GO:0016021		R
FPS10_06280	proline--tRNA ligase		K01881	J
FPS10_06285	histidine phosphatase family protein			T
FPS10_06290	hypothetical protein			U
FPS10_06295	lipoprotein-releasing ABC transporter permease subunit	GO:0005886,GO:0016020,GO:0016021,GO:0042953,GO:0042954	K09808	M
FPS10_06300	ABC transporter ATP-binding protein	GO:0000166,GO:0005524,GO:0005886,GO:0006810,GO:0008152,GO:0016020,GO:0016787,GO:0016887,GO:0042953,GO:0042954,GO:0043190	K09810	V
FPS10_06305	cytochrome c			
FPS10_06310	c-type cytochrome	GO:0009055,GO:0020037		C
FPS10_06315	MipA/OmpV family protein			M
FPS10_06320	EAL domain-containing protein			T
FPS10_06325	glycosyltransferase family 2 protein	GO:0008152,GO:0016020,GO:0016021,GO:0016740		M
FPS10_06330	hypothetical protein			
FPS10_06335	flippase-like domain-containing protein		K07027	
FPS10_06340	hypothetical protein			
FPS10_06345	dienelactone hydrolase family protein	GO:0008152,GO:0016787	K01061	Q
FPS10_06350	AhpC/TSA family protein	GO:0016209,GO:0016491,GO:0055114,GO:0098869		O
FPS10_06355	AraC family transcriptional regulator			K
FPS10_06360	CapA family protein			M
FPS10_06365	succinyl-diaminopimelate desuccinylase	GO:0008152,GO:0008270,GO:0008652,GO:0009014,GO:0009085,GO:0009089,GO:0016787,GO:0019877,GO:0046872,GO:0050897	K01439	E
FPS10_06370	ABC transporter ATP-binding protein	GO:0000166,GO:0005524,GO:0006810,GO:0008152,GO:0015833,GO:0016887	K02031	R
FPS10_06375	ABC transporter permease	GO:0005886,GO:0006810,GO:0016020,GO:0016021	K02034	EP
FPS10_06380	ABC transporter permease	GO:0005886,GO:0006810,GO:0016020,GO:0016021	K02033	EP
FPS10_06385	ABC transporter substrate-binding protein	GO:0043190,GO:0055085	K02035	E
FPS10_06390	LysR family transcriptional regulator			K
FPS10_06395	hypothetical protein			
FPS10_06400	cytochrome c	GO:0009055,GO:0020037		C
FPS10_06405	serine O-acetyltransferase		K00640	E
FPS10_06410	DUF2793 domain-containing protein			
FPS10_06415	host specificity protein			
FPS10_06420	peptidase			M
FPS10_06425	DUF2163 domain-containing protein			S
FPS10_06430	TIGR02217 family protein			S
FPS10_06435	phage tail tape measure protein			S
FPS10_06440	phage tail assembly chaperone			
FPS10_06445	gene transfer agent family protein			
FPS10_06450	phage major tail protein%2C TP901-1 family			S
FPS10_06455	DUF3168 domain-containing protein			
FPS10_06460	head-tail adaptor protein			R
FPS10_06465	hypothetical protein			
FPS10_06470	phage major capsid protein			R
FPS10_06475	HK97 family phage prohead protease	GO:0006508,GO:0008233	K06904	R
FPS10_06480	phage portal protein			S

FPS10_06485	ATP-binding protein			S
FPS10_06490	hypothetical protein			
FPS10_06495	endolytic transglycosylase MltG	GO:0003676	K07082	R
FPS10_06500	beta-ketoacyl-ACP synthase II	GO:0003824,GO:0006633,GO:0008152,GO:0016740,GO:0016746,GO:0016747,GO:0033817	K09458	IQ
FPS10_06505	carbon starvation protein A	GO:0009267,GO:0016020,GO:0016021		T
FPS10_06510	DNA gyrase subunit A	GO:0000166,GO:0003677,GO:0003916,GO:0003918,GO:0005524,GO:0005694,GO:0005737,GO:0006259,GO:0006261,GO:0006265,GO:0016853	K02469	L
FPS10_06515	hypothetical protein			
FPS10_06520	methylenetetrahydrofolate--tRNA-(uracil(54)- C(5))-methyltransferase (FADH(2)-oxidizing) TrmFO		K04094	J
FPS10_06525	hypothetical protein			
FPS10_06530	tRNA glutamyl-Q(34) synthetase GluQRS	GO:0000166,GO:0004812,GO:0005524,GO:0005737,GO:0006412,GO:0006418,GO:0016874,GO:0016876,GO:0043039	K01894	J
FPS10_06535	nucleoside-diphosphate kinase	GO:0000166,GO:0004550,GO:0005524,GO:0005737,GO:0006165,GO:0006183,GO:0006228,GO:0006241,GO:0009117,GO:0016301,GO:0016310,GO:0016740,GO:0046872	K00940	F
FPS10_06540	hypothetical protein			
FPS10_06545	ABC-F family ATP-binding cassette domain-containing protein	GO:0000166,GO:0005524,GO:0008152,GO:0016887	K06158	R
FPS10_06550	hypothetical protein			
FPS10_06555	NAAT family transporter	GO:0005886,GO:0016020,GO:0016021	K05595	U
FPS10_06560	TIGR02281 family clan AA aspartic protease		K06985	R
FPS10_06565	O-acetylhomoserine aminocarboxypropyltransferase/cysteine synthase	GO:0003824,GO:0003961,GO:0006520,GO:0016740,GO:0016765,GO:0030170,GO:0071266	K01740	E
FPS10_06570	DUF541 domain-containing protein		K09807	S
FPS10_06575	hypothetical protein			
FPS10_06580	potassium transporter	GO:0006810,GO:0006811,GO:0006812,GO:0006813,GO:0008324,GO:0015297,GO:0015299,GO:0016020,GO:0016021,GO:0055085,GO:0098655,GO:1902600	K03455	P
FPS10_06585	nicotinate-nucleotide--dimethylbenzimidazole phosphoribosyltransferase	GO:0008939,GO:0009163,GO:0009236,GO:0015420,GO:0015889,GO:0016740,GO:0016757,GO:0035461	K00768	H
FPS10_06590	adenosylcobinamide-GDP ribazoletransferase	GO:0005886,GO:0008818,GO:0009236,GO:0016020,GO:0016021,GO:0016740,GO:0051073	K02233	H
FPS10_06595	inorganic phosphate transporter	GO:0005315,GO:0006817,GO:0016020,GO:0016021,GO:005508 5	K03306	P
FPS10_06600	CarD family transcriptional regulator		K07736	K
FPS10_06605	ferredoxin family protein	GO:0009055,GO:0046872,GO:0051536,GO:0051539	K05524	C
FPS10_06610	RNA-binding S4 domain-containing protein	GO:0003723,GO:0004812,GO:0008152	K04762	J
FPS10_06615	disulfide oxidoreductase		K17675	LKJ
FPS10_06620	tetratricopeptide repeat protein			NU
FPS10_06625	SCP2 sterol-binding domain-containing protein			I
FPS10_06630	alpha/beta hydrolase	GO:0008152,GO:0016787	K01048	I
FPS10_06635	3%2C4-dihydroxy-2-butanone-4-phosphate synthase		K14652	H
FPS10_06640	6%2C7-dimethyl-8-ribityllumazine synthase	GO:0000906,GO:0009231,GO:0009349,GO:0016740	K00794	H
FPS10_06645	transcription antitermination factor NusB	GO:0003723,GO:0006351,GO:0006353,GO:0006355	K03625	K
FPS10_06650	hypothetical protein			
FPS10_06655	2-(1%2C2-epoxy-1%2C2-dihydrophenyl)acetyl-CoA isomerase	GO:0003824,GO:0008152,GO:0016853	K15866	I
FPS10_06660	cytochrome c-type biogenesis protein CcmH	GO:0016020,GO:0016021	K02200	O
FPS10_06665	heme lyase CcmF/NrfE family subunit	GO:0015232,GO:0015886,GO:0016020,GO:0016021,GO:0017004,GO:0020037	K02198	O
FPS10_06670	hypothetical protein			
FPS10_06675	hypothetical protein			
FPS10_06680	D-galactarate dehydratase			
FPS10_06685	GTP 3'%2C8-cyclase MoaA	GO:0000166,GO:0003824,GO:0005525,GO:0006777,GO:0016829,GO:0019008,GO:0046872,GO:0051536,GO:0051539,GO:0061597	K03639	H
FPS10_06690	rhodanese-like domain-containing protein			P
FPS10_06695	glycosyl transferase			
FPS10_06700	hypothetical protein	GO:0003677,GO:0006351,GO:0006355		TK
FPS10_06705	DNA-3-methyladenine glycosylase 2 family protein	GO:0003824,GO:0006281,GO:0006284	K01247	L
FPS10_06710	MFS transporter	GO:0008152,GO:0016020,GO:0016021,GO:0016740,GO:005508 5		G
FPS10_06715	HlyD family secretion protein	GO:0016020,GO:0016021		V
FPS10_06725	NAD(P)(+) transhydrogenase (Re/Si-specific) subunit beta	GO:0005886,GO:0008750,GO:0016020,GO:0016021,GO:0016491,GO:0050661,GO:0055114	K00325	C
FPS10_06730	Re/Si-specific NAD(P)(+) transhydrogenase subunit alpha	GO:0000166,GO:0008746,GO:0008750,GO:0015992,GO:0016020,GO:0016021,GO:0016491,GO:0055114	K00324	C
FPS10_06735	isoprenylcysteine carboxylmethyltransferase family protein			O
FPS10_06740	MBL fold metallo-hydrolase			R

FPS10_06745	kynureninase	GO:0003824,GO:0005737,GO:0006569,GO:0009435,GO:0016787,GO:0019363,GO:0019805,GO:0030170,GO:0030429,GO:0034354,GO:0043420,GO:0097053	K01556	E
FPS10_06750	GNAT family N-acetyltransferase		K03830	
FPS10_06755	gamma-glutamyl-gamma-aminobutyrate hydrolase family protein	GO:0003674,GO:0005575,GO:0006541,GO:0008150,GO:0016787	K07010	R
FPS10_06760	heme-binding protein			R
FPS10_06765	malate synthase G	GO:0003824,GO:0004474,GO:0005737,GO:0006097,GO:0006099,GO:0016740,GO:0046872	K01638	C
FPS10_06770	chemotaxis protein CheD	GO:0006935,GO:0008152,GO:0016787,GO:0050568	K03411	NT
FPS10_06775	chemotaxis response regulator protein-glutamate methylesterase	GO:0000156,GO:0000160,GO:0004871,GO:0005737,GO:0006935,GO:0007165,GO:0008152,GO:0008984,GO:0016787	K03412	NT
FPS10_06780	cyclic nucleotide-binding domain-containing protein			T
FPS10_06785	hydrogenase maturation protease		K03605	C
FPS10_06790	alpha/beta hydrolase			R
FPS10_06795	DUF2478 domain-containing protein			
FPS10_06800	ANTAR domain-containing protein	GO:0000160,GO:0003723,GO:0005622	K07183	T
FPS10_06805	ABC transporter substrate-binding protein		K22067	P
FPS10_06810	ABC transporter substrate-binding protein		K15576	P
FPS10_06815	ABC transporter permease		K15577	P
FPS10_06820	ABC transporter ATP-binding protein		K15578	P
FPS10_06825	GAF domain-containing protein			
FPS10_06830	hemin receptor	GO:0005344,GO:0006810,GO:0015671,GO:0019825,GO:0020037		C
FPS10_06835	NAD(P)/FAD-dependent oxidoreductase	GO:0008942,GO:0016491,GO:0020037,GO:0042128,GO:0046872,GO:0050660,GO:0050661,GO:0051536,GO:0051539,GO:0055114	K00362	C
FPS10_06840	nitrite reductase small subunit NirD	GO:0008942,GO:0016491,GO:0042128,GO:0051537,GO:0055114	K00363	PR
FPS10_06845	molybdopterin-dependent oxidoreductase	GO:0009703,GO:0016491,GO:0030151,GO:0046872,GO:0051536,GO:0051539,GO:0055114	K00372	C
FPS10_06850	glycosyl transferase family protein			E
FPS10_06855	siroheme synthase		K02302	H
FPS10_06860	flagellar motor switch protein FliG		K02410	N
FPS10_06865	lysophospholipid acyltransferase family protein		K02517	M
FPS10_06870	DUF1223 domain-containing protein			S
FPS10_06875	hypothetical protein			
FPS10_06880	bifunctional aconitate hydratase 2/2-methylisocitrate dehydratase	GO:0003994,GO:0006099,GO:0008152,GO:0051539	K01682	C
FPS10_06885	aconitate hydratase AcnA	GO:0003994,GO:0008152,GO:0016829,GO:0051536,GO:0051539	K01681	C
FPS10_06890	hypothetical protein			
FPS10_06895	hypothetical protein			
FPS10_06900	cytochrome c biogenesis protein CcdA	GO:0016020,GO:0016021,GO:0017004,GO:0055114	K06196	O
FPS10_06905	sulfurtransferase TusA family protein		K04085	O
FPS10_06910	fumarylacetoacetate hydrolase family protein	GO:0003824,GO:0008152,GO:0016853		Q
FPS10_06915	pyridoxal phosphate-dependent aminotransferase			E
FPS10_06920	phospholipase D family protein	GO:0003824,GO:0008152	K06132	I
FPS10_06925	HAMP domain-containing protein	GO:0000155,GO:0000160,GO:0000166,GO:0004871,GO:0005524,GO:0005622,GO:0007165,GO:0016020,GO:0016021,GO:0016301,GO:0016310,GO:0016740,GO:0016772,GO:0023014	K07638	T
FPS10_06930	MBL fold metallo-hydrolase	GO:0004416,GO:0008152,GO:0016787		R
FPS10_06935				
FPS10_06940	tyrosine-type recombinase/integrase			
FPS10_06945	hypothetical protein			
FPS10_06950	recombinase family protein	GO:0000150,GO:0003677,GO:0006310		L
FPS10_06955	DUF3604 domain-containing protein			
FPS10_06960	AraC family transcriptional regulator			K
FPS10_06965	DUF3604 domain-containing protein	GO:0003824,GO:0008152		
FPS10_06970	DUF3604 domain-containing protein			
FPS10_06975	hypothetical protein			C
FPS10_06980	Crp/Fnr family transcriptional regulator		K10914	T
FPS10_06985	extracellular solute-binding protein		K11069	E
FPS10_06990	ABC transporter ATP-binding protein	GO:0000166,GO:0005215,GO:0005524,GO:0006810,GO:0008152,GO:0016820,GO:0016887,GO:0043190,GO:0055085	K11072	E
FPS10_06995	ABC transporter permease		K11071	E
FPS10_07000	ABC transporter permease	GO:0005886,GO:0006810,GO:0016020,GO:0016021	K11070	E
FPS10_07005	MurR/RpiR family transcriptional regulator	GO:0003677,GO:0003700,GO:0005975,GO:0006355,GO:0030246		K
FPS10_07010	N-formylglutamate amidohydrolase			E
FPS10_07015	TRAP transporter small permease subunit	GO:0016020,GO:0016021		Q
FPS10_07020	TRAP transporter large permease subunit	GO:0016020,GO:0016021		Q
FPS10_07025	twin-arginine translocation signal domain-containing protein	GO:0006810,GO:0030288		Q

FPS10_07030	glutamine synthetase	GO:0003824,GO:0004356,GO:0006542,GO:0006807	K01915	E
FPS10_07035	DUF4440 domain-containing protein			S
FPS10_07040	aldehyde dehydrogenase family protein			C
FPS10_07045	iron-containing alcohol dehydrogenase			C
FPS10_07050	MFS transporter	GO:0016020,GO:0016021,GO:0055085		G
FPS10_07055	nuclear transport factor 2 family protein			R
FPS10_07060	GAF domain-containing protein	GO:0003677,GO:0006351,GO:0006355		TK
FPS10_07065	tripartite tricarboxylate transporter protein TctA		K07793	S
FPS10_07070	tripartite tricarboxylate transporter TctB family protein			
FPS10_07075	tripartite tricarboxylate transporter substrate binding protein			S
FPS10_07080	sulfatase			P
FPS10_07085	LysR family transcriptional regulator			K
FPS10_07095	hypothetical protein			
FPS10_07100	hypothetical protein			NU
FPS10_07105	phosphatase PAP2 family protein	GO:0004601,GO:0055114,GO:0098869		I
FPS10_07110	hypothetical protein			
FPS10_07115	hypothetical protein			
FPS10_07120	hypothetical protein			
FPS10_07125	hypothetical protein			M
FPS10_07140	hypothetical protein			
FPS10_07145	MotA/TolQ/ExbB proton channel family protein	GO:0006810,GO:0008565,GO:0015031,GO:0016020,GO:0016021	K03561	U
FPS10_07150	biopolymer transporter ExbD	GO:0005215,GO:0005886,GO:0006810,GO:0015031,GO:0016020,GO:0016021	K03559	U
FPS10_07155	biopolymer transporter ExbD		K03559	U
FPS10_07160	DUF4384 domain-containing protein			
FPS10_07165	PepSY domain-containing protein			S
FPS10_07170	3-carboxy-cis%2Ccis-muconate cycloisomerase	GO:0003824,GO:0008152,GO:0016853,GO:0047472	K01857	F
FPS10_07175	3-keto-5-aminohexanoate cleavage protein	GO:0003824,GO:0016740,GO:0019475		S
FPS10_07180	protocatechuate 3%2C4-dioxygenase subunit alpha	GO:0003824,GO:0005506,GO:0006725,GO:0008199,GO:0016491,GO:0016702,GO:0018578,GO:0019439,GO:0051213,GO:0055114	K00448	Q
FPS10_07185	protocatechuate 3%2C4-dioxygenase subunit beta	GO:0003824,GO:0005506,GO:0006725,GO:0008199,GO:0016702,GO:0018578,GO:0019619,GO:0051213,GO:0055114	K00449	Q
FPS10_07190	4-carboxymuconolactone decarboxylase	GO:0016829,GO:0047575,GO:0051920,GO:0055114,GO:0098869	K01607	S
FPS10_07195	3-oxoadipate enol-lactonase	GO:0016787,GO:0042952,GO:0047570	K01055	R
FPS10_07200	4-hydroxybenzoate 3-monooxygenase	GO:0004497,GO:0016491,GO:0018659,GO:0043639,GO:0050660,GO:0055114,GO:0071949	K00481	HC
FPS10_07205	pca operon transcription factor PcaQ	GO:0003677,GO:0003700,GO:0006351,GO:0006355,GO:0019619,GO:0045893	K02623	K
FPS10_07210	acyl-CoA dehydrogenase	GO:0003995,GO:0008152,GO:0016491,GO:0016627,GO:0050660,GO:0055114		I
FPS10_07215	amidohydrolase	GO:0008152,GO:0016787	K07045	R
FPS10_07220	feruloyl-CoA synthase	GO:0003824,GO:0008152	K12508	IQ
FPS10_07225	MarR family transcriptional regulator	GO:0003677,GO:0003700,GO:0006351,GO:0006355		K
FPS10_07230	crotonase/enoyl-CoA hydratase family protein	GO:0003824,GO:0008152,GO:0016853	K20036	I
FPS10_07235	SDR family NAD(P)-dependent oxidoreductase	GO:0008152,GO:0016491,GO:0055114		IQR
FPS10_07240	TRAP transporter large permease	GO:0016021		G
FPS10_07245	TRAP transporter small permease			
FPS10_07250	TRAP transporter substrate-binding protein	GO:0006810,GO:0030288		G
FPS10_07255	DoxX family protein	GO:0016020,GO:0016021	K15977	S
FPS10_07260	4%2C5-DOPA dioxygenase extradiol		K15777	S
FPS10_07265	ribulokinase	GO:0000166,GO:0005524,GO:0005975,GO:0008741,GO:0016301,GO:0016310,GO:0016740,GO:0016773,GO:0019568,GO:0019569,GO:0046835	K00853	C
FPS10_07270	arabinose operon transcriptional regulator AraC		K02099	K
FPS10_07275	SMP-30/gluconolactonase/LRE family protein		K01053	G
FPS10_07280	L-arabinose isomerase		K01804	G
FPS10_07285	L-ribulose-5-phosphate 4-epimerase	GO:0008270,GO:0008742,GO:0016853,GO:0019572	K03077	G
FPS10_07290	sugar ABC transporter permease		K10547	G
FPS10_07295	sugar ABC transporter ATP-binding protein	GO:0000166,GO:0005524,GO:0008152,GO:0016887	K10548	G
FPS10_07300	sugar ABC transporter substrate-binding protein		K10546	G
FPS10_07310	galactarate dehydratase		K01708	G
FPS10_07315	tripartite tricarboxylate transporter permease		K07793	S
FPS10_07320	tripartite tricarboxylate transporter	GO:0016020,GO:0016021		
FPS10_07325	ABC transporter substrate-binding protein			S
FPS10_07330	FadR family transcriptional regulator	GO:0003677,GO:0003700,GO:0006351,GO:0006355		K
FPS10_07335	glucarate dehydratase	GO:0000287,GO:0003824,GO:0008152,GO:0008872,GO:0016829,GO:0019394,GO:0046872	K01706	MR
FPS10_07340	2%2C4-dihydroxyhept-2-ene-1%2C7-dioic acid aldolase	GO:0003824,GO:0008152,GO:0016829,GO:0046872	K02510	G

FPS10_07345	2-hydroxy-3-oxopropionate reductase	GO:0004616,GO:0008679,GO:0016491,GO:0046487,GO:0051287,GO:0055114	K00042	I
FPS10_07350	glycerate kinase		K11529	G
FPS10_07355	HAD-IA family hydrolase			R
FPS10_07360	FGGY-family carbohydrate kinase	GO:0005975,GO:0008741,GO:0016301,GO:0016310,GO:0016740,GO:0016773,GO:0046835	K00875	C
FPS10_07365	SDR family oxidoreductase	GO:0008152,GO:0016491,GO:0055114	K00039	R
FPS10_07370	SDR family oxidoreductase	GO:0008152,GO:0016491,GO:0050038,GO:0055114		IQR
FPS10_07375	ribose ABC transporter permease		K10440	G
FPS10_07380	sugar ABC transporter ATP-binding protein	GO:0000166,GO:0005524,GO:0008152,GO:0016887	K10441	G
FPS10_07385	substrate-binding domain-containing protein		K10439	G
FPS10_07390	AraC family transcriptional regulator	GO:0003677,GO:0003700,GO:0006351,GO:0006355,GO:00435[illegible]5		G
FPS10_07395	xylulokinase	GO:0004856,GO:0005975,GO:0005997,GO:0016301,GO:0016310,GO:0016740,GO:0016773,GO:0046835	K00854	G
FPS10_07400	NAD(P)-dependent alcohol dehydrogenase	GO:0008270,GO:0016491,GO:0046526,GO:0046872,GO:00551[illegible]4	K05351	ER
FPS10_07405	cyclic nucleotide-binding domain-containing protein	GO:0006810,GO:0006812,GO:0015299,GO:0016020,GO:0016021,GO:0055085,GO:1902600	K03316	P
FPS10_07410	winged helix DNA-binding protein			K
FPS10_07415	TRAP transporter large permease subunit	GO:0016020,GO:0016021		G
FPS10_07420	TRAP transporter small permease	GO:0016020,GO:0016021		G
FPS10_07425	TRAP transporter substrate-binding protein			G
FPS10_07430	allophanate hydrolase subunit 1	GO:0008152,GO:0016787		E
FPS10_07435	urea amidolyase			E
FPS10_07440	5-oxoprolinase subunit PxpA	GO:0003824,GO:0005975	K07160	R
FPS10_07445	histidinol dehydrogenase	GO:0000105,GO:0004399,GO:0008152,GO:0008270,GO:0008652,GO:0016491,GO:0046872,GO:0051287,GO:0055114	K15509	E
FPS10_07450	phosphoenolpyruvate carboxylase		K01595	C
FPS10_07455	sulfurtransferase			P
FPS10_07460	polyribonucleotide nucleotidyltransferase		K00962	J
FPS10_07465	antibiotic biosynthesis monooxygenase			S
FPS10_07470	ferrous iron transport protein A	GO:0046914	K04758	P
FPS10_07475	ferrous iron transport protein A		K04758	P
FPS10_07480	Fe(2+) transporter permease subunit FeoB	GO:0005525,GO:0005886,GO:0006810,GO:0015093,GO:0015684,GO:0016020,GO:0016021,GO:0055072,GO:1903874	K04759	P
FPS10_07485	hypothetical protein			K
FPS10_07490	DUF839 domain-containing protein		K07093	R
FPS10_07495	hypothetical protein			
FPS10_07500	cytochrome c			C
FPS10_07505	TRAP transporter small permease subunit			Q
FPS10_07510	TRAP transporter large permease subunit			Q
FPS10_07515	TRAP transporter substrate-binding protein	GO:0006810,GO:0030288,GO:0042597,GO:0046872		Q
FPS10_07520	TRAP transporter substrate-binding protein	GO:0006810,GO:0030288,GO:0042597,GO:0046872		Q
FPS10_07525	NAD-dependent epimerase/dehydratase family protein	GO:0005975,GO:0016857	K08679	MG
FPS10_07530	hypothetical protein	GO:0016020,GO:0016021		
FPS10_07535				
FPS10_07540	guanylate cyclase	GO:0020037		
FPS10_07545	response regulator	GO:0000155,GO:0000160,GO:0004673,GO:0004871,GO:0005622,GO:0007165,GO:0016301,GO:0016310,GO:0016740,GO:0016772,GO:0018106,GO:0023014		T
FPS10_07550	calcium-binding protein			Q
FPS10_07555	sugar transferase	GO:0009058,GO:0016020,GO:0016021,GO:0016740,GO:00167[illegible]7		M
FPS10_07560	response regulator transcription factor	GO:0000160,GO:0003677,GO:0005622,GO:0006351,GO:00063[illegible]5		TK
FPS10_07565	sugar transporter		K01991	M
FPS10_07570	polysaccharide pyruvyl transferase family protein			S
FPS10_07575	oligosaccharide flippase family protein			R
FPS10_07580	coenzyme F420 hydrogenase		K00441	C
FPS10_07585	hypothetical protein			
FPS10_07590	glycosyltransferase family 2 protein			R
FPS10_07595	glycosyltransferase family 4 protein	GO:0008152,GO:0016740		M
FPS10_07600	UDP-N-acetylglucosamine--LPS N-acetylglucosamine transferase			M
FPS10_07605	glycosyl transferase family 28			S
FPS10_07610	glycosyltransferase	GO:0008152,GO:0016740		M
FPS10_07615	hypothetical protein			M
FPS10_07620	hypothetical protein			
FPS10_07625	aspartate/glutamate racemase family protein	GO:0006520,GO:0006807,GO:0008152,GO:0016853,GO:0016855,GO:0036361,GO:0047689	K01779	M
FPS10_07630	serine hydrolase			V

FPS10_07635	cupin domain-containing protein		K06995	R
FPS10_07640	helix-turn-helix transcriptional regulator			K
FPS10_07645	hypothetical protein			
FPS10_07650	IS1182 family transposase			L
FPS10_07655	IS3 family transposase			L
FPS10_07660	dihydroxyacetone kinase subunit DhaK	GO:0004371,GO:0006071,GO:0016310	K05878	G
FPS10_07665	winged helix-turn-helix transcriptional regulator			K
FPS10_07670	dihydroxyacetone kinase subunit DhaK		K05878	G
FPS10_07675	pentose kinase		K00854	G
FPS10_07680	deoxyribose-phosphate aldolase		K01619	F
FPS10_07685	hypothetical protein			
FPS10_07690	TetR family transcriptional regulator			K
FPS10_07695	hypothetical protein			K
FPS10_07700	hypothetical protein			
FPS10_07705	alpha/beta hydrolase			
FPS10_07715	hypothetical protein			
FPS10_07720	hypothetical protein			
FPS10_07725	hypothetical protein			
FPS10_07730	hypothetical protein			
FPS10_07735	hypothetical protein			
FPS10_07740	hypothetical protein			
FPS10_07745	AAA family ATPase			O
FPS10_07750	DUF2384 domain-containing protein	GO:0003677		R
FPS10_07755	RES domain-containing protein			
FPS10_07760	hypothetical protein			
FPS10_07765	site-specific DNA-methyltransferase	GO:0003677,GO:0006306,GO:0008168,GO:0008170,GO:00322[illegible]9		L
FPS10_07780	transposase		K07483	L
FPS10_07790	elongation factor Tu	GO:0000166,GO:0003746,GO:0003924,GO:0005525,GO:0005622,GO:0005737,GO:0006412,GO:0006414	K02358	J
FPS10_07795	transposase	GO:0003677,GO:0004803,GO:0006313,GO:0043565	K07483	L
FPS10_07800	IS66 family insertion sequence element accessory protein TnpB		K07484	L
FPS10_07805	IS66 family transposase			L
FPS10_07810	GTP-binding protein			R
FPS10_07815	amidohydrolase family protein			Q
FPS10_07820	DUF1269 domain-containing protein			S
FPS10_07825	cardiolipin synthase	GO:0003824,GO:0005886,GO:0006629,GO:0008654,GO:00088[illegible]8,GO:0016020,GO:0016021,GO:0016740,GO:0016780,GO:0032049	K06131	I
FPS10_07830	DUF3237 domain-containing protein	GO:0016021		
FPS10_07835	TRAP transporter substrate-binding protein	GO:0006810,GO:0030288		G
FPS10_07840	cytochrome P450	GO:0004497,GO:0005506,GO:0016491,GO:0016705,GO:0020037,GO:0046872,GO:0055114	K21034	Q
FPS10_07845	LysR family transcriptional regulator			K
FPS10_07850	flavin-dependent oxidoreductase	GO:0004497,GO:0016491,GO:0055114,GO:0071949	K20940	HC
FPS10_07860	AraC family transcriptional regulator			K
FPS10_07865	SDR family oxidoreductase	GO:0008152,GO:0016491,GO:0055114		IQR
FPS10_07870	hypothetical protein	GO:0016020,GO:0016021		
FPS10_07875	TetR/AcrR family transcriptional regulator			
FPS10_07880	hypothetical protein			
FPS10_07885	FAD-binding protein	GO:0016491,GO:0055114		C
FPS10_07890	YaeQ family protein			S
FPS10_07895	aspartate aminotransferase family protein	GO:0003824,GO:0004837,GO:0006520,GO:0016829,GO:0016831,GO:0019752,GO:0030170	K01593	E
FPS10_07900	hypothetical protein		K09988	R
FPS10_07905	ABC transporter permease		K10440	G
FPS10_07910	sugar ABC transporter ATP-binding protein		K10441	G
FPS10_07915	ABC transporter permease		K10440	G
FPS10_07920	sugar ABC transporter substrate-binding protein		K10439	G
FPS10_07925	3-hydroxy-5-phosphonooxypentane-2%2C4-dione thiolase		K08321	G
FPS10_07930	hypothetical protein			
FPS10_07935	hypothetical protein			
FPS10_07940	hypothetical protein			
FPS10_07945	methionine--tRNA ligase	GO:0000166,GO:0004812,GO:0004825,GO:0005524,GO:00057[illegible]7,GO:0006412,GO:0006418,GO:0006431,GO:0016874,GO:0046872	K01874	J
FPS10_07950	ABC transporter ATP-binding protein	GO:0000166,GO:0005524,GO:0008152,GO:0016887	K02003	V
FPS10_07955	ABC transporter permease	GO:0016020,GO:0016021	K02004	V
FPS10_07960	iron ABC transporter permease	GO:0008152,GO:0016787		R

FPS10_07965	valine--tRNA ligase	GO:0000166,GO:0002161,GO:0004812,GO:0004832,GO:0005524,GO:0005737,GO:0006412,GO:0006418,GO:0006438,GO:0006450,GO:0016874	K01873	J
FPS10_07970	DUF3300 domain-containing protein			
FPS10_07975	DUF2950 family protein			
FPS10_07980	DUF1992 domain-containing protein			
FPS10_07985	multidrug effflux MFS transporter		K07552	G
FPS10_07990	PilZ domain-containing protein			
FPS10_07995	threonine--tRNA ligase	GO:0000166,GO:0004812,GO:0004829,GO:0005524,GO:0005737,GO:0006412,GO:0006418,GO:0006435,GO:0016874,GO:0016876,GO:0043039,GO:0046872	K01868	J
FPS10_08000	DUF2282 domain-containing protein			S
FPS10_08005	DUF2282 domain-containing protein			S
FPS10_08010	DUF692 domain-containing protein		K09930	S
FPS10_08015	DUF2063 domain-containing protein	GO:0003674,GO:0005575,GO:0008150		S
FPS10_08020	DoxX family protein	GO:0016020,GO:0016021	K15977	S
FPS10_08025	cold-shock protein	GO:0003676,GO:0003677,GO:0005737,GO:0006351,GO:00063[illegible]5	K03704	K
FPS10_08030	hypothetical protein			
FPS10_08035	arsenate reductase			P
FPS10_08040	TraR/DksA family transcriptional regulator	GO:0008270	K06204	T
FPS10_08045	BolA family transcriptional regulator			T
FPS10_08050	Grx4 family monothiol glutaredoxin	GO:0005623,GO:0009055,GO:0015035,GO:0045454,GO:0046872,GO:0051536,GO:0051537,GO:0055114	K07390	O
FPS10_08055	cell division protein ZapA		K09888	S
FPS10_08060	hypothetical protein			D
FPS10_08065	transketolase	GO:0003824,GO:0004802,GO:0008152,GO:0016740,GO:00468[illegible]2	K00615	G
FPS10_08070	hypothetical protein			
FPS10_08075	DNA polymerase III subunit chi	GO:0003677,GO:0003887,GO:0006260,GO:0071897	K02339	L
FPS10_08080	leucyl aminopeptidase	GO:0004177,GO:0005622,GO:0005737,GO:0006508,GO:00082[illegible]3,GO:0008235,GO:0016787,GO:0019538,GO:0030145,GO:0046872	K01255	E
FPS10_08085	DUF2270 domain-containing protein			S
FPS10_08090	hypothetical protein			
FPS10_08095	exopolysaccharide biosynthesis protein			R
FPS10_08100	DEAD/DEAH box helicase	GO:0000166,GO:0003676,GO:0004386,GO:0005524,GO:0008152,GO:0016787		LKJ
FPS10_08105	BLUF domain-containing protein			
FPS10_08110	ribonuclease J	GO:0003723,GO:0046872	K12574	R
FPS10_08115	type III pantothenate kinase	GO:0000166,GO:0004594,GO:0005524,GO:0005737,GO:00159[illegible]7,GO:0016301,GO:0016310,GO:0016740,GO:0043169,GO:0046872	K03525	K
FPS10_08120	biotin--[acetyl-CoA-carboxylase] ligase	GO:0004077,GO:0006464,GO:0016874	K03524	H
FPS10_08125	NADH-quinone oxidoreductase subunit NuoN	GO:0005886,GO:0006810,GO:0008137,GO:0016020,GO:00160[illegible]1,GO:0016491,GO:0042773,GO:0048038,GO:0050136,GO:0055114	K00343	C
FPS10_08130	NADH-quinone oxidoreductase subunit M	GO:0006120,GO:0008137,GO:0016020,GO:0016021,GO:0016491,GO:0042773,GO:0055114	K00342	C
FPS10_08135	NADH-quinone oxidoreductase subunit L		K00341	CP
FPS10_08140	NADH-quinone oxidoreductase subunit NuoK	GO:0005886,GO:0006810,GO:0016020,GO:0016021,GO:00164[illegible]1,GO:0016651,GO:0042773,GO:0048038,GO:0050136,GO:0055114	K00340	C
FPS10_08145	NADH-quinone oxidoreductase subunit J	GO:0008137,GO:0016020,GO:0016021,GO:0016491,GO:00551[illegible]4	K00339	C
FPS10_08150	carboxymuconolactone decarboxylase family protein	GO:0051920,GO:0055114,GO:0098869	K01607	S
FPS10_08155	NADH-quinone oxidoreductase subunit NuoI	GO:0005506,GO:0005886,GO:0016020,GO:0016491,GO:0016651,GO:0046872,GO:0048038,GO:0050136,GO:0051536,GO:0051539,GO:0055114	K00338	C
FPS10_08160	NADH-quinone oxidoreductase subunit NuoH	GO:0005886,GO:0016020,GO:0016021,GO:0016491,GO:0016655,GO:0048038,GO:0055114	K00337	C
FPS10_08165	hypothetical protein			
FPS10_08170	NADH-quinone oxidoreductase subunit G		K00336	C
FPS10_08175	SDR family oxidoreductase			IQR
FPS10_08180	hypothetical protein			
FPS10_08185	NADH-quinone oxidoreductase subunit NuoF	GO:0008137,GO:0010181,GO:0016651,GO:0046872,GO:0051287,GO:0051536,GO:0051539,GO:0055114	K00335	C
FPS10_08190	hypothetical protein	GO:0016020,GO:0016021		
FPS10_08195	NADH-quinone oxidoreductase subunit E		K00334	C
FPS10_08200	NADH-quinone oxidoreductase subunit D	GO:0005886,GO:0006810,GO:0016020,GO:0016491,GO:0016651,GO:0048038,GO:0050136,GO:0051287,GO:0055114	K00333	C
FPS10_08205	thioesterase		K18700	R
FPS10_08210	NADH-quinone oxidoreductase subunit C		K00332	C
FPS10_08215	NADH-quinone oxidoreductase subunit B	GO:0005506,GO:0005886,GO:0006810,GO:0008137,GO:0016020,GO:0016491,GO:0046872,GO:0048038,GO:0050136,GO:0051536,GO:0051539,GO:0055114	K00331	C

FPS10_08220	NADH-quinone oxidoreductase subunit A	GO:0005886,GO:0006810,GO:0008137,GO:0016020,GO:0016021,GO:0016491,GO:0016651,GO:0048038,GO:0050136,GO:0055114	K00330	C
FPS10_08225	amidohydrolase	GO:0008152,GO:0016787,GO:0016810		R
FPS10_08230	crotonase/enoyl-CoA hydratase family protein	GO:0003824,GO:0004300,GO:0008152,GO:0016829	K13766	I
FPS10_08235	hydroxymethylglutaryl-CoA lyase	GO:0003824,GO:0008152,GO:0016829	K01640	E
FPS10_08240	acetyl/propionyl/methylcrotonyl-CoA carboxylase subunit alpha	GO:0003824,GO:0004075,GO:0005524,GO:0008152,GO:0046872	K01968	I
FPS10_08245	methylcrotonoyl-CoA carboxylase	GO:0004485,GO:0006552,GO:0016874	K01969	I
FPS10_08250	EcsC family protein			
FPS10_08255	AMP-binding protein	GO:0003824,GO:0008152	K01895	I
FPS10_08260	hypothetical protein			
FPS10_08265	DUF1311 domain-containing protein			S
FPS10_08270	DUF1622 domain-containing protein	GO:0016020,GO:0016021		S
FPS10_08275	isovaleryl-CoA dehydrogenase	GO:0003995,GO:0008152,GO:0016491,GO:0016627,GO:0050660,GO:0055114	K00253	I
FPS10_08280	GFA family protein			S
FPS10_08285	excinuclease ABC subunit UvrA	GO:0000166,GO:0003677,GO:0003824,GO:0004518,GO:0005524,GO:0005737,GO:0006281,GO:0006289,GO:0006974,GO:0008270,GO:0009380,GO:0009381,GO:0009432,GO:0016887,GO:0046872,GO:0090305	K03701	L
FPS10_08290	helix-turn-helix transcriptional regulator	GO:0003677,GO:0003700,GO:0006351,GO:0006355	K03892	K
FPS10_08295	permease	GO:0016020,GO:0016021	K07089	R
FPS10_08300	thioredoxin family protein	GO:0008152,GO:0016853		
FPS10_08305	glycine zipper 2TM domain-containing protein			
FPS10_08310	hypothetical protein			
FPS10_08315	dihydrolipoyl dehydrogenase	GO:0004148,GO:0006086,GO:0006096,GO:0016491,GO:0016668,GO:0045250,GO:0045454,GO:0050660,GO:0055114	K00382	C
FPS10_08320	DUF924 domain-containing protein	GO:0003674,GO:0005575,GO:0008150		S
FPS10_08325	MFS transporter	GO:0016020,GO:0016021,GO:0055085		
FPS10_08330	hypothetical protein			R
FPS10_08335	GNAT family N-acetyltransferase			R
FPS10_08340	hypothetical protein			
FPS10_08345	tRNA preQ1(34) S-adenosylmethionine ribosyltransferase-isomerase QueA	GO:0005737,GO:0008616,GO:0016740,GO:0016853	K07568	J
FPS10_08350	hypothetical protein			
FPS10_08355	thioredoxin-dependent thiol peroxidase	GO:0016209,GO:0016491,GO:0055114,GO:0098869	K03564	O
FPS10_08360	ferritin-like domain-containing protein			S
FPS10_08365	peptidoglycan DD-metalloendopeptidase family protein	GO:0016020,GO:0016021		M
FPS10_08370	polymer-forming cytoskeletal protein			M
FPS10_08375	hypothetical protein			
FPS10_08380	DUF2189 domain-containing protein	GO:0016020,GO:0016021		S
FPS10_08385	pyridoxal-phosphate dependent enzyme		K01738	E
FPS10_08390	DMT family transporter	GO:0016020,GO:0016021		GER
FPS10_08395	peptide chain release factor 2	GO:0003747,GO:0005737,GO:0006412,GO:0006415,GO:0016149	K02836	J
FPS10_08400	PBP1A family penicillin-binding protein	GO:0008152,GO:0008658,GO:0016020,GO:0016021,GO:0016740,GO:0016757	K05366	M
FPS10_08405	class I SAM-dependent methyltransferase	GO:0008168,GO:0016740,GO:0032259		QR
FPS10_08410	N-acetylmuramoyl-L-alanine amidase	GO:0008745,GO:0009253	K01448	M
FPS10_08415	aminotransferase class I/II-fold pyridoxal phosphate-dependent enzyme	GO:0003824,GO:0008483,GO:0009058,GO:0016740,GO:0030170		E
FPS10_08420	DsbA family protein			O
FPS10_08425	glycerophosphodiester phosphodiesterase family protein		K01126	C
FPS10_08430	flavodoxin-dependent (E)-4-hydroxy-3-methylbut-2-enyl-diphosphate synthase		K03526	I
FPS10_08435	DUF4115 domain-containing protein	GO:0003677,GO:0016020,GO:0016021		S
FPS10_08440	5-aminolevulinate synthase	GO:0003824,GO:0003870,GO:0006782,GO:0006783,GO:0008132,GO:0009058,GO:0016740,GO:0016746,GO:0030170,GO:0033014	K00643	H
FPS10_08445	hypothetical protein			
FPS10_08450	LuxR family transcriptional regulator			K
FPS10_08455				
FPS10_08460	acyl-CoA carboxylase subunit beta	GO:0003989,GO:0004658,GO:0006633,GO:0009317,GO:0016740,GO:0016874		I
FPS10_08465	hypothetical protein		K23352	
FPS10_08470	acetyl-CoA carboxylase biotin carboxyl carrier protein subunit		K23351	I
FPS10_08475	sodium ion-translocating decarboxylase subunit beta	GO:0006814,GO:0008152,GO:0016020,GO:0016021,GO:0016829	K20509	C
FPS10_08480	hypothetical protein			
FPS10_08485	ArsB/NhaD family transporter	GO:0015105,GO:0015700,GO:0016020,GO:0016021,GO:0055085		P

FPS10_08490	CBS domain-containing protein			R
FPS10_08495	M48 family metallopeptidase	GO:0004222,GO:0006508,GO:0008233,GO:0008237,GO:00167[illegible]	K07387	R
FPS10_08500	DMT family transporter			GER
FPS10_08505	VWA domain-containing protein		K09989	S
FPS10_08510	NAD-dependent epimerase/dehydratase family protein			MG
FPS10_08515	apolipoprotein acyltransferase			
FPS10_08520	DUF2927 domain-containing protein			
FPS10_08525	GNAT family N-acetyltransferase	GO:0008080,GO:0008152,GO:0016740,GO:0016746		R
FPS10_08530	DMT family transporter	GO:0016020,GO:0016021		GER
FPS10_08535	YdiU family protein			S
FPS10_08540	PIN domain-containing protein			
FPS10_08545	class I SAM-dependent rRNA methyltransferase	GO:0005737,GO:0006364,GO:0008168,GO:0016740,GO:00322[illegible]	K06969	R
FPS10_08550	phosphogluconate dehydratase	GO:0003824,GO:0004456,GO:0004792,GO:0008152,GO:0009255,GO:0016829	K01690	EG
FPS10_08555	bifunctional 4-hydroxy-2-oxoglutarate aldolase/2-dehydro-3-deoxy-phosphogluconate aldolase	GO:0003824,GO:0008152,GO:0008675,GO:0008700,GO:0016829	K01625	G
FPS10_08560	hypothetical protein			
FPS10_08565	glutamine-synthetase adenylyltransferase	GO:0000166,GO:0005524,GO:0008152,GO:0008882,GO:0016740,GO:0016779,GO:0016874	K00982	OT
FPS10_08570	YbaK/EbsC family protein			S
FPS10_08575	DUF2852 domain-containing protein	GO:0016020,GO:0016021		
FPS10_08580	RDD family protein			
FPS10_08585	arginyltransferase	GO:0004057,GO:0005737,GO:0016598,GO:0016740,GO:0016746,GO:0030163	K21420	O
FPS10_08590	hypothetical protein			
FPS10_08595	TRAP transporter large permease subunit	GO:0016020,GO:0016021		Q
FPS10_08600	C4-dicarboxylate ABC transporter permease	GO:0016020,GO:0016021		Q
FPS10_08605	twin-arginine translocation signal domain-containing protein	GO:0006810,GO:0030288,GO:0042597,GO:0046872		Q
FPS10_08610	histidine kinase	GO:0000155,GO:0000160,GO:0005622,GO:0007165,GO:0016020,GO:0016021,GO:0016301,GO:0016310,GO:0016740,GO:0023014,GO:0046983	K02480	T
FPS10_08615	response regulator transcription factor	GO:0000160,GO:0003677,GO:0005622,GO:0006351,GO:00063[illegible]		TK
FPS10_08620	HAMP domain-containing protein	GO:0000155,GO:0000160,GO:0004871,GO:0005622,GO:0006935,GO:0007165,GO:0016020,GO:0023014	K03406	NT
FPS10_08630	Crp/Fnr family transcriptional regulator			T
FPS10_08635	hypothetical protein			
FPS10_08640				
FPS10_08645	DUF192 domain-containing protein		K09005	S
FPS10_08650	cold shock domain-containing protein	GO:0003676,GO:0003677,GO:0005737,GO:0006355	K03704	K
FPS10_08655	pyridoxamine 5'-phosphate oxidase	GO:0004733,GO:0008615,GO:0010181,GO:0016491,GO:0016638,GO:0042816,GO:0042823,GO:0055114	K00275	H
FPS10_08660	enoyl-ACP reductase FabI	GO:0004318,GO:0006633,GO:0016491,GO:0055114	K00208	I
FPS10_08665	xanthine phosphoribosyltransferase	GO:0000287,GO:0000310,GO:0005886,GO:0006166,GO:00091[illegible]6,GO:0016020,GO:0016740,GO:0016757,GO:0032265,GO:00468[illegible]	K00769	R
FPS10_08670	DUF560 domain-containing protein			
FPS10_08675	aminotransferase	GO:0003824,GO:0009058,GO:0030170		E
FPS10_08680	peptidylprolyl isomerase		K03770	O
FPS10_08685	anthranilate synthase component I	GO:0000162,GO:0004049,GO:0009058,GO:0016829,GO:00168[illegible]	K01657	EH
FPS10_08690	AI-2E family transporter			R
FPS10_08695	metal-dependent hydrolase	GO:0008152,GO:0016787		R
FPS10_08700	cardiolipin synthase	GO:0003824,GO:0005886,GO:0008654,GO:0008808,GO:0016020,GO:0016021,GO:0016740,GO:0016780,GO:0032049	K06131	I
FPS10_08705	hypothetical protein			S
FPS10_08710	aminodeoxychorismate/anthranilate synthase component II	GO:0004049,GO:0006541,GO:0008152,GO:0016740,GO:00168[illegible]	K01658	EH
FPS10_08715	anthranilate phosphoribosyltransferase	GO:0000162,GO:0000287,GO:0004048,GO:0008152,GO:0008652,GO:0009073,GO:0016740,GO:0016757,GO:0046872	K00766	E
FPS10_08720	uracil-DNA glycosylase	GO:0004844,GO:0005737,GO:0006281,GO:0006284,GO:0006974,GO:0008152,GO:0016787,GO:0016798	K03648	L
FPS10_08725	indole-3-glycerol phosphate synthase TrpC	GO:0000162,GO:0003824,GO:0004425,GO:0006568,GO:0008152,GO:0008652,GO:0009073,GO:0016829,GO:0016831	K01609	E
FPS10_08730	cyclic pyranopterin monophosphate synthase MoaC	GO:0006777	K03637	H
FPS10_08735	molybdopterin molybdotransferase MoeA	GO:0032324	K03750	H
FPS10_08740	transcriptional repressor LexA	GO:0003677,GO:0004252,GO:0006260,GO:0006281,GO:0006351,GO:0006355,GO:0006508,GO:0006974,GO:0009432,GO:0016787,GO:0045892	K01356	KT
FPS10_08745	ComEC family competence protein	GO:0016020,GO:0016021	K02238	R
FPS10_08750	glutamate--tRNA ligase	GO:0000049,GO:0000166,GO:0004812,GO:0004818,GO:0005524,GO:0005737,GO:0006412,GO:0006418,GO:0006424,GO:0016874,GO:0016876,GO:0043039	K01885	J

FPS10_08755	citrate (Si)-synthase		K01647	C
FPS10_08760	hypothetical protein			
FPS10_08765	hypothetical protein			
FPS10_08770	carboxylesterase	GO:0016020,GO:0016021		
FPS10_08775	peptidoglycan-binding protein			R
FPS10_08780	CidA/LrgA family protein	GO:0005886,GO:0016020,GO:0016021		R
FPS10_08785	LrgB family protein	GO:0016020,GO:0016021		M
FPS10_08790	cytochrome c maturation protein CcmE	GO:0005886,GO:0016020,GO:0016021,GO:0017003,GO:0017004,GO:0046872	K02197	O
FPS10_08795	N-acetyl-gamma-glutamyl-phosphate reductase	GO:0003942,GO:0005737,GO:0006526,GO:0008652,GO:0016491,GO:0016620,GO:0046983,GO:0051287,GO:0055114	K00145	E
FPS10_08800	glutamate racemase	GO:0006807,GO:0008152,GO:0008360,GO:0008881,GO:0009252,GO:0016853,GO:0016855,GO:0036361,GO:0071555	K01776	M
FPS10_08805	acyltransferase	GO:0008152,GO:0016740,GO:0016746		R
FPS10_08810	LysR family transcriptional regulator	GO:0003677,GO:0003700,GO:0006351,GO:0006355		K
FPS10_08815	lytic murein transglycosylase			M
FPS10_08820	phosphoribosylformylglycinamidine synthase subunit PurL	GO:0000166,GO:0000287,GO:0004642,GO:0005524,GO:0005737,GO:0006164,GO:0006189,GO:0016874,GO:0046872	K23269	F
FPS10_08825	thymidylate synthase	GO:0004799,GO:0005737,GO:0006231,GO:0006235,GO:0008168,GO:0009165,GO:0016740,GO:0032259	K00560	F
FPS10_08830	dihydrofolate reductase		K00287	H
FPS10_08835	hypothetical protein			
FPS10_08840	DUF560 domain-containing protein			O
FPS10_08845	lactoylglutathione lyase	GO:0004462,GO:0008152,GO:0046872,GO:0051213,GO:00551[illegible]4	K01759	E
FPS10_08850	DUF1194 domain-containing protein			
FPS10_08855	MarR family transcriptional regulator	GO:0003677,GO:0003700,GO:0006351,GO:0006355		K
FPS10_08860	succinate dehydrogenase assembly factor 2		K09159	S
FPS10_08865	helix-turn-helix domain-containing protein	GO:0003677,GO:0043565		K
FPS10_08870	pyridoxal phosphate-dependent aminotransferase	GO:0003824,GO:0004069,GO:0008483,GO:0009058,GO:0016740,GO:0030170,GO:0080130	K00812	E
FPS10_08875	hypothetical protein			
FPS10_08880	MATE family efflux transporter	GO:0006855,GO:0015238,GO:0015297,GO:0016020,GO:0016021,GO:0055085	K03327	V
FPS10_08885	DNA topoisomerase IV subunit B	GO:0000166,GO:0000287,GO:0003677,GO:0003916,GO:0003918,GO:0005524,GO:0005694,GO:0006265,GO:0007059,GO:0016853,GO:0046872	K02622	L
FPS10_08890	hypothetical protein			OU
FPS10_08895	RNA methyltransferase		K07444	L
FPS10_08900	hypothetical protein			
FPS10_08905	aminopeptidase N	GO:0004177,GO:0006508,GO:0008237,GO:0008270,GO:0016787	K01256	E
FPS10_08910	glycerol acyltransferase	GO:0008152,GO:0016740,GO:0016746		R
FPS10_08915	LysE family translocator			E
FPS10_08920	Lrp/AsnC family transcriptional regulator		K03719	K
FPS10_08925	lytic transglycosylase domain-containing protein			M
FPS10_08930	Asp-tRNA(Asn)/Glu-tRNA(Gln) amidotransferase subunit GatB	GO:0000166,GO:0005524,GO:0006412,GO:0016874,GO:0016884,GO:0050567	K02434	J
FPS10_08935	DUF4177 domain-containing protein			
FPS10_08940	BolA family transcriptional regulator		K05527	T
FPS10_08945	J domain-containing protein			O
FPS10_08950	hypothetical protein			
FPS10_08955	cobaltochelatase subunit CobS	GO:0005524,GO:0008152,GO:0016887	K09882	R
FPS10_08960	DUF427 domain-containing protein			S
FPS10_08965	aminopeptidase P family protein	GO:0008152,GO:0016787	K01262	E
FPS10_08970	cobaltochelatase subunit CobT	GO:0009236	K09883	H
FPS10_08975	CHAD domain-containing protein			S
FPS10_08980	type VI secretion protein			S
FPS10_08985	hypothetical protein			
FPS10_08990	hypothetical protein			
FPS10_08995	chloride channel protein		K03281	P
FPS10_09000	FAD-binding protein	GO:0003824,GO:0004458,GO:0016491,GO:0016614,GO:0050660,GO:0055114	K00102	C
FPS10_09005	DNA repair protein RecN	GO:0005524,GO:0006281,GO:0006310,GO:0006974	K03631	L
FPS10_09010	outer membrane protein assembly factor BamD	GO:0009279,GO:0016020,GO:0043165,GO:0051205	K05807	R
FPS10_09015	UDP-3-O-acyl-N-acetylglucosamine deacetylase	GO:0006629,GO:0008759,GO:0009245,GO:0016787,GO:00468[illegible]2	K02535	M
FPS10_09020	cell division protein FtsZ	GO:0000166,GO:0000917,GO:0003924,GO:0005525,GO:0005737,GO:0007049,GO:0008152,GO:0032153,GO:0043093,GO:0051258,GO:0051301	K03531	D

FPS10_09025	cell division protein FtsA	GO:0005886,GO:0007049,GO:0008360,GO:0009898,GO:0016020,GO:0032153,GO:0043093,GO:0051301	K03590	D
FPS10_09030	cell division protein FtsQ	GO:0000917,GO:0005886,GO:0005887,GO:0007049,GO:0016020,GO:0016021,GO:0032153,GO:0043093,GO:0051301	K03589	M
FPS10_09035	D-alanine--D-alanine ligase		K01921	M
FPS10_09040	UDP-N-acetylmuramate dehydrogenase		K00075	M
FPS10_09045	DUF2484 family protein	GO:0016020,GO:0016021		
FPS10_09050	DUF2484 family protein	GO:0016020,GO:0016021		
FPS10_09055	UDP-N-acetylmuramate--L-alanine ligase	GO:0000166,GO:0005524,GO:0005737,GO:0007049,GO:0008360,GO:0008763,GO:0009058,GO:0009252,GO:0016874,GO:0051301,GO:0071555	K01924	M
FPS10_09060	UDP-N-acetylglucosamine--N-acetylmuramyl-(pentapeptide) pyrophosphoryl-undecaprenol N-acetylglucosamine transferase	GO:0005886,GO:0005975,GO:0007049,GO:0008360,GO:0009252,GO:0016020,GO:0016740,GO:0016757,GO:0016758,GO:0030259,GO:0050511,GO:0051301,GO:0051991,GO:0071555	K02563	M
FPS10_09065	putative lipid II flippase FtsW	GO:0005886,GO:0008360,GO:0009252,GO:0016020,GO:0016021,GO:0051301	K03588	D
FPS10_09070	NAD(P)/FAD-dependent oxidoreductase	GO:0016491,GO:0055114		R
FPS10_09075	UDP-N-acetylmuramoyl-L-alanine--D-glutamate ligase		K01925	M
FPS10_09080	SDR family NAD(P)-dependent oxidoreductase	GO:0004316,GO:0008152,GO:0016491,GO:0055114		IQR
FPS10_09085	phospho-N-acetylmuramoyl-pentapeptide- transferase	GO:0005886,GO:0007049,GO:0008360,GO:0008963,GO:0009252,GO:0016020,GO:0016021,GO:0016740,GO:0051301,GO:0051992,GO:0071555	K01000	M
FPS10_09090	UDP-N-acetylmuramoyl-tripeptide--D-alanyl-D- alanine ligase	GO:0000166,GO:0005524,GO:0005737,GO:0007049,GO:0008360,GO:0009058,GO:0009252,GO:0016874,GO:0047480,GO:0051301,GO:0071555	K01929	M
FPS10_09095	UDP-N-acetylmuramoyl-L-alanyl-D-glutamate--2%2C 6-diaminopimelate ligase	GO:0000166,GO:0005524,GO:0005737,GO:0007049,GO:0008360,GO:0009058,GO:0009252,GO:0016874,GO:0016881,GO:0051301,GO:0071555	K01928	M
FPS10_09100	penicillin-binding protein 2	GO:0008152,GO:0008658,GO:0008955,GO:0016020,GO:0016021,GO:0016740,GO:0016757,GO:0051301	K03587	M
FPS10_09105	cell division protein FtsL			S
FPS10_09110	16S rRNA (cytosine(1402)-N(4))-methyltransferase RsmH	GO:0005737,GO:0006364,GO:0008168,GO:0016740,GO:0032259,GO:0070475,GO:0071424	K03438	M
FPS10_09115	division/cell wall cluster transcriptional repressor MraZ		K03925	S
FPS10_09120	Mrp/NBP35 family ATP-binding protein	GO:0000166,GO:0005524,GO:0008152,GO:0016787,GO:0016887,GO:0046872,GO:0051536	K03593	D
FPS10_09125	DUF1127 domain-containing protein			
FPS10_09130	DUF1127 domain-containing protein			
FPS10_09135	DUF1127 domain-containing protein			S
FPS10_09140	DUF1127 domain-containing protein			S
FPS10_09145	response regulator			T
FPS10_09150	AAA family ATPase		K03657	L
FPS10_09155	pyruvate carboxylase	GO:0000166,GO:0003677,GO:0003824,GO:0004075,GO:0004736,GO:0005524,GO:0006090,GO:0006094,GO:0009374,GO:0016874,GO:0046872	K01958	C
FPS10_09160	GFA family protein	GO:0008152,GO:0016846		S
FPS10_09165	DUF2147 domain-containing protein			S
FPS10_09170	1-(5-phosphoribosyl)-5-[(5-phosphoribosylamino)methylideneamino]imidazole-4-carboxamide isomerase		K01814	E
FPS10_09175	DUF302 domain-containing protein			S
FPS10_09180	imidazole glycerol phosphate synthase subunit HisF	GO:0000105,GO:0000107,GO:0003824,GO:0005737,GO:0008152,GO:0008652,GO:0016829	K02500	E
FPS10_09185	phosphoribosyl-ATP diphosphatase	GO:0000105,GO:0000166,GO:0004636,GO:0005524,GO:0005737,GO:0008652,GO:0016787	K01523	E
FPS10_09190	CoA-binding protein	GO:0048037	K06929	R
FPS10_09195	hypothetical protein			
FPS10_09200	23S rRNA (guanosine(2251)-2'-O)-methyltransferase RlmB	GO:0001510,GO:0003723,GO:0006396,GO:0008168,GO:0008173,GO:0016740,GO:0032259	K03218	J
FPS10_09205	metal ABC transporter permease	GO:0005524,GO:0005886,GO:0006810,GO:0016020,GO:0016021,GO:0042626,GO:0055085	K11709	P
FPS10_09210	metal ABC transporter permease		K11708	P
FPS10_09215	metal ABC transporter ATP-binding protein	GO:0000166,GO:0005524,GO:0008152,GO:0016887	K11710	P
FPS10_09220	manganese transporter	GO:0006810,GO:0007155,GO:0030001,GO:0046872	K11707	P
FPS10_09225				
FPS10_09230	hypothetical protein			
FPS10_09235	hypothetical protein			
FPS10_09240	GTP-binding protein			R
FPS10_09250	hypothetical protein			
FPS10_09255	hypothetical protein			

FPS10_09260	transposase			
FPS10_09265	transposase			
FPS10_09270	hypothetical protein			
FPS10_09275	hypothetical protein			
FPS10_09280	hypothetical protein			
FPS10_09285	transposase			L
FPS10_09290	hypothetical protein			
FPS10_09295	transcriptional activator RfaH	GO:0006351,GO:0006353,GO:0006355,GO:0031564,GO:0032784	K05785	K
FPS10_09300	MarR family EPS-associated transcriptional regulator			K
FPS10_09305	hypothetical protein			
FPS10_09310	hypothetical protein			R
FPS10_09315	capsular biosynthesis protein	GO:0008152,GO:0016787		
FPS10_09320	hypothetical protein			
FPS10_09325	capsular biosynthesis protein			I
FPS10_09330	hypothetical protein			
FPS10_09335	hypothetical protein			
FPS10_09340	sulfotransferase			
FPS10_09345	hypothetical protein			
FPS10_09350	glycosyltransferase	GO:0008152,GO:0016740		M
FPS10_09355	hypothetical protein			
FPS10_09360	polysaccharide biosynthesis tyrosine autokinase		K16554	D
FPS10_09365	GDP-mannose 4%2C6-dehydratase		K01711	M
FPS10_09370	GDP-L-fucose synthase	GO:0003824,GO:0008152,GO:0016491,GO:0016853,GO:0042351,GO:0050577,GO:0050662,GO:0055114,GO:0070401	K02377	MG
FPS10_09375	tyrosine-type recombinase/integrase			L
FPS10_09380	hypothetical protein			
FPS10_09385	helix-turn-helix domain-containing protein			
FPS10_09390	hypothetical protein			
FPS10_09395	VRR-NUC domain-containing protein			
FPS10_09400	helix-turn-helix domain-containing protein			
FPS10_09405	hypothetical protein			
FPS10_09410	hypothetical protein		K09004	S
FPS10_09415	cytochrome c			C
FPS10_09420	hypothetical protein			C
FPS10_09425	cytochrome c-type protein NapC		K02569	C
FPS10_09430	sulfurtransferase			P
FPS10_09435	CIA30 family protein			
FPS10_09440	DUF1311 domain-containing protein			S
FPS10_09445	DUF3489 domain-containing protein			
FPS10_09450	type 1 glutamine amidotransferase domain-containing protein			R
FPS10_09455	NADP-dependent oxidoreductase	GO:0008270,GO:0016491,GO:0055114	K23256	R
FPS10_09460	TetR/AcrR family transcriptional regulator	GO:0003677,GO:0006351,GO:0006355	K16137	K
FPS10_09465	hypothetical protein			E
FPS10_09470	aminotransferase class I/II-fold pyridoxal phosphate-dependent enzyme			E
FPS10_09475	aminotransferase class I/II-fold pyridoxal phosphate-dependent enzyme			E
FPS10_09480	aminotransferase class I/II-fold pyridoxal phosphate-dependent enzyme			E
FPS10_09485	hypothetical protein			
FPS10_09490	ATP-dependent zinc metalloprotease FtsH		K03798	O
FPS10_09495	alkaline phosphatase family protein			R
FPS10_09500	HlyD family secretion protein			V
FPS10_09505	hypothetical protein		K04102	P
FPS10_09510	sulfatase-like hydrolase/transferase		K01130	P
FPS10_09515	hypothetical protein			NU
FPS10_09520	alpha/beta hydrolase			R
FPS10_09525	helix-turn-helix transcriptional regulator			
FPS10_09530	site-specific integrase	GO:0003677,GO:0006310,GO:0015074		L
FPS10_09535	hypothetical protein			
FPS10_09540	ABC transporter substrate-binding protein		K01999	E
FPS10_09545	helix-turn-helix domain-containing protein			K
FPS10_09550	transporter			
FPS10_09555	D-glutamate deacylase			Q
FPS10_09560	hydrolase			Q
FPS10_09565	DUF3299 domain-containing protein		K09950	S

FPS10_09570	amidohydrolase family protein			Q
FPS10_09575	hypothetical protein			
FPS10_09580	alpha/beta hydrolase			S
FPS10_09585	ATP-binding cassette domain-containing protein		K02003	V
FPS10_09590	ABC transporter permease	GO:0005886,GO:0016020,GO:0016021	K02004	V
FPS10_09595	DUF3299 domain-containing protein		K09950	S
FPS10_09600	mechanosensitive ion channel family protein		K16052	M
FPS10_09605	LysR family transcriptional regulator	GO:0003677,GO:0003700,GO:0006351,GO:0006355	K03717	K
FPS10_09610	oxidoreductase	GO:0008137,GO:0016020,GO:0016021,GO:0016491,GO:0055114	K05577	CP
FPS10_09615	DUF2309 domain-containing protein		K09822	S
FPS10_09620	DUF1214 domain-containing protein			S
FPS10_09625	DUF1214 domain-containing protein			S
FPS10_09630	glycine zipper 2TM domain-containing protein	GO:0019867		
FPS10_09635	AraC family transcriptional regulator			K
FPS10_09640	hypothetical protein	GO:0003677,GO:0004803,GO:0006313		
FPS10_09645	hypothetical protein			
FPS10_09650	hypothetical protein			
FPS10_09655	META domain-containing protein			M
FPS10_09660	hypothetical protein			
FPS10_09665	hypothetical protein			
FPS10_09670	hypothetical protein			
FPS10_09675	hypothetical protein	GO:0016020,GO:0016021		
FPS10_09680	GlsB/YeaQ/YmgE family stress response membrane protein			S
FPS10_09690	IS1182 family transposase			L
FPS10_09700	crotonase/enoyl-CoA hydratase family protein	GO:0003824,GO:0004300,GO:0008152,GO:0016829		I
FPS10_09705	CoA transferase			C
FPS10_09710	NIPSNAP family protein			
FPS10_09715	aldehyde dehydrogenase family protein	GO:0008152,GO:0016491,GO:0016620,GO:0055114	K22187	C
FPS10_09720	hypothetical protein		K09780	S
FPS10_09725	tripartite tricarboxylate transporter substrate binding protein			S
FPS10_09730	tripartite tricarboxylate transporter permease	GO:0016020,GO:0016021		S
FPS10_09735	tripartite tricarboxylate transporter TctB family protein			
FPS10_09740	Ldh family oxidoreductase	GO:0008152,GO:0016491,GO:0055114		C
FPS10_09745	alpha/beta fold hydrolase	GO:0008152,GO:0016787	K06889	I
FPS10_09750	NAD(P)H-dependent oxidoreductase		K19784	R
FPS10_09755	glyoxalase			
FPS10_09760	fumarylacetoacetate hydrolase family protein	GO:0003824,GO:0008152		Q
FPS10_09765	FCD domain-containing protein			K
FPS10_09770	FAD-binding oxidoreductase			C
FPS10_09775	transposase			
FPS10_09780	transposase			L
FPS10_09785	transposase			L
FPS10_09790	hypothetical protein			
FPS10_09795	hypothetical protein			
FPS10_09800	hypothetical protein			
FPS10_09805	hypothetical protein			
FPS10_09810	LysR family transcriptional regulator	GO:0003677,GO:0003700,GO:0006351,GO:0006355		K
FPS10_09815	transporter substrate-binding domain-containing protein		K02030	ET
FPS10_09820	creatininase family protein		K01470	R
FPS10_09825	amino acid ABC transporter permease	GO:0005215,GO:0005886,GO:0006810,GO:0016020,GO:0016021	K02029	E
FPS10_09830	amino acid ABC transporter permease	GO:0005215,GO:0005886,GO:0006810,GO:0016020,GO:0016021	K02029	E
FPS10_09835	amino acid ABC transporter ATP-binding protein			E
FPS10_09840	aspartate aminotransferase family protein			H
FPS10_09845	GNAT family N-acetyltransferase			R
FPS10_09850	CoA transferase subunit A	GO:0008152,GO:0008410,GO:0016740	K01034	I
FPS10_09855	3-oxoacid CoA-transferase subunit B		K01029	I
FPS10_09860	ArgE/DapE family deacylase	GO:0008152,GO:0008777,GO:0016787,GO:0016813	K01438	E
FPS10_09865	acetyl-CoA C-acyltransferase	GO:0003824,GO:0003988,GO:0008152,GO:0016740,GO:0016746,GO:0016747	K00626	I
FPS10_09870	D-lyxose/D-mannose family sugar isomerase		K09988	R
FPS10_09875	sn-glycerol-3-phosphate ABC transporter ATP-binding protein UgpC	GO:0000166,GO:0005215,GO:0005524,GO:0006810,GO:0008152,GO:0008643,GO:0016820,GO:0016887,GO:0043190,GO:0055085	K10112	G
FPS10_09880	carbohydrate ABC transporter permease		K17317	G
FPS10_09885	sugar ABC transporter permease		K17316	G
FPS10_09890	carbohydrate ABC transporter substrate-binding protein		K17315	G
FPS10_09895	LacI family DNA-binding transcriptional regulator		K02529	K

FPS10_09900	M56 family metallopeptidase			KT
FPS10_09905	BlaI/MecI/CopY family transcriptional regulator	GO:0003677,GO:0045892		K
FPS10_09910	hypothetical protein	GO:0003674,GO:0005575,GO:0008150,GO:0016020,GO:0016021		S
FPS10_09915	carbohydrate ABC transporter permease	GO:0005351,GO:0005886,GO:0006810,GO:0008643,GO:0015992,GO:0016020,GO:0016021	K17323	G
FPS10_09920	sugar ABC transporter permease	GO:0005886,GO:0006810,GO:0016020,GO:0016021	K17322	G
FPS10_09925	ABC transporter ATP-binding protein	GO:0000166,GO:0005215,GO:0005524,GO:0006810,GO:0008152,GO:0016820,GO:0016887,GO:0043190,GO:0055085	K17325	G
FPS10_09930	ABC transporter ATP-binding protein	GO:0000166,GO:0005524,GO:0006810,GO:0008152,GO:0016887	K17324	G
FPS10_09935	carbohydrate ABC transporter substrate-binding protein		K17321	G
FPS10_09940	anaerobic glycerol-3-phosphate dehydrogenase subunit B		K00112	E
FPS10_09945	anaerobic glycerol-3-phosphate dehydrogenase subunit A		K00111	C
FPS10_09950	(Fe-S)-binding protein			C
FPS10_09955	hydrogenase expression/formation protein HypE		K04655	O
FPS10_09960	carbamoyltransferase HypF	GO:0003725,GO:0008270,GO:0016743,GO:0046872,GO:0046944	K04656	O
FPS10_09965	hydrogenase formation protein HypD	GO:0046872	K04654	O
FPS10_09970	hydrogenase nickel incorporation protein HypB		K04652	OK
FPS10_09975	hydrogenase maturation nickel metallochaperone HypA	GO:0006464,GO:0016151	K04651	R
FPS10_09980	HypC/HybG/HupF family hydrogenase formation chaperone		K04653	O
FPS10_09985	hydrogenase maturation protease		K03605	C
FPS10_09990	rhodanese-like domain-containing protein			P
FPS10_09995	hypothetical protein		K07112	R
FPS10_10000	TusE/DsrC/DsvC family sulfur relay protein		K23077	P
FPS10_10005	sulfurtransferase complex subunit TusB		K07237	P
FPS10_10010	sulfurtransferase complex subunit TusC		K07236	P
FPS10_10015	sulfurtransferase complex subunit TusD	GO:0005737,GO:0008033,GO:0016740,GO:0016783	K07235	P
FPS10_10020	sulfurtransferase TusA family protein		K04085	O
FPS10_10025	peroxiredoxin family protein	GO:0016020,GO:0016021		S
FPS10_10030	nickel-dependent hydrogenase large subunit		K06281	C
FPS10_10035	(Fe-S)-binding protein	GO:0051536		C
FPS10_10040	respiratory nitrate reductase subunit gamma			
FPS10_10045	hydrogenase small subunit	GO:0008901,GO:0009375,GO:0016491,GO:0033748,GO:0046872,GO:0051536,GO:0051538,GO:0051539,GO:0055114	K06282	C
FPS10_10050	hydrogenase expression/formation protein		K03618	
FPS10_10055	DUF167 domain-containing protein		K09131	S
FPS10_10060	AlpA family phage regulatory protein			K
FPS10_10065				
FPS10_10070	selenide%2C water dikinase SelD	GO:0005524,GO:0016301,GO:0016310,GO:0016491,GO:00551[illegible]4	K01008	E
FPS10_10075	tRNA 2-selenouridine(34) synthase MnmH	GO:0004792,GO:0016740,GO:0016783,GO:0043828,GO:0070329	K06917	R
FPS10_10080	isopenicillin N synthase family oxygenase			R
FPS10_10085	FtsX-like permease family protein	GO:0016020,GO:0016021	K02004	Q
FPS10_10090	ATP-binding cassette domain-containing protein	GO:0000166,GO:0005524,GO:0008152,GO:0016887	K02003	Q
FPS10_10095	arylesterase		K10804	E
FPS10_10100	FAD-binding oxidoreductase			C
FPS10_10105	hypothetical protein			
FPS10_10110	cytochrome C			
FPS10_10115	Crp/Fnr family transcriptional regulator			T
FPS10_10120	heme lyase CcmF/NrfE family subunit	GO:0015232,GO:0015886,GO:0016020,GO:0016021,GO:0017004,GO:0020037	K02198	O
FPS10_10125	cytochrome c-type biogenesis protein CcmH		K02200	O
FPS10_10130	c-type cytochrome biogenesis protein CcmI		K02200	O
FPS10_10135	L-glyceraldehyde 3-phosphate reductase		K19265	C
FPS10_10140	c-type cytochrome	GO:0009055,GO:0020037	K08738	R
FPS10_10145	formate dehydrogenase subunit gamma		K00127	C
FPS10_10150	4Fe-4S dicluster domain-containing protein	GO:0008863,GO:0009055,GO:0009061,GO:0009326	K00124	C
FPS10_10155	formate dehydrogenase subunit alpha	GO:0008863,GO:0016491,GO:0030151,GO:0046872,GO:0051536,GO:0051539,GO:0055114	K00123	C
FPS10_10160	twin-arginine translocation signal domain-containing protein	GO:0003674,GO:0008150		
FPS10_10165	molecular chaperone TorD family protein			R
FPS10_10170	DUF3306 domain-containing protein			
FPS10_10175	DUF3305 domain-containing protein			
FPS10_10180	4Fe-4S dicluster domain-containing protein			C
FPS10_10185	hypothetical protein	GO:0009055,GO:0020037		H
FPS10_10190	Mrp/NBP35 family ATP-binding protein	GO:0000166,GO:0005524,GO:0008152,GO:0016787,GO:0016887,GO:0046872,GO:0051536	K03593	D
FPS10_10195	DUF4444 domain-containing protein	GO:0006464		H

FPS10_10200	hypothetical protein			
FPS10_10205	sulfate ABC transporter substrate-binding protein		K05772	H
FPS10_10210	ATP-binding cassette domain-containing protein	GO:0000166,GO:0005524,GO:0008152,GO:0015408,GO:0015682,GO:0016787,GO:0016887	K06857	E
FPS10_10215	ABC transporter permease subunit		K05773	H
FPS10_10220	molybdopterin molybdenumtransferase MoeA	GO:0006777,GO:0032324	K03750	H
FPS10_10225	molybdopterin-guanine dinucleotide biosynthesis protein B	GO:0005525,GO:0006777	K03753	H
FPS10_10230	molybdenum cofactor guanylyltransferase MobA	GO:0000166,GO:0003824,GO:0005525,GO:0005737,GO:0006777,GO:0016740,GO:0019720,GO:0046872,GO:0061603,GO:0070568	K03752	H
FPS10_10235	formate dehydrogenase accessory sulfurtransferase FdhD	GO:0003824,GO:0008152,GO:0016783	K02379	C
FPS10_10245				
FPS10_10250				
FPS10_10255				
FPS10_10260				
FPS10_10265				
FPS10_10270	hypothetical protein			
FPS10_10275	IS110 family transposase	GO:0003677,GO:0004803,GO:0006313		L
FPS10_10280	HAMP domain-containing protein		K03406	NT
FPS10_10285	amino acid ABC transporter substrate-binding protein		K02030	ET
FPS10_10290	NmrA family transcriptional regulator			MG
FPS10_10295	DUF4345 domain-containing protein	GO:0016020,GO:0016021		
FPS10_10300	EamA family transporter	GO:0016020,GO:0016021	K15269	GER
FPS10_10305	NAD(P)H:quinone oxidoreductase	GO:0000166,GO:0003955,GO:0010181,GO:0016491,GO:0045892,GO:0050660,GO:0050661,GO:0051287,GO:0055114	K03809	R
FPS10_10310	LysR family transcriptional regulator	GO:0003677,GO:0003700,GO:0006351,GO:0006355		K
FPS10_10315	AraC family transcriptional regulator	GO:0003677,GO:0003700,GO:0006351,GO:0006355,GO:0043565		K
FPS10_10320	aspartate kinase	GO:0004072,GO:0016301,GO:0016310,GO:0016740	K00928	E
FPS10_10325	ectoine synthase	GO:0016829,GO:0016836,GO:0019491,GO:0033990	K06720	
FPS10_10330	diaminobutyrate--2-oxoglutarate transaminase	GO:0003824,GO:0008483,GO:0009058,GO:0016740,GO:0019491,GO:0030170,GO:0045303,GO:0047307	K00836	E
FPS10_10335	diaminobutyrate acetyltransferase		K06718	M
FPS10_10340	MarR family transcriptional regulator	GO:0003677,GO:0003700,GO:0006351,GO:0006355		K
FPS10_10345	PQQ-binding-like beta-propeller repeat protein			R
FPS10_10350	mechanosensitive ion channel		K22044	M
FPS10_10355	caspase family protein			
FPS10_10360	alpha/beta fold hydrolase			S
FPS10_10365	hypothetical protein			
FPS10_10370	CHAT domain-containing protein			S
FPS10_10375	dUTP diphosphatase	GO:0004170,GO:0009117,GO:0016787,GO:0046080,GO:0046872	K01520	F
FPS10_10380	bifunctional phosphopantothenoylcysteine decarboxylase/phosphopantothenate--cysteine ligase CoaBC	GO:0003824,GO:0004632,GO:0004633,GO:0010181,GO:0015937,GO:0015941,GO:0016829,GO:0016874	K13038	H
FPS10_10385	acyl-CoA dehydrogenase	GO:0003995,GO:0008152,GO:0016491,GO:0016627,GO:0050660,GO:0055114	K14448	I
FPS10_10390	sulfite exporter TauE/SafE family protein	GO:0016020,GO:0016021	K07090	R
FPS10_10395	molybdenum ABC transporter ATP-binding protein	GO:0000041,GO:0000166,GO:0005524,GO:0005886,GO:0006810,GO:0008152,GO:0015098,GO:0015412,GO:0015689,GO:0016020,GO:0016787,GO:0016887,GO:0030001,GO:0042888,GO:0043190	K02017	P
FPS10_10400	molybdate ABC transporter permease subunit	GO:0005886,GO:0006810,GO:0015098,GO:0015689,GO:0016020,GO:0016021	K02018	P
FPS10_10405	molybdate ABC transporter substrate-binding protein	GO:0015412,GO:0015689,GO:0030288	K02020	P
FPS10_10410	phosphoenolpyruvate carboxykinase	GO:0000166,GO:0004611,GO:0004612,GO:0005524,GO:0005737,GO:0006094,GO:0016301,GO:0016310,GO:0016829,GO:0016831,GO:0017076,GO:0046872	K01610	C
FPS10_10415	phosphoenolpyruvate carboxykinase (GTP)		K01596	C
FPS10_10420	response regulator transcription factor	GO:0000160,GO:0003677,GO:0005622,GO:0006351,GO:0006355	K14981	TK
FPS10_10425	HAMP domain-containing protein	GO:0000155,GO:0000160,GO:0000166,GO:0004673,GO:0004871,GO:0005524,GO:0005622,GO:0007165,GO:0016020,GO:0016021,GO:0016301,GO:0016310,GO:0016740,GO:0016772,GO:0018106,GO:0023014	K14980	T
FPS10_10430	hypothetical protein	GO:0000155,GO:0000160,GO:0004672,GO:0005524,GO:0005622,GO:0006109,GO:0006468,GO:0016301,GO:0016310,GO:0016740,GO:0023014		T
FPS10_10435	RNase adapter RapZ		K06958	R
FPS10_10440	PTS fructose transporter subunit IIA		K02793	G
FPS10_10445	HPr family phosphocarrier protein	GO:0005737,GO:0009401,GO:0016301,GO:0016310,GO:0016740	K11189	G
FPS10_10450	acyltransferase			R
FPS10_10455	3-hydroxybutyryl-CoA dehydrogenase	GO:0003857,GO:0006631,GO:0016491,GO:0055114,GO:0070403	K00074	I
FPS10_10460	hypothetical protein			

FPS10_10465	electron transfer flavoprotein subunit alpha	GO:0009055,GO:0050660	K03522	C
FPS10_10470	electron transfer flavoprotein subunit beta/FixA family protein	GO:0009055	K03521	C
FPS10_10475	cob(I)yrinic acid a%2Cc-diamide adenosyltransferase		K00798	S
FPS10_10480	twin transmembrane helix small protein	GO:0016020,GO:0016021		
FPS10_10485	SDR family oxidoreductase	GO:0008152,GO:0016491,GO:0055114		R
FPS10_10490	SH3 domain-containing protein			T
FPS10_10495	DNA topoisomerase IV subunit A	GO:0003677,GO:0003916,GO:0003918,GO:0005524,GO:0005694,GO:0005886,GO:0006259,GO:0006265,GO:0007059,GO:0016020,GO:0016853,GO:0019897	K02621	L
FPS10_10500	DUF2062 domain-containing protein		K09928	S
FPS10_10505	bifunctional (p)ppGpp synthetase/guanosine-3'%2C5'-bis(diphosphate) 3'-pyrophosphohydrolase	GO:0008152,GO:0008728,GO:0008893,GO:0015969,GO:0016597,GO:0016740,GO:0016787	K00951	TK
FPS10_10510	DNA-directed RNA polymerase subunit omega	GO:0003677,GO:0003899,GO:0006351,GO:0016740,GO:00167[illegible]	K03060	K
FPS10_10515	2-amino-4-hydroxy-6- hydroxymethyldihydropteridine diphosphokinase		K00950	H
FPS10_10520	NYN domain-containing protein			S
FPS10_10525	4-hydroxy-3-methylbut-2-enyl diphosphate reductase	GO:0008299,GO:0016114,GO:0016491,GO:0019288,GO:00468[illegible]2,GO:0050992,GO:0051536,GO:0051538,GO:0051745,GO:0055114	K03527	IM
FPS10_10530	LysE family translocator	GO:0005886,GO:0006865,GO:0016020,GO:0016021		E
FPS10_10535	class I SAM-dependent methyltransferase	GO:0008152,GO:0008168,GO:0016740,GO:0032259		H
FPS10_10540	ribonuclease HI		K03469	L
FPS10_10545	methionyl-tRNA formyltransferase	GO:0003824,GO:0004479,GO:0006412,GO:0006413,GO:0009058,GO:0016740,GO:0016742,GO:0071951	K00604	J
FPS10_10550	peptide deformylase	GO:0005506,GO:0006412,GO:0016787,GO:0042586,GO:00468[illegible]	K01462	J
FPS10_10555	peptide deformylase	GO:0005506,GO:0006412,GO:0016787,GO:0042586,GO:00468[illegible]	K01462	J
FPS10_10560	peptide deformylase	GO:0005506,GO:0006412,GO:0016787,GO:0042586,GO:00468[illegible]	K01462	J
FPS10_10565	pyridoxal phosphate-dependent aminotransferase		K14155	E
FPS10_10570	L%2CD-transpeptidase family protein	GO:0008152,GO:0016740		S
FPS10_10575	hypothetical protein			
FPS10_10580	glutathione S-transferase family protein		K07393	O
FPS10_10585	response regulator			T
FPS10_10590	threonine-phosphate decarboxylase	GO:0003824,GO:0008483,GO:0009058,GO:0009236,GO:0016740,GO:0030170	K02225	E
FPS10_10595	cobalamin biosynthesis protein	GO:0005886,GO:0009236,GO:0015420,GO:0015889,GO:0016020,GO:0016021,GO:0016874,GO:0035461,GO:0048472	K02227	H
FPS10_10600	carbamoyl-phosphate synthase large subunit		K01955	F
FPS10_10605	hypothetical protein			
FPS10_10610	FAD-binding protein	GO:0006744,GO:0016491,GO:0016709,GO:0050660,GO:0055114,GO:0071949	K03185	HC
FPS10_10615	alanine--glyoxylate aminotransferase family protein	GO:0003824,GO:0008152,GO:0008483,GO:0016740		E
FPS10_10620	GGDEF domain-containing protein			T
FPS10_10625	Lrp/AsnC family transcriptional regulator	GO:0003677,GO:0003700,GO:0005622,GO:0006351,GO:0006355,GO:0043565		K
FPS10_10630	Lrp/AsnC family transcriptional regulator	GO:0003677,GO:0003700,GO:0005622,GO:0006351,GO:0006355,GO:0043565	K05800	K
FPS10_10635	ketol-acid reductoisomerase	GO:0004455,GO:0008652,GO:0009082,GO:0009097,GO:0009099,GO:0016491,GO:0055114	K00053	EH
FPS10_10640	DMT family transporter	GO:0016020,GO:0016021		GER
FPS10_10645	phosphoglucosamine mutase	GO:0000287,GO:0005975,GO:0008966,GO:0016853,GO:0016868,GO:0046872,GO:0071704	K03431	G
FPS10_10650	dihydropteroate synthase	GO:0004156,GO:0009396,GO:0016740,GO:0042558,GO:0044237,GO:0046654,GO:0046656,GO:0046872	K00796	H
FPS10_10655	dihydroneopterin aldolase	GO:0004150,GO:0006760	K01633	H
FPS10_10660	cell wall hydrolase	GO:0008152,GO:0016787		M
FPS10_10665	pyruvate%2C phosphate dikinase	GO:0003824,GO:0005524,GO:0006090,GO:0016301,GO:0016310,GO:0016740,GO:0016772,GO:0046872,GO:0050242	K01006	G
FPS10_10670	glycine--tRNA ligase subunit beta	GO:0000166,GO:0004812,GO:0004814,GO:0004820,GO:0005524,GO:0005737,GO:0006412,GO:0006418,GO:0006420,GO:0006426,GO:0016874	K01879	J
FPS10_10675	sodium/glutamate symporter	GO:0015501,GO:0015813,GO:0016020,GO:0016021,GO:00897[illegible]1	K03312	E
FPS10_10680	histidine kinase	GO:0016020,GO:0016021		
FPS10_10685	glycine--tRNA ligase subunit alpha	GO:0000166,GO:0004812,GO:0004820,GO:0005524,GO:0005737,GO:0006412,GO:0006426,GO:0016874	K01878	J
FPS10_10690	peptidoglycan-binding protein			O
FPS10_10695	TrkH family potassium uptake protein	GO:0005886,GO:0006810,GO:0006811,GO:0006812,GO:0006813,GO:0008324,GO:0016020,GO:0016021,GO:0022820,GO:0055085,GO:0071805	K03498	P
FPS10_10700	LuxR family transcriptional regulator			TK
FPS10_10705	GTP cyclohydrolase I FolE2	GO:0003933,GO:0003934,GO:0016787,GO:0035998	K09007	S

FPS10_10710	glutamine amidotransferase	GO:0003922,GO:0006541,GO:0016740,GO:0016874	K01951	F
FPS10_10715	helix-turn-helix transcriptional regulator			K
FPS10_10720	DUF2061 domain-containing protein	GO:0016020,GO:0016021		S
FPS10_10725	O-succinylhomoserine sulfhydrylase	GO:0003824,GO:0030170,GO:0071268	K10764	E
FPS10_10730	glutathione S-transferase family protein		K11209	O
FPS10_10735	dimethlysulfonioproprionate lyase DddL	GO:0008152,GO:0047869	K16953	T
FPS10_10740	septation protein IspZ	GO:0000917,GO:0005886,GO:0005887,GO:0007049,GO:0016020,GO:0016021,GO:0051301	K06190	D
FPS10_10745	EamA family transporter	GO:0016020,GO:0016021		GER
FPS10_10750	signal recognition particle-docking protein FtsY	GO:0000166,GO:0003924,GO:0005525,GO:0005737,GO:0005886,GO:0006612,GO:0006614,GO:0008152,GO:0016020,GO:0031226	K03110	U
FPS10_10755	lysoplasmalogenase			S
FPS10_10760	hypothetical protein			
FPS10_10765	exodeoxyribonuclease VII large subunit		K03601	L
FPS10_10770	methyltransferase domain-containing protein	GO:0008152,GO:0008168,GO:0016740,GO:0032259		H
FPS10_10775	phosphoribosylamine--glycine ligase	GO:0000166,GO:0000287,GO:0003824,GO:0004637,GO:0005524,GO:0006164,GO:0006189,GO:0009113,GO:0016874,GO:0030145,GO:0046872	K01945	F
FPS10_10780	DMT family transporter	GO:0016020,GO:0016021		GER
FPS10_10785	nuclear transport factor 2 family protein			S
FPS10_10790	acetylornithine deacetylase/succinyl-diaminopimelate desuccinylase family protein	GO:0006508,GO:0008152,GO:0008237,GO:0016787,GO:0016813	K01439	E
FPS10_10795	hypothetical protein	GO:0016020,GO:0016021		
FPS10_10800	DUF1523 family protein	GO:0016020,GO:0016021		
FPS10_10805	ribulose 1%2C5-bisphosphate carboxylase		K01601	G
FPS10_10810	four-carbon acid sugar kinase family protein		K23247	S
FPS10_10815	semialdehyde dehydrogenase		K23245	CHR
FPS10_10820	DEAD/DEAH box helicase	GO:0000166,GO:0003676,GO:0004386,GO:0005524,GO:0008152,GO:0016787	K11927	LKJ
FPS10_10825	transcriptional regulator NanR			K
FPS10_10830	hydratase	GO:0003824,GO:0008152		Q
FPS10_10835	dienelactone hydrolase family protein	GO:0008152,GO:0016787	K01061	Q
FPS10_10840	TAXI family TRAP transporter solute-binding subunit		K07080	R
FPS10_10845	TRAP transporter fused permease subunit			R
FPS10_10850	amidohydrolase family protein		K10221	R
FPS10_10855	LysR family transcriptional regulator			K
FPS10_10860	4-oxalomesaconate tautomerase		K16514	S
FPS10_10865	4-carboxy-4-hydroxy-2-oxoadipate aldolase/oxaloacetate decarboxylase		K10218	H
FPS10_10870	PIG-L family deacetylase		K16515	S
FPS10_10875	LysR family transcriptional regulator	GO:0003677,GO:0003700,GO:0006351,GO:0006355		K
FPS10_10880	NAD(P)-dependent oxidoreductase			I
FPS10_10885	hypothetical protein		K11900	S
FPS10_10890	hypothetical protein			
FPS10_10895	aldehyde dehydrogenase		K07248	C
FPS10_10900	mandelate racemase/muconate lactonizing enzyme family protein	GO:0003824,GO:0008152,GO:0046872		MR
FPS10_10905	ABC transporter permease		K23509	G
FPS10_10910	ABC transporter permease		K23509	G
FPS10_10915	sugar ABC transporter ATP-binding protein		K10820	G
FPS10_10920	ABC transporter substrate-binding protein		K23508	G
FPS10_10925	FCD domain-containing protein	GO:0003677,GO:0003700,GO:0006351,GO:0006355	K22104	K
FPS10_10930	FAD-binding protein			E
FPS10_10935	SDR family oxidoreductase			IQR
FPS10_10945				
FPS10_10950	HAMP domain-containing histidine kinase			T
FPS10_10955	dihydrolipoamide dehydrogenase	GO:0005623,GO:0016491,GO:0016668,GO:0045454,GO:0050660,GO:0055114		C
FPS10_10960	TVP38/TMEM64 family protein	GO:0016020,GO:0016021		S
FPS10_10965	50S ribosomal protein L34	GO:0003735,GO:0005622,GO:0005840,GO:0006412,GO:0030529	K02914	J
FPS10_10970	ribonuclease P protein component		K03536	J
FPS10_10975	membrane protein insertion efficiency factor YidD		K08998	S
FPS10_10980	tRNA 2-thiocytidine(32) synthetase TtcA	GO:0000166,GO:0005524,GO:0005737,GO:0008033	K14058	D
FPS10_10985	GGDEF domain-containing protein	GO:0016020,GO:0016021		T
FPS10_10990	membrane protein insertase YidC	GO:0005886,GO:0006810,GO:0015031,GO:0016020,GO:0016021,GO:0051205	K03217	U
FPS10_10995	MOSC domain-containing protein	GO:0003824,GO:0008152,GO:0030151,GO:0030170	K07140	R

FPS10_11000	YihA family ribosome biogenesis GTP-binding protein	GO:0000166,GO:0000287,GO:0000917,GO:0003924,GO:0005525,GO:0007049,GO:0008152,GO:0046872,GO:0051301	K03978	R
FPS10_11005	acetylglutamate kinase	GO:0000166,GO:0003991,GO:0005524,GO:0005737,GO:000652[illegible]6,GO:0006561,GO:0008652,GO:0016301,GO:0016310,GO:0016740	K00930	E
FPS10_11010	ferredoxin			
FPS10_11015	histidine phosphatase family protein		K08296	T
FPS10_11020	amino acid ABC transporter ATP-binding protein	GO:0000166,GO:0003333,GO:0005524,GO:0008152,GO:0015424,GO:0015426,GO:0016787,GO:0016887	K09972	E
FPS10_11025	amino acid ABC transporter permease		K09971	E
FPS10_11030	ABC transporter permease subunit		K09970	E
FPS10_11035	amino acid ABC transporter substrate-binding protein		K09969	ET
FPS10_11040	ATPase	GO:0043461		O
FPS10_11045	HAD-IA family hydrolase	GO:0008152,GO:0016787	K01091	R
FPS10_11050	RluA family pseudouridine synthase	GO:0001522,GO:0003723,GO:0009451,GO:0009982,GO:00168[illegible]3	K06179	J
FPS10_11055	fluoride efflux transporter CrcB	GO:0005886,GO:0005887,GO:0006810,GO:0015103,GO:0015698,GO:0016020,GO:0016021,GO:0034220	K06199	D
FPS10_11060	cytochrome B			C
FPS10_11065	cytochrome c			C
FPS10_11070	replication-associated recombination protein A	GO:0003677,GO:0006260,GO:0006281,GO:0006310,GO:0009378,GO:0032508	K07478	L
FPS10_11075	LuxR family transcriptional regulator	GO:0003677,GO:0006355	K07782	TK
FPS10_11080	50S ribosomal protein L17	GO:0003735,GO:0005622,GO:0005840,GO:0006412,GO:00305[illegible]9	K02879	J
FPS10_11085	DNA-directed RNA polymerase subunit alpha	GO:0003677,GO:0003899,GO:0006351,GO:0016740,GO:0016779,GO:0046983	K03040	K
FPS10_11090	30S ribosomal protein S11		K02948	J
FPS10_11095	30S ribosomal protein S13	GO:0000049,GO:0003676,GO:0003723,GO:0003735,GO:0005622,GO:0005840,GO:0006412,GO:0019843,GO:0030529	K02952	J
FPS10_11100	JAB domain-containing protein		K03630	L
FPS10_11105	hypothetical protein		K09857	
FPS10_11110	MCE family protein		K06192	R
FPS10_11115	paraquat-inducible protein A	GO:0016020,GO:0016021	K03808	S
FPS10_11120	paraquat-inducible protein A		K03808	S
FPS10_11125	cytochrome c			
FPS10_11130	ATP-binding cassette domain-containing protein	GO:0000166,GO:0005524,GO:0006810,GO:0008152,GO:0015833,GO:0016887	K10823	E
FPS10_11135	oligopeptide ABC transporter ATP-binding protein OppD	GO:0000166,GO:0005524,GO:0006810,GO:0008152,GO:0015833,GO:0016887	K15583	EP
FPS10_11140	ABC transporter permease subunit		K15582	EP
FPS10_11145	oligopeptide ABC transporter permease OppB	GO:0005886,GO:0006810,GO:0016020,GO:0016021	K15581	EP
FPS10_11150	peptide ABC transporter substrate-binding protein	GO:0043190,GO:0055085	K15580	E
FPS10_11155	beta-lactamase family protein			V
FPS10_11160	phosphomannomutase/phosphoglucomutase	GO:0000287,GO:0004615,GO:0005975,GO:0016853,GO:0016868,GO:0046872,GO:0071704	K01840	G
FPS10_11165	NnrU family protein	GO:0016020,GO:0016021		S
FPS10_11170	hypothetical protein			
FPS10_11175	calcium-binding protein			Q
FPS10_11180	3-keto-5-aminohexanoate cleavage protein	GO:0003824,GO:0016740,GO:0019475		S
FPS10_11185	ETC complex I subunit	GO:0008137,GO:0016491,GO:0016651,GO:0022900,GO:00551[illegible]4		
FPS10_11190	excinuclease ABC subunit UvrB	GO:0000166,GO:0003677,GO:0004386,GO:0004518,GO:0005524,GO:0005737,GO:0006281,GO:0006289,GO:0006974,GO:0009381,GO:0009432,GO:0016787,GO:0090305	K03702	L
FPS10_11195	hypothetical protein		K01142	L
FPS10_11200	hypothetical protein			
FPS10_11205	hypothetical protein			
FPS10_11210	YdcF family protein			S
FPS10_11215	universal stress protein	GO:0006950		T
FPS10_11220	NAD(P)H-dependent oxidoreductase		K00355	R
FPS10_11225	antibiotic biosynthesis monooxygenase	GO:0004497,GO:0055114		
FPS10_11230	universal stress protein	GO:0006950		T
FPS10_11235	HugZ family protein	GO:0010181,GO:0016491,GO:0055114	K07226	P
FPS10_11240	hypothetical protein			
FPS10_11245	PLP-dependent transferase		K01761	E
FPS10_11250	DNA polymerase III subunit alpha	GO:0003677,GO:0003824,GO:0003887,GO:0006260,GO:0008408,GO:0016740,GO:0016779,GO:0071897,GO:0090305	K02337	L
FPS10_11255	sulfite exporter TauE/SafE family protein		K07090	R
FPS10_11260	xanthine dehydrogenase small subunit	GO:0003824,GO:0009055,GO:0016491,GO:0016614,GO:0046872,GO:0050660,GO:0051536,GO:0051537,GO:0055114	K13481	F
FPS10_11265	xanthine dehydrogenase molybdopterin binding subunit		K13482	F

FPS10_11270	xanthine dehydrogenase accessory protein XdhC		K07402	O
FPS10_11275	ABC transporter ATP-binding protein	GO:0000166,GO:0005524,GO:0008152,GO:0016887	K23537	R
FPS10_11280	ABC transporter permease	GO:0005215,GO:0005886,GO:0006810,GO:0016020,GO:0016021	K23535	R
FPS10_11285	ABC transporter permease	GO:0005215,GO:0005886,GO:0006810,GO:0016020,GO:0016021	K23536	R
FPS10_11290	BMP family ABC transporter substrate-binding protein	GO:0005886	K07335	R
FPS10_11295	hypothetical protein			
FPS10_11300	hypothetical protein	GO:0016020,GO:0016021		
FPS10_11305	DeoR/GlpR transcriptional regulator		K02444	KG
FPS10_11310	TRAP transporter small permease			G
FPS10_11315	TRAP transporter large permease	GO:0016020,GO:0016021		G
FPS10_11320	C4-dicarboxylate ABC transporter substrate-binding protein			G
FPS10_11325	sel1 repeat family protein		K07126	R
FPS10_11330	methylamine utilization protein MauG		K00428	P
FPS10_11335	sugar kinase	GO:0004747,GO:0006014,GO:0016301,GO:0016310,GO:0016740,GO:0016773,GO:0046835	K18478	G
FPS10_11340	DUF2090 domain-containing protein	GO:0003824,GO:0008152,GO:0009025,GO:0016829	K01635	G
FPS10_11345	hypothetical protein	GO:0005737,GO:0005975,GO:0005996,GO:0006004,GO:0008733,GO:0008736,GO:0016853,GO:0016861,GO:0019568,GO:0019569,GO:0046872		G
FPS10_11350	Gfo/Idh/MocA family oxidoreductase	GO:0008152,GO:0016491,GO:0055114		R
FPS10_11355	galactose mutarotase		K01785	G
FPS10_11360	NAD-dependent succinate-semialdehyde dehydrogenase		K00135	C
FPS10_11365	hydroxyacid dehydrogenase	GO:0008152,GO:0016491,GO:0016616,GO:0051287,GO:0055114	K16843	HE
FPS10_11370	sulfopyruvate decarboxylase subunit alpha		K06034	R
FPS10_11375	sulfopyruvate decarboxylase subunit beta	GO:0003824,GO:0008152,GO:0016829,GO:0030976,GO:0050545	K13039	EH
FPS10_11380	hypothetical protein			
FPS10_11385	MarR family transcriptional regulator			K
FPS10_11390	MBL fold metallo-hydrolase			R
FPS10_11395	FAD-dependent oxidoreductase	GO:0016491,GO:0055114,GO:0071949	K05712	HC
FPS10_11400	DUF2783 domain-containing protein			
FPS10_11405	S8 family serine peptidase			O
FPS10_11410	hypothetical protein			
FPS10_11415	hypothetical protein			
FPS10_11420	hypothetical protein			
FPS10_11425	HlyD family type I secretion periplasmic adaptor subunit			V
FPS10_11430	ATP-binding cassette domain-containing protein			R
FPS10_11435	hypothetical protein			
FPS10_11440	hypothetical protein			
FPS10_11445	hypothetical protein			
FPS10_11450	hypothetical protein			
FPS10_11455	DUF1611 domain-containing protein			S
FPS10_11460	alanine/ornithine racemase family PLP-dependent enzyme		K21898	E
FPS10_11470	hypothetical protein			
FPS10_11475	helix-turn-helix transcriptional regulator			K
FPS10_11480	alpha/beta hydrolase	GO:0003824,GO:0008152,GO:0016787		R
FPS10_11495	IS110 family transposase			L
FPS10_11500	hypothetical protein			
FPS10_11505	IS5 family transposase			L
FPS10_11510	DUF1214 domain-containing protein			S
FPS10_11515	DUF1254 domain-containing protein			S
FPS10_11520	DUF1254 domain-containing protein			S
FPS10_11525	DUF1214 domain-containing protein			S
FPS10_11530	DUF1214 domain-containing protein			S
FPS10_11535	RcnB family protein			
FPS10_11540	DUF1214 domain-containing protein			S
FPS10_11545	hypothetical protein			
FPS10_11550	hypothetical protein			
FPS10_11555	hypothetical protein			
FPS10_11560	hypothetical protein			
FPS10_11565	DUF3604 domain-containing protein	GO:0003824,GO:0008152		
FPS10_11570	hypothetical protein			
FPS10_11580	IS630 family transposase			L
FPS10_11590	TetR family transcriptional regulator	GO:0003677,GO:0006351,GO:0006355		K
FPS10_11595	glycosyltransferase			M
FPS10_11600	glycosyltransferase family 4 protein			M
FPS10_11605	NAD(P)/FAD-dependent oxidoreductase			R

FPS10_11610	mannose-1-phosphate guanylyltransferase/mannose-6-phosphate isomerase	GO:0000271,GO:0004476,GO:0005976,GO:0008928,GO:0009058,GO:0016740,GO:0016779,GO:0016853	K16011	G
FPS10_11615	hypothetical protein			
FPS10_11620	glycosyltransferase family 2 protein	GO:0008152,GO:0016020,GO:0016021,GO:0016740		R
FPS10_11625	glycosyltransferase family 2 protein			R
FPS10_11635	WecB/TagA/CpsF family glycosyltransferase			M
FPS10_11640	tyrosine-protein kinase family protein			D
FPS10_11645	lipopolysaccharide biosynthesis protein			M
FPS10_11650	AAA family ATPase			U
FPS10_11655	tetratricopeptide repeat protein			R
FPS10_11660	glycosyltransferase family 2 protein			
FPS10_11665	sulfotransferase domain-containing protein			
FPS10_11670	oligosaccharide flippase family protein			R
FPS10_11675	VPLPA-CTERM-specific exosortase XrtD	GO:0016020,GO:0016021		
FPS10_11680	DNA polymerase III subunit gamma/tau	GO:0003677,GO:0003887,GO:0005524,GO:0006260,GO:0009360,GO:0071897	K02343	L
FPS10_11685	hypothetical protein			R
FPS10_11690	ribosome small subunit-dependent GTPase A	GO:0000166,GO:0003924,GO:0005525,GO:0008152,GO:0016787,GO:0046872	K06949	R
FPS10_11695				
FPS10_11700	cell wall hydrolase			M
FPS10_11705	ribose 5-phosphate isomerase B	GO:0004751,GO:0005975,GO:0006098,GO:0016853	K01808	G
FPS10_11710	glycosyl transferase		K13693	
FPS10_11715	mannosyl-3-phosphoglycerate phosphatase		K07026	R
FPS10_11720	ABC transporter permease	GO:0005886,GO:0006810,GO:0016020,GO:0016021	K02050	P
FPS10_11725	ABC transporter ATP-binding protein		K02049	P
FPS10_11730	ABC transporter substrate-binding protein		K02051	P
FPS10_11735	DMT family transporter	GO:0016020,GO:0016021		GER
FPS10_11740	PLP-dependent aminotransferase family protein	GO:0003677,GO:0003700,GO:0003824,GO:0006351,GO:0006355,GO:0009058,GO:0030170	K00375	KE
FPS10_11745	pyridoxamine 5'-phosphate oxidase family protein	GO:0010181,GO:0016491,GO:0055114	K07005	R
FPS10_11750	SRPBCC family protein			
FPS10_11755	NAD(+) diphosphatase		K03426	L
FPS10_11760	bifunctional 2'%2C3'-cyclic-nucleotide 2'-phosphodiesterase/3'-nucleotidase	GO:0000166,GO:0009166,GO:0016787,GO:0016788,GO:0046872	K01119	F
FPS10_11765	nuclear transport factor 2 family protein			
FPS10_11770	prephenate dehydratase	GO:0004664,GO:0008152,GO:0009094,GO:0016597,GO:00168[illegible]	K04518	E
FPS10_11775	c-type cytochrome		K08738	C
FPS10_11780	hypothetical protein			
FPS10_11785	hypothetical protein			
FPS10_11790	ABC transporter substrate-binding protein		K13893	E
FPS10_11795	ABC transporter permease subunit	GO:0005886,GO:0006810,GO:0016020,GO:0016021	K13894	R
FPS10_11800	ABC transporter permease		K13895	R
FPS10_11805	ABC transporter ATP-binding protein	GO:0000166,GO:0005524,GO:0006810,GO:0008152,GO:0015833,GO:0016887	K13896	R
FPS10_11810	acyl-CoA thioesterase		K10806	I
FPS10_11815	oxygen-independent coproporphyrinogen III oxidase	GO:0003824,GO:0004109,GO:0005737,GO:0006779,GO:0006782,GO:0016491,GO:0046872,GO:0051536,GO:0051539,GO:0051989,GO:0055114	K02495	H
FPS10_11820	Crp/Fnr family transcriptional regulator	GO:0003677,GO:0003700,GO:0005622,GO:0006351,GO:00063[illegible]	K01420	T
FPS10_11825	universal stress protein	GO:0006950		T
FPS10_11830	cytochrome-c oxidase%2C cbb3-type subunit I	GO:0004129,GO:0005506,GO:0009055,GO:0009060,GO:0016020,GO:0016021,GO:0016491,GO:0020037,GO:0045278,GO:0046872,GO:0055114,GO:0070469,GO:1902600	K00404	O
FPS10_11835	cytochrome-c oxidase%2C cbb3-type subunit II	GO:0004129,GO:0005506,GO:0009055,GO:0009060,GO:0016020,GO:0016021,GO:0020037,GO:0055114,GO:1902600	K00405	C
FPS10_11840	CcoQ/FixQ family Cbb3-type cytochrome c oxidase assembly chaperone		K00407	O
FPS10_11845	cytochrome-c oxidase%2C cbb3-type subunit III	GO:0005506,GO:0005886,GO:0006119,GO:0006811,GO:0009055,GO:0015992,GO:0016020,GO:0016021,GO:0016491,GO:0020037,GO:0046872,GO:0055114,GO:0070469	K00406	C
FPS10_11850	cytochrome c oxidase accessory protein CcoG			C
FPS10_11855	FixH family protein			P
FPS10_11860	cadmium-translocating P-type ATPase	GO:0000166,GO:0005886,GO:0006812,GO:0008152,GO:0016020,GO:0016021,GO:0016787,GO:0019829,GO:0030001,GO:0046872,GO:0098655	K01533	P
FPS10_11865	cbb3-type cytochrome oxidase assembly protein CcoS	GO:0016020,GO:0016021		P
FPS10_11870	D-alanyl-D-alanine carboxypeptidase	GO:0004180,GO:0006508,GO:0009002	K01286	M
FPS10_11875	HAD family hydrolase	GO:0008152,GO:0016787	K07025	R

FPS10_11880	ATP-dependent Clp protease adapter ClpS	GO:0006508,GO:0008233,GO:0030163	K06891	S
FPS10_11885	endonuclease/exonuclease/phosphatase family protein	GO:0004519,GO:0004527,GO:0090305		R
FPS10_11890	DMT family transporter	GO:0016020,GO:0016021		GER
FPS10_11895	30S ribosomal protein S12		K02950	J
FPS10_11900	30S ribosomal protein S7	GO:0000049,GO:0003723,GO:0003735,GO:0005840,GO:0006412,GO:0015935,GO:0019843,GO:0030529	K02992	J
FPS10_11905	elongation factor G	GO:0000166,GO:0003746,GO:0003924,GO:0005525,GO:0005622,GO:0005737,GO:0006412,GO:0006414	K02355	J
FPS10_11910	site-specific DNA-methyltransferase	GO:0003676,GO:0003677,GO:0006306,GO:0008168,GO:0008170,GO:0032259		L
FPS10_11915	hypothetical protein		K06952	
FPS10_11920	hypothetical protein			
FPS10_11925	elements of external origin			
FPS10_11930	phage terminase large subunit family protein			R
FPS10_11935	hypothetical protein			
FPS10_11940	phage portal protein	GO:0005198,GO:0019068		R
FPS10_11945	peptidase U35			
FPS10_11950	DUF2190 family protein			S
FPS10_11955	hypothetical protein			
FPS10_11960	hypothetical protein			
FPS10_11965	acyl-CoA transferase			
FPS10_11970	hypothetical protein			
FPS10_11975	hypothetical protein			
FPS10_11980	hypothetical protein			
FPS10_11985	hypothetical protein			S
FPS10_11990	TIGR02217 family protein			S
FPS10_11995	hypothetical protein	GO:0016020,GO:0016021		
FPS10_12000	lysozyme	GO:0003796,GO:0003824,GO:0008152,GO:0009253,GO:0016787,GO:0016798,GO:0016998,GO:0019835,GO:0042742	K01185	R
FPS10_12005	hypothetical protein			
FPS10_12010	DUF2163 domain-containing protein			S
FPS10_12015	peptidase			M
FPS10_12020	hypothetical protein			
FPS10_12025	DUF2793 domain-containing protein			
FPS10_12030	DNA cytosine methyltransferase		K00558	L
FPS10_12035	hypothetical protein			
FPS10_12040	hypothetical protein			
FPS10_12045	sialate O-acetylesterase			
FPS10_12050	amidohydrolase family protein			Q
FPS10_12055	AraC family transcriptional regulator			K
FPS10_12060				
FPS10_12065	hypothetical protein			S
FPS10_12070	hypothetical protein			S
FPS10_12075	prolyl oligopeptidase family serine peptidase			Q
FPS10_12080	tryptophan-rich sensory protein		K05770	T
FPS10_12085	peroxiredoxin			O
FPS10_12090	IclR family transcriptional regulator	GO:0003677,GO:0006351,GO:0006355	K13641	K
FPS10_12095	malate synthase G	GO:0003824,GO:0004474,GO:0005737,GO:0006097,GO:0006099,GO:0016740,GO:0016746,GO:0046872	K01638	C
FPS10_12100	heme-binding protein			R
FPS10_12105	D-glycerate dehydrogenase		K00015	CHR
FPS10_12110	hypothetical protein			
FPS10_12115	amidase	GO:0008152,GO:0016884		J
FPS10_12120	TRAP transporter large permease subunit	GO:0016020,GO:0016021		Q
FPS10_12125	TRAP transporter small permease subunit			Q
FPS10_12130	TRAP transporter substrate-binding protein			Q
FPS10_12135	IclR family transcriptional regulator	GO:0003677,GO:0006351,GO:0006355	K13641	K
FPS10_12140	LysR family transcriptional regulator			K
FPS10_12145	DUF2218 domain-containing protein		K09956	S
FPS10_12150	MBL fold metallo-hydrolase			R
FPS10_12155	iron reductase		K17247	S
FPS10_12160	SDR family oxidoreductase		K19267	MG
FPS10_12165	hypothetical protein			
FPS10_12170	hypothetical protein			
FPS10_12175	transposase		K07483	
FPS10_12180	ATP-binding protein	GO:0005524		L
FPS10_12185	transposase			

FPS10_12190	helix-turn-helix domain-containing protein			
FPS10_12195	hypothetical protein			
FPS10_12200	tyrosine-type recombinase/integrase			L
FPS10_12205				
FPS10_12210	30S ribosomal protein S4		K02986	J
FPS10_12215	histidinol-phosphate transaminase	GO:0000105,GO:0003824,GO:0004400,GO:0008483,GO:0008652,GO:0009058,GO:0016740,GO:0030170,GO:0080130	K00817	E
FPS10_12220	prephenate/arogenate dehydrogenase family protein	GO:0004665,GO:0006571,GO:0008977,GO:0055114	K00220	E
FPS10_12225	extensin family protein			S
FPS10_12230	GNAT family N-acetyltransferase			R
FPS10_12235	GrpB family protein			S
FPS10_12240	nucleoside hydrolase	GO:0008152,GO:0008477,GO:0016787,GO:0016798,GO:0016799	K01239	F
FPS10_12245	OsmC family protein	GO:0006979	K06889	R
FPS10_12255	hypothetical protein			
FPS10_12260	Lrp/AsnC family transcriptional regulator			K
FPS10_12265	ornithine cyclodeaminase	GO:0008152,GO:0008473,GO:0016829		E
FPS10_12270	amidinotransferase			S
FPS10_12275	PEP-CTERM sorting domain-containing protein			
FPS10_12280	type I glutamate--ammonia ligase	GO:0000166,GO:0003824,GO:0004356,GO:0005524,GO:0005737,GO:0006542,GO:0006807,GO:0009399,GO:0016874	K01915	E
FPS10_12285	P-II family nitrogen regulator	GO:0006351,GO:0006355,GO:0006808,GO:0030234,GO:0050790	K04751	E
FPS10_12290	bifunctional ADP-dependent NAD(P)H-hydrate dehydratase/NAD(P)H-hydrate epimerase	GO:0008152,GO:0052855		S
FPS10_12295	DUF4167 domain-containing protein			
FPS10_12300	peptide chain release factor N(5)-glutamine methyltransferase	GO:0003676,GO:0006479,GO:0008168,GO:0008276,GO:0016740,GO:0018364,GO:0032259,GO:0036009	K02493	J
FPS10_12305	peptide chain release factor 1	GO:0003747,GO:0005737,GO:0006412,GO:0006415,GO:0016149	K02835	J
FPS10_12310	hypothetical protein			
FPS10_12315	SLC13 family permease	GO:0006813,GO:0008324,GO:0016020,GO:0016021,GO:0055085,GO:0098655		P
FPS10_12320	amidohydrolase			R
FPS10_12325	nucleoside triphosphate pyrophosphohydrolase	GO:0008152,GO:0016787	K04765	R
FPS10_12330	hypothetical protein			
FPS10_12335	DUF2125 domain-containing protein			S
FPS10_12340	gamma-glutamylcyclotransferase		K07232	P
FPS10_12350	peptidoglycan -binding protein	GO:0016020,GO:0016021	K02557	D
FPS10_12355	M23 family metallopeptidase			M
FPS10_12360	ATP-dependent Clp protease ATP-binding subunit ClpA	GO:0000166,GO:0004176,GO:0005524,GO:0006508,GO:0019538	K03694	O
FPS10_12365	hydroxyacylglutathione hydrolase	GO:0004416,GO:0016787,GO:0019243,GO:0046872	K01069	R
FPS10_12370	class I SAM-dependent methyltransferase			H
FPS10_12375	F0F1 ATP synthase subunit delta	GO:0005886,GO:0006754,GO:0006810,GO:0006811,GO:0015986,GO:0015992,GO:0016020,GO:0042777,GO:0045261,GO:0046933	K02113	C
FPS10_12380	F0F1 ATP synthase subunit alpha	GO:0000166,GO:0005524,GO:0005886,GO:0006754,GO:0006810,GO:0006811,GO:0015986,GO:0015991,GO:0015992,GO:0016020,GO:0016787,GO:0016820,GO:0033178,GO:0042777,GO:0045261,GO:0046034,GO:0046933,GO:0046961	K02111	C
FPS10_12385	F0F1 ATP synthase subunit gamma	GO:0005524,GO:0005886,GO:0006754,GO:0006810,GO:0006811,GO:0015986,GO:0015992,GO:0016020,GO:0016787,GO:0042777,GO:0045261,GO:0046933,GO:0046961	K02115	C
FPS10_12390	F0F1 ATP synthase subunit beta	GO:0000166,GO:0005524,GO:0005886,GO:0006754,GO:0006810,GO:0006811,GO:0015986,GO:0015991,GO:0015992,GO:0016020,GO:0016787,GO:0016820,GO:0033178,GO:0042777,GO:0045261,GO:0046034,GO:0046933	K02112	C
FPS10_12395	F0F1 ATP synthase subunit epsilon		K02114	C
FPS10_12400	tol-pal system-associated acyl-CoA thioesterase	GO:0008152,GO:0016787,GO:0016790	K07107	R
FPS10_12405	hypothetical protein			L
FPS10_12410	aminomethyl-transferring glycine dehydrogenase	GO:0003824,GO:0004375,GO:0006544,GO:0006546,GO:0016491,GO:0019464,GO:0055114	K00281	E
FPS10_12415	glycine cleavage system protein GcvH	GO:0005960,GO:0019464	K02437	E
FPS10_12420	glycine cleavage system aminomethyltransferase GcvT	GO:0004047,GO:0006546,GO:0008483,GO:0016740,GO:0032259	K00605	E
FPS10_12425	gamma-glutamylcyclotransferase			P
FPS10_12430	MATE family efflux transporter	GO:0006855,GO:0015238,GO:0015297,GO:0016020,GO:0016021,GO:0055085		V
FPS10_12435	hypothetical protein			
FPS10_12440	ACR3 family arsenite efflux transporter		K03325	P
FPS10_12445	organoarsenical effux MFS transporter ArsJ			
FPS10_12450	ArsJ-associated glyceraldehyde-3-phosphate dehydrogenase	GO:0006006,GO:0016491,GO:0016620,GO:0050661,GO:0051287,GO:0055114	K00134	G
FPS10_12455	metalloregulator ArsR/SmtB family transcription factor	GO:0003677,GO:0003700,GO:0006351,GO:0006355	K03741	T

FPS10_12460	N-acyl homoserine lactonase family protein			R
FPS10_12465	Gfo/Idh/MocA family oxidoreductase		K23244	R
FPS10_12470	GntR family transcriptional regulator	GO:0003677,GO:0003700,GO:0006351,GO:0006355		K
FPS10_12475	ureidoglycolate lyase		K01483	F
FPS10_12480	allantoinase PuuE			G
FPS10_12485	hydroxyisourate hydrolase	GO:0006144,GO:0016491,GO:0016787,GO:0033971,GO:00551[illegible]4	K07127	R
FPS10_12490	hypothetical protein		K09800	S
FPS10_12495	outer membrane protein assembly factor		K07278	M
FPS10_12500	ParA family protein		K03496	D
FPS10_12505	YegS/Rv2252/BmrU family lipid kinase			IR
FPS10_12510	L%2CD-transpeptidase	GO:0008152,GO:0016740		S
FPS10_12515	carboxymuconolactone decarboxylase family protein	GO:0004601,GO:0016209,GO:0016491,GO:0051920,GO:0055114,GO:0098869		S
FPS10_12520	FAD-binding oxidoreductase	GO:0016491,GO:0055114		E
FPS10_12525	hypothetical protein			
FPS10_12530	hemolysin-type calcium-binding protein			
FPS10_12535				
FPS10_12540	hypothetical protein			
FPS10_12550				
FPS10_12555	protein TolQ	GO:0006810,GO:0008565,GO:0015031,GO:0016020,GO:0016021,GO:0051301	K03562	U
FPS10_12560	protein TolR	GO:0005215,GO:0005886,GO:0006810,GO:0015031,GO:0016020,GO:0016021	K03560	U
FPS10_12565	hypothetical protein			
FPS10_12570	pilus assembly protein	GO:0016020,GO:0016021		
FPS10_12575	hypothetical protein			
FPS10_12580	Flp pilus assembly protein CpaB		K02279	U
FPS10_12585	type II and III secretion system protein family protein	GO:0005623,GO:0009306	K02280	U
FPS10_12590	hypothetical protein			
FPS10_12595	AAA family ATPase		K02282	U
FPS10_12600	CpaF family protein	GO:0005524,GO:0006810	K02283	U
FPS10_12605	pilus assembly protein TadB	GO:0016020,GO:0016021	K12510	U
FPS10_12610	type II secretion system F family protein	GO:0016020,GO:0016021	K12511	NU
FPS10_12615	glycerol-3-phosphate 1-O-acyltransferase PlsY	GO:0005886,GO:0006629,GO:0006644,GO:0008654,GO:0016020,GO:0016021,GO:0016740,GO:0043772	K08591	S
FPS10_12620	DUF805 domain-containing protein	GO:0016020,GO:0016021		S
FPS10_12625	DUF805 domain-containing protein			S
FPS10_12630	glutamate--cysteine ligase	GO:0004357,GO:0006750,GO:0016874,GO:0042398	K01919	H
FPS10_12635	hypothetical protein			
FPS10_12640	GNAT family N-acetyltransferase			R
FPS10_12645	16S rRNA (uracil(1498)-N(3))-methyltransferase	GO:0005737,GO:0006364,GO:0008168,GO:0016740,GO:00322[illegible]9	K09761	S
FPS10_12650	4-hydroxybenzoate octaprenyltransferase	GO:0004659,GO:0005886,GO:0006744,GO:0008412,GO:0016020,GO:0016021,GO:0016740	K03179	H
FPS10_12655	OmpA family protein	GO:0009279,GO:0016020,GO:0016021	K03286	M
FPS10_12660	hypothetical protein	GO:0016020,GO:0016021		
FPS10_12665	molybdenum cofactor biosynthesis protein MoaE	GO:0006777	K03635	H
FPS10_12670	molybdopterin converting factor subunit 1	GO:0000166,GO:0006777,GO:0016740	K03636	H
FPS10_12675	CDP-diacylglycerol--glycerol-3-phosphate 3-phosphatidyltransferase	GO:0006629,GO:0008444,GO:0008654,GO:0016020,GO:0016021,GO:0016740,GO:0016780	K00995	I
FPS10_12680	excinuclease ABC subunit UvrC		K03703	L
FPS10_12685	SDR family oxidoreductase	GO:0008152,GO:0016491,GO:0055114		IQR
FPS10_12690	calcium/sodium antiporter	GO:0016020,GO:0016021,GO:0055085	K07301	P
FPS10_12695	hypothetical protein			
FPS10_12700	S49 family peptidase	GO:0006508,GO:0008233,GO:0016787		OU
FPS10_12705	glycerate kinase	GO:0016491,GO:0016618,GO:0055114	K11529	G
FPS10_12710	sn-glycerol-3-phosphate ABC transporter ATP-binding protein UgpC		K10111	G
FPS10_12715	hypothetical protein			
FPS10_12720	carbohydrate ABC transporter permease	GO:0005886,GO:0006810,GO:0016020,GO:0016021	K02026	G
FPS10_12725	sugar ABC transporter permease	GO:0005886,GO:0006810,GO:0016020,GO:0016021	K02025	G
FPS10_12730	extracellular solute-binding protein		K02027	G
FPS10_12735	LacI family transcriptional regulator	GO:0003677,GO:0003700,GO:0006351,GO:0006355	K02529	K
FPS10_12740	SHOCT domain-containing protein	GO:0016020,GO:0016021		
FPS10_12745	hypothetical protein			
FPS10_12750	hypothetical protein			
FPS10_12755	tryptophan 2%2C3-dioxygenase	GO:0004833,GO:0006569,GO:0016491,GO:0019441,GO:0020037,GO:0046872,GO:0051213,GO:0055114	K00453	E

FPS10_12760	ROK family transcriptional regulator			K
FPS10_12765	nucleoside/nucleotide kinase family protein			H
FPS10_12770	tryptophan 2%2C3-dioxygenase			E
FPS10_12775	dipeptide ABC transporter ATP-binding protein		K12372	E
FPS10_12780	ABC transporter ATP-binding protein	GO:0000166,GO:0005524,GO:0006810,GO:0008152,GO:0015833,GO:0016887	K12371	EP
FPS10_12785	ABC transporter permease subunit	GO:0005886,GO:0006810,GO:0016020,GO:0016021	K12370	EP
FPS10_12790	ABC transporter permease subunit	GO:0005886,GO:0006810,GO:0016020,GO:0016021	K12369	EP
FPS10_12795	aminopeptidase P family protein		K01271	E
FPS10_12800	ABC transporter substrate-binding protein	GO:0043190,GO:0055085	K12368	E
FPS10_12805	helix-turn-helix domain-containing protein	GO:0003677,GO:0003700,GO:0006351,GO:0006355,GO:0043565		K
FPS10_12810	3-keto-5-aminohexanoate cleavage protein	GO:0003824,GO:0016740,GO:0019475		S
FPS10_12815	hypothetical protein			
FPS10_12820	carnitine 3-dehydrogenase	GO:0003857,GO:0005737,GO:0006631,GO:0009437,GO:0016491,GO:0042413,GO:0047728,GO:0051287,GO:0055114	K17735	R
FPS10_12825				
FPS10_12830	tyrosine-type recombinase/integrase			
FPS10_12835	hypothetical protein			
FPS10_12840	hypothetical protein			
FPS10_12845	hypothetical protein			
FPS10_12850	autotransporter outer membrane beta-barrel domain-containing protein			MU
FPS10_12855	TIGR03032 family protein			
FPS10_12860	hypothetical protein			
FPS10_12865	hypothetical protein			
FPS10_12870	MFS transporter	GO:0016020,GO:0016021,GO:0055085		G
FPS10_12875	aminopeptidase P family protein		K01262	E
FPS10_12880	amidase	GO:0008152,GO:0016884	K01426	J
FPS10_12885	ABC transporter ATP-binding protein		K11072	E
FPS10_12890	ABC transporter permease	GO:0005886,GO:0006810,GO:0016020,GO:0016021	K11071	E
FPS10_12895	ABC transporter permease		K11070	E
FPS10_12900	extracellular solute-binding protein	GO:0006810,GO:0015846,GO:0019808,GO:0042597	K11069	E
FPS10_12905	amidase		K01426	J
FPS10_12910	N-carbamoylputrescine amidase		K12251	R
FPS10_12915	histone deacetylase family protein			BQ
FPS10_12920	hypothetical protein			
FPS10_12930	aldehyde dehydrogenase family protein	GO:0008152,GO:0016491,GO:0016620,GO:0055114	K15515	C
FPS10_12935	Gfo/Idh/MocA family oxidoreductase	GO:0008152,GO:0016491,GO:0055114		R
FPS10_12940	GntR family transcriptional regulator	GO:0003677,GO:0003700,GO:0006351,GO:0006355		K
FPS10_12945	crotonase/enoyl-CoA hydratase family protein			I
FPS10_12950	cation transporter		K16264	P
FPS10_12955	hypothetical protein			
FPS10_12960	DNA polymerase I		K02335	L
FPS10_12965	zinc-finger domain-containing protein			S
FPS10_12970	ABC transporter ATP-binding protein	GO:0000166,GO:0005524,GO:0008152,GO:0016887	K01990	V
FPS10_12975	cyanophycin synthetase		K03802	M
FPS10_12980	histidine triad nucleotide-binding protein	GO:0003824,GO:0008152		FGR
FPS10_12985	beta-1%2C6-N-acetylglucosaminyltransferase	GO:0008152,GO:0008375,GO:0016020		
FPS10_12990	sulfotransferase family protein	GO:0008146,GO:0008152,GO:0016021		
FPS10_12995	helix-turn-helix transcriptional regulator			K
FPS10_13000	adenosine kinase	GO:0016301,GO:0016310,GO:0016740		G
FPS10_13005	endonuclease III	GO:0003677,GO:0003824,GO:0003906,GO:0004519,GO:0006281,GO:0006284,GO:0006974,GO:0008152,GO:0016787,GO:0016798,GO:0016829,GO:0019104,GO:0046872,GO:0051536,GO:0051539,GO:0090305	K10773	L
FPS10_13010	AtpZ/AtpI family protein		K02116	S
FPS10_13015	F0F1 ATP synthase subunit A		K02108	C
FPS10_13020	F0F1 ATP synthase subunit C		K02110	C
FPS10_13025	F0F1 ATP synthase subunit B'	GO:0005886,GO:0006754,GO:0006810,GO:0006811,GO:0015078,GO:0015986,GO:0015992,GO:0016020,GO:0016021,GO:0016787,GO:0042777,GO:0045263,GO:0046933	K02109	C
FPS10_13030	F0F1 ATP synthase subunit B	GO:0005886,GO:0006754,GO:0006810,GO:0006811,GO:0015078,GO:0015986,GO:0015992,GO:0016020,GO:0016021,GO:0042777,GO:0045263,GO:0046933	K02109	C
FPS10_13035	hypothetical protein			
FPS10_13040	FCD domain-containing protein	GO:0003677,GO:0003700,GO:0006351,GO:0006355	K05799	K
FPS10_13045	OmpA family protein	GO:0009279,GO:0016020,GO:0016021		M

FPS10_13050	methylated-DNA--[protein]-cysteine S-methyltransferase	GO:0003677,GO:0003700,GO:0003824,GO:0003908,GO:0006281,GO:0006351,GO:0006355,GO:0032259,GO:0043565	K10778	L
FPS10_13055	cytochrome c			C
FPS10_13060	hypothetical protein			
FPS10_13065	hypothetical protein			
FPS10_13070	DMT family transporter	GO:0016020,GO:0016021		GER
FPS10_13075	winged helix-turn-helix transcriptional regulator			K
FPS10_13080	DMT family transporter			GER
FPS10_13085	LysR family transcriptional regulator	GO:0003677,GO:0003700,GO:0006351,GO:0006355		K
FPS10_13090	hypothetical protein			
FPS10_13095	hypothetical protein	GO:0016020,GO:0016021		
FPS10_13100	LysR family transcriptional regulator	GO:0003677,GO:0003700,GO:0006351,GO:0006355		K
FPS10_13105	membrane dipeptidase	GO:0006508,GO:0008235,GO:0008239,GO:0016805	K01273	E
FPS10_13110	ABC transporter substrate-binding protein		K02035	E
FPS10_13115	ABC transporter permease	GO:0005886,GO:0006810,GO:0016020,GO:0016021	K02033	EP
FPS10_13120	ABC transporter permease	GO:0005886,GO:0006810,GO:0016020,GO:0016021	K02034	EP
FPS10_13125	ABC transporter ATP-binding protein		K02031	EP
FPS10_13130	ABC transporter ATP-binding protein	GO:0000166,GO:0005524,GO:0008152,GO:0016887		EP
FPS10_13135	HAD family hydrolase	GO:0008152,GO:0016787		R
FPS10_13140	MarR family transcriptional regulator	GO:0003677,GO:0003700,GO:0006352,GO:0006355,GO:0016987,GO:0030246		K
FPS10_13145	sugar ABC transporter substrate-binding protein	GO:0005215,GO:0006810	K10227	G
FPS10_13150	sugar ABC transporter permease	GO:0005886,GO:0006810,GO:0016020,GO:0016021	K10228	G
FPS10_13155	carbohydrate ABC transporter permease	GO:0005886,GO:0006810,GO:0016020,GO:0016021	K10229	G
FPS10_13160	ABC transporter ATP-binding protein		K10111	G
FPS10_13165	SDR family oxidoreductase	GO:0008152,GO:0016491,GO:0047833,GO:0055114		IQR
FPS10_13170	mannitol dehydrogenase family protein	GO:0003824,GO:0008152,GO:0016491,GO:0019594,GO:0050086,GO:0050662,GO:0055114	K00045	G
FPS10_13175	hypothetical protein			
FPS10_13180	LysR family transcriptional regulator			K
FPS10_13185	AbrB family transcriptional regulator		K07120	R
FPS10_13190	sulfatase-like hydrolase/transferase	GO:0003824,GO:0008152,GO:0008484		P
FPS10_13195	elongation factor 4	GO:0000166,GO:0003746,GO:0003924,GO:0005525,GO:0005886,GO:0006412,GO:0006414,GO:0016020,GO:0016787,GO:0043022,GO:0045727	K03596	M
FPS10_13200	nuclear transport factor 2 family protein			S
FPS10_13205	GYD domain-containing protein			S
FPS10_13210	CBS domain-containing protein			R
FPS10_13215	hypothetical protein		K09004	S
FPS10_13220	glycine zipper family protein			
FPS10_13225	tetratricopeptide repeat protein			R
FPS10_13230	CTP synthetase			
FPS10_13235	hypothetical protein			
FPS10_13240	low molecular weight phosphotyrosine protein phosphatase	GO:0004725,GO:0006470,GO:0035335	K01104	T
FPS10_13245	NAD-dependent deacylase	GO:0008152,GO:0016787,GO:0036047,GO:0036049,GO:0036054,GO:0036055,GO:0070403	K12410	K
FPS10_13250	copper chaperone PCu(A)C		K09796	S
FPS10_13255	hypothetical protein			
FPS10_13260	50S ribosomal protein L28	GO:0003735,GO:0005622,GO:0005840,GO:0006412,GO:0030529	K02902	J
FPS10_13265	MBL fold metallo-hydrolase			Q
FPS10_13270	hypothetical protein			
FPS10_13275	META domain-containing protein			O
FPS10_13280	hypothetical protein			
FPS10_13285	hypothetical protein			
FPS10_13290	hypothetical protein			
FPS10_13300	hypothetical protein			
FPS10_13310	hypothetical protein		K03607	T
FPS10_13315	hypothetical protein		K06919	R
FPS10_13320	hypothetical protein			
FPS10_13325	hypothetical protein			
FPS10_13330	hypothetical protein			
FPS10_13335	hypothetical protein			
FPS10_13340	hypothetical protein			
FPS10_13345	tyrosine-type recombinase/integrase			L
FPS10_13350	thermonuclease family protein			L
FPS10_13355	hypothetical protein			
FPS10_13365	hypothetical protein			

FPS10_13370	hypothetical protein			
FPS10_13375	hypothetical protein			
FPS10_13380	tetratricopeptide repeat protein			R
FPS10_13385	helix-turn-helix transcriptional regulator			K
FPS10_13390	hypothetical protein			
FPS10_13395	FRG domain-containing protein			
FPS10_13400	ATP-binding protein			L
FPS10_13405	IS21 family transposase			L
FPS10_13410	transposase			L
FPS10_13415	integrase			L
FPS10_13420	hypothetical protein			
FPS10_13425	hypothetical protein			S
FPS10_13430	hypothetical protein			
FPS10_13435	tetratricopeptide repeat protein			
FPS10_13440	tyrosine-type recombinase/integrase			L
FPS10_13445	hypothetical protein			
FPS10_13450	hypothetical protein			
FPS10_13455	GntR family transcriptional regulator			K
FPS10_13460	peptidoglycan DD-metalloendopeptidase family protein		K12056	M
FPS10_13465	pilus assembly protein		K12072	
FPS10_13470	conjugal transfer protein TraF		K12057	
FPS10_13475	hypothetical protein			
FPS10_13480	conjugal transfer protein TraN		K12058	
FPS10_13485	type-F conjugative transfer system pilin assembly protein TrbC		K12059	
FPS10_13490	conjugal transfer protein		K12060	
FPS10_13495	hypothetical protein			
FPS10_13500	type-F conjugative transfer system protein TraW		K12061	
FPS10_13505	type VI secretion protein		K12062	OU
FPS10_13510	conjugal transfer protein TrbI			
FPS10_13515	hypothetical protein			
FPS10_13520	type IV secretion system protein TraC		K12063	U
FPS10_13525	hypothetical protein			
FPS10_13530	DsbC family protein	GO:0008152,GO:0016853	K03981	O
FPS10_13535	conjugal transfer protein TraB	GO:0016020,GO:0016021	K12065	S
FPS10_13540	conjugal transfer protein TraK		K12066	
FPS10_13545	conjugal transfer protein TraE	GO:0000746,GO:0016020,GO:0016021	K12067	
FPS10_13550	type IV conjugative transfer system protein TraL		K12068	
FPS10_13555	hypothetical protein			
FPS10_13560	hypothetical protein			
FPS10_13565	hypothetical protein			
FPS10_13570	hypothetical protein			
FPS10_13575	hypothetical protein			
FPS10_13580	hypothetical protein			
FPS10_13585	lytic transglycosylase domain-containing protein			M
FPS10_13595	recombinase family protein			L
FPS10_13600	chromate efflux transporter	GO:0015109,GO:0015703,GO:0016020,GO:0016021	K07240	P
FPS10_13605	superoxide dismutase		K04564	P
FPS10_13610	IS110 family transposase	GO:0003677,GO:0004803,GO:0006281,GO:0006313		
FPS10_13620	ABC transporter permease	GO:0005886,GO:0006810,GO:0016020,GO:0016021	K02053	E
FPS10_13625	ABC transporter permease			
FPS10_13630	hypothetical protein			
FPS10_13635	flagellar motor stator protein MotA	GO:0006810,GO:0008565,GO:0015031,GO:0016020,GO:00160[illegible]1	K02556	N
FPS10_13640	hypothetical protein			S
FPS10_13645	hypothetical protein			
FPS10_13650	flagellar basal body protein FliL	GO:0006935,GO:0009425,GO:0016020,GO:0016021,GO:00719[illegible]3	K02415	N
FPS10_13655	ribonuclease D	GO:0003676,GO:0004527,GO:0006139,GO:0008408,GO:0090[illegible]5	K03684	J
FPS10_13660	KpsF/GutQ family sugar-phosphate isomerase	GO:0005975,GO:0016853,GO:0030246	K06041	R
FPS10_13665	hypothetical protein		K11719	S
FPS10_13670	lipopolysaccharide transport periplasmic protein LptA		K09774	S
FPS10_13675	LPS export ABC transporter ATP-binding protein	GO:0000166,GO:0005524,GO:0008152,GO:0016787,GO:0016887,GO:0043190,GO:0055085	K06861	R
FPS10_13680	ribosome-associated translation inhibitor RaiA	GO:0044238		J
FPS10_13685	PTS lactose transporter subunit IIC		K02806	GT
FPS10_13690	nodulation protein NodH			
FPS10_13695	beta-1%2C6-N-acetylglucosaminyltransferase	GO:0008152,GO:0008375,GO:0016020		

FPS10_13700	glycosyltransferase family 2 protein			
FPS10_13705	glycosyltransferase family 2 protein			
FPS10_13710	UDP-glucose 4-epimerase GalE	GO:0003824,GO:0003978,GO:0006012,GO:0016853,GO:00506[illegible]	K01784	M
FPS10_13715	UTP--glucose-1-phosphate uridylyltransferase GalU	GO:0003983,GO:0006011,GO:0009058,GO:0016740,GO:00167[illegible]	K00963	M
FPS10_13720	3-deoxy-manno-octulosonate cytidylyltransferase	GO:0005737,GO:0008152,GO:0008690,GO:0016740,GO:00167[illegible]	K00979	M
FPS10_13725	3'(2')%2C5'-bisphosphate nucleotidase CysQ		K01082	P
FPS10_13730	ABC transporter permease	GO:0016020,GO:0016021		GM
FPS10_13735	SH3 domain-containing protein			S
FPS10_13740	alpha-ketoglutarate-dependent dioxygenase AlkB		K03919	L
FPS10_13745	molecular chaperone DnaK	GO:0000166,GO:0005524,GO:0006457,GO:0051082	K04043	O
FPS10_13750	molecular chaperone DnaJ	GO:0005524,GO:0005737,GO:0006260,GO:0006457,GO:0008270,GO:0009408,GO:0031072,GO:0046872,GO:0051082	K03686	O
FPS10_13755	JAB domain-containing protein		K03630	L
FPS10_13760	OmpA family protein		K02040	P
FPS10_13765	hypothetical protein			
FPS10_13770	preprotein translocase subunit SecA	GO:0000166,GO:0005524,GO:0005737,GO:0005886,GO:0006605,GO:0006810,GO:0006886,GO:0015031,GO:0016020,GO:0017038,GO:0046872,GO:0065002	K03070	U
FPS10_13775	peptidylprolyl isomerase		K03769	O
FPS10_13780	bifunctional glutamate N-acetyltransferase/amino-acid acetyltransferase ArgJ	GO:0003824,GO:0004042,GO:0004358,GO:0005737,GO:0006526,GO:0008152,GO:0008652,GO:0016740,GO:0016746	K00620	E
FPS10_13785	8-oxo-dGTP diphosphatase MutT	GO:0008152,GO:0016787	K03574	F
FPS10_13790	translation initiation factor IF-2	GO:0000166,GO:0003743,GO:0003924,GO:0005525,GO:0005622,GO:0005737,GO:0006412,GO:0006413	K02519	J
FPS10_13795	RNA-binding protein		K07742	K
FPS10_13800	transcription termination/antitermination protein NusA	GO:0000166,GO:0003676,GO:0003700,GO:0003723,GO:0003746,GO:0005737,GO:0006351,GO:0006353,GO:0006355,GO:0006414,GO:0031554,GO:0031564	K02600	E
FPS10_13805	ribosome maturation factor RimP	GO:0005737,GO:0042254,GO:0042274	K09748	S
FPS10_13810	sodium:proton antiporter			P
FPS10_13815	prolyl aminopeptidase	GO:0004177,GO:0005737,GO:0006508,GO:0008233,GO:00167[illegible]7	K01259	R
FPS10_13820	bifunctional 2-polyprenyl-6-hydroxyphenol methylase/3-demethylubiquinol 3-O-methyltransferase UbiG	GO:0006744,GO:0008168,GO:0008425,GO:0016740,GO:0032259,GO:0061542	K00568	H
FPS10_13825	RNA 2'%2C3'-cyclic phosphodiesterase		K01975	J
FPS10_13830	MarR family transcriptional regulator	GO:0003677,GO:0003700,GO:0006351,GO:0006355		K
FPS10_13835	carbon-nitrogen hydrolase family protein	GO:0006807,GO:0016810	K11206	R
FPS10_13840	glutaredoxin 3	GO:0005623,GO:0009055,GO:0015035,GO:0045454,GO:00551[illegible]4	K03676	O
FPS10_13845	ComF family protein			R
FPS10_13850	SAM-dependent methyltransferase	GO:0008152,GO:0008168,GO:0032259		
FPS10_13855	ferrochelatase	GO:0004325,GO:0005737,GO:0006779,GO:0006783,GO:0016829,GO:0046872	K01772	H
FPS10_13860	peptide-methionine (S)-S-oxide reductase MsrA	GO:0006464,GO:0006979,GO:0008113,GO:0016491,GO:0016671,GO:0030091,GO:0055114	K07304	O
FPS10_13865	aminotransferase class IV		K00826	EH
FPS10_13870	sulfotransferase			
FPS10_13875	SCP2 sterol-binding domain-containing protein			I
FPS10_13880	argininosuccinate synthase	GO:0000166,GO:0004055,GO:0005524,GO:0005737,GO:0006526,GO:0008652,GO:0016874	K01940	E
FPS10_13885	threonine ammonia-lyase IlvA	GO:0004794,GO:0006520,GO:0008652,GO:0009082,GO:0009097,GO:0016829,GO:0030170	K01754	E
FPS10_13890	Hpt domain-containing protein	GO:0000160,GO:0004871,GO:0005622		
FPS10_13895	fused response regulator/phosphatase	GO:0000160,GO:0003824,GO:0005622,GO:0008152	K07315	TK
FPS10_13900	NUDIX hydrolase	GO:0008152,GO:0016787	K03574	LR
FPS10_13905	Hsp33 family molecular chaperone HslO	GO:0005737,GO:0006457,GO:0051082	K04083	O
FPS10_13910	CoA pyrophosphatase	GO:0000287,GO:0009132,GO:0016787,GO:0016818,GO:00301[illegible]5		LR
FPS10_13915	CCA tRNA nucleotidyltransferase	GO:0000166,GO:0003723,GO:0005524,GO:0006396,GO:0016740,GO:0016779	K00970	J
FPS10_13920	LysE family translocator			E
FPS10_13925	ABC transporter ATP-binding protein	GO:0000166,GO:0005524,GO:0006810,GO:0008152,GO:0016020,GO:0016021,GO:0016887,GO:0042626,GO:0055085	K18893	V
FPS10_13930	ABC transporter ATP-binding protein	GO:0000166,GO:0005524,GO:0006810,GO:0008152,GO:0016020,GO:0016021,GO:0016887,GO:0042626,GO:0055085	K06147	V
FPS10_13935	class I SAM-dependent RNA methyltransferase	GO:0001510,GO:0006396,GO:0008168,GO:0008173,GO:0016740,GO:0032259	K03215	J
FPS10_13940	ion transporter	GO:0005216,GO:0006810,GO:0006811,GO:0016020,GO:0016021,GO:0034220,GO:0055085	K08714	
FPS10_13945	L%2CD-transpeptidase family protein	GO:0008152,GO:0016740		S
FPS10_13950	CAP domain-containing protein			S
FPS10_13955	L%2CD-transpeptidase			S

FPS10_13960	hypothetical protein	GO:0016020,GO:0016021		
FPS10_13965	hypothetical protein			
FPS10_13970	ribokinase	GO:0004747,GO:0006014,GO:0016301,GO:0016310,GO:0016740,GO:0046835	K00852	G
FPS10_13975	NADP-dependent malic enzyme		K00029	C
FPS10_13980	DNA mismatch repair protein MutS		K03555	L
FPS10_13985	nucleotide exchange factor GrpE		K03687	O
FPS10_13990	heat-inducible transcriptional repressor HrcA	GO:0003677,GO:0006351,GO:0006355,GO:0045892	K03705	K
FPS10_13995	ribonuclease PH		K00989	J
FPS10_14000	RdgB/HAM1 family non-canonical purine NTP pyrophosphatase	GO:0000166,GO:0006163,GO:0009117,GO:0009143,GO:0016787,GO:0017111,GO:0046872,GO:0047429	K02428	F
FPS10_14005	coproporphyrinogen III oxidase	GO:0003824,GO:0004109,GO:0005737,GO:0006779,GO:0051536,GO:0055114		H
FPS10_14010	DUF454 domain-containing protein	GO:0016020,GO:0016021	K09790	S
FPS10_14015	ParB/RepB/Spo0J family partition protein		K03497	K
FPS10_14020	ParA family protein		K03496	D
FPS10_14025	16S rRNA (guanine(527)-N(7))-methyltransferase RsmG	GO:0005737,GO:0006364,GO:0008168,GO:0008649,GO:0016740,GO:0031167,GO:0032259,GO:0070043,GO:0070476	K03501	M
FPS10_14030	tRNA uridine-5-carboxymethylaminomethyl(34) synthesis enzyme MnmG	GO:0002098,GO:0005737,GO:0008033,GO:0016491,GO:0050660,GO:0055114	K03495	D
FPS10_14035	tRNA uridine-5-carboxymethylaminomethyl(34) synthesis GTPase MnmE	GO:0000166,GO:0003924,GO:0005525,GO:0005622,GO:0005737,GO:0006400,GO:0008033,GO:0016787,GO:0046872	K03650	R
FPS10_14040	transcription termination factor Rho	GO:0000166,GO:0003676,GO:0003723,GO:0004386,GO:0005524,GO:0006351,GO:0006353,GO:0006355,GO:0008186,GO:0016787	K03628	K
FPS10_14045	CopD family protein		K08973	S
FPS10_14050	septum formation protein Maf	GO:0005737	K06287	D
FPS10_14055	shikimate dehydrogenase	GO:0004764,GO:0008652,GO:0009073,GO:0009423,GO:0016491,GO:0019632,GO:0050661,GO:0055114	K00014	E
FPS10_14060	dephospho-CoA kinase	GO:0000166,GO:0004140,GO:0005524,GO:0005737,GO:0015937,GO:0016301,GO:0016310,GO:0016740	K00859	H
FPS10_14065	DNA polymerase III subunit epsilon	GO:0003676,GO:0003677,GO:0003887,GO:0006260,GO:0016740,GO:0016779,GO:0071897	K02342	L
FPS10_14070	glycoside hydrolase family 25 protein	GO:0003796,GO:0009975,GO:0009253,GO:0016787,GO:0016998	K07273	M
FPS10_14075	ATP-dependent protease ATPase subunit HslU		K03667	O
FPS10_14080	alpha/beta fold hydrolase			S
FPS10_14085	MFS transporter	GO:0016020,GO:0016021,GO:0055085		
FPS10_14090	DNA mismatch repair protein MutS			S
FPS10_14095	murein transglycosylase	GO:0004553,GO:0009254,GO:0019867	K08304	M
FPS10_14100	Tim44 domain-containing protein			S
FPS10_14105	FxsA family protein	GO:0016020,GO:0016021	K07113	R
FPS10_14110	protein-export chaperone SecB	GO:0005737,GO:0006457,GO:0006810,GO:0015031,GO:0051082,GO:0051262	K03071	U
FPS10_14115	ATP-dependent protease subunit HslV		K01419	O
FPS10_14120	thioredoxin		K03671	O
FPS10_14125	double-strand break repair helicase AddA	GO:0000166,GO:0003677,GO:0004003,GO:0004386,GO:0004518,GO:0004527,GO:0005524,GO:0006281,GO:0006974,GO:0016787,GO:0032508,GO:0090305	K16898	L
FPS10_14130	double-strand break repair protein AddB			L
FPS10_14135	nucleotidyltransferase family protein	GO:0008152,GO:0016740	K00992	MJ
FPS10_14140	phosphotransferase		K07102	R
FPS10_14145	tRNA (adenosine(37)-N6)-threonylcarbamoyltransferase complex ATPase subunit type 1 TsaE	GO:0002949	K06925	R
FPS10_14150	diguanylate cyclase			
FPS10_14155	ActS/PrrB/RegB family redox-sensitive histidine kinase	GO:0000155,GO:0000160,GO:0000166,GO:0005524,GO:0005622,GO:0007165,GO:0016020,GO:0016021,GO:0016301,GO:0016310,GO:0016740,GO:0016772,GO:0023014	K15011	T
FPS10_14160	SCO family protein		K07152	R
FPS10_14165	ActR/PrrA/RegA family redox response regulator transcription factor	GO:0000160,GO:0003677,GO:0005622,GO:0043565	K15012	TK
FPS10_14170	hypothetical protein			
FPS10_14175	hypothetical protein			
FPS10_14180	HD family hydrolase	GO:0008152,GO:0016787	K06952	R
FPS10_14185	adenosylhomocysteinase		K01251	H
FPS10_14190	DUF2853 family protein	GO:0003674,GO:0005575,GO:0008150		
FPS10_14195	acyl carrier protein			
FPS10_14200	type III PLP-dependent enzyme		K01586	E
FPS10_14205	acyl--CoA ligase			IQ
FPS10_14210	GSCFA domain-containing protein			
FPS10_14215	GNAT family N-acetyltransferase			J

FPS10_14220	amidohydrolase family protein	GO:0004151,GO:0006221,GO:0008270,GO:0016787,GO:0016810,GO:0044205,GO:0046872	K01465	F
FPS10_14225	hypothetical protein			
FPS10_14230	aspartate carbamoyltransferase catalytic subunit	GO:0004070,GO:0006207,GO:0006221,GO:0006520,GO:0016597,GO:0016740,GO:0016743,GO:0044205	K00609	F
FPS10_14235	aspartate aminotransferase family protein	GO:0003824,GO:0008152,GO:0008483,GO:0016740,GO:00301[illegible]0		H
FPS10_14240	class 1 fructose-bisphosphatase	GO:0000287,GO:0005737,GO:0005975,GO:0016051,GO:0016311,GO:0016787,GO:0042132,GO:0042578,GO:0046872	K03841	G
FPS10_14245	transketolase		K00615	G
FPS10_14250	RpiB/LacA/LacB family sugar-phosphate isomerase		K21911	G
FPS10_14255	triose-phosphate isomerase	GO:0003824,GO:0004807,GO:0005737,GO:0006094,GO:0006096,GO:0006098,GO:0008152,GO:0016853	K21910	G
FPS10_14260	DeoR/GlpR transcriptional regulator	GO:0003677,GO:0003700,GO:0005622,GO:0006351,GO:00063[illegible]5		KG
FPS10_14265	dihydroxyacetone kinase subunit DhaK		K00863	G
FPS10_14270	GntR family transcriptional regulator	GO:0003677,GO:0003700,GO:0006351,GO:0006355		K
FPS10_14275	SDR family oxidoreductase	GO:0003824,GO:0008152,GO:0050662	K22025	MG
FPS10_14280	sugar phosphate isomerase/epimerase			G
FPS10_14285	TRAP transporter substrate-binding protein	GO:0006810,GO:0030288		Q
FPS10_14290	TRAP transporter small permease	GO:0016020,GO:0016021		G
FPS10_14295	TRAP transporter large permease subunit			Q
FPS10_14300	DMT family transporter	GO:0016020,GO:0016021		GER
FPS10_14305	SDR family oxidoreductase	GO:0008152,GO:0016491,GO:0055114		R
FPS10_14310	branched-chain amino acid ABC transporter permease	GO:0016020,GO:0016021		E
FPS10_14315	AzlD domain-containing protein	GO:0016020,GO:0016021		S
FPS10_14320	RraA family protein	GO:0003824		H
FPS10_14325	LysE family translocator	GO:0005886,GO:0006865,GO:0016020,GO:0016021		E
FPS10_14330	dihydrodipicolinate synthase family protein	GO:0003824,GO:0008152,GO:0016829	K13876	EM
FPS10_14335	fumarylacetoacetate hydrolase family protein	GO:0003674,GO:0003824,GO:0005575,GO:0008150,GO:00081[illegible]2		R
FPS10_14340	malonate transporter		K07088	R
FPS10_14345	2-hydroxyacid dehydrogenase	GO:0008152,GO:0016616,GO:0051287,GO:0055114		CHR
FPS10_14350	SMP-30/gluconolactonase/LRE family protein		K01053	G
FPS10_14355	ABC transporter permease		K10440	G
FPS10_14360	ABC transporter permease	GO:0005215,GO:0005886,GO:0006810,GO:0016020,GO:001602[illegible]1	K10440	G
FPS10_14365	sugar ABC transporter ATP-binding protein	GO:0000166,GO:0005524,GO:0008152,GO:0016887	K10441	G
FPS10_14370	ABC transporter substrate-binding protein		K10439	G
FPS10_14375	ATP-binding protein			T
FPS10_14380	response regulator transcription factor	GO:0000160,GO:0003677,GO:0005622,GO:0006351,GO:00063[illegible]5		TK
FPS10_14385	shikimate dehydrogenase		K00014	E
FPS10_14390	2-hydroxy-3-oxopropionate reductase	GO:0004616,GO:0008679,GO:0016491,GO:0046487,GO:0051287,GO:0055114	K00042	I
FPS10_14395	hydroxypyruvate isomerase	GO:0008152,GO:0008903,GO:0016853	K01816	G
FPS10_14400	transketolase	GO:0003824,GO:0004802,GO:0008152,GO:0016740,GO:00468[illegible]2	K00615	G
FPS10_14405	GntR family transcriptional regulator			K
FPS10_14410	aldehyde dehydrogenase (NADP(+))	GO:0008152,GO:0016491,GO:0016620,GO:0055114	K13877	C
FPS10_14415				
FPS10_14420	heme biosynthesis protein HemY	GO:0016020,GO:0016021	K02498	S
FPS10_14425	hypothetical protein			
FPS10_14430	uroporphyrinogen-III synthase		K01719	H
FPS10_14435	tRNA (adenosine(37)-N6)-threonylcarbamoyltransferase complex transferase subunit TsaD	GO:0002949,GO:0004222,GO:0005506,GO:0005737,GO:0006508,GO:0008033,GO:0016740,GO:0016746,GO:0016747,GO:0046872,GO:0061711	K01409	O
FPS10_14440	flagellar basal body P-ring protein FlgI	GO:0001539,GO:0005198,GO:0009288,GO:0009425,GO:0009428,GO:0030288,GO:0042597,GO:0071973	K02394	N
FPS10_14445	hypothetical protein		K02397	
FPS10_14450	flagellar hook-associated protein FlgK		K02396	N
FPS10_14455	flagellar hook-basal body complex protein	GO:0009288,GO:0009425,GO:0071973	K02390	N
FPS10_14460	chemotaxis protein MotB	GO:0016020,GO:0016021	K02557	N
FPS10_14465	polysaccharide export protein		K01991	M
FPS10_14470	hypothetical protein			
FPS10_14475	rhomboid family intramembrane serine protease			R
FPS10_14480	hypothetical protein			
FPS10_14485	EVE domain-containing protein			S
FPS10_14490	YciI family protein		K09780	S
FPS10_14495	sugar phosphate isomerase/epimerase		K01805	G
FPS10_14500	hypothetical protein			
FPS10_14505	DUF1810 domain-containing protein			S
FPS10_14510	SDR family oxidoreductase			MG

FPS10_14515	hypothetical protein			
FPS10_14520	1-deoxy-D-xylulose-5-phosphate synthase	GO:0000287,GO:0003824,GO:0008152,GO:0008299,GO:0008661,GO:0009228,GO:0016114,GO:0016740,GO:0030976,GO:0046872,GO:0052865	K01662	HI
FPS10_14525	polyprenyl synthetase family protein	GO:0004161,GO:0004337,GO:0008299,GO:0016740	K00795	H
FPS10_14530	exodeoxyribonuclease VII small subunit	GO:0004518,GO:0004527,GO:0005737,GO:0006308,GO:0008855,GO:0009318,GO:0016787,GO:0090305	K03602	L
FPS10_14535	histone deacetylase family protein			BQ
FPS10_14540	response regulator	GO:0000160,GO:0003677,GO:0005622,GO:0006351,GO:00063[illegible]5	K07659	TK
FPS10_14545	MarR family transcriptional regulator	GO:0003677,GO:0003700,GO:0006351,GO:0006355		K
FPS10_14550	branched-chain amino acid aminotransferase	GO:0003824,GO:0004084,GO:0008152,GO:0008483,GO:0009081,GO:0016740,GO:0052654,GO:0052655,GO:0052656	K00826	EH
FPS10_14555	hypothetical protein			
FPS10_14560	hypothetical protein			
FPS10_14565	tRNA epoxyqueuosine(34) reductase QueG	GO:0005737,GO:0008033,GO:0008616,GO:0016491,GO:0046872,GO:0051536,GO:0051539,GO:0052693,GO:0055114	K18979	C
FPS10_14570	glutathione S-transferase family protein	GO:0008152,GO:0016740	K00799	O
FPS10_14575	monofunctional biosynthetic peptidoglycan transglycosylase		K03814	M
FPS10_14580	hypothetical protein			
FPS10_14585	glutamate synthase large subunit		K00265	E
FPS10_14590	hypothetical protein			
FPS10_14595	NAD(P)-dependent oxidoreductase	GO:0006537,GO:0016491,GO:0016639,GO:0051536,GO:00551[illegible]4	K00266	ER
FPS10_14600	undecaprenyl-diphosphate phosphatase	GO:0005886,GO:0008360,GO:0009252,GO:0016020,GO:00160[illegible]1,GO:0016311,GO:0016787,GO:0046677,GO:0050380,GO:0071555	K06153	V
FPS10_14605	complex I NDUFA9 subunit family protein	GO:0003824,GO:0008152,GO:0050662	K00329	MG
FPS10_14610				
FPS10_14615	tyrosine-type recombinase/integrase			L
FPS10_14620	hypothetical protein			S
FPS10_14625	recombinase family protein			L
FPS10_14630	DUF1127 domain-containing protein			S
FPS10_14635	substrate-binding domain-containing protein		K02529	G
FPS10_14640	FAD-dependent oxidoreductase	GO:0016491,GO:0051536,GO:0051539,GO:0055114	K15022	ER
FPS10_14645	(2Fe-2S)-binding protein	GO:0005506,GO:0008901,GO:0009055,GO:0016491,GO:0046872,GO:0051536,GO:0051539,GO:0055114		R
FPS10_14650	ABC transporter substrate-binding protein		K02051	P
FPS10_14655	ABC transporter permease subunit		K02050	P
FPS10_14660	ATP-binding cassette domain-containing protein		K02049	P
FPS10_14665	DUF3329 domain-containing protein			
FPS10_14670	MarR family transcriptional regulator			K
FPS10_14675	response regulator	GO:0000155,GO:0000160,GO:0005622,GO:0007165,GO:0016301,GO:0016310,GO:0016772,GO:0023014		T
FPS10_14680	alpha-2-macroglobulin family protein	GO:0004866,GO:0010951	K06894	R
FPS10_14685	penicillin-binding protein 1C	GO:0008658,GO:0008955,GO:0009252	K05367	M
FPS10_14690	acetyl-CoA C-acyltransferase family protein	GO:0003824,GO:0003985,GO:0008152,GO:0016740,GO:0016746,GO:0016747	K00626	I
FPS10_14695	winged helix-turn-helix transcriptional regulator	GO:0003677,GO:0003700,GO:0006351,GO:0006355		K
FPS10_14700	glycine C-acetyltransferase	GO:0003824,GO:0006567,GO:0008890,GO:0009058,GO:0016740,GO:0016746,GO:0016874,GO:0019518,GO:0030170	K00639	H
FPS10_14705	L-threonine 3-dehydrogenase		K00060	ER
FPS10_14710	amino acid ABC transporter substrate-binding protein			ET
FPS10_14715	homogentisate 1%2C2-dioxygenase	GO:0004411,GO:0006559,GO:0006570,GO:0016491,GO:0046872,GO:0051213,GO:0055114	K00451	Q
FPS10_14720	phosphoglycerate dehydrogenase	GO:0004617,GO:0006564,GO:0008152,GO:0008652,GO:0016491,GO:0016616,GO:0051287,GO:0055114	K00058	HE
FPS10_14725	phosphoserine transaminase	GO:0003824,GO:0004648,GO:0005737,GO:0006564,GO:0008483,GO:0016740	K00831	HE
FPS10_14730	phosphoserine phosphatase SerB		K01079	E
FPS10_14735	TSUP family transporter		K07090	R
FPS10_14740	hypothetical protein			S
FPS10_14745	asparaginase			E
FPS10_14750	hypothetical protein			
FPS10_14755	23S rRNA (adenine(2503)-C(2))-methyltransferase RlmN	GO:0000049,GO:0002935,GO:0003824,GO:0005737,GO:0006364,GO:0008033,GO:0008168,GO:0008173,GO:0016740,GO:0019843,GO:0030488,GO:0032259,GO:0046872,GO:0051536,GO:0051539,GO:0070040,GO:0070475	K06941	R
FPS10_14760	hypothetical protein			S
FPS10_14765	class I SAM-dependent methyltransferase			QR
FPS10_14770	TIGR00730 family Rossman fold protein	GO:0009691,GO:0016787,GO:0016799		R
FPS10_14775	threonine/serine dehydratase	GO:0004794,GO:0006520,GO:0016829,GO:0030170	K01754	E

FPS10_14780	2%2C3%2C4%2C5-tetrahydropyridine-2%2C6-dicarboxylate N-succinyltransferase	GO:0005737,GO:0008652,GO:0008666,GO:0009085,GO:0009089,GO:0016740,GO:0016746,GO:0019877	K00674	E
FPS10_14785	hypothetical protein			
FPS10_14790	hypothetical protein			
FPS10_14795	hypothetical protein			
FPS10_14800	MFS transporter		K06902	R
FPS10_14805	penicillin-insensitive murein endopeptidase	GO:0004252,GO:0006508,GO:0030288	K07261	M
FPS10_14810	esterase-like activity of phytase family protein			S
FPS10_14815	glycine/betaine ABC transporter substrate-binding protein	GO:0005215,GO:0006810	K02002	E
FPS10_14820	response regulator			T
FPS10_14825	YjjW family glycine radical enzyme activase	GO:0003824,GO:0006006,GO:0016491,GO:0051536,GO:0051539,GO:0055114		O
FPS10_14830	YjjI family glycine radical enzyme			
FPS10_14835	isocitrate/isopropylmalate dehydrogenase family protein	GO:0000287,GO:0016491,GO:0016616,GO:0051287,GO:0055114	K00052	CE
FPS10_14840	lasso peptide biosynthesis B2 protein			
FPS10_14845	PqqD family protein			
FPS10_14850	phosphoenolpyruvate carboxykinase (ATP)			T
FPS10_14855	ABC transporter ATP-binding protein			V
FPS10_14860	adenine deaminase	GO:0000034,GO:0006146,GO:0016787,GO:0016810	K01486	F
FPS10_14865	AMP nucleosidase	GO:0003824,GO:0008152,GO:0008714,GO:0009116,GO:0016787,GO:0016798,GO:0044209,GO:0046033	K01241	F
FPS10_14870	HU family DNA-binding protein	GO:0003677	K03530	L
FPS10_14875	DMT family transporter	GO:0016020,GO:0016021		GER
FPS10_14880	cytochrome c biogenesis protein CcdA	GO:0016020,GO:0016021,GO:0017004,GO:0055114		O
FPS10_14885	thioredoxin family protein	GO:0005623,GO:0045454		OC
FPS10_14890	AEC family transporter		K07088	R
FPS10_14895	chorismate synthase	GO:0004107,GO:0008652,GO:0009073,GO:0009423,GO:0016829	K01736	E
FPS10_14900	DUF1349 domain-containing protein		K09702	S
FPS10_14905	DMT family transporter	GO:0016020,GO:0016021		GER
FPS10_14910	hypothetical protein			
FPS10_14915	cytochrome c1	GO:0008121,GO:0009055,GO:0016020,GO:0016021,GO:0016491,GO:0020037,GO:0055114,GO:1902600	K00413	C
FPS10_14920	cytochrome b		K00412	C
FPS10_14925	ubiquinol-cytochrome c reductase iron-sulfur subunit	GO:0008121,GO:0016020,GO:0016491,GO:0016679,GO:0046872,GO:0051536,GO:0051537,GO:0055114,GO:1902600	K00411	C
FPS10_14930	porin family protein	GO:0016021	K12980	M
FPS10_14935	glutathione S-transferase	GO:0008152,GO:0016740		O
FPS10_14940	tRNA (N(6)-L-threonylcarbamoyladenosine(37)-C(2))-methylthiotransferase MtaB	GO:0003824,GO:0009451,GO:0016740,GO:0043412,GO:0051536,GO:0051539	K18707	J
FPS10_14945	diaminopimelate epimerase		K01778	E
FPS10_14950				
FPS10_14955	hypothetical protein		K06076	I
FPS10_14960	hypothetical protein		K05569	P
FPS10_14965	cation:proton antiporter	GO:0015075,GO:0016020,GO:0016021,GO:0034220	K05570	P
FPS10_14970	monovalent cation/H(+) antiporter subunit G	GO:0005451,GO:0015672,GO:0015992,GO:0016020,GO:0016021,GO:1902600	K05571	P
FPS10_14975	DUF4040 domain-containing protein	GO:0016020,GO:0016021		P
FPS10_14980	sodium:proton antiporter		K05566	P
FPS10_14985	cation:proton antiporter		K05567	P
FPS10_14990	NADH-quinone oxidoreductase subunit F			CP
FPS10_14995	NADH-quinone oxidoreductase subunit H	GO:0005886,GO:0016020,GO:0016021,GO:0016491,GO:0055114	K00337	C
FPS10_15000	NADH-quinone oxidoreductase subunit B		K00331	C
FPS10_15005	NADH-quinone oxidoreductase subunit C	GO:0008137,GO:0016491,GO:0055114	K00332	C
FPS10_15010	NADH-quinone oxidoreductase subunit D	GO:0016491,GO:0016651,GO:0048038,GO:0051287,GO:0055114	K00333	C
FPS10_15015	4Fe-4S dicluster domain-containing protein		K00266	ER
FPS10_15020	rubrerythrin	GO:0016491,GO:0046872,GO:0055114		S
FPS10_15025	N-carbamoyl-D-amino-acid hydrolase	GO:0006807,GO:0016740,GO:0016746,GO:0016810	K01459	R
FPS10_15030	diaminopropionate ammonia-lyase	GO:0008152,GO:0008838,GO:0016829,GO:0030170	K01751	E
FPS10_15035	DctP family TRAP transporter solute-binding subunit	GO:0006810,GO:0030288	K21395	G
FPS10_15040	TRAP transporter large permease			G
FPS10_15045	TRAP transporter small permease	GO:0016020,GO:0016021		G
FPS10_15050	GNAT family N-acetyltransferase	GO:0008080,GO:0008152		R
FPS10_15060	ABC transporter ATP-binding protein	GO:0000166,GO:0005215,GO:0005524,GO:0006810,GO:0008152,GO:0016820,GO:0016887,GO:0043190,GO:0055085	K02052	E
FPS10_15065	extracellular solute-binding protein		K02055	E
FPS10_15070	ABC transporter permease	GO:0005886,GO:0006810,GO:0016020,GO:0016021	K02054	E
FPS10_15075	ABC transporter permease	GO:0005886,GO:0006810,GO:0016020,GO:0016021	K02053	E

FPS10_15080	HyuE hydantoin racemase	GO:0006807,GO:0036361	K16841	E
FPS10_15085	sugar ABC transporter substrate-binding protein		K10552	G
FPS10_15090	ABC transporter permease	GO:0005215,GO:0005886,GO:0006810,GO:0016020,GO:0016021	K10553	G
FPS10_15095	sugar ABC transporter ATP-binding protein	GO:0005524,GO:0008152,GO:0015407,GO:0015749,GO:0016787,GO:0016887	K10554	G
FPS10_15100	hypothetical protein			
FPS10_15105	phosphoglycerate dehydrogenase	GO:0051287,GO:0055114		HE
FPS10_15110	sulfite exporter TauE/SafE family protein	GO:0016020,GO:0016021	K07090	R
FPS10_15115	matrixin family metalloprotease			
FPS10_15120	matrixin family metalloprotease			
FPS10_15125	30S ribosomal protein S21		K02970	J
FPS10_15130	COQ9 family protein	GO:0006744	K18587	S
FPS10_15135	NAD(P)H-quinone oxidoreductase			CR
FPS10_15140	aquaporin Z	GO:0005215,GO:0005886,GO:0006810,GO:0006833,GO:0015250,GO:0016020,GO:0016021,GO:0055085	K06188	G
FPS10_15145	ribonuclease T	GO:0003723,GO:0033897,GO:0090502	K01166	J
FPS10_15150	DUF1013 domain-containing protein		K09987	S
FPS10_15155	hypothetical protein			
FPS10_15160	recombination protein RecR		K06187	L
FPS10_15165	YbaB/EbfC family nucleoid-associated protein	GO:0003677,GO:0005737,GO:0009295,GO:0043590	K09747	S
FPS10_15170	iron reductase		K17247	S
FPS10_15175	phenylalanine--tRNA ligase subunit beta		K01890	J
FPS10_15180	hypothetical protein			
FPS10_15185	hypothetical protein			
FPS10_15190	phenylalanine--tRNA ligase subunit alpha	GO:0000049,GO:0000166,GO:0000287,GO:0004812,GO:0004826,GO:0005524,GO:0005737,GO:0006412,GO:0006418,GO:0006432,GO:0016874,GO:0043039,GO:0046872	K01889	J
FPS10_15195	hypothetical protein			
FPS10_15200	50S ribosomal protein L20		K02887	J
FPS10_15205	50S ribosomal protein L35	GO:0003735,GO:0005622,GO:0005840,GO:0006412,GO:0030529	K02916	J
FPS10_15210	hypothetical protein			S
FPS10_15215	N-acetyltransferase		K03824	R
FPS10_15220	hypothetical protein	GO:0016020,GO:0016021		
FPS10_15225	pyruvate kinase	GO:0000287,GO:0003824,GO:0004743,GO:0006096,GO:0016301,GO:0016310,GO:0016740,GO:0030955	K00873	G
FPS10_15230	N-formylglutamate amidohydrolase	GO:0008745,GO:0009253,GO:0016787		E
FPS10_15235	DUF1244 domain-containing protein		K09948	S
FPS10_15240	hypothetical protein			
FPS10_15245	D-amino-acid transaminase		K00824	EH
FPS10_15250	dipeptide epimerase	GO:0003824,GO:0008152,GO:0046872	K19802	MR
FPS10_15255	DUF1611 domain-containing protein			S
FPS10_15260	4-(cytidine 5'-diphospho)-2-C-methyl-D-erythritol kinase	GO:0000166,GO:0005524,GO:0008299,GO:0016114,GO:0016301,GO:0016310,GO:0016740,GO:0019288,GO:0050515	K00919	I
FPS10_15265	tetratricopeptide repeat protein			R
FPS10_15270	electron transfer flavoprotein-ubiquinone oxidoreductase	GO:0004174,GO:0016491,GO:0055114	K00311	C
FPS10_15275	dihydroxy-acid dehydratase		K01687	EG
FPS10_15280	transcription elongation factor GreA		K03624	K
FPS10_15285	hypothetical protein			
FPS10_15290	GNAT family N-acetyltransferase	GO:0008080,GO:0008152		R
FPS10_15300	aa3-type cytochrome c oxidase subunit IV			
FPS10_15310	MBL fold metallo-hydrolase			R
FPS10_15315	acyl-CoA dehydrogenase	GO:0003995,GO:0008152,GO:0016491,GO:0016627,GO:0050660,GO:0055114		I
FPS10_15320	threonylcarbamoyl-AMP synthase	GO:0000166,GO:0003725,GO:0005524,GO:0005737,GO:0008033,GO:0016740,GO:0016779,GO:0061710	K07566	J
FPS10_15325	hypothetical protein			
FPS10_15330	TlpA family protein disulfide reductase			OC
FPS10_15335	argininosuccinate lyase		K01755	E
FPS10_15340	hypothetical protein			
FPS10_15345	DUF374 domain-containing protein		K09778	S
FPS10_15350	diaminopimelate decarboxylase		K01586	E
FPS10_15355	TIGR02302 family protein	GO:0016020,GO:0016021		
FPS10_15360	fatty acid desaturase		K10255	I
FPS10_15365	polyprenyl synthetase family protein	GO:0008299,GO:0016740	K02523	H
FPS10_15370	DUF2007 domain-containing protein	GO:0006461,GO:0016151,GO:0019627		
FPS10_15375	methyltransferase	GO:0003676,GO:0008168,GO:0032259		R
FPS10_15380	DUF465 domain-containing protein			S

FPS10_15385	EamA family transporter	GO:0016020,GO:0016021		GER
FPS10_15390	hypothetical protein			G
FPS10_15395	GDP-mannose 4%2C6-dehydratase		K01711	M
FPS10_15400	GDP-L-fucose synthase	GO:0003824,GO:0008152,GO:0016491,GO:0016853,GO:0042351,GO:0050577,GO:0050662,GO:0055114,GO:0070401	K02377	MG
FPS10_15405	RNA pyrophosphohydrolase	GO:0008152,GO:0016787,GO:0016818,GO:0046872	K08311	LR
FPS10_15410	S41 family peptidase	GO:0006508,GO:0008233,GO:0008236,GO:0016787	K03797	M
FPS10_15415	peptidoglycan DD-metalloendopeptidase family protein			D
FPS10_15420	2%2C3-bisphosphoglycerate-independent phosphoglycerate mutase	GO:0003824,GO:0004619,GO:0005737,GO:0006007,GO:00060[illegible]6,GO:0008152,GO:0016853,GO:0030145,GO:0046537,GO:0046872	K15633	G
FPS10_15425	23S rRNA (pseudouridine(1915)-N(3))-methyltransferase RlmH	GO:0005737,GO:0006364,GO:0008168,GO:0016740,GO:0031167,GO:0032259,GO:0070038	K00783	S
FPS10_15430	ribosome silencing factor		K09710	S
FPS10_15435	mechanosensitive ion channel	GO:0016020,GO:0016021,GO:0055085		M
FPS10_15440	3-isopropylmalate dehydratase large subunit	GO:0003861,GO:0008152,GO:0008652,GO:0009082,GO:0009098,GO:0016829,GO:0046872,GO:0051536,GO:0051539	K01703	E
FPS10_15445	hypothetical protein			S
FPS10_15450	3-isopropylmalate dehydratase small subunit	GO:0003861,GO:0008152,GO:0008652,GO:0009082,GO:0009098,GO:0009316,GO:0016829	K01704	E
FPS10_15460	endonuclease/exonuclease/phosphatase family protein			
FPS10_15465	3-isopropylmalate dehydrogenase	GO:0000287,GO:0003862,GO:0005737,GO:0008652,GO:0009082,GO:0009098,GO:0016491,GO:0016616,GO:0046872,GO:0051287,GO:0055114	K00052	CE
FPS10_15470	DMT family transporter	GO:0016020,GO:0016021		GER
FPS10_15475				
FPS10_15485	hypothetical protein			
FPS10_15490	HAMP domain-containing protein		K03406	NT
FPS10_15495	winged helix-turn-helix domain-containing protein		K09927	S
FPS10_15500	hypothetical protein		K00616	G
FPS10_15505	carbohydrate kinase		K00847	G
FPS10_15510	L-iditol 2-dehydrogenase	GO:0008152,GO:0016491,GO:0047833,GO:0055114	K21620	IQR
FPS10_15515	SIS domain-containing protein	GO:0005975,GO:0030246	K08094	M
FPS10_15520	class II D-tagatose-bisphosphate aldolase%2C non-catalytic subunit		K16371	G
FPS10_15525	sugar kinase		K21621	G
FPS10_15530	LacI family transcriptional regulator			K
FPS10_15535	sugar ABC transporter substrate-binding protein	GO:0005215,GO:0006810	K10227	G
FPS10_15540	sugar ABC transporter permease		K10228	G
FPS10_15545	carbohydrate ABC transporter permease	GO:0005886,GO:0006810,GO:0016020,GO:0016021	K10229	G
FPS10_15550	ABC transporter ATP-binding protein		K10111	G
FPS10_15555	galactose mutarotase		K01785	G
FPS10_15560	L-iditol 2-dehydrogenase	GO:0003939,GO:0008152,GO:0016491,GO:0055114	K21620	IQR
FPS10_15565	mannitol dehydrogenase family protein	GO:0003824,GO:0008152,GO:0016491,GO:0019594,GO:0050662,GO:0055114	K00045	G
FPS10_15570	zinc-dependent alcohol dehydrogenase family protein		K21616	ER
FPS10_15575	extracellular solute-binding protein	GO:0006810,GO:0015846,GO:0019808,GO:0042597	K11069	E
FPS10_15580	ABC transporter permease	GO:0005886,GO:0006810,GO:0016020,GO:0016021	K11070	E
FPS10_15585	ABC transporter permease	GO:0005886,GO:0006810,GO:0016020,GO:0016021	K11071	E
FPS10_15590	ABC transporter ATP-binding protein	GO:0000166,GO:0005215,GO:0005524,GO:0005886,GO:0006810,GO:0008152,GO:0015417,GO:0015594,GO:0015595,GO:0015846,GO:0015847,GO:0016020,GO:0016787,GO:0016820,GO:0016887,GO:0043190,GO:0055085,GO:1902047,GO:1903711	K11072	E
FPS10_15595	N-acetylneuraminate lyase	GO:0003824,GO:0008152,GO:0016829	K01639	EM
FPS10_15605	DctP family TRAP transporter solute-binding subunit	GO:0006810,GO:0030288		G
FPS10_15610	TRAP transporter small permease subunit	GO:0016020,GO:0016021		G
FPS10_15615	TRAP transporter large permease	GO:0016020,GO:0016021		G
FPS10_15620	putative N-acetylmannosamine-6-phosphate 2-epimerase		K01788	G
FPS10_15625	ROK family protein	GO:0016301,GO:0016310	K00885	KG
FPS10_15630	glucosamine-6-phosphate deaminase	GO:0004342,GO:0005975,GO:0006044,GO:0016787	K02564	G
FPS10_15635	DeoR/GlpR transcriptional regulator			KG
FPS10_15640	ABC transporter permease		K17203	G
FPS10_15645	sugar ABC transporter ATP-binding protein		K17204	G
FPS10_15650	DUF2291 domain-containing protein			R
FPS10_15655	D-ribose ABC transporter substrate-binding protein		K17202	G
FPS10_15660	fucose isomerase	GO:0005737,GO:0005996,GO:0006004,GO:0008736,GO:00168[illegible]1		G
FPS10_15665	transketolase		K00615	G
FPS10_15670	transketolase family protein	GO:0003824,GO:0008152	K00615	G
FPS10_15675	glycerol kinase	GO:0005975,GO:0016301,GO:0016310,GO:0016740,GO:00167[illegible]3	K00864	C

FPS10_15680	HAD-IIA family hydrolase			G
FPS10_15685	DUF3830 family protein			
FPS10_15695	NAD-glutamate dehydrogenase		K15371	E
FPS10_15700	sugar phosphate isomerase/epimerase		K23248	G
FPS10_15705	hypothetical protein			
FPS10_15710	metal-binding protein			R
FPS10_15715	multicopper oxidase family protein	GO:0005507,GO:0016491,GO:0055114		Q
FPS10_15720	cytochrome c			C
FPS10_15725	c-type cytochrome			P
FPS10_15730	peptidase		K07231	P
FPS10_15735	(2Fe-2S)-binding protein			P
FPS10_15740	bacterioferritin		K03594	P
FPS10_15745	c-type cytochrome	GO:0009055,GO:0020037		C
FPS10_15750	imelysin family protein		K07338	R
FPS10_15755	DUF1513 domain-containing protein		K09947	S
FPS10_15760	ATP-binding cassette domain-containing protein		K02062	H
FPS10_15765	thiamine/thiamine pyrophosphate ABC transporter permease ThiP		K02063	P
FPS10_15770	thiamine ABC transporter substrate binding subunit	GO:0005215,GO:0006810,GO:0015888,GO:0030288,GO:00309[illegible]5	K02064	H
FPS10_15775	BCCT family transporter		K03451	M
FPS10_15780	universal stress protein	GO:0006950		T
FPS10_15785	aminotransferase class V-fold PLP-dependent enzyme	GO:0003824,GO:0008152,GO:0008483,GO:0016740		E
FPS10_15790	DNA-directed RNA polymerase subunit beta'	GO:0003677,GO:0003899,GO:0006351,GO:0016740,GO:00167[illegible]9	K03046	K
FPS10_15795	DNA-directed RNA polymerase subunit beta	GO:0003677,GO:0003899,GO:0006351,GO:0016740,GO:0016779,GO:0032549	K03043	K
FPS10_15800	50S ribosomal protein L7/L12	GO:0003735,GO:0005622,GO:0005840,GO:0006412,GO:00305[illegible]9	K02935	J
FPS10_15805	50S ribosomal protein L10	GO:0003723,GO:0003735,GO:0005622,GO:0005840,GO:0006412,GO:0019843,GO:0030529,GO:0042254,GO:0070180	K02864	J
FPS10_15810	NAD(P)H-dependent oxidoreductase	GO:0016491,GO:0055114		R
FPS10_15815	lytic murein transglycosylase			M
FPS10_15820	50S ribosomal protein L1	GO:0000049,GO:0003723,GO:0003735,GO:0005840,GO:0006412,GO:0006417,GO:0015934,GO:0019843,GO:0030529	K02863	J
FPS10_15825	50S ribosomal protein L11		K02867	J
FPS10_15830	transcription termination/antitermination protein NusG	GO:0006351,GO:0006353,GO:0006354,GO:0006355,GO:0031564,GO:0032784	K02601	K
FPS10_15835	preprotein translocase subunit SecE	GO:0005622,GO:0005623,GO:0006605,GO:0006810,GO:0006886,GO:0009306,GO:0015031,GO:0015450,GO:0016020,GO:0016021,GO:0071806	K03073	U
FPS10_15840	1-phosphofructokinase	GO:0000166,GO:0005524,GO:0005975,GO:0008662,GO:0016301,GO:0016310,GO:0016740,GO:0016773,GO:0046835	K00882	G
FPS10_15845	DUF1217 domain-containing protein			
FPS10_15850	flagellar biosynthesis repressor FlbT		K06601	N
FPS10_15855	flagellar biosynthesis regulator FlaF	GO:0044781	K06602	N
FPS10_15860	flagellin	GO:0005198,GO:0005576,GO:0009288,GO:0071973	K02406	N
FPS10_15865	hypothetical protein			
FPS10_15870	flagellar biosynthesis protein FlgJ			
FPS10_15875	hypothetical protein			
FPS10_15880	hypothetical protein		K02389	N
FPS10_15885	2-polyprenylphenol 6-hydroxylase	GO:0000166,GO:0005524,GO:0005886,GO:0006744,GO:0016020,GO:0016021,GO:0016301,GO:0016310,GO:0016740	K03688	R
FPS10_15890	bifunctional demethylmenaquinone methyltransferase/2-methoxy-6-polyprenyl-1%2C4-benzoquinol methylase UbiE	GO:0006744,GO:0008168,GO:0008425,GO:0009060,GO:0009234,GO:0016740,GO:0032259	K03183	H
FPS10_15895	bifunctional DNA-formamidopyrimidine glycosylase/DNA-(apurinic or apyrimidinic site) lyase	GO:0003676,GO:0003677,GO:0003684,GO:0003824,GO:0003906,GO:0006281,GO:0006284,GO:0006289,GO:0006974,GO:0008152,GO:0008270,GO:0008534,GO:0016787,GO:0016798,GO:0016799,GO:0016829,GO:0046872	K10563	L
FPS10_15900	enoyl-CoA hydratase	GO:0003824,GO:0004300,GO:0008152,GO:0016829,GO:00168[illegible]3	K01715	I
FPS10_15905	30S ribosomal protein S20		K02968	J
FPS10_15910	chromosomal replication initiator protein DnaA	GO:0000166,GO:0003677,GO:0003688,GO:0005524,GO:0005737,GO:0006260,GO:0006270,GO:0006275,GO:0043565	K02313	L
FPS10_15915	DNA polymerase III subunit beta		K02338	L
FPS10_15920	DNA replication/repair protein RecF		K03629	L
FPS10_15925	DNA topoisomerase (ATP-hydrolyzing) subunit B	GO:0000166,GO:0000287,GO:0003677,GO:0003916,GO:0003918,GO:0005524,GO:0005694,GO:0005737,GO:0006261,GO:0006265,GO:0016853,GO:0046872	K02470	L
FPS10_15930	HNH endonuclease			V
FPS10_15935	ImmA/IrrE family metallo-endopeptidase			
FPS10_15940	N-6 DNA methylase			V
FPS10_15945	beta-lactamase family protein		K01286	V

FPS10_15950	hypothetical protein			
FPS10_15955	hypothetical protein			
FPS10_15960	hypothetical protein			
FPS10_15965	DUF3955 domain-containing protein			
FPS10_15970	hypothetical protein			
FPS10_15975	alpha/beta hydrolase			
FPS10_15980	recombinase family protein			L
FPS10_15985	site-specific integrase			
FPS10_15990	site-specific integrase			
FPS10_15995	helix-turn-helix domain-containing protein			
FPS10_16000	hypothetical protein			
FPS10_16005	alpha/beta hydrolase			S
FPS10_16010	biotin/lipoyl-binding protein			V
FPS10_16015	carbohydrate porin		K07267	
FPS10_16020	DUF126 domain-containing protein	GO:0016310,GO:0016772	K09128	S
FPS10_16025	DUF521 domain-containing protein			S
FPS10_16030	SDR family oxidoreductase		K00059	IQR
FPS10_16035	DUF120 domain-containing protein		K07732	KH
FPS10_16040	LLM class flavin-dependent oxidoreductase			C
FPS10_16045	helix-turn-helix domain-containing protein		K04033	K
FPS10_16050	hypothetical protein			
FPS10_16055	IS91 family transposase			
FPS10_16060	hypothetical protein			
FPS10_16065	DUF1254 domain-containing protein			S
FPS10_16070	IS110 family transposase	GO:0003677,GO:0004803,GO:0006313		L
FPS10_16075	arylsulfatase	GO:0003824,GO:0008152,GO:0008484	K01130	P
FPS10_16080	hypothetical protein			
FPS10_16085	DUF2092 domain-containing protein			S
FPS10_16090	hypothetical protein			
FPS10_16095	helix-turn-helix transcriptional regulator			K
FPS10_16100	phenylacetate--CoA ligase family protein			H
FPS10_16105	inorganic phosphate transporter	GO:0005315,GO:0006817,GO:0016020,GO:0016021,GO:0055085	K03306	P
FPS10_16110	Na/Pi cotransporter family protein	GO:0015321,GO:0016020,GO:0016021,GO:0044341	K03324	P
FPS10_16115	hypothetical protein			
FPS10_16125	IS6 family transposase		K07498	L
FPS10_16130	IS110 family transposase			
FPS10_16135	hypothetical protein			
FPS10_16140	hypothetical protein			
FPS10_16145	hypothetical protein			
FPS10_16150	DUF3604 domain-containing protein			
FPS10_16155	AraC family transcriptional regulator			K
FPS10_16160	DUF3604 domain-containing protein			
FPS10_16165	DUF1330 domain-containing protein			S
FPS10_16180	hypothetical protein			
FPS10_16185				
FPS10_16190	hypothetical protein			R
FPS10_16195	DNA methylase N-4			L
FPS10_16200	DUF3489 domain-containing protein			
FPS10_16205	DUF2924 domain-containing protein			
FPS10_16210	recombinase family protein	GO:0000150,GO:0003677,GO:0006310		L
FPS10_16215	protein-methionine-sulfoxide reductase heme-binding subunit MsrQ	GO:0016020,GO:0016021	K17247	S
FPS10_16220	hypothetical protein			
FPS10_16225	protein-methionine-sulfoxide reductase catalytic subunit MsrP	GO:0016491,GO:0016667,GO:0016672,GO:0030091,GO:0042128,GO:0043546,GO:0046872,GO:0055114	K07147	R
FPS10_16230	ATP-dependent chaperone ClpB	GO:0000166,GO:0005524,GO:0005737,GO:0009408,GO:0016485,GO:0019538	K03695	O
FPS10_16235	orotidine-5'-phosphate decarboxylase		K01591	F
FPS10_16240	hypothetical protein	GO:0016020,GO:0016021		OU
FPS10_16245	SPFH/Band 7/PHB domain protein	GO:0016020,GO:0016021		O
FPS10_16250	NUDIX domain-containing protein		K03574	F
FPS10_16255	ATP-binding cassette domain-containing protein	GO:0000166,GO:0005524,GO:0006810,GO:0008152,GO:0016020,GO:0016021,GO:0016887,GO:0042626,GO:0055085	K06147	V
FPS10_16260	acyl-CoA synthetase	GO:0003824,GO:0004321,GO:0008152,GO:0016740,GO:0016746	K00666	IQ
FPS10_16265	MFS transporter	GO:0016020,GO:0016021,GO:0055085		G
FPS10_16270	hypothetical protein			

FPS10_16275	Asp-tRNA(Asn)/Glu-tRNA(Gln) amidotransferase subunit GatC		K02435	J
FPS10_16280	Asp-tRNA(Asn)/Glu-tRNA(Gln) amidotransferase subunit GatA	GO:0000166,GO:0005524,GO:0006412,GO:0016740,GO:0016874,GO:0016884,GO:0050567	K02433	J
FPS10_16285	hypothetical protein			
FPS10_16290	N-acetylmuramoyl-L-alanine amidase			V
FPS10_16295				
FPS10_16300	50S ribosomal protein L33	GO:0003735,GO:0005622,GO:0005840,GO:0006412,GO:00305[illegible]	K02913	J
FPS10_16305	Bax inhibitor-1/YccA family protein	GO:0016020,GO:0016021	K06890	R
FPS10_16310	DUF1127 domain-containing protein			S
FPS10_16315	LysR family transcriptional regulator			K
FPS10_16320	NADPH:quinone oxidoreductase family protein	GO:0008270,GO:0016491,GO:0055114	K00344	CR
FPS10_16325	helix-turn-helix transcriptional regulator	GO:0003677,GO:0043565		K
FPS10_16330	inositol monophosphatase family protein			G
FPS10_16335	8-oxoguanine deaminase	GO:0008152,GO:0016787,GO:0016810,GO:0018763		FR
FPS10_16340	CAP domain-containing protein			S
FPS10_16345	guanine deaminase	GO:0006147,GO:0008270,GO:0008892,GO:0016787,GO:00168[illegible]	K01487	FR
FPS10_16350	magnesium transporter	GO:0005886,GO:0006810,GO:0006812,GO:0008324,GO:0015095,GO:0015693,GO:0016020,GO:0016021,GO:0046872,GO:0098655,GO:1903830	K06213	P
FPS10_16355	5-formyltetrahydrofolate cyclo-ligase	GO:0000166,GO:0005524,GO:0008152,GO:0016874,GO:0030272,GO:0046872	K01934	H
FPS10_16360	YmdB family metallophosphoesterase		K09769	S
FPS10_16365	SLC13 family permease			P
FPS10_16370	YebC/PmpR family DNA-binding transcriptional regulator	GO:0003677,GO:0005737,GO:0006351,GO:0006355		S
FPS10_16375	cytochrome c			C
FPS10_16380	amino acid transporter		K06895	R
FPS10_16385	DMT family transporter	GO:0016020,GO:0016021		GER
FPS10_16390	N-formylglutamate amidohydrolase	GO:0008745,GO:0009253,GO:0016787,GO:0050415		E
FPS10_16395				
FPS10_16400	50S ribosomal protein L36	GO:0003735,GO:0005622,GO:0005840,GO:0006412,GO:00305[illegible]	K02919	J
FPS10_16405	anaerobic sulfatase maturase	GO:0003824,GO:0008152,GO:0016491,GO:0046872,GO:0051536,GO:0051539,GO:0055114	K06871	R
FPS10_16410	Lrp/AsnC family transcriptional regulator	GO:0003677,GO:0003700,GO:0005622,GO:0006351,GO:0006355,GO:0043565		K
FPS10_16415	alanine dehydrogenase	GO:0000286,GO:0016491,GO:0042853,GO:0055114	K00259	E
FPS10_16420	peptidoglycan-binding protein			
FPS10_16425	phenylacetate--CoA ligase family protein	GO:0003824,GO:0008152,GO:0016874	K01912	H
FPS10_16430	acetyl-CoA carboxylase carboxyltransferase subunit alpha	GO:0000166,GO:0003989,GO:0005524,GO:0005737,GO:0006629,GO:0006631,GO:0006633,GO:0009317,GO:0016740,GO:0016874,GO:2001295	K01962	I
FPS10_16435	CoA ester lyase	GO:0003824,GO:0008152,GO:0016829,GO:0046872,GO:00500[illegible]	K08691	G
FPS10_16440	hypothetical protein			S
FPS10_16445	ATP-binding cassette domain-containing protein		K09812	D
FPS10_16450	cell division protein FtsX	GO:0016020,GO:0016021	K09811	D
FPS10_16455	1-acyl-sn-glycerol-3-phosphate acyltransferase	GO:0008152,GO:0016020,GO:0016021,GO:0016740,GO:00167[illegible]	K00655	I
FPS10_16460	class I SAM-dependent methyltransferase			QR
FPS10_16465	sulfatase-like hydrolase/transferase	GO:0003824,GO:0008152,GO:0008484,GO:0016787		P
FPS10_16470	carbohydrate ABC transporter permease	GO:0005886,GO:0006810,GO:0016020,GO:0016021		G
FPS10_16475	sugar ABC transporter permease		K17245	G
FPS10_16480	extracellular solute-binding protein		K02027	G
FPS10_16485	ACT domain-containing protein		K09964	S
FPS10_16490	LysR family transcriptional regulator			K
FPS10_16495	rhodanese-like domain-containing protein			P
FPS10_16500	ABC transporter ATP-binding protein	GO:0005524,GO:0008152,GO:0016887	K01996	E
FPS10_16505	ABC transporter substrate-binding protein		K01999	E
FPS10_16510	branched-chain amino acid ABC transporter permease		K01998	E
FPS10_16515	hypothetical protein	GO:0016020,GO:0016021		
FPS10_16520	branched-chain amino acid ABC transporter permease	GO:0005215,GO:0005886,GO:0006810,GO:0016020,GO:00160[illegible]	K01997	E
FPS10_16525	ABC transporter ATP-binding protein	GO:0005524,GO:0008152,GO:0016887	K01995	E
FPS10_16530	long-chain fatty acid--CoA ligase		K01897	I
FPS10_16535	L%2CD-transpeptidase	GO:0008152,GO:0016740		S
FPS10_16540	peroxiredoxin	GO:0016209,GO:0016491,GO:0051920,GO:0055114,GO:0098[illegible]		O
FPS10_16545	aldehyde dehydrogenase family protein	GO:0008152,GO:0016491,GO:0016620,GO:0055114	K00128	C
FPS10_16550	urate hydroxylase PuuD	GO:0016020,GO:0016021		S
FPS10_16555	allantoicase	GO:0000256,GO:0004037,GO:0006144,GO:0016787	K01477	F
FPS10_16560	glutathione gamma-glutamylcysteinyltransferase			

FPS10_16565	preprotein translocase subunit SecY	GO:0005622,GO:0005886,GO:0006605,GO:0006810,GO:0015031,GO:0016020,GO:0016021,GO:0043952,GO:0065002	K03076	U
FPS10_16570	50S ribosomal protein L15		K02876	J
FPS10_16575	hypothetical protein			
FPS10_16580	glycerophosphodiester phosphodiesterase		K01126	C
FPS10_16585	50S ribosomal protein L30		K02907	J
FPS10_16590	30S ribosomal protein S5	GO:0003723,GO:0003735,GO:0005840,GO:0006412,GO:0015935,GO:0019843,GO:0030529	K02988	J
FPS10_16595	50S ribosomal protein L18	GO:0003723,GO:0003735,GO:0005622,GO:0005840,GO:0006412,GO:0019843,GO:0030529	K02881	J
FPS10_16600	50S ribosomal protein L6		K02933	J
FPS10_16605	30S ribosomal protein S8	GO:0003723,GO:0003735,GO:0005840,GO:0006412,GO:0019843,GO:0030529	K02994	J
FPS10_16610	30S ribosomal protein S14	GO:0003723,GO:0003735,GO:0005622,GO:0005840,GO:0006412,GO:0019843,GO:0030529	K02954	J
FPS10_16615	50S ribosomal protein L5	GO:0000049,GO:0003723,GO:0003735,GO:0005622,GO:0005840,GO:0006412,GO:0019843,GO:0030529	K02931	J
FPS10_16620	50S ribosomal protein L24	GO:0003723,GO:0003735,GO:0005622,GO:0005840,GO:0006412,GO:0019843,GO:0030529	K02895	J
FPS10_16625	50S ribosomal protein L14	GO:0003723,GO:0003735,GO:0005840,GO:0006412,GO:0015934,GO:0019843,GO:0030529	K02874	J
FPS10_16630	30S ribosomal protein S17	GO:0003723,GO:0003735,GO:0005622,GO:0005840,GO:0006412,GO:0019843,GO:0030529	K02961	J
FPS10_16635	50S ribosomal protein L29	GO:0003735,GO:0005622,GO:0005840,GO:0006412,GO:0030529	K02904	J
FPS10_16640	hypothetical protein			
FPS10_16645	thermonuclease family protein			L
FPS10_16650	type II toxin-antitoxin system Phd/YefM family antitoxin			
FPS10_16655	hypothetical protein			
FPS10_16660	50S ribosomal protein L16		K02878	J
FPS10_16665	30S ribosomal protein S3		K02982	J
FPS10_16670	50S ribosomal protein L22		K02890	J
FPS10_16675	30S ribosomal protein S19	GO:0003723,GO:0003735,GO:0005840,GO:0006412,GO:0015935,GO:0019843,GO:0030529	K02965	J
FPS10_16680	50S ribosomal protein L2	GO:0003723,GO:0003735,GO:0005622,GO:0005840,GO:0006412,GO:0015934,GO:0016740,GO:0019843,GO:0030529	K02886	J
FPS10_16685	hypothetical protein			
FPS10_16690	50S ribosomal protein L23		K02892	J
FPS10_16695	50S ribosomal protein L4		K02926	J
FPS10_16700	50S ribosomal protein L3	GO:0003723,GO:0003735,GO:0005622,GO:0005840,GO:0006412,GO:0019843,GO:0030529	K02906	J
FPS10_16705	30S ribosomal protein S10	GO:0000049,GO:0003735,GO:0005840,GO:0006412,GO:0030529	K02946	J
FPS10_16710	IS5/IS1182 family transposase			
FPS10_16715	IS1182 family transposase			L
FPS10_16720	carboxylate-amine ligase	GO:0000166,GO:0004357,GO:0005524,GO:0016874,GO:0016879,GO:0042398	K06048	S
FPS10_16725	hypothetical protein		K07214	P
FPS10_16730	hypothetical protein			S
FPS10_16735	ATP-grasp domain-containing protein			I
FPS10_16745	porin			
FPS10_16750	pyruvate dehydrogenase complex dihydrolipoamide acetyltransferase		K00627	C
FPS10_16755	pyruvate dehydrogenase complex E1 component subunit beta	GO:0003824,GO:0004739,GO:0008152,GO:0016491,GO:0055114	K00162	C
FPS10_16760	pyruvate dehydrogenase (acetyl-transferring) E1 component subunit alpha	GO:0004739,GO:0006086,GO:0008152,GO:0016491,GO:0016624,GO:0043231,GO:0055114	K00161	C
FPS10_16765	DUF3604 domain-containing protein			
FPS10_16770	septum formation initiator family protein	GO:0003674,GO:0005575,GO:0007049,GO:0008150,GO:0016020,GO:0016021		D
FPS10_16775	alpha-D-glucose phosphate-specific phosphoglucomutase		K01835	G
FPS10_16780	4-alpha-glucanotransferase		K00705	G
FPS10_16785	glycogen debranching protein GlgX		K01214	G
FPS10_16790	glycogen synthase GlgA	GO:0004373,GO:0005978,GO:0009011,GO:0016740,GO:0016757,GO:0033201	K00703	G
FPS10_16795	glucose-1-phosphate adenylyltransferase	GO:0000166,GO:0005524,GO:0005975,GO:0005977,GO:0005978,GO:0008878,GO:0009058,GO:0016740,GO:0016779	K00975	G
FPS10_16800	1%2C4-alpha-glucan branching protein GlgB	GO:0003824,GO:0003844,GO:0004553,GO:0005975,GO:0005977,GO:0005978,GO:0016740,GO:0016757,GO:0043169	K00700	G
FPS10_16805	glycogen/starch/alpha-glucan phosphorylase	GO:0004645,GO:0005975,GO:0008184,GO:0016740,GO:0016757,GO:0030170	K00688	G
FPS10_16810	oxidoreductase			R

FPS10_16815	flavin reductase			R
FPS10_16820	YtoQ family protein	GO:0008152,GO:0016740		
FPS10_16825	acetolactate synthase large subunit	GO:0000287,GO:0003824,GO:0008152,GO:0030976	K01652	EH
FPS10_16830	GAF domain-containing protein			T
FPS10_16835	hypothetical protein			
FPS10_16845	alpha-D-ribose 1-methylphosphonate 5-triphosphate diphosphatase	GO:0016810,GO:0019700	K06162	P
FPS10_16850	hypothetical protein	GO:0000166,GO:0005524,GO:0008152,GO:0016829,GO:0016887		P
FPS10_16855	magnesium transporter		K03284	P
FPS10_16860	DUF1045 domain-containing protein			
FPS10_16865	alpha-D-ribose 1-methylphosphonate 5-triphosphate diphosphatase	GO:0016810,GO:0019700	K06162	P
FPS10_16870	hypothetical protein			
FPS10_16875	bifunctional enoyl-CoA hydratase/phosphate acetyltransferase	GO:0008152,GO:0016740,GO:0016746,GO:0050182		C
FPS10_16880	aldo/keto reductase			C
FPS10_16885	YeeE/YedE family protein	GO:0016020,GO:0016021	K07112	
FPS10_16890	helix-turn-helix transcriptional regulator			K
FPS10_16895	thioredoxin family protein			O
FPS10_16900	cytochrome c biogenesis protein CcdA	GO:0016020,GO:0016021,GO:0017004,GO:0055114	K06196	O
FPS10_16905	thioredoxin			O
FPS10_16910	sulfur oxidation c-type cytochrome SoxX		K17223	C
FPS10_16915	thiosulfate oxidation carrier protein SoxY		K17226	S
FPS10_16920	thiosulfate oxidation carrier complex protein SoxZ		K17227	
FPS10_16925	sulfur oxidation c-type cytochrome SoxA		K17222	C
FPS10_16930	thiosulfohydrolase SoxB	GO:0000166,GO:0009166,GO:0016787	K17224	F
FPS10_16935	sulfite dehydrogenase	GO:0016491,GO:0030151,GO:0042128,GO:0055114	K17225	R
FPS10_16940	c-type cytochrome		K22622	C
FPS10_16945	c-type cytochrome	GO:0009055,GO:0020037	K08738	C
FPS10_16950	NAD(P)/FAD-dependent oxidoreductase	GO:0016491,GO:0050660,GO:0055114		R
FPS10_16955	DUF302 domain-containing protein			S
FPS10_16965	hypothetical protein			
FPS10_16975	hypothetical protein			
FPS10_16980	response regulator		K03413	T
FPS10_16985	hypothetical protein		K00575	NT
FPS10_16990	purine-binding chemotaxis protein CheW		K03408	NT
FPS10_16995	chemotaxis protein CheA		K03407	NT
FPS10_17000	response regulator	GO:0000160,GO:0005622	K03413	TK
FPS10_17005	STAS domain-containing protein		K03409	T
FPS10_17010	glucokinase	GO:0004340,GO:0005524,GO:0006096,GO:0016301,GO:0016310,GO:0051156	K00845	G
FPS10_17015	beta-glucosidase	GO:0004553,GO:0005975,GO:0008152,GO:0008422,GO:0016787,GO:0016798,GO:0030245	K05350	G
FPS10_17020	LacI family DNA-binding transcriptional regulator		K02529	K
FPS10_17025	carbohydrate ABC transporter substrate-binding protein	GO:0005215,GO:0006810	K10232	G
FPS10_17030	sugar ABC transporter permease		K10233	G
FPS10_17035	carbohydrate ABC transporter permease	GO:0005886,GO:0006810,GO:0016020,GO:0016021	K10234	G
FPS10_17040	DUF3459 domain-containing protein		K01187	G
FPS10_17045	sn-glycerol-3-phosphate ABC transporter ATP-binding protein UgpC	GO:0000166,GO:0005215,GO:0005524,GO:0006810,GO:0008152,GO:0016820,GO:0016887,GO:0043190,GO:0055085	K10235	G
FPS10_17050	HpcH/HpaI aldolase/citrate lyase family protein	GO:0003824,GO:0008152,GO:0016829,GO:0046872	K02510	G
FPS10_17055	hypothetical protein			
FPS10_17060	IMP dehydrogenase		K00088	F
FPS10_17065	acetylornithine deacetylase	GO:0005737,GO:0006508,GO:0006526,GO:0008152,GO:0008237,GO:0008270,GO:0008652,GO:0008777,GO:0016787,GO:0046872,GO:0050897	K01438	E
FPS10_17070	M24 family metallopeptidase	GO:0008152,GO:0016787	K15783	E
FPS10_17075	ABC transporter substrate-binding protein	GO:0043190,GO:0055085	K02035	E
FPS10_17080	ABC transporter substrate-binding protein	GO:0043190,GO:0055085	K02035	E
FPS10_17085	ABC transporter permease	GO:0005886,GO:0006810,GO:0016020,GO:0016021	K02033	EP
FPS10_17090	ABC transporter permease	GO:0005886,GO:0006810,GO:0016020,GO:0016021	K02034	EP
FPS10_17095	ABC transporter ATP-binding protein	GO:0000166,GO:0005524,GO:0006810,GO:0008152,GO:0015833,GO:0016887	K02031	R
FPS10_17100	choline-sulfatase	GO:0003824,GO:0008152,GO:0008484	K01133	P
FPS10_17105	lytic transglycosylase domain-containing protein			M
FPS10_17110	single-stranded DNA-binding protein	GO:0003677,GO:0003697,GO:0006260,GO:0006281,GO:0006310,GO:0006974	K03111	L
FPS10_17115	RNA-binding transcriptional accessory protein	GO:0003676,GO:0003677,GO:0006139,GO:0006281	K06959	K

FPS10_17120	disulfide bond formation protein B	GO:0005886,GO:0015035,GO:0016020,GO:0016021,GO:0016491,GO:0055114		O
FPS10_17125	DedA family protein	GO:0016020,GO:0016021		S
FPS10_17130	hypothetical protein	GO:0016020,GO:0016021	K07054	R
FPS10_17135	flavin-dependent oxidoreductase	GO:0004497,GO:0016491,GO:0055114,GO:0071949	K20940	HC
FPS10_17140				
FPS10_17145	hypothetical protein			R
FPS10_17150	hypothetical protein			
FPS10_17155	regulatory protein NosR		K19339	C
FPS10_17160	TAT-dependent nitrous-oxide reductase	GO:0004129,GO:0005507,GO:0005509,GO:0016020,GO:0016491,GO:0050304,GO:0055114,GO:1902600	K00376	C
FPS10_17165	nitrous oxide reductase family maturation protein NosD		K07218	P
FPS10_17170	ABC transporter ATP-binding protein		K19340	V
FPS10_17175	ABC transporter permease subunit	GO:0016020,GO:0016021,GO:0019333	K19341	R
FPS10_17180	copper resistance protein CopZ		K19342	C
FPS10_17185	FAD:protein FMN transferase		K03734	H
FPS10_17190	hypothetical protein	GO:0016020,GO:0016021		
FPS10_17195	NnrS family protein	GO:0003674,GO:0016020,GO:0016021,GO:0019333	K07234	P
FPS10_17200	cytochrome c	GO:0009055,GO:0016020,GO:0016021,GO:0016491,GO:0016966,GO:0020037,GO:0055114	K02305	
FPS10_17205	nitric-oxide reductase large subunit	GO:0004129,GO:0005506,GO:0009055,GO:0009060,GO:0016020,GO:0016021,GO:0016491,GO:0016966,GO:0019333,GO:0020037,GO:0055114,GO:0070469,GO:1902600	K04561	P
FPS10_17210	CbbQ/NirQ/NorQ/GpvN family protein		K04748	R
FPS10_17215	VWA domain-containing protein		K02448	P
FPS10_17220	cytochrome c oxidase subunit 3 family protein		K02164	C
FPS10_17225	nitric oxide reductase F protein			
FPS10_17230	hypothetical protein			
FPS10_17235	Crp/Fnr family transcriptional regulator	GO:0003677,GO:0006351,GO:0006355,GO:0008150		T
FPS10_17240	AraC family transcriptional regulator			K
FPS10_17245	hypothetical protein			
FPS10_17250	hypothetical protein			
FPS10_17255	carbohydrate porin		K07267	M
FPS10_17260	acetyl-CoA hydrolase/transferase family protein		K18118	C
FPS10_17265	HAMP domain-containing protein		K03406	NT
FPS10_17270	tetratricopeptide repeat protein			C
FPS10_17275	Crp/Fnr family transcriptional regulator		K10914	T
FPS10_17280	arylsulfatase		K01130	P
FPS10_17285	arylsulfatase	GO:0003824,GO:0008152,GO:0008484	K01130	P
FPS10_17290	haloacid dehalogenase-like hydrolase			
FPS10_17295	MoxR family ATPase	GO:0005524,GO:0008152,GO:0016887	K03924	R
FPS10_17300	DUF58 domain-containing protein			R
FPS10_17305	DUF4381 domain-containing protein			
FPS10_17310	VWA domain-containing protein	GO:0016020,GO:0016021	K07114	H
FPS10_17315	VWA domain-containing protein	GO:0016020,GO:0016021	K07114	
FPS10_17320	protein BatD			
FPS10_17325	tRNA (N6-threonylcarbamoyladenosine(37)-N6)-methyltransferase TrmO			S
FPS10_17335	hypothetical protein			
FPS10_17340	FAD-dependent oxidoreductase	GO:0016491,GO:0055114	K00315	E
FPS10_17345	transcription-repair coupling factor	GO:0000166,GO:0000716,GO:0003676,GO:0003677,GO:0003684,GO:0004386,GO:0005524,GO:0005737,GO:0006281,GO:0006355,GO:0006974,GO:0016787	K03723	LK
FPS10_17350	hypothetical protein			
FPS10_17355	multidrug effflux MFS transporter		K07552	G
FPS10_17360	DsbA family oxidoreductase	GO:0015035,GO:0055114		Q
FPS10_17365	acyl--CoA ligase	GO:0003824,GO:0008152,GO:0016874		IQ
FPS10_17370	XRE family transcriptional regulator			K
FPS10_17375	hypothetical protein			
FPS10_17380	TetR/AcrR family transcriptional regulator	GO:0003677,GO:0006351,GO:0006355		K
FPS10_17385	polyphosphate kinase 2		K22468	S
FPS10_17390	alpha/beta hydrolase			R
FPS10_17395	homoserine O-succinyltransferase		K00651	E
FPS10_17400	ATPase			
FPS10_17405	efflux RND transporter permease subunit			V
FPS10_17410	efflux RND transporter periplasmic adaptor subunit			M

FPS10_17415	RsmB/NOP family class I SAM-dependent RNA methyltransferase	GO:0008168,GO:0032259	K03500	J
FPS10_17420	response regulator	GO:0000155,GO:0000160,GO:0005622,GO:0007165,GO:0016020,GO:0016021,GO:0016301,GO:0016310,GO:0016772,GO:0023014	K13587	T
FPS10_17425	recombinase RecA	GO:0000166,GO:0003677,GO:0003684,GO:0003697,GO:0005524,GO:0005737,GO:0006259,GO:0006281,GO:0006310,GO:0006974,GO:0008094,GO:0009432	K03553	L
FPS10_17430	alanine--tRNA ligase	GO:0000049,GO:0000166,GO:0003676,GO:0003723,GO:0004812,GO:0004813,GO:0005524,GO:0005737,GO:0006412,GO:0006419,GO:0008270,GO:0016874,GO:0016876,GO:0043039,GO:0046872	K01872	J
FPS10_17435	DUF1330 domain-containing protein			S
FPS10_17440	hypothetical protein			
FPS10_17445	translational GTPase TypA	GO:0000166,GO:0003924,GO:0005525,GO:0008152	K06207	T
FPS10_17450	tyrosine--tRNA ligase	GO:0000166,GO:0003723,GO:0004812,GO:0004831,GO:0005524,GO:0005737,GO:0006412,GO:0006418,GO:0006437,GO:0016874	K01866	J
FPS10_17455	anhydro-N-acetylmuramic acid kinase	GO:0000166,GO:0005524,GO:0005975,GO:0006040,GO:0009234,GO:0016301,GO:0016310,GO:0016740,GO:0016773,GO:0097175	K09001	O
FPS10_17460	cupin domain-containing protein		K09705	S
FPS10_17465	NUDIX hydrolase	GO:0008152,GO:0016787		LR
FPS10_17470	phosphopyruvate hydratase	GO:0000015,GO:0000287,GO:0004634,GO:0005576,GO:0005737,GO:0006096,GO:0009986,GO:0016829,GO:0046872	K01689	G
FPS10_17475	DMT family transporter	GO:0016020,GO:0016021		GER
FPS10_17480	transcriptional repressor	GO:0003677,GO:0003700,GO:0006355,GO:0046872	K03711	P
FPS10_17485	J domain-containing protein			O
FPS10_17490	MoxR family ATPase			R
FPS10_17495	RNA polymerase-binding protein DksA	GO:0005737,GO:0008270,GO:0010468,GO:0046872	K06204	T
FPS10_17500	monooxygenase	GO:0016491,GO:0055114,GO:0071949	K00480	HC
FPS10_17505	site-specific tyrosine recombinase XerD	GO:0003677,GO:0005737,GO:0006310,GO:0006313,GO:0007049,GO:0007059,GO:0009037,GO:0015074,GO:0051301	K04763	L
FPS10_17510	hypothetical protein			
FPS10_17515	shikimate kinase	GO:0000166,GO:0000287,GO:0004765,GO:0005524,GO:0005737,GO:0008652,GO:0009073,GO:0009423,GO:0016301,GO:0016310,GO:0016740,GO:0046872	K00891	E
FPS10_17520	3-dehydroquinate synthase	GO:0003856,GO:0005737,GO:0008652,GO:0009073,GO:0009423,GO:0016829,GO:0016838	K01735	E
FPS10_17525	aldehyde dehydrogenase family protein	GO:0008152,GO:0016491,GO:0016620,GO:0055114	K00128	C
FPS10_17530	Hsp20 family protein		K04080	O
FPS10_17535	trypsin-like serine protease			E
FPS10_17540	bifunctional proline dehydrogenase/L-glutamate gamma-semialdehyde dehydrogenase PutA	GO:0003677,GO:0003700,GO:0003842,GO:0004657,GO:0006351,GO:0006355,GO:0006560,GO:0006561,GO:0008152,GO:0010133,GO:0016491,GO:0016620,GO:0055114	K13821	E
FPS10_17545	winged helix-turn-helix transcriptional regulator		K03719	K
FPS10_17550	hypothetical protein			
FPS10_17555	LacI family transcriptional regulator			K
FPS10_17560	tripartite tricarboxylate transporter substrate binding protein	GO:0030288		S
FPS10_17565	tripartite tricarboxylate transporter TctB family protein			
FPS10_17570	tripartite tricarboxylate transporter permease	GO:0016020,GO:0016021		S
FPS10_17575	aldolase	GO:0003824,GO:0008152,GO:0046872	K02510	G
FPS10_17580	ester cyclase			R
FPS10_17585	DUF882 domain-containing protein			S
FPS10_17590	L%2CD-transpeptidase family protein	GO:0008152,GO:0016740	K21470	S
FPS10_17595	UDP-3-O-(3-hydroxymyristoyl)glucosamine N-acyltransferase	GO:0006629,GO:0009245,GO:0016410,GO:0016740,GO:0016746,GO:0016747	K02536	M
FPS10_17600	acyl carrier protein		K02078	IQ
FPS10_17605	beta-ketoacyl-[acyl-carrier-protein] synthase family protein	GO:0003824,GO:0008152,GO:0016740	K14660	IQ
FPS10_17610	motif:HMM:PF06776.10			R
FPS10_17615	DUF853 family protein		K06915	R
FPS10_17620	HlyC/CorC family transporter			P
FPS10_17625	hypothetical protein			
FPS10_17630				
FPS10_17635	trigger factor		K03545	O
FPS10_17640	GNAT family N-acetyltransferase	GO:0008080,GO:0008152,GO:0016740		E
FPS10_17645	hypothetical protein			
FPS10_17650	aldo/keto reductase			C
FPS10_17655	50S ribosomal protein L9	GO:0003723,GO:0003735,GO:0005622,GO:0005840,GO:0006412,GO:0019843,GO:0030529	K02939	J
FPS10_17660	30S ribosomal protein S18	GO:0003723,GO:0003735,GO:0005622,GO:0005840,GO:0006412,GO:0019843,GO:0030529	K02963	J

FPS10_17665	30S ribosomal protein S6	GO:0003723,GO:0003735,GO:0005840,GO:0006412,GO:0019843,GO:0030529	K02990	J
FPS10_17670	hypothetical protein			
FPS10_17675	ACP S-malonyltransferase	GO:0003824,GO:0004314,GO:0008152,GO:0016740,GO:0016746	K00645	I
FPS10_17680	calcium/sodium antiporter		K07301	P
FPS10_17685	acetolactate synthase 3 large subunit	GO:0000287,GO:0003824,GO:0003984,GO:0008652,GO:0009082,GO:0009097,GO:0009099,GO:0016740,GO:0030976,GO:0046872,GO:0050660	K01652	EH
FPS10_17690	MBL fold metallo-hydrolase			R
FPS10_17695	thioredoxin family protein	GO:0016209,GO:0016491,GO:0055114,GO:0098869		O
FPS10_17700	HAD-IA family hydrolase	GO:0005975,GO:0008152,GO:0008967,GO:0016311,GO:0016787,GO:0046295,GO:0046872	K01091	R
FPS10_17705	HAD-IA family hydrolase	GO:0008152,GO:0016787		R
FPS10_17710	hypothetical protein			
FPS10_17715	META domain-containing protein		K09914	S
FPS10_17720	Rrf2 family transcriptional regulator		K13771	K
FPS10_17725	thiol reductant ABC exporter subunit CydD	GO:0000166,GO:0005524,GO:0006810,GO:0008152,GO:0016020,GO:0016021,GO:0016887,GO:0042626,GO:0042883,GO:0055085	K16013	CO
FPS10_17730	ATP-binding cassette domain-containing protein	GO:0000166,GO:0005524,GO:0006810,GO:0008152,GO:0016020,GO:0016021,GO:0016887,GO:0042626,GO:0055085	K16012	CO
FPS10_17735	cytochrome bd-I ubiquinol oxidase subunit CydA		K00425	C
FPS10_17740	cytochrome d ubiquinol oxidase subunit II		K00426	C
FPS10_17745	cytochrome bd-I oxidase subunit CydX		K00424	
FPS10_17750	NAD(P)/FAD-dependent oxidoreductase	GO:0016491,GO:0055114	K17218	R
FPS10_17755	hypothetical protein	GO:0016020,GO:0016021		
FPS10_17760	C4-dicarboxylate ABC transporter		K03304	P
FPS10_17765	DUF1289 domain-containing protein			
FPS10_17770	hemin uptake protein HemP			P
FPS10_17775	peptidase	GO:0000502,GO:0004298,GO:0005839,GO:0006508,GO:0008233,GO:0051603	K07395	O
FPS10_17780	transglutaminase family protein			E
FPS10_17785	alpha-E domain-containing protein			S
FPS10_17790	circularly permuted type 2 ATP-grasp protein			S
FPS10_17795	D-tyrosyl-tRNA(Tyr) deacylase	GO:0005737,GO:0016787,GO:0016788,GO:0019478	K07560	J
FPS10_17800	carbohydrate kinase	GO:0004747,GO:0006014,GO:0008865,GO:0016301,GO:0016310,GO:0016740,GO:0016773,GO:0046835	K00847	G
FPS10_17805	primosomal protein N' (replication factor Y) - superfamily II helicase	GO:0016020,GO:0016021		
FPS10_17810	SPFH domain-containing protein			S
FPS10_17815	DUF2927 domain-containing protein			
FPS10_17820	toxic anion resistance protein			P
FPS10_17825	hypothetical protein			R
FPS10_17830	hypothetical protein			
FPS10_17835	sel1 repeat family protein			R
FPS10_17840	transporter			I
FPS10_17845	DMT family transporter	GO:0016020,GO:0016021		GER
FPS10_17850	glycosyltransferase family 8 protein			M
FPS10_17855	antibiotic biosynthesis monooxygenase			S
FPS10_17860	glutamine-hydrolyzing GMP synthase	GO:0000166,GO:0003922,GO:0005524,GO:0006164,GO:0006177,GO:0006541,GO:0016462,GO:0016874	K01951	F
FPS10_17865	LysR family transcriptional regulator	GO:0003677,GO:0003700,GO:0006351,GO:0006355		K
FPS10_17870	NAD(P)-dependent alcohol dehydrogenase			CR
FPS10_17875	trimethylamine methyltransferase family protein	GO:0008168,GO:0015948,GO:0016740,GO:0032259	K14083	H
FPS10_17880	hypothetical protein			P
FPS10_17885	hypothetical protein			
FPS10_17890	helix-turn-helix domain-containing protein	GO:0003674,GO:0005575,GO:0008150		
FPS10_17895	hypothetical protein			
FPS10_17900	peroxiredoxin	GO:0016491,GO:0055114		O
FPS10_17905	lipoyl synthase	GO:0003824,GO:0005737,GO:0009107,GO:0009249,GO:0016740,GO:0016783,GO:0016992,GO:0046872,GO:0051536,GO:0051539	K03644	H
FPS10_17910	c-type cytochrome			C
FPS10_17915	hypothetical protein			
FPS10_17920	hypoxanthine phosphoribosyltransferase		K00760	F
FPS10_17925	type II toxin-antitoxin system RatA family toxin		K18588	I
FPS10_17930	ammonium transporter	GO:0006810,GO:0008519,GO:0015696,GO:0016020,GO:0016021,GO:0072488	K03320	P
FPS10_17935	CinA family protein		K03743	R
FPS10_17940	hypothetical protein		K01095	

FPS10_17945	phosphatidylglycerophosphatase A		K01095	I
FPS10_17950	bifunctional 2-C-methyl-D-erythritol 4-phosphate cytidylyltransferase/2-C-methyl-D-erythritol 2%2C4-cyclodiphosphate synthase	GO:0003824,GO:0008152,GO:0008299,GO:0008685,GO:0016114,GO:0016740,GO:0016779,GO:0016829,GO:0019288,GO:0046872,GO:0050518	K12506	I
FPS10_17955	tRNA dihydrouridine synthase DusB	GO:0002943,GO:0003824,GO:0008033,GO:0016491,GO:0017150,GO:0050660,GO:0055114		J
FPS10_17960	PAS domain-containing sensor histidine kinase	GO:0000155,GO:0000160,GO:0000166,GO:0005524,GO:0005622,GO:0007165,GO:0016301,GO:0016310,GO:0016740,GO:0016772,GO:0023014	K07708	T
FPS10_17965	response regulator	GO:0000160,GO:0000166,GO:0003677,GO:0005524,GO:0005622,GO:0006351,GO:0006355,GO:0008134,GO:0043565	K07712	T
FPS10_17970	PAS domain-containing sensor histidine kinase		K13598	T
FPS10_17975	sigma-54-dependent Fis family transcriptional regulator		K13599	T
FPS10_17980	Trk system potassium transporter TrkA	GO:0005886,GO:0006813,GO:0008324,GO:0015079,GO:0071805,GO:0098655	K03499	P
FPS10_17985	TrkH family potassium uptake protein	GO:0006812,GO:0008324,GO:0016020,GO:0016021,GO:0055085,GO:0098655	K03498	P
FPS10_17990	RNA chaperone Hfq	GO:0003723,GO:0006355	K03666	R
FPS10_17995	GTPase HflX	GO:0000166,GO:0000287,GO:0003924,GO:0005525,GO:0005737,GO:0008152,GO:0046872	K03665	R
FPS10_18000	penicillin acylase family protein		K01434	R
FPS10_18005	NAD(P)-dependent oxidoreductase	GO:0004616,GO:0016491,GO:0051287,GO:0055114	K00020	I
FPS10_18010	hypothetical protein	GO:0016020,GO:0016021		
FPS10_18015	cytochrome P450	GO:0004497,GO:0005506,GO:0016491,GO:0016705,GO:0020037,GO:0046872,GO:0055114		Q
FPS10_18020	ribbon-helix-helix domain-containing protein			R
FPS10_18025	hypothetical protein			
FPS10_18030	DUF4169 family protein			
FPS10_18035	class II fumarate hydratase	GO:0003824,GO:0004333,GO:0005737,GO:0006099,GO:0006106,GO:0016829,GO:0045239	K01679	C
FPS10_18040	hypothetical protein		K09985	S
FPS10_18045	ester cyclase			
FPS10_18050				
FPS10_18055	rubrerythrin family protein	GO:0016020,GO:0016021,GO:0016491,GO:0046872,GO:00551[illegible]	K22737	S
FPS10_18060	damage-inducible protein DinB			S
FPS10_18065	oxidoreductase	GO:0008270,GO:0016491,GO:0055114	K19745	CR
FPS10_18070				
FPS10_18075				
FPS10_18080	imidazoleglycerol-phosphate dehydratase HisB	GO:0000105,GO:0004424,GO:0005737,GO:0008652,GO:00168[illegible]	K01693	E
FPS10_18085	imidazole glycerol phosphate synthase subunit HisH		K02501	E
FPS10_18090	aminotransferase class V-fold PLP-dependent enzyme			E
FPS10_18095	deoxyribodipyrimidine photo-lyase	GO:0003904,GO:0016829,GO:0018298	K01669	L
FPS10_18100	TrgA family protein			
FPS10_18105	NUDIX domain-containing protein			
FPS10_18110	cysteine synthase A	GO:0004124,GO:0006535,GO:0016740,GO:0016829	K01738	E
FPS10_18115	mechanosensitive ion channel family protein		K05802	M
FPS10_18120	alanyl-tRNA editing protein	GO:0000166,GO:0003676,GO:0004813,GO:0005524,GO:0006419,GO:0016876,GO:0043039	K07050	R
FPS10_18125				
FPS10_18130	site-specific integrase			L
FPS10_18135	hypothetical protein			
FPS10_18140	helix-turn-helix domain-containing protein			L
FPS10_18145	hypothetical protein			
FPS10_18150	hypothetical protein			
FPS10_18155	hypothetical protein			
FPS10_18160	DUF3427 domain-containing protein			
FPS10_18165	hypothetical protein			
FPS10_18170	hypothetical protein			
FPS10_18175	DUF3768 domain-containing protein			
FPS10_18180	hypothetical protein			
FPS10_18185	ATP-binding protein			
FPS10_18195	DUF87 domain-containing protein		K06915	R
FPS10_18200	phosphoadenosine phosphosulfate reductase family protein			EH
FPS10_18205	hypothetical protein			
FPS10_18210	hypothetical protein			
FPS10_18215	hypothetical protein			
FPS10_18220	hypothetical protein			
FPS10_18225	hypothetical protein			

FPS10_18230	hypothetical protein			
FPS10_18235	DUF1738 domain-containing protein			L
FPS10_18240	chromosome partitioning protein ParB		K03497	K
FPS10_18245	methylase			
FPS10_18250	virulence-associated protein E			
FPS10_18255	lytic transglycosylase domain-containing protein			M
FPS10_18260	hypothetical protein			
FPS10_18265	hypothetical protein			
FPS10_18270	AAA family ATPase		K03496	D
FPS10_18275	ParB/RepB/Spo0J family partition protein	GO:0003677	K03497	K
FPS10_18280	hypothetical protein			
FPS10_18285	tyrosine-type recombinase/integrase			L
FPS10_18290	thiolase domain-containing protein		K00626	I
FPS10_18295	acetyl-CoA C-acyltransferase	GO:0003824,GO:0008152,GO:0016740,GO:0016747	K00626	I
FPS10_18300	acyl--CoA ligase			IQ
FPS10_18305	SDR family NAD(P)-dependent oxidoreductase		K12405	IQR
FPS10_18310	AMP-binding protein		K00666	IQ
FPS10_18315	nuclear transport factor 2 family protein			R
FPS10_18320	SDR family NAD(P)-dependent oxidoreductase		K11168	IQR
FPS10_18325	TonB-dependent receptor			P
FPS10_18330	helix-turn-helix transcriptional regulator			K
FPS10_18335	DUF1295 domain-containing protein			S
FPS10_18340	9-cis-epoxycarotenoid dioxygenase			Q
FPS10_18345	phenylacetic acid degradation protein PaaN			C
FPS10_18350	thiolase family protein			I
FPS10_18355	nitronate monooxygenase		K00459	R
FPS10_18360	PaaI family thioesterase			Q
FPS10_18365	IS5 family transposase			L
FPS10_18370	hypothetical protein			
FPS10_18375	transposase		K07483	
FPS10_18380	IS66 family insertion sequence element accessory protein TnpB		K07484	L
FPS10_18385	transposase		K07484	L
FPS10_18395	flagellar type III secretion system pore protein FliP	GO:0005886,GO:0006810,GO:0008152,GO:0009306,GO:00094[illegible]5,GO:0015031,GO:0016020,GO:0016021,GO:0016787,GO:0044781	K02419	NU
FPS10_18400	FliM/FliN family flagellar motor switch protein	GO:0003774,GO:0006935,GO:0008152,GO:0009425,GO:00719[illegible]3	K02417	NU
FPS10_18405	hypothetical protein		K02411	NU
FPS10_18410	flagellar M-ring protein FliF		K02409	NU
FPS10_18415	queuosine precursor transporter	GO:0016020,GO:0016021	K09125	S
FPS10_18420	aminoacyl-tRNA hydrolase	GO:0003747,GO:0006415	K15034	J
FPS10_18425	nicotinate phosphoribosyltransferase	GO:0004514,GO:0004516,GO:0009435,GO:0016740,GO:0016757,GO:0016874,GO:0019357,GO:0019363	K00763	H
FPS10_18430	PAS domain S-box protein			T
FPS10_18435	bifunctional nicotinamidase/pyrazinamidase	GO:0003824,GO:0008152,GO:0008936,GO:0016787	K08281	Q
FPS10_18440	sugar transporter		K01991	M
FPS10_18445	YjbF family lipoprotein			
FPS10_18450	YjbH domain-containing protein			
FPS10_18455	sugar transferase			M
FPS10_18460	serine/threonine protein phosphatase		K07313	
FPS10_18465	ferredoxin-type protein NapF		K02572	C
FPS10_18470	nitrate reductase		K02570	P
FPS10_18475	nitrate reductase catalytic subunit NapA	GO:0005506,GO:0005576,GO:0006777,GO:0008940,GO:0009055,GO:0016491,GO:0030151,GO:0042128,GO:0042597,GO:0046872,GO:0051536,GO:0051539,GO:0055114	K02567	C
FPS10_18480	ferredoxin-type protein NapG		K02573	C
FPS10_18485	quinol dehydrogenase ferredoxin subunit NapH		K02574	C
FPS10_18490	nitrate reductase cytochrome c-type subunit	GO:0042597,GO:0046872,GO:0055114	K02568	C
FPS10_18495	4Fe-4S ferredoxin		K02569	C
FPS10_18500	hypothetical protein	GO:0016020,GO:0016021		
FPS10_18505	Crp/Fnr family transcriptional regulator	GO:0003677,GO:0006351,GO:0006355,GO:0008150		T
FPS10_18510	LacI family DNA-binding transcriptional regulator		K06145	K
FPS10_18515	four-carbon acid sugar kinase family protein		K21948	S
FPS10_18520	aldolase		K22130	G
FPS10_18525	TIM barrel protein	GO:0008152,GO:0008903,GO:0016853	K22131	G
FPS10_18530	response regulator transcription factor	GO:0000160,GO:0003677,GO:0005622,GO:0006351,GO:00063[illegible]5	K07774	TK

FPS10_18535	sensor histidine kinase	GO:0000155,GO:0000160,GO:0000166,GO:0004871,GO:0005524,GO:0005622,GO:0007165,GO:0016020,GO:0016021,GO:0016301,GO:0016310,GO:0016740,GO:0016772,GO:0023014	K07649	T
FPS10_18540	ABC transporter substrate-binding protein	GO:0003674,GO:0005575,GO:0008150	K02012	P
FPS10_18545	tripartite tricarboxylate transporter substrate binding protein		K07795	S
FPS10_18550	tripartite tricarboxylate transporter TctB family protein		K07794	
FPS10_18555	tripartite tricarboxylate transporter permease	GO:0016020,GO:0016021	K07793	S
FPS10_18560	DUF1446 domain-containing protein			
FPS10_18565	DUF4387 family protein			
FPS10_18570	[citrate (pro-3S)-lyase] ligase	GO:0000166,GO:0003824,GO:0005524,GO:0008080,GO:0008771,GO:0009058,GO:0016829,GO:0016874	K01910	C
FPS10_18575	citrate lyase acyl carrier protein		K01646	C
FPS10_18580	citrate (pro-3S)-lyase subunit beta	GO:0003824,GO:0005737,GO:0006084,GO:0008815,GO:0008816,GO:0009346,GO:0016829,GO:0046872	K01644	G
FPS10_18585	citrate lyase subunit alpha	GO:0005737,GO:0006084,GO:0008814,GO:0008815,GO:0009346,GO:0016829	K01643	C
FPS10_18590	triphosphoribosyl-dephospho-CoA synthase CitG	GO:0000166,GO:0005524,GO:0016310,GO:0016740,GO:0016757,GO:0046917	K05966	H
FPS10_18595	citrate lyase holo-[acyl-carrier protein] synthase		K05964	HI
FPS10_18600	alpha/beta hydrolase		K07002	R
FPS10_18605	adenosylcobinamide amidohydrolase			S
FPS10_18610	ABC transporter ATP-binding protein	GO:0000166,GO:0005524,GO:0008152,GO:0016887	K02013	PH
FPS10_18615	iron ABC transporter permease	GO:0005215,GO:0005886,GO:0006810,GO:0016020,GO:0016021	K02015	P
FPS10_18620	ABC transporter substrate-binding protein		K02016	P
FPS10_18625	TonB-dependent receptor		K16092	H
FPS10_18630	TonB family protein		K03832	M
FPS10_18635	biopolymer transporter ExbD		K03559	U
FPS10_18640	biopolymer transporter ExbD	GO:0005215,GO:0005886,GO:0006810,GO:0015031,GO:0016020,GO:0016021	K03559	U
FPS10_18645	MotA/TolQ/ExbB proton channel family protein	GO:0006810,GO:0008565,GO:0015031,GO:0016020,GO:0016021	K03561	U
FPS10_18650	putative transporter		K07085	R
FPS10_18655				
FPS10_18660	hypothetical protein			
FPS10_18665	nicotinate-nucleotide adenylyltransferase		K00969	H
FPS10_18670	MBL fold metallo-hydrolase			R
FPS10_18675	GFA family protein			S
FPS10_18680	energy-dependent translational throttle protein EttA	GO:0000166,GO:0005524,GO:0008152,GO:0016887		R
FPS10_18685	hypothetical protein			
FPS10_18695	mandelate racemase/muconate lactonizing enzyme family protein	GO:0003824,GO:0008152,GO:0046872		MR
FPS10_18700	TRAP transporter substrate-binding protein			Q
FPS10_18705	GntR family transcriptional regulator	GO:0003677,GO:0003700,GO:0006351,GO:0006355		K
FPS10_18710	mandelate racemase/muconate lactonizing enzyme family protein	GO:0003824,GO:0008152,GO:0046872	K18983	MR
FPS10_18715	F0F1 ATP synthase subunit beta		K02112	C
FPS10_18720	F0F1 ATP synthase subunit epsilon	GO:0005524,GO:0005886,GO:0006754,GO:0006810,GO:0006811,GO:0015986,GO:0015992,GO:0016020,GO:0042777,GO:0045261,GO:0046933,GO:0046961	K02114	C
FPS10_18725	AtpZ/AtpI family protein		K02116	
FPS10_18730	hypothetical protein			
FPS10_18735	F0F1 ATP synthase subunit A	GO:0005886,GO:0006754,GO:0006810,GO:0006811,GO:0015078,GO:0015986,GO:0015992,GO:0016020,GO:0016021,GO:0042777,GO:0045263,GO:0046933	K02108	C
FPS10_18740	F0F1 ATP synthase subunit C		K02110	C
FPS10_18745	hypothetical protein	GO:0005886,GO:0006754,GO:0006810,GO:0006811,GO:0015078,GO:0015986,GO:0015992,GO:0016020,GO:0016021,GO:0042777,GO:0045263,GO:0046933	K02109	C
FPS10_18750	F0F1 ATP synthase subunit alpha	GO:0000166,GO:0005524,GO:0005886,GO:0006754,GO:0006810,GO:0006811,GO:0015986,GO:0015991,GO:0015992,GO:0016020,GO:0016787,GO:0016820,GO:0033178,GO:0042777,GO:0045261,GO:0046034,GO:0046933,GO:0046961	K02111	C
FPS10_18755	hypothetical protein	GO:0015986,GO:0045261,GO:0046933,GO:0046961	K02115	C
FPS10_18760	carbon-nitrogen hydrolase family protein	GO:0006807,GO:0016787,GO:0016810	K01506	R
FPS10_18765	aminotransferase class III-fold pyridoxal phosphate-dependent enzyme	GO:0003824,GO:0008152,GO:0008483,GO:0030170		H
FPS10_18770	glycerol-3-phosphate dehydrogenase/oxidase		K00111	C
FPS10_18775	carbohydrate kinase		K00880	G
FPS10_18780	DeoR/GlpR transcriptional regulator	GO:0003677,GO:0003700,GO:0005622,GO:0006351,GO:0006355		KG
FPS10_18785	transaldolase		K00616	G

FPS10_18790	sugar-binding transcriptional regulator	GO:0030246		K
FPS10_18795	aminotransferase		K21909	
FPS10_18800	glycerol-3-phosphate dehydrogenase	GO:0004368,GO:0006072,GO:0009331,GO:0016491,GO:005259 0,GO:0052591,GO:0055114	K21054	C
FPS10_18805	carbohydrate kinase		K00862	G
FPS10_18810	DUF2291 domain-containing protein			R
FPS10_18815	ATP-binding cassette domain-containing protein	GO:0000166,GO:0005524,GO:0008152,GO:0016887	K17204	G
FPS10_18820	ABC transporter permease	GO:0005215,GO:0005886,GO:0006810,GO:0016020,GO:001602 1	K17203	G
FPS10_18825	D-ribose ABC transporter substrate-binding protein		K17202	G
FPS10_18830	hypothetical protein			
FPS10_18835	FadR family transcriptional regulator			K
FPS10_18850	4Fe-4S dicluster domain-containing protein			C
FPS10_18855	aldehyde ferredoxin oxidoreductase family protein	GO:0009055,GO:0016491,GO:0016625,GO:0033726,GO:005153 6,GO:0055114	K03738	C
FPS10_18860	NAD(P)/FAD-dependent oxidoreductase			C
FPS10_18865	MoaD/ThiS family protein			H
FPS10_18870	hypothetical protein			
FPS10_18875	HesA/MoeB/ThiF family protein		K21029	H
FPS10_18880	tripartite tricarboxylate transporter substrate binding protein			S
FPS10_18885	tripartite tricarboxylate transporter TctB family protein			
FPS10_18890	tripartite tricarboxylate transporter permease	GO:0016020,GO:0016021		S
FPS10_18895	phosphoribosyltransferase	GO:0009116,GO:0016740,GO:0016757		F
FPS10_18900	amidohydrolase			R
FPS10_18905	adenine deaminase	GO:0000034,GO:0006146,GO:0016787,GO:0016810	K01486	F
FPS10_18910	nucleoside hydrolase	GO:0008152,GO:0016787		F
FPS10_18915	ABC transporter ATP-binding protein		K02052	E
FPS10_18920	ABC transporter permease		K02053	E
FPS10_18925	ABC transporter permease		K02054	E
FPS10_18930	ABC transporter substrate-binding protein		K02055	E
FPS10_18935	LacI family transcriptional regulator	GO:0003677,GO:0003700,GO:0006351,GO:0006355	K02529	K
FPS10_18940	GntR family transcriptional regulator		K22293	K
FPS10_18945	glucuronate isomerase	GO:0006064,GO:0008880,GO:0016853	K01812	G
FPS10_18950	TRAP transporter substrate-binding protein	GO:0006810,GO:0030288		G
FPS10_18955	TRAP transporter small permease			G
FPS10_18960	TRAP transporter large permease	GO:0016020,GO:0016021		G
FPS10_18965	cupin domain-containing protein			S
FPS10_18970	5-dehydro-4-deoxy-D-glucuronate isomerase	GO:0008270,GO:0008697,GO:0016853,GO:0016861,GO:004549 0,GO:0046872	K01815	G
FPS10_18975	FadR family transcriptional regulator		K05799	K
FPS10_18980	tagaturonate reductase	GO:0003824,GO:0008152,GO:0009026,GO:0016491,GO:005066 2,GO:0055114	K00041	G
FPS10_18985	altronate dehydratase	GO:0008152,GO:0016829,GO:0016853	K01685	G
FPS10_18990	polysaccharide deacetylase family protein			G
FPS10_18995	nuclear transport factor 2 family protein			R
FPS10_19000	cryptochrome/photolyase family protein		K06876	R
FPS10_19005	SDR family NAD(P)-dependent oxidoreductase			IQR
FPS10_19010	TRAP transporter large permease			G
FPS10_19015	TRAP transporter small permease subunit	GO:0016020,GO:0016021		G
FPS10_19020	TRAP transporter substrate-binding protein	GO:0006810,GO:0030288		G
FPS10_19025	hypothetical protein			G
FPS10_19030	RbsD/FucU transporter		K02431	G
FPS10_19035	SDR family oxidoreductase		K00059	IQR
FPS10_19040				
FPS10_19050	SPOR domain-containing protein			
FPS10_19055	D-alanyl-D-alanine carboxypeptidase		K07258	M
FPS10_19060	dTMP kinase	GO:0000166,GO:0004798,GO:0005524,GO:0006233,GO:00062[illegible] 5,GO:0009165,GO:0016301,GO:0016310,GO:0016740,GO:00469 39	K00943	F
FPS10_19065	DNA polymerase III subunit delta'		K02341	L
FPS10_19070	TatD family deoxyribonuclease	GO:0004536,GO:0006259,GO:0008152,GO:0016787,GO:001678 8,GO:0016888	K03424	L
FPS10_19075	MBL fold metallo-hydrolase		K06167	R
FPS10_19080	AEC family transporter	GO:0016020,GO:0016021,GO:0055085	K07088	R
FPS10_19085	ABC transporter permease	GO:0005886,GO:0006810,GO:0016020,GO:0016021	K02053	E
FPS10_19090	ABC transporter permease	GO:0005886,GO:0006810,GO:0016020,GO:0016021	K02054	E
FPS10_19095	ABC transporter substrate-binding protein		K02055	E

FPS10_19100	ABC transporter ATP-binding protein	GO:0000166,GO:0005215,GO:0005524,GO:0005886,GO:0006810,GO:0008152,GO:0015417,GO:0015846,GO:0016020,GO:0016787,GO:0016820,GO:0016887,GO:0043190,GO:0055085,GO:1902047	K02052	E
FPS10_19105	zinc ABC transporter substrate-binding protein	GO:0030001,GO:0046872	K09815	P
FPS10_19110	transcriptional repressor		K09823	P
FPS10_19115	metal ABC transporter ATP-binding protein	GO:0000166,GO:0005524,GO:0005886,GO:0006810,GO:0006811,GO:0006829,GO:0008152,GO:0015633,GO:0016020,GO:0016787,GO:0016887,GO:0043190,GO:0071577	K09817	P
FPS10_19120	hypothetical protein		K09816	P
FPS10_19125	DUF2892 domain-containing protein	GO:0016020,GO:0016021		
FPS10_19130	HAMP domain-containing protein		K03406	NT
FPS10_19135	hypothetical protein			
FPS10_19140	DUF1566 domain-containing protein			
FPS10_19145	TrkH family potassium uptake protein	GO:0005886,GO:0006810,GO:0006811,GO:0006812,GO:0006813,GO:0008324,GO:0016020,GO:0016021,GO:0022820,GO:0055085,GO:0071805	K03498	P
FPS10_19150	polysaccharide biosynthesis tyrosine autokinase		K16554	D
FPS10_19155	DeoR/GlpR transcriptional regulator	GO:0003677,GO:0003700,GO:0005622,GO:0006351,GO:0006355	K02444	KG
FPS10_19160	glycerol-3-phosphate dehydrogenase	GO:0004368,GO:0006071,GO:0006072,GO:0009331,GO:0016491,GO:0052590,GO:0052591,GO:0055114	K00111	C
FPS10_19165	glycerol kinase GlpK	GO:0000166,GO:0004370,GO:0005524,GO:0005975,GO:0006071,GO:0006072,GO:0016301,GO:0016310,GO:0016740,GO:0016773,GO:0019563	K00864	C
FPS10_19170	UDP-glucose/GDP-mannose dehydrogenase family protein		K00012	M
FPS10_19175	phospholipid carrier-dependent glycosyltransferase			M
FPS10_19180	lipid A biosynthesis protein			S
FPS10_19185	glycosyltransferase family 2 protein	GO:0008152,GO:0016740,GO:0016757		M
FPS10_19190	LacI family DNA-binding transcriptional regulator	GO:0003677,GO:0003700,GO:0006351,GO:0006355	K06145	K
FPS10_19195	flagellar basal body-associated protein FliL			
FPS10_19200	flagellar basal body L-ring protein FlgH		K02393	N
FPS10_19205	flagellar basal body P-ring formation protein FlgA	GO:0042597,GO:0044781	K02386	NO
FPS10_19210	flagellar basal-body rod protein FlgG	GO:0009288,GO:0009425,GO:0009426,GO:0071973	K02392	N
FPS10_19215	flagellar hook-basal body complex protein	GO:0009288,GO:0009425,GO:0030694,GO:0071973	K02391	N
FPS10_19220	flagellar biosynthetic protein FliQ	GO:0009306,GO:0016020,GO:0016021,GO:0044780	K02420	NU
FPS10_19225	flagellar hook-basal body complex protein FliE	GO:0003774,GO:0005198,GO:0008152,GO:0009288,GO:0071973	K02408	NU
FPS10_19230	flagellar basal body rod protein FlgC	GO:0009288,GO:0009425,GO:0030694,GO:0071973	K02388	N
FPS10_19235	FlgB family protein	GO:0071973	K02387	N
FPS10_19240	FliI/YscN family ATPase		K02412	NU
FPS10_19245	hypothetical protein			
FPS10_19250	flagellar biosynthesis protein FlhB		K02401	NU
FPS10_19255	type III secretion protein	GO:0005622,GO:0006605,GO:0016020,GO:0016021	K02421	NU
FPS10_19260	flagellar biosynthesis protein FlhA	GO:0009306,GO:0016020,GO:0016021,GO:0044780	K02400	NU
FPS10_19265	hypothetical protein			
FPS10_19270	hypothetical protein			
FPS10_19275	hypothetical protein			
FPS10_19280	hypothetical protein			
FPS10_19285	hypothetical protein			
FPS10_19290	hypothetical protein			
FPS10_19295	TIGR03862 family flavoprotein	GO:0016491,GO:0055114		R
FPS10_19300	HNH endonuclease			
FPS10_19305	enoyl-CoA hydratase/isomerase family protein	GO:0003824,GO:0008152,GO:0016853		I
FPS10_19310	SDR family oxidoreductase	GO:0008152,GO:0016491,GO:0055114		R
FPS10_19315	DUF2125 domain-containing protein			
FPS10_19320	DUF2796 domain-containing protein			
FPS10_19325	ABC transporter ATP-binding protein	GO:0000166,GO:0005524,GO:0008152,GO:0016887	K02003	V
FPS10_19330	ABC transporter permease	GO:0005886,GO:0016020,GO:0016021	K02004	V
FPS10_19335	hypothetical protein			
FPS10_19340	DUF3299 domain-containing protein		K09950	S
FPS10_19345	ubiquinone biosynthesis protein UbiB		K03688	R
FPS10_19350	PDZ domain-containing protein			O
FPS10_19355	TldD/PmbA family protein		K03592	R
FPS10_19360	3'(2')%2C5'-bisphosphate nucleotidase CysQ	GO:0008934,GO:0016311,GO:0016787,GO:0046854,GO:0052832,GO:0052833,GO:0052834	K01092	G
FPS10_19365	DUF4170 domain-containing protein			
FPS10_19370	3-deoxy-D-manno-octulosonic acid transferase		K02527	M
FPS10_19375	tetraacyldisaccharide 4'-kinase	GO:0000166,GO:0005524,GO:0006629,GO:0009029,GO:0009245,GO:0016301,GO:0016310,GO:0016740	K00912	M
FPS10_19380	DsbA family protein			O

FPS10_19385	DUF721 domain-containing protein			S
FPS10_19390	A/G-specific adenine glycosylase		K03575	L
FPS10_19395	alkane 1-monooxygenase	GO:0006629,GO:0016020,GO:0016021	K00496	
FPS10_19400	class I SAM-dependent methyltransferase	GO:0008168,GO:0016740,GO:0032259		H
FPS10_19405	site-specific DNA-methyltransferase		K13581	L
FPS10_19410	ribonuclease HII	GO:0003676,GO:0003723,GO:0004518,GO:0004519,GO:0004523,GO:0005737,GO:0006401,GO:0016787,GO:0030145,GO:0046872,GO:0090305,GO:0090502	K03470	L
FPS10_19415	glutathione S-transferase family protein	GO:0008152,GO:0016740	K00799	O
FPS10_19420	DNA polymerase III subunit delta	GO:0003677,GO:0006260	K02340	L
FPS10_19425	hypothetical protein		K03643	
FPS10_19430	leucine--tRNA ligase	GO:0000166,GO:0002161,GO:0004812,GO:0004823,GO:0005524,GO:0005737,GO:0006412,GO:0006418,GO:0006429,GO:0006450,GO:0016874	K01869	J
FPS10_19435	DUF3576 domain-containing protein			
FPS10_19440	porin		K08720	
FPS10_19445	tetratricopeptide repeat protein			R
FPS10_19450	YggS family pyridoxal phosphate-dependent enzyme		K06997	R
FPS10_19455	L%2CD-transpeptidase family protein			S
FPS10_19460	GTP cyclohydrolase II	GO:0000166,GO:0003935,GO:0005525,GO:0009231,GO:0016787,GO:0046872	K01497	H
FPS10_19465	response regulator transcription factor	GO:0000160,GO:0003677,GO:0005622,GO:0006351,GO:00063[illegible]5		TK
FPS10_19470	exodeoxyribonuclease III		K01142	L
FPS10_19475	co-chaperone YbbN	GO:0005623,GO:0006662,GO:0015035,GO:0045454,GO:00551[illegible]4	K05838	O
FPS10_19480	ATP-dependent protease		K07157	R
FPS10_19485	Trm112 family protein		K09791	S
FPS10_19490	2-octaprenyl-6-methoxyphenyl hydroxylase	GO:0006744,GO:0016491,GO:0016709,GO:0050660,GO:0055114,GO:0071949	K03185	HC
FPS10_19495	amidase	GO:0008152,GO:0016884	K02433	J
FPS10_19500	aminotransferase class I/II-fold pyridoxal phosphate-dependent enzyme	GO:0003824,GO:0008483,GO:0009058,GO:0016740,GO:0030170		E
FPS10_19505	DNA translocase FtsK		K03466	P
FPS10_19510	outer membrane lipoprotein carrier protein LolA	GO:0006810,GO:0015031,GO:0042597		M
FPS10_19515	lytic transglycosylase			S
FPS10_19520	AEC family transporter		K07088	R
FPS10_19525	heat shock protein HspQ	GO:0003677	K11940	S
FPS10_19530	gamma-glutamyltransferase family protein	GO:0003840,GO:0006749	K00681	E
FPS10_19535	ATP-binding protein		K04757	T
FPS10_19540	STAS domain-containing protein	GO:0006355,GO:0045152	K04749	T
FPS10_19545	acetyl-CoA C-acyltransferase	GO:0003824,GO:0003985,GO:0008152,GO:0016740,GO:0016746,GO:0016747	K00626	I
FPS10_19550	GAF domain-containing protein		K08968	T
FPS10_19555	RluA family pseudouridine synthase	GO:0001522,GO:0003723,GO:0009451,GO:0009982	K06177	J
FPS10_19560	aldehyde dehydrogenase family protein	GO:0008152,GO:0016491,GO:0016620,GO:0055114	K00128	C
FPS10_19565	deoxyribose-phosphate aldolase	GO:0003824,GO:0004139,GO:0005737,GO:0009264,GO:00168[illegible]9	K01619	F
FPS10_19570	hypothetical protein			
FPS10_19575	hypothetical protein			
FPS10_19580	sulfate permease		K03321	P
FPS10_19585	TIGR01244 family phosphatase	GO:0016491,GO:0016787,GO:0055114	K17218	R
FPS10_19590	MBL fold metallo-hydrolase			R
FPS10_19600	YeeE/YedE family protein	GO:0016020,GO:0016021	K07112	R
FPS10_19605	DUF2892 domain-containing protein	GO:0016020,GO:0016021		
FPS10_19610	elongation factor G	GO:0003746,GO:0005525,GO:0006414	K02355	J
FPS10_19615	aldo/keto reductase	GO:0004032,GO:0016491,GO:0050580,GO:0055114	K06221	R
FPS10_19620	CTP synthase	GO:0000166,GO:0003883,GO:0005524,GO:0006221,GO:0006541,GO:0016874,GO:0044210	K01937	F
FPS10_19625	preprotein translocase subunit SecG		K03075	U
FPS10_19630	adenylosuccinate synthase	GO:0000166,GO:0000287,GO:0004019,GO:0005525,GO:0005737,GO:0006164,GO:0016874,GO:0044208,GO:0046872	K01939	F
FPS10_19635	DUF2842 domain-containing protein	GO:0016020,GO:0016021		
FPS10_19640	hypothetical protein			
FPS10_19645	thiamine diphosphokinase	GO:0004788,GO:0005524,GO:0006772,GO:0009229,GO:0016301,GO:0016310,GO:0030975	K00949	H
FPS10_19650	DMT family transporter	GO:0016020,GO:0016021		GER
FPS10_19655	L-serine ammonia-lyase	GO:0003941,GO:0006094,GO:0016829,GO:0051539	K01752	E
FPS10_19660	LacI family transcriptional regulator		K02529	K
FPS10_19665	ABC transporter permease	GO:0005215,GO:0005886,GO:0006810,GO:0016020,GO:00160[illegible]1	K02057	G
FPS10_19670	autoinducer 2 ABC transporter substrate-binding protein		K02058	G

FPS10_19675	sugar ABC transporter ATP-binding protein	GO:0000166,GO:0005524,GO:0008152,GO:0016887		G
FPS10_19680	ABC transporter permease	GO:0005215,GO:0005886,GO:0006810,GO:0016020,GO:0016021	K02057	G
FPS10_19685	dihydroxyacetone kinase subunit DhaK		K05878	G
FPS10_19690	dihydroxyacetone kinase subunit L		K05879	G
FPS10_19695	ribose-5-phosphate isomerase RpiA	GO:0004751,GO:0006098,GO:0009052,GO:0016853	K01807	G
FPS10_19700	hypothetical protein			
FPS10_19705	hypothetical protein			H
FPS10_19710	YecA family protein			
FPS10_19715	hypothetical protein			
FPS10_19720	AraC family transcriptional regulator			K
FPS10_19725	hypothetical protein			
FPS10_19730	helix-turn-helix transcriptional regulator	GO:0003677,GO:0006351,GO:0006355		TK
FPS10_19740	hypothetical protein			
FPS10_19745	META domain-containing protein			O
FPS10_19750	hypothetical protein			
FPS10_19755	hypothetical protein			
FPS10_19760	DUF1127 domain-containing protein			
FPS10_19765	hypothetical protein			
FPS10_19770	hypothetical protein			
FPS10_19775	hypothetical protein	GO:0004222,GO:0005886,GO:0006508,GO:0008233,GO:0008237,GO:0008270,GO:0016020,GO:0016021,GO:0016787,GO:0046872		
FPS10_19780	rhomboid family intramembrane serine protease	GO:0004252,GO:0006508,GO:0016020,GO:0016021		R
FPS10_19785	hypothetical protein			
FPS10_19790	GNAT family N-acetyltransferase			R
FPS10_19795	methyltransferase domain-containing protein			H
FPS10_19800	phosphotransferase			
FPS10_19805	hypothetical protein			
FPS10_19810	hypothetical protein			
FPS10_19815	FAD-dependent oxidoreductase		K00274	E
FPS10_19825	AAA family ATPase			D
FPS10_19830	DNA repair exonuclease	GO:0004527,GO:0008152,GO:0016787,GO:0090305		L
FPS10_19835	multidrug efflux SMR transporter		K03297	P
FPS10_19840	MerR family transcriptional regulator			K
FPS10_19860	tyrosine-type recombinase/integrase	GO:0003677,GO:0006310,GO:0015074		L
FPS10_19865	AlpA family phage regulatory protein		K07733	K
FPS10_19870	hypothetical protein			
FPS10_19875	hypothetical protein			
FPS10_19885	hypothetical protein			
FPS10_19890	alpha/beta fold hydrolase			R
FPS10_19895	hypothetical protein			
FPS10_19905	IS6 family transposase	GO:0003676	K07498	L
FPS10_19910	transposase			
FPS10_19915	integrase			
FPS10_19920	NAD(P)-dependent alcohol dehydrogenase	GO:0008270,GO:0016491,GO:0055114		CR
FPS10_19925	NAD(P)-dependent alcohol dehydrogenase	GO:0008270,GO:0016491,GO:0055114		CR
FPS10_19930	N-acyl homoserine lactonase family protein		K13075	R
FPS10_19935	2TM domain-containing protein			
FPS10_19940	ABC transporter ATP-binding protein		K01990	V
FPS10_19945	ABC transporter permease		K01992	V
FPS10_19950	ABC transporter permease subunit		K01992	V
FPS10_19960	AraC family transcriptional regulator			K
FPS10_19965	transporter			
FPS10_19970	hypothetical protein			
FPS10_19975	hypothetical protein			
FPS10_19980	DUF1214 domain-containing protein			S
FPS10_19985	DUF1214 domain-containing protein			S
FPS10_19990	DUF1214 domain-containing protein			S
FPS10_19995	hypothetical protein			
FPS10_20000	transposase			
FPS10_20005	hypothetical protein	GO:0016020,GO:0016021		S
FPS10_20015	glutamate synthase	GO:0003824,GO:0006537,GO:0015930,GO:0016638,GO:0043231,GO:0051537,GO:0055114		E
FPS10_20020	ferric reductase	GO:0016020,GO:0016021		
FPS10_20025	twin-arginine translocation pathway signal	GO:0005506,GO:0016702,GO:0055114		Q
FPS10_20030	DUF1194 domain-containing protein			
FPS10_20035	histidine phosphatase family protein			T

FPS10_20040	dienelactone hydrolase family protein			Q
FPS10_20055	carbohydrate porin		K07267	M
FPS10_20060	DoxX family protein	GO:0016020,GO:0016021		
FPS10_20065	hypothetical protein			
FPS10_20070	DUF5117 domain-containing protein			
FPS10_20075	amidohydrolase family protein	GO:0006508,GO:0008152,GO:0016787,GO:0016805,GO:00168[illegible]0		Q
FPS10_20080	amidohydrolase family protein			Q
FPS10_20085	AraC family transcriptional regulator			K
FPS10_20090	rhamnulose-1-phosphate aldolase		K01629	G
FPS10_20100	class II aldolase/adducin family protein		K01628	G
FPS10_20105	hypothetical protein			S
FPS10_20110	carbohydrate ABC transporter permease	GO:0005886,GO:0006810,GO:0016020,GO:0016021	K02026	G
FPS10_20115	sugar ABC transporter permease		K02025	G
FPS10_20120	extracellular solute-binding protein	GO:0005215,GO:0006810	K02027	G
FPS10_20125	sn-glycerol-3-phosphate ABC transporter ATP-binding protein UgpC	GO:0000166,GO:0005215,GO:0005524,GO:0006810,GO:0008152,GO:0016820,GO:0016887,GO:0043190,GO:0055085	K10112	G
FPS10_20130	GntR family transcriptional regulator	GO:0003677,GO:0003700,GO:0006351,GO:0006355		K
FPS10_20135	N-acyl homoserine lactonase family protein			R
FPS10_20140	hypothetical protein			G
FPS10_20145	class II aldolase			S
FPS10_20150	carbohydrate kinase	GO:0005975,GO:0016301,GO:0016310,GO:0016773	K22935	G
FPS10_20155	hypothetical protein		K15778	G
FPS10_20160	DUF995 domain-containing protein			
FPS10_20165	SDR family oxidoreductase		K08678	MG
FPS10_20170	UDP-glucose 4-epimerase GalE	GO:0003824,GO:0003978,GO:0006012,GO:0016853,GO:00506[illegible]2	K12448	M
FPS10_20175	glycosyltransferase			M
FPS10_20180	beta-mannosidase			G
FPS10_20185	hypothetical protein			
FPS10_20190	hypothetical protein			
FPS10_20195	hypothetical protein	GO:0005975		
FPS10_20200	hypothetical protein			
FPS10_20205	hypothetical protein	GO:0016020,GO:0016021		
FPS10_20210	LysR family transcriptional regulator	GO:0003677,GO:0003700,GO:0006351,GO:0006355		K
FPS10_20215	multidrug effflux MFS transporter		K07552	G
FPS10_20220	biotin/lipoyl-binding protein			M
FPS10_20225	efflux RND transporter permease subunit			V
FPS10_20230	DSD1 family PLP-dependent enzyme		K18425	E
FPS10_20235	aminotransferase class V-fold PLP-dependent enzyme	GO:0003824,GO:0008152,GO:0008483,GO:0016740	K00830	E
FPS10_20240	hypothetical protein			
FPS10_20245	host attachment protein			N
FPS10_20250	phosphoribosyltransferase	GO:0009116,GO:0016740,GO:0016757	K07100	R
FPS10_20255	TRAP transporter large permease			G
FPS10_20260	TRAP transporter small permease subunit	GO:0016020,GO:0016021		G
FPS10_20265	TRAP transporter substrate-binding protein	GO:0006810,GO:0030288		G
FPS10_20270	GntR family transcriptional regulator			K
FPS10_20275	4-hydroxythreonine-4-phosphate dehydrogenase		K00097	H
FPS10_20280	hypothetical protein			
FPS10_20285	hypothetical protein	GO:0006520,GO:0008851,GO:0016829,GO:0031419,GO:00463[illegible]6	K03736	E
FPS10_20290	glucose 1-dehydrogenase	GO:0008152,GO:0016491,GO:0055114	K00059	IQR
FPS10_20310	hypothetical protein			K
FPS10_20315	IS30 family transposase			L
FPS10_20320	hypothetical protein			
FPS10_20325	helix-turn-helix transcriptional regulator			K
FPS10_20330	DUF1254 domain-containing protein			S
FPS10_20335	DUF1254 domain-containing protein			S
FPS10_20340	DUF1254 domain-containing protein			S
FPS10_20345	DUF1254 domain-containing protein			S
FPS10_20350	DUF1254 domain-containing protein			S
FPS10_20355	hypothetical protein			
FPS10_20360	mechanosensitive ion channel		K16052	M
FPS10_20365	hypothetical protein			
FPS10_20370	hypothetical protein			S
FPS10_20375	hypothetical protein			
FPS10_20380	TetR/AcrR family transcriptional regulator			K
FPS10_20385	AraC family transcriptional regulator			K
FPS10_20390	AI-2E family transporter			R

FPS10_20395	mechanosensitive ion channel family protein		K16052	M
FPS10_20400	helix-turn-helix transcriptional regulator			K
FPS10_20405	hypothetical protein			
FPS10_20410	hypothetical protein			S
FPS10_20415	hypothetical protein			
FPS10_20420	hypothetical protein			
FPS10_20425	hypothetical protein			R
FPS10_20430	hypothetical protein			
FPS10_20435	hypothetical protein	GO:0016020,GO:0016021		
FPS10_20440	hypothetical protein			
FPS10_20445	hypothetical protein			
FPS10_20450	DUF805 domain-containing protein			S
FPS10_20455	hypothetical protein			
FPS10_20465	DUF3955 domain-containing protein			
FPS10_20470	hypothetical protein			
FPS10_20480	transposase			
FPS10_20485	citryl-CoA lyase		K01647	C
FPS10_20490	phenylacetate--CoA ligase family protein		K01912	H
FPS10_20495	VOC family protein			E
FPS10_20500	hemerythrin domain-containing protein			
FPS10_20505	hypothetical protein			
FPS10_20510	methyltransferase			J
FPS10_20515	hypothetical protein			G
FPS10_20520	sn-glycerol-3-phosphate ABC transporter substrate-binding protein UgpB		K05813	G
FPS10_20525	sn-glycerol-3-phosphate ABC transporter permease UgpA	GO:0005886,GO:0006810,GO:0016020,GO:0016021	K05814	G
FPS10_20530	sn-glycerol-3-phosphate ABC transporter permease UgpE	GO:0001407,GO:0005886,GO:0005887,GO:0006810,GO:0015794,GO:0016020,GO:0016021,GO:0055052	K05815	G
FPS10_20535	sn-glycerol-3-phosphate ABC transporter ATP-binding protein UgpC	GO:0000166,GO:0005215,GO:0005524,GO:0006810,GO:0008152,GO:0015430,GO:0015794,GO:0016787,GO:0016820,GO:0016887,GO:0043190,GO:0055085	K05816	G
FPS10_20540	hypothetical protein			
FPS10_20545	hypothetical protein			
FPS10_20550	Na/Pi cotransporter family protein		K03324	P
FPS10_20555	inorganic phosphate transporter	GO:0005315,GO:0006817,GO:0016020,GO:0016021,GO:0055085	K03306	P
FPS10_20560	TRAP transporter large permease subunit	GO:0016020,GO:0016021		G
FPS10_20565	TRAP transporter small permease subunit			G
FPS10_20570	C4-dicarboxylate ABC transporter substrate-binding protein			G
FPS10_20575	OsmC family protein			O
FPS10_20580	ABC transporter permease		K01992	V
FPS10_20585	ABC transporter ATP-binding protein	GO:0000166,GO:0005524,GO:0008152,GO:0016887	K01990	V
FPS10_20590	HlyD family efflux transporter periplasmic adaptor subunit		K01993	V
FPS10_20595	DUF1956 domain-containing protein			K
FPS10_20600	tripartite tricarboxylate transporter substrate binding protein			S
FPS10_20605	hypothetical protein			
FPS10_20610	tripartite tricarboxylate transporter permease	GO:0016020,GO:0016021		S
FPS10_20615	SDR family oxidoreductase			IQR
FPS10_20620	FadR family transcriptional regulator		K05799	K
FPS10_20625	RraA family protein			H
FPS10_20630	fucose isomerase			G
FPS10_20645	transposase			
FPS10_20650	EAL domain-containing protein			T
FPS10_20655	hypothetical protein			T
FPS10_20660	FAD-binding protein	GO:0016491,GO:0055114		R
FPS10_20665	(2Fe-2S)-binding protein	GO:0009055,GO:0051536		
FPS10_20670	FAD-binding oxidoreductase	GO:0016020,GO:0016021,GO:0016491,GO:0055114		E
FPS10_20675	C4-dicarboxylate ABC transporter	GO:0006810,GO:0030288		G
FPS10_20680	TRAP transporter large permease	GO:0016020,GO:0016021		G
FPS10_20685	TRAP transporter small permease	GO:0016020,GO:0016021		G
FPS10_20690	LysR family transcriptional regulator	GO:0003677,GO:0003700,GO:0006351,GO:0006355		K
FPS10_20695	fasciclin domain-containing protein			M
FPS10_20700	DUF3047 domain-containing protein			
FPS10_20705	lipocalin family protein		K03098	M
FPS10_20710	sn-glycerol-3-phosphate ABC transporter ATP-binding protein UgpC		K10112	G
FPS10_20715	GntR family transcriptional regulator	GO:0003700,GO:0006355		K
FPS10_20720	alpha/beta hydrolase	GO:0008152,GO:0016787	K01066	I

FPS10_20725	sugar ABC transporter substrate-binding protein		K02027	G
FPS10_20730	sugar ABC transporter permease	GO:0005886,GO:0006810,GO:0016020,GO:0016021	K02025	G
FPS10_20735	carbohydrate ABC transporter permease	GO:0005886,GO:0006810,GO:0016020,GO:0016021	K02026	G
FPS10_20740	sugar phosphate isomerase/epimerase		K18910	G
FPS10_20745	Gfo/Idh/MocA family oxidoreductase			R
FPS10_20750	DMT family transporter	GO:0016020,GO:0016021		GER
FPS10_20755	HAD family phosphatase			R
FPS10_20760	HAMP domain-containing protein			T
FPS10_20765	response regulator transcription factor	GO:0000160,GO:0003677,GO:0005622,GO:0006351,GO:00063[illegible] 5	K02483	TK
FPS10_20770	ABC transporter substrate-binding protein		K02012	P
FPS10_20775	iron ABC transporter permease		K02011	P
FPS10_20780	ABC transporter ATP-binding protein	GO:0000166,GO:0005215,GO:0005524,GO:0006810,GO:0008152,GO:0016820,GO:0016887,GO:0043190,GO:0055085	K02010	E
FPS10_20785	Gfo/Idh/MocA family oxidoreductase	GO:0008152,GO:0016491,GO:0055114		R
FPS10_20790	Gfo/Idh/MocA family oxidoreductase			R
FPS10_20795	hypothetical protein			
FPS10_20800	Gfo/Idh/MocA family oxidoreductase	GO:0016491,GO:0055114		R
FPS10_20805	substrate-binding domain-containing protein		K02529	G
FPS10_20810	CocE/NonD family hydrolase	GO:0006508,GO:0008152,GO:0008239,GO:0016787	K06978	R
FPS10_20815	ABC transporter substrate-binding protein	GO:0043190,GO:0055085	K02035	E
FPS10_20820	dihydroxy-acid dehydratase		K01687	EG
FPS10_20825	dihydroxy-acid dehydratase		K01687	EG
FPS10_20830	hypothetical protein			
FPS10_20835	cation-transporting P-type ATPase	GO:0000166,GO:0005524,GO:0008152,GO:0016020,GO:0016021,GO:0016787,GO:0046872		P
FPS10_20840	hypothetical protein			
FPS10_20845	AAA family ATPase	GO:0004176,GO:0004252,GO:0005524,GO:0006508,GO:0030[illegible] 3		O
FPS10_20850	hypothetical protein			
FPS10_20855	pyruvate dehydrogenase (acetyl-transferring) E1 component subunit alpha		K00161	C
FPS10_20860	alpha-ketoacid dehydrogenase subunit beta	GO:0003824,GO:0008152	K00162	C
FPS10_20865	2-oxo acid dehydrogenase subunit E2	GO:0008152,GO:0016740,GO:0016746	K00627	C
FPS10_20870	Glu/Leu/Phe/Val dehydrogenase	GO:0006520,GO:0016491,GO:0016639,GO:0055114	K00261	E
FPS10_20875	hypothetical protein	GO:0016020,GO:0016021		
FPS10_20880	Hsp20/alpha crystallin family protein		K13993	O
FPS10_20885	NAD(P)-dependent oxidoreductase			MG
FPS10_20890	cytochrome C			
FPS10_20895	DUF1924 domain-containing protein			
FPS10_20900	cytochrome B			C
FPS10_20905	HAMP domain-containing histidine kinase			T
FPS10_20910	response regulator transcription factor	GO:0000160,GO:0003677,GO:0005622,GO:0006351,GO:00063[illegible] 5		TK
FPS10_20915	peptidase			S
FPS10_20920	efflux RND transporter permease subunit		K18138	V
FPS10_20925	efflux RND transporter periplasmic adaptor subunit		K03585	M
FPS10_20930	TetR/AcrR family transcriptional regulator	GO:0003677,GO:0006351,GO:0006355		K
FPS10_20935	LysR family transcriptional regulator			K
FPS10_20940	NAD(P)H-dependent oxidoreductase			C
FPS10_20945	SDR family NAD(P)-dependent oxidoreductase			R
FPS10_20950	hypothetical protein			
FPS10_20955	hypothetical protein			
FPS10_20960	EAL domain-containing protein			T
FPS10_20965	extracellular solute-binding protein		K11073	E
FPS10_20970	substrate-binding domain-containing protein		K10439	G
FPS10_20975	sugar ABC transporter ATP-binding protein		K10441	G
FPS10_20980	ABC transporter permease	GO:0005215,GO:0005886,GO:0006810,GO:0016020,GO:00160[illegible] 1	K10440	G
FPS10_20985	SMP-30/gluconolactonase/LRE family protein		K01053	G
FPS10_20990	LysR family transcriptional regulator	GO:0003677,GO:0003700,GO:0006351,GO:0006355		K
FPS10_20995	epimerase			MG
FPS10_21000	PAS domain S-box protein			TK
FPS10_21005	ABC transporter substrate-binding protein		K01999	E
FPS10_21010	ABC transporter ATP-binding protein	GO:0005524,GO:0008152,GO:0016887	K01995	E
FPS10_21015	ABC transporter ATP-binding protein		K01996	E
FPS10_21020	branched-chain amino acid ABC transporter permease	GO:0005215,GO:0005886,GO:0006810,GO:0016020,GO:00160[illegible] 1	K01997	E
FPS10_21025	branched-chain amino acid ABC transporter permease		K01998	E
FPS10_21030	alpha/beta hydrolase	GO:0008152,GO:0016787		R
FPS10_21035	DUF1214 domain-containing protein			S
FPS10_21040	DUF1254 domain-containing protein			S

FPS10_21045	DUF1254 domain-containing protein			
FPS10_21050	carboxylating nicotinate-nucleotide diphosphorylase	GO:0003824,GO:0004514,GO:0009435,GO:0016740,GO:0016757,GO:0016763,GO:0019363	K00767	H
FPS10_21055	L-aspartate oxidase	GO:0005737,GO:0008734,GO:0009435,GO:0016491,GO:0019363,GO:0044318,GO:0055114	K00278	H
FPS10_21060	quinolinate synthase NadA	GO:0003824,GO:0005737,GO:0008987,GO:0009435,GO:0016740,GO:0016765,GO:0019363,GO:0019805,GO:0046872,GO:0051536,GO:0051539	K03517	H
FPS10_21065	hypothetical protein			R
FPS10_21070	hypothetical protein			KE
FPS10_21075	hypothetical protein			
FPS10_21080	aminotransferase class III-fold pyridoxal phosphate-dependent enzyme	GO:0003824,GO:0008152,GO:0008483,GO:0016740,GO:0030170	K03851	H
FPS10_21085	LysR family transcriptional regulator	GO:0003677,GO:0003700,GO:0006351,GO:0006355		K
FPS10_21090	ABC transporter ATP-binding protein	GO:0000166,GO:0005524,GO:0008152,GO:0016887	K01996	E
FPS10_21095	ABC transporter ATP-binding protein	GO:0005524,GO:0008152,GO:0016887	K01995	E
FPS10_21100	branched-chain amino acid ABC transporter permease	GO:0005215,GO:0005886,GO:0006810,GO:0016020,GO:0016021	K01998	E
FPS10_21105	branched-chain amino acid ABC transporter permease	GO:0005215,GO:0005886,GO:0006810,GO:0016020,GO:0016021	K01997	E
FPS10_21110	ABC transporter substrate-binding protein		K01999	E
FPS10_21115	hypothetical protein			M
FPS10_21120	OmpA family protein	GO:0009279,GO:0016020,GO:0016021		S
FPS10_21125	caspase family protein	GO:0004197,GO:0006508		R
FPS10_21130	response regulator			TK
FPS10_21135	cytochrome C	GO:0005506,GO:0009055,GO:0009276,GO:0016021,GO:0020037	K03532	C
FPS10_21140	trimethylamine-N-oxide reductase TorA		K07812	C
FPS10_21145	hypothetical protein		K03532	
FPS10_21150	response regulator			T
FPS10_21155	bifunctional sugar-binding transcriptional regulator/dihydroxyacetone kinase subunit DhaK		K05878	G
FPS10_21160	dihydroxyacetone kinase subunit L		K05879	G
FPS10_21165	dihydroxyacetone kinase subunit L	GO:0004371,GO:0006071,GO:0016301,GO:0016310,GO:001677[illegible]2	K05879	G
FPS10_21170	dihydroxyacetone kinase subunit DhaK	GO:0004371,GO:0006071,GO:0016301,GO:0016310,GO:0016740	K05878	G
FPS10_21175	D-threitol dehydrogenase	GO:0008152,GO:0016491,GO:0055114		IQR
FPS10_21180	sugar ABC transporter substrate-binding protein		K17205	G
FPS10_21185	ABC transporter permease	GO:0005215,GO:0005886,GO:0006810,GO:0008152,GO:0015407,GO:0015749,GO:0016020,GO:0016021,GO:0016787	K17206	G
FPS10_21190	sugar ABC transporter ATP-binding protein		K17207	G
FPS10_21195	hypothetical protein			
FPS10_21200	hypothetical protein			
FPS10_21205	phosphoenolpyruvate--protein phosphotransferase		K02768	G
FPS10_21210	PTS glucose transporter subunit IIB	GO:0005355,GO:0005886,GO:0006810,GO:0008643,GO:0008982,GO:0009401,GO:0015758,GO:0016020,GO:0016021,GO:0016301,GO:0016310,GO:0016740,GO:0034219,GO:1904659	K02778	G
FPS10_21215	DEAD/DEAH box helicase	GO:0000166,GO:0003676,GO:0004386,GO:0005524,GO:0008152,GO:0016787	K05592	LKJ
FPS10_21220	sulfotransferase			
FPS10_21225	hypothetical protein			
FPS10_21230	hypothetical protein			
FPS10_21235	GtrA family protein			
FPS10_21240	UbiA family prenyltransferase			H
FPS10_21245	hypothetical protein			
FPS10_21250	HAMP domain-containing protein		K07642	T
FPS10_21255	response regulator	GO:0000160,GO:0003677,GO:0005622,GO:0006351,GO:0006355	K07664	TK
FPS10_21260	acyltransferase			I
FPS10_21265	lipoyl(octanoyl) transferase LipB	GO:0005737,GO:0006464,GO:0009107,GO:0016415,GO:0016740,GO:0016746,GO:0033819	K03801	H
FPS10_21270	hypothetical protein			
FPS10_21275	SDR family oxidoreductase			IQR
FPS10_21280	cytochrome c oxidase subunit I	GO:0004129,GO:0005506,GO:0005507,GO:0005886,GO:0006810,GO:0009055,GO:0009060,GO:0016020,GO:0016021,GO:0016491,GO:0020037,GO:0022900,GO:0055114,GO:0070469,GO:1902600	K02274	C
FPS10_21285	DUF2244 domain-containing protein	GO:0016020,GO:0016021		S
FPS10_21290	glycosyltransferase family 2 protein			
FPS10_21295	GNAT family N-acetyltransferase	GO:0008080,GO:0008152,GO:0016740		R
FPS10_21300	hypothetical protein			
FPS10_21305	N-acetyltransferase		K03823	M
FPS10_21310	GatB/YqeY domain-containing protein		K09117	S

FPS10_21315	glutamine-hydrolyzing carbamoyl-phosphate synthase small subunit	GO:0000166,GO:0004088,GO:0005524,GO:0006207,GO:0006221,GO:0006526,GO:0006541,GO:0008652,GO:0016874,GO:0044205	K01956	EF
FPS10_21320	glycosyltransferase			M
FPS10_21325	GntR family transcriptional regulator	GO:0003677,GO:0003700,GO:0006351,GO:0006355		K
FPS10_21330	pyrimidine 5'-nucleotidase	GO:0008152,GO:0016787	K07025	R
FPS10_21335	GlsB/YeaQ/YmgE family stress response membrane protein	GO:0016020,GO:0016021		S
FPS10_21340	sulfotransferase			
FPS10_21345	beta-ketoacyl-ACP synthase III	GO:0003824,GO:0004315,GO:0006633,GO:0008152,GO:0016740,GO:0016746	K16872	I
FPS10_21350	dihydrolipoyl dehydrogenase	GO:0004148,GO:0005623,GO:0006096,GO:0016491,GO:0016668,GO:0045454,GO:0050660,GO:0055114	K00382	C
FPS10_21355	2-oxoglutarate dehydrogenase complex dihydrolipoyllysine-residue succinyltransferase	GO:0004149,GO:0006099,GO:0008152,GO:0016740,GO:0016746,GO:0033512,GO:0045252	K00658	C
FPS10_21360	2-oxoglutarate dehydrogenase E1 component	GO:0004591,GO:0006099,GO:0008152,GO:0016491,GO:0016624,GO:0030976,GO:0055114	K00164	C
FPS10_21365	DUF805 domain-containing protein			S
FPS10_21370	succinate--CoA ligase subunit alpha	GO:0000166,GO:0003824,GO:0004775,GO:0005524,GO:0008152,GO:0016874,GO:0048037	K01902	C
FPS10_21375	ADP-forming succinate--CoA ligase subunit beta	GO:0000166,GO:0000287,GO:0003824,GO:0004775,GO:0005524,GO:0006099,GO:0008152,GO:0016874,GO:0030145,GO:0046872	K01903	C
FPS10_21380	hypothetical protein			D
FPS10_21385	malate dehydrogenase	GO:0003824,GO:0005975,GO:0006099,GO:0006108,GO:0016491,GO:0016616,GO:0019752,GO:0030060,GO:0055114	K00024	C
FPS10_21390	sulfite exporter TauE/SafE family protein	GO:0016020,GO:0016021	K07090	R
FPS10_21395	hypothetical protein			
FPS10_21400	CoA ester lyase		K14451	G
FPS10_21405	hypothetical protein			S
FPS10_21410	DUF1737 domain-containing protein			S
FPS10_21415	MaoC family dehydratase		K14449	I
FPS10_21420	divalent metal cation transporter	GO:0005215,GO:0005886,GO:0006810,GO:0006811,GO:0015293,GO:0016020,GO:0016021,GO:0030001,GO:0046872,GO:0055085	K03322	P
FPS10_21425	manganese-binding transcriptional regulator MntR		K11924	K
FPS10_21430	succinate dehydrogenase%2C cytochrome b556 subunit	GO:0000104,GO:0006099,GO:0009055,GO:0016020,GO:0016021,GO:0016627,GO:0045281	K00241	C
FPS10_21435	hypothetical protein		K00242	C
FPS10_21440	succinate dehydrogenase flavoprotein subunit	GO:0005886,GO:0006099,GO:0008177,GO:0016020,GO:0016491,GO:0016627,GO:0022900,GO:0050660,GO:0055114	K00239	C
FPS10_21445	hypothetical protein			
FPS10_21450	succinate dehydrogenase iron-sulfur subunit	GO:0006099,GO:0008177,GO:0009055,GO:0016491,GO:0046872,GO:0051536,GO:0051537,GO:0051538,GO:0051539,GO:0055114	K00240	C
FPS10_21455	DUF1989 domain-containing protein		K09967	S
FPS10_21460	tRNA-binding protein		K06878	R
FPS10_21465	pyrroline-5-carboxylate reductase	GO:0004735,GO:0005737,GO:0006561,GO:0008652,GO:0016491,GO:0055114,GO:0055129	K00286	E
FPS10_21470	diacylglyceryl transferase			S
FPS10_21475	accessory factor UbiK family protein		K09806	S
FPS10_21480	prolipoprotein diacylglyceryl transferase	GO:0005886,GO:0008961,GO:0009249,GO:0016020,GO:0016021,GO:0016740,GO:0016757,GO:0042158	K13292	M
FPS10_21485	class I SAM-dependent methyltransferase			S
FPS10_21490	peptidoglycan editing factor PgeF		K05810	S
FPS10_21495	hypothetical protein			
FPS10_21500	AAA family ATPase			L
FPS10_21505	autoinducer synthase		K20249	TQ
FPS10_21510	LuxR family transcriptional regulator	GO:0003677,GO:0006351,GO:0006355	K19733	K
FPS10_21515	hypothetical protein			
FPS10_21520	crotonyl-CoA carboxylase/reductase	GO:0008270,GO:0016491,GO:0043880,GO:0055114	K14446	CR
FPS10_21525	protein meaA	GO:0003824,GO:0004494,GO:0008152,GO:0016853,GO:0016866,GO:0031419,GO:0046872	K14447	I
FPS10_21530	H-NS histone family protein	GO:0003677,GO:0005622,GO:0006355	K03746	R
FPS10_21535	purine-nucleoside phosphorylase	GO:0003824,GO:0004731,GO:0006139,GO:0009116,GO:0016740,GO:0016757,GO:0016763,GO:0042278	K03784	F
FPS10_21540	thymidine kinase	GO:0000166,GO:0004797,GO:0005524,GO:0005737,GO:0006259,GO:0008270,GO:0009157,GO:0016301,GO:0016310,GO:0016740,GO:0046872,GO:0071897	K00857	F
FPS10_21545	GntR family transcriptional regulator	GO:0003677,GO:0003700,GO:0006351,GO:0006355		K
FPS10_21550	M48 family metallopeptidase		K07043	R
FPS10_21555	TIGR02300 family protein			S
FPS10_21560				

FPS10_21565	3-hydroxybenzoate 4-monooxygenase	GO:0004497,GO:0016491,GO:0018662,GO:0055114,GO:007194[illegible]	K03380	HC
FPS10_21570	MarR family transcriptional regulator	GO:0003677,GO:0003700,GO:0006351,GO:0006355		K
FPS10_21575	LysR family transcriptional regulator	GO:0003677,GO:0003700,GO:0006351,GO:0006355		K
FPS10_21580	TRAP transporter large permease	GO:0016020,GO:0016021		G
FPS10_21585	TRAP transporter small permease	GO:0016020,GO:0016021		G
FPS10_21590	TRAP transporter substrate-binding protein	GO:0006810,GO:0030288	K21395	G
FPS10_21595	SDR family oxidoreductase	GO:0008152,GO:0016491,GO:0055114		IQR
FPS10_21600	transketolase family protein		K00615	G
FPS10_21605	transketolase		K00615	G
FPS10_21610	TRAP transporter large permease subunit			Q
FPS10_21615	TRAP transporter small permease	GO:0016020,GO:0016021		G
FPS10_21625	L-idonate 5-dehydrogenase		K00098	ER
FPS10_21630	SDR family oxidoreductase	GO:0008152,GO:0016491,GO:0055114	K00046	IQR
FPS10_21635	mandelate racemase/muconate lactonizing enzyme family protein	GO:0003824,GO:0008152,GO:0046872		MR
FPS10_21640	FadR family transcriptional regulator			K
FPS10_21645	hypothetical protein			
FPS10_21650	aminotransferase class V-fold PLP-dependent enzyme	GO:0003824,GO:0008152		E
FPS10_21655	BioY family transporter		K03523	R
FPS10_21660	energy-coupling factor transporter transmembrane protein EcfT		K16783	P
FPS10_21665	ABC transporter ATP-binding protein	GO:0005524,GO:0008152,GO:0016887	K16784	P
FPS10_21670	L%2CD-transpeptidase family protein		K21470	S
FPS10_21675	acetate/propionate family kinase	GO:0000166,GO:0000287,GO:0005524,GO:0005622,GO:0005737,GO:0006082,GO:0006085,GO:0008152,GO:0008776,GO:0016301,GO:0016310,GO:0016740,GO:0016774,GO:0046872	K00925	C
FPS10_21680	NAD(P)-dependent glycerol-3-phosphate dehydrogenase		K00057	C
FPS10_21685	YihY/virulence factor BrkB family protein		K07058	S
FPS10_21690	3-deoxy-D-manno-octulosonic acid transferase		K02527	M
FPS10_21695	VOC family protein	GO:0008168,GO:0016740,GO:0032259	K04750	S
FPS10_21700	redox-regulated ATPase YchF	GO:0000166,GO:0005524,GO:0005525,GO:0008150,GO:0008152,GO:0016887,GO:0043022,GO:0043023	K06942	J
FPS10_21705	glycosyltransferase family 4 protein			M
FPS10_21710	hypothetical protein			
FPS10_21715	hypothetical protein			
FPS10_21720	AAA family ATPase		K16554	D
FPS10_21725	glycosyltransferase family 4 protein	GO:0008152,GO:0016740		M
FPS10_21730	4'-phosphopantetheinyl transferase superfamily protein		K06133	H
FPS10_21735	condensation protein			Q
FPS10_21740	hybrid non-ribosomal peptide synthetase/type I polyketide synthase			H
FPS10_21745	methyltransferase%2C TIGR04325 family			
FPS10_21750	oligosaccharide flippase family protein			R
FPS10_21755	sugar transferase			M
FPS10_21760	glycosyltransferase family 2 protein	GO:0008152,GO:0016740		R
FPS10_21765	crotonobetainyl-CoA hydratase		K08299	I
FPS10_21770	acetate--CoA ligase family protein			C
FPS10_21775	acyl-CoA dehydrogenase	GO:0003995,GO:0008152,GO:0016491,GO:0016627,GO:0050660,GO:0055114		I
FPS10_21780	NAD(P)-dependent oxidoreductase	GO:0003824,GO:0008152,GO:0050662		MG
FPS10_21785	glycosyl transferase	GO:0008152,GO:0016740,GO:0016757		M
FPS10_21790	NAD-dependent epimerase/dehydratase family protein	GO:0003824,GO:0008152,GO:0016787,GO:0046507,GO:0050662	K06118	MG
FPS10_21795	hypothetical protein			
FPS10_21800	hypothetical protein			
FPS10_21805	hypothetical protein			
FPS10_21810	peptidase C14%2C caspase catalytic subunit p20	GO:0004197,GO:0006508		R
FPS10_21815	M15 family metallopeptidase			
FPS10_21820	OmpA family protein	GO:0009279,GO:0016020,GO:0016021		M
FPS10_21825	protein kinase			RTKL
FPS10_21830	serine/threonine-protein phosphatase	GO:0003824,GO:0004722,GO:0006470		T
FPS10_21835	type VI secretion system-associated protein TagF		K11890	S
FPS10_21840	type VI secretion system membrane subunit TssM	GO:0016020,GO:0016021	K11891	S
FPS10_21845	type VI secretion system protein TssL		K11892	N
FPS10_21850	type VI secretion system baseplate subunit TssK		K11893	S
FPS10_21855	type VI secretion system-associated FHA domain protein TagH		K11913	T
FPS10_21860	hypothetical protein			

FPS10_21865	type VI secretion system tip protein VgrG		K11904	S
FPS10_21870	type VI secretion system ATPase TssH	GO:0000166,GO:0005524,GO:0019538	K11907	O
FPS10_21875	type VI secretion system baseplate subunit TssG		K11895	S
FPS10_21880	type VI secretion system baseplate subunit TssF		K11896	S
FPS10_21885	type VI secretion system baseplate subunit TssE		K11897	S
FPS10_21890	virulence protein SciE type		K11898	R
FPS10_21895	type VI secretion system tube protein Hcp		K11903	S
FPS10_21900	type VI secretion system contractile sheath large subunit		K11900	S
FPS10_21905	type VI secretion system contractile sheath small subunit		K11901	S
FPS10_21910	type VI secretion system protein TssA		K11902	S
FPS10_21915	precorrin-4 C(11)-methyltransferase	GO:0006779,GO:0008152,GO:0008168,GO:0009236,GO:0016740,GO:0032259,GO:0043115,GO:0046026,GO:0055114	K05936	H
FPS10_21920	precorrin-3B C(17)-methyltransferase	GO:0008152,GO:0008168,GO:0009236,GO:0032259	K13541	H
FPS10_21925	precorrin-2 C(20)-methyltransferase	GO:0006779,GO:0008152,GO:0008168,GO:0008757,GO:0009236,GO:0016740,GO:0030788,GO:0032259,GO:0043115,GO:0055114	K03394	H
FPS10_21930	bifunctional cobalt-precorrin-7 (C(5))-methyltransferase/cobalt-precorrin-6B (C(15))-methyltransferase	GO:0006479,GO:0008152,GO:0008168,GO:0008276,GO:0009236,GO:0016740,GO:0032259,GO:0046025	K00595	H
FPS10_21935	precorrin-8X methylmutase	GO:0009236,GO:0016993	K06042	H
FPS10_21940	sirohydrochlorin chelatase	GO:0009236,GO:0016852	K03795	S
FPS10_21945	hypothetical protein			
FPS10_21950	DUF4198 domain-containing protein		K02009	P
FPS10_21955	cobalt transporter CbiM	GO:0000041,GO:0016020,GO:0016021	K02007	P
FPS10_21960	cobalt ABC transporter permease	GO:0016020,GO:0016021	K16915	
FPS10_21965	cobalt ECF transporter T component CbiQ	GO:0005887,GO:0006824,GO:0016020,GO:0016021,GO:0043190	K02008	P
FPS10_21970	ABC transporter ATP-binding protein	GO:0000166,GO:0005524,GO:0008152,GO:0015415,GO:0016787,GO:0016887,GO:0035435	K02006	P
FPS10_21975	metal-sensing transcriptional repressor	GO:0003677,GO:0006355,GO:0046872	K07807	S
FPS10_21980	altronate dehydratase		K01685	G
FPS10_21985	MarR family transcriptional regulator			K
FPS10_21990	efflux RND transporter periplasmic adaptor subunit	GO:0016020,GO:0055085		M
FPS10_21995	efflux RND transporter permease subunit	GO:0005215,GO:0006810,GO:0016020,GO:0016021		V
FPS10_22000	inositol monophosphatase			G
FPS10_22005	FAD-binding oxidoreductase	GO:0016491,GO:0055114		E
FPS10_22010	ABC transporter permease subunit		K11074	E
FPS10_22015	ABC transporter permease subunit	GO:0005886,GO:0006810,GO:0016020,GO:0016021	K11075	E
FPS10_22020	ABC transporter ATP-binding protein	GO:0000166,GO:0005215,GO:0005524,GO:0005886,GO:0006810,GO:0008152,GO:0015417,GO:0015846,GO:0016020,GO:0016787,GO:0016820,GO:0016887,GO:0043190,GO:0055085,GO:1902047	K11076	E
FPS10_22025	polyamine ABC transporter substrate-binding protein	GO:0006810,GO:0015846,GO:0019808,GO:0042597	K11073	E
FPS10_22030	GntR family transcriptional regulator	GO:0003677,GO:0003700,GO:0006351,GO:0006355		K
FPS10_22035	glutamine synthetase		K01915	E
FPS10_22040	aspartate aminotransferase family protein	GO:0003824,GO:0008152,GO:0008483,GO:0016740,GO:0030170	K12256	H
FPS10_22045	FAD-binding oxidoreductase	GO:0016491,GO:0055114	K09471	E
FPS10_22050	DNA polymerase IV	GO:0000287,GO:0003677,GO:0003684,GO:0003887,GO:0005737,GO:0006260,GO:0006261,GO:0006281,GO:0006974,GO:0016740,GO:0016779,GO:0046872,GO:0071897	K02346	L
FPS10_22055	methylglyoxal synthase	GO:0008929,GO:0016829,GO:0019242	K01734	G
FPS10_22060	ABC transporter permease subunit		K15552	P
FPS10_22065	ABC transporter ATP-binding protein	GO:0000166,GO:0005524,GO:0005886,GO:0006810,GO:0008152,GO:0015411,GO:0015734,GO:0015837,GO:0016020,GO:0016787,GO:0016887,GO:0043190	K10831	P
FPS10_22070	taurine ABC transporter substrate-binding protein		K15551	P
FPS10_22075	LysR family transcriptional regulator	GO:0003677,GO:0003700,GO:0006351,GO:0006355	K03566	K
FPS10_22080	PLP-dependent aminotransferase family protein	GO:0003677,GO:0003700,GO:0003824,GO:0006351,GO:0006355,GO:0009058,GO:0030170	K00375	KE
FPS10_22085	phospholipid carrier-dependent glycosyltransferase			M
FPS10_22090	sulfoacetaldehyde acetyltransferase	GO:0000287,GO:0003824,GO:0005737,GO:0016740,GO:0016746,GO:0019529,GO:0030976,GO:0050487	K03852	EH
FPS10_22095	putative sulfate exporter family transporter	GO:0005886,GO:0016020,GO:0016021		S
FPS10_22100	helix-turn-helix domain-containing protein	GO:0003677,GO:0043565		K
FPS10_22105	FAD-dependent oxidoreductase	GO:0016491,GO:0016740,GO:0055114	K00315	E
FPS10_22110	trimethylamine methyltransferase family protein	GO:0008168,GO:0015948,GO:0016740,GO:0032259	K14083	H
FPS10_22115	FAD-dependent oxidoreductase	GO:0016491,GO:0016740,GO:0055114		E
FPS10_22120	Lrp/AsnC family transcriptional regulator		K05800	K
FPS10_22125	decarboxylase		K01578	
FPS10_22130	transporter	GO:0016020,GO:0016021	K07793	S
FPS10_22135	hypothetical protein			

FPS10_22140	tripartite tricarboxylate transporter substrate binding protein	GO:0030288		S
FPS10_22145	Cu(I)-responsive transcriptional regulator	GO:0003677,GO:0003700,GO:0005507,GO:0006355,GO:0045893	K19591	K
FPS10_22150	copper-translocating P-type ATPase	GO:0000166,GO:0005886,GO:0006812,GO:0008152,GO:0016020,GO:0016021,GO:0016787,GO:0019829,GO:0030001,GO:0046872,GO:0098655	K17686	P
FPS10_22155	copper chaperone		K07213	P
FPS10_22160	GntR family transcriptional regulator	GO:0003677,GO:0003700,GO:0006351,GO:0006355	K22293	K
FPS10_22165	sugar kinase	GO:0016301,GO:0016310,GO:0016740,GO:0016773	K00874	G
FPS10_22170	hypothetical protein			
FPS10_22175	EAL domain-containing protein			NT
FPS10_22180	phosphate/phosphite/phosphonate ABC transporter substrate-binding protein		K02044	P
FPS10_22185	substrate-binding domain-containing protein	GO:0003677,GO:0003700,GO:0006351,GO:0006355	K02529	K
FPS10_22190	PhzF family phenazine biosynthesis protein	GO:0003824,GO:0009058	K06998	R
FPS10_22195	hypothetical protein			
FPS10_22200	DUF1330 domain-containing protein			S
FPS10_22205	TerB family tellurite resistance protein			S
FPS10_22210	TerB family tellurite resistance protein			S
FPS10_22215	PhnD/SsuA/transferrin family substrate-binding protein			P
FPS10_22220	amino acid ABC transporter ATP-binding protein	GO:0000166,GO:0003333,GO:0005524,GO:0008152,GO:0015424,GO:0016887		E
FPS10_22225	transporter substrate-binding domain-containing protein		K02030	ET
FPS10_22230	ABC transporter permease subunit	GO:0005886,GO:0006810,GO:0016020,GO:0016021	K02029	E
FPS10_22235	ABC transporter permease subunit	GO:0005215,GO:0005886,GO:0006810,GO:0016020,GO:0016021	K02029	E
FPS10_22240	glutamine synthetase	GO:0003824,GO:0004356,GO:0006542,GO:0006807,GO:0016874	K01915	E
FPS10_22245	type 1 glutamine amidotransferase			F
FPS10_22250	glutamine synthetase	GO:0003824,GO:0004356,GO:0006542,GO:0006807,GO:0016874	K01915	E
FPS10_22255	aminotransferase class I/II-fold pyridoxal phosphate-dependent enzyme			M
FPS10_22260	LysR family transcriptional regulator	GO:0003677,GO:0003700,GO:0006351,GO:0006355,GO:0016020,GO:0016021		K
FPS10_22265	polyhydroxyalkanoate synthesis repressor PhaR			S
FPS10_22270	phasin%2C PhaP			
FPS10_22275	class I poly(R)-hydroxyalkanoic acid synthase	GO:0005737,GO:0016746,GO:0042619	K03821	I
FPS10_22280	polyhydroxyalkanoate depolymerase	GO:0008152,GO:0016787,GO:0050526	K05973	I
FPS10_22285	alpha/beta hydrolase	GO:0008152,GO:0016787		R
FPS10_22290	VOC family protein	GO:0051213,GO:0055114	K07032	R
FPS10_22295	alpha/beta fold hydrolase			I
FPS10_22300	alpha/beta hydrolase			R
FPS10_22305	pyridoxal phosphate-dependent aminotransferase	GO:0003824,GO:0008152,GO:0008483,GO:0009058,GO:0016740,GO:0030170	K00817	E
FPS10_22310	hypothetical protein			
FPS10_22315	aminotransferase class V-fold PLP-dependent enzyme	GO:0003824,GO:0008152,GO:0008483,GO:0016740	K00830	E
FPS10_22320	protein tyrosine phosphatase			T
FPS10_22325	glycosyltransferase	GO:0008152,GO:0016740		M
FPS10_22330	folate-binding protein YgfZ		K06980	R
FPS10_22335	fructosamine kinase family protein	GO:0016301,GO:0016310,GO:0016740		G
FPS10_22340	elongation factor P		K02356	J
FPS10_22345	elongation factor P			
FPS10_22350	hypothetical protein			R
FPS10_22355	TolC family outer membrane protein	GO:0005215,GO:0006810,GO:0019867	K12340	MU
FPS10_22360	protein-L-isoaspartate O-methyltransferase	GO:0004719,GO:0006464,GO:0006479,GO:0008168,GO:0016740,GO:0032259	K00573	O
FPS10_22365	hypothetical protein			
FPS10_22370	urea ABC transporter ATP-binding subunit UrtE	GO:0005524,GO:0008152,GO:0016887	K11963	E
FPS10_22375	urea ABC transporter ATP-binding protein UrtD	GO:0005524,GO:0008152,GO:0016887	K11962	R
FPS10_22380	urea ABC transporter permease subunit UrtC	GO:0005215,GO:0005886,GO:0006810,GO:0016020,GO:0016021	K11961	E
FPS10_22385	urea ABC transporter permease subunit UrtB	GO:0005215,GO:0005886,GO:0006810,GO:0016020,GO:0016021	K11960	E
FPS10_22390	urea ABC transporter substrate-binding protein		K11959	E
FPS10_22395	phage holin family protein			
FPS10_22400	hypothetical protein			
FPS10_22405	AI-2E family transporter	GO:0016020,GO:0016021		R
FPS10_22410	ABC transporter permease	GO:0005886,GO:0006810,GO:0016020,GO:0016021	K15599	P
FPS10_22415	ABC transporter ATP-binding protein	GO:0000166,GO:0005524,GO:0008152,GO:0016887	K15600	P
FPS10_22420	ABC transporter ATP-binding protein	GO:0009228	K15598	P
FPS10_22425	bifunctional hydroxymethylpyrimidine kinase/phosphomethylpyrimidine kinase	GO:0000166,GO:0005524,GO:0008902,GO:0008972,GO:0009228,GO:0016301,GO:0016310,GO:0016740	K00941	H

FPS10_22430	thiamine phosphate synthase	GO:0000287,GO:0003824,GO:0004789,GO:0009228,GO:0009229,GO:0016740,GO:0046872	K00788	H
FPS10_22435	hydroxyethylthiazole kinase		K00878	H
FPS10_22440	CoA transferase	GO:0003824,GO:0008152		C
FPS10_22445	malonyl-CoA synthase	GO:0003824,GO:0008152	K18661	IQ
FPS10_22450	C4-dicarboxylate ABC transporter substrate-binding protein	GO:0006810,GO:0030288		G
FPS10_22455	TRAP transporter small permease	GO:0016020,GO:0016021		Q
FPS10_22460	TRAP transporter large permease	GO:0016020,GO:0016021		G
FPS10_22465	sn-glycerol-3-phosphate ABC transporter ATP-binding protein UgpC	GO:0000166,GO:0005215,GO:0005524,GO:0006810,GO:0008152,GO:0016820,GO:0016887,GO:0043190,GO:0055085	K10191	G
FPS10_22470	IclR family transcriptional regulator	GO:0003677,GO:0006351,GO:0006355		K
FPS10_22475	extracellular solute-binding protein		K02027	G
FPS10_22480	sugar ABC transporter permease		K02025	G
FPS10_22485	carbohydrate ABC transporter permease		K02026	G
FPS10_22490	alpha-galactosidase	GO:0003824,GO:0004557,GO:0005975,GO:0008152,GO:0016787,GO:0016798,GO:0052692	K07407	G
FPS10_22495	SDR family oxidoreductase		K22215	IQR
FPS10_22500	2-dehydro-3-deoxygalactonokinase	GO:0008671,GO:0016301,GO:0016310,GO:0016740,GO:0034194,GO:0046835	K00883	G
FPS10_22505	2-dehydro-3-deoxy-6-phosphogalactonate aldolase	GO:0003824,GO:0008152,GO:0008674,GO:0016829	K01631	G
FPS10_22510	SMP-30/gluconolactonase/LRE family protein			G
FPS10_22515	beta-galactosidase		K12308	G
FPS10_22520	galactose mutarotase	GO:0003824,GO:0004034,GO:0005975,GO:0016853,GO:0030246	K01785	G
FPS10_22525	glutamate--tRNA ligase	GO:0000049,GO:0000166,GO:0004812,GO:0004818,GO:0005524,GO:0005737,GO:0006412,GO:0006418,GO:0006424,GO:0016874,GO:0016876,GO:0043039	K01885	J
FPS10_22530	metallopeptidase family protein			S
FPS10_22535	MBL fold metallo-hydrolase			R
FPS10_22540	1-phosphofructokinase family hexose kinase	GO:0000166,GO:0005524,GO:0005975,GO:0016301,GO:0016310,GO:0016740,GO:0016773	K16370	G
FPS10_22545	arsenate reductase (glutaredoxin)		K00537	P
FPS10_22550	bifunctional (p)ppGpp synthetase/guanosine-3'%2C5'-bis(diphosphate) 3'-pyrophosphohydrolase	GO:0008152,GO:0016787		TK
FPS10_22555	magnesium transporter		K06213	P
FPS10_22560	5-aminolevulinate synthase			
FPS10_22565	NAD+ synthase		K01916	H
FPS10_22570	2-isopropylmalate synthase			S
FPS10_22575	2-isopropylmalate synthase	GO:0003824,GO:0003852,GO:0008652,GO:0009082,GO:0009098,GO:0016740,GO:0016746,GO:0019752,GO:0046912	K01649	E
FPS10_22580	YdcF family protein			S
FPS10_22585	rod shape-determining protein	GO:0000902	K03569	D
FPS10_22590	rod shape-determining protein MreC	GO:0008360,GO:0016020,GO:0016021	K03570	M
FPS10_22595	rod shape-determining protein MreD		K03571	
FPS10_22600	penicillin-binding protein 2	GO:0008152,GO:0008658,GO:0008955,GO:0016020,GO:0016021,GO:0016740,GO:0016757	K05515	M
FPS10_22605	rod shape-determining protein RodA	GO:0007049,GO:0008360,GO:0016020,GO:0016021,GO:0051301	K05837	D
FPS10_22610	glyoxylate/hydroxypyruvate reductase A	GO:0051287,GO:0055114	K12972	HE
FPS10_22615	class I SAM-dependent methyltransferase			
FPS10_22620				
FPS10_22625	DUF3466 family protein			S
FPS10_22630	hypothetical protein			
FPS10_22635	cytochrome c	GO:0009055,GO:0020037		C
FPS10_22640	hypothetical protein		K11751	F
FPS10_22645	hypothetical protein			
FPS10_22650	hypothetical protein			
FPS10_22655	hypothetical protein			
FPS10_22660	hypothetical protein			
FPS10_22665	AlpA family phage regulatory protein		K07733	K
FPS10_22670	hypothetical protein			
FPS10_22675	tyrosine-type recombinase/integrase			L
FPS10_22680	hypothetical protein	GO:0003824,GO:0006596	K01581	E
FPS10_22685	N-acetyltransferase			R
FPS10_22690	homospermidine synthase	GO:0016020,GO:0016021,GO:0016491,GO:0016740,GO:0047296,GO:0055114	K00808	Q
FPS10_22695	monooxygenase			
FPS10_22700	LysR family transcriptional regulator		K21703	K
FPS10_22705	class 1 fructose-bisphosphatase	GO:0000287,GO:0005737,GO:0005975,GO:0006094,GO:0016031,GO:0016311,GO:0016787,GO:0042132,GO:0042578,GO:0046872	K03841	G

FPS10_22710	phosphoribulokinase	GO:0000166,GO:0005524,GO:0005975,GO:0008152,GO:0008974,GO:0016301,GO:0016310,GO:0016740	K00855	C
FPS10_22715	transketolase	GO:0003824,GO:0004802,GO:0008152,GO:0016740,GO:0046872	K00615	G
FPS10_22720	fructose-bisphosphate aldolase class II	GO:0003824,GO:0004332,GO:0005975,GO:0006096,GO:0008270,GO:0016829,GO:0016832,GO:0046872	K01624	G
FPS10_22725	ribulose-phosphate 3-epimerase	GO:0003824,GO:0004750,GO:0005975,GO:0006098,GO:0008152,GO:0016853,GO:0016857,GO:0046872	K01783	G
FPS10_22730	HAD-IA family hydrolase	GO:0005975,GO:0008152,GO:0008967,GO:0016311,GO:0016787,GO:0046295,GO:0046872	K01091	R
FPS10_22735	HAD-IA family hydrolase	GO:0008152,GO:0016787		R
FPS10_22740	LysR family transcriptional regulator		K21703	K
FPS10_22745	form I ribulose bisphosphate carboxylase large subunit	GO:0000287,GO:0004497,GO:0015977,GO:0016491,GO:0016829,GO:0016984,GO:0019253,GO:0046872,GO:0055114	K01601	G
FPS10_22750	ribulose bisphosphate carboxylase small subunit	GO:0008152,GO:0016829,GO:0016984	K01602	C
FPS10_22755	CbbQ/NirQ/NorQ/GpvN family protein	GO:0005524,GO:0008152,GO:0016887	K04748	R
FPS10_22760	VWA domain-containing protein	GO:0003674,GO:0019538		P
FPS10_22765	hypothetical protein			
FPS10_22770	ester cyclase			R
FPS10_22775	D-lyxose/D-mannose family sugar isomerase			R
FPS10_22780	diguanylate cyclase	GO:0008152,GO:0016787,GO:0071111		T
FPS10_22785	heme NO-binding protein	GO:0020037		
FPS10_22790	trimethylamine methyltransferase family protein		K14083	H
FPS10_22795	HAD family hydrolase		K01091	R
FPS10_22800	DUF3572 family protein			
FPS10_22805	diguanylate cyclase	GO:0000160,GO:0005622	K02488	T
FPS10_22810	periplasmic heavy metal sensor			S
FPS10_22815	hypothetical protein			
FPS10_22820	RNA polymerase sigma factor	GO:0003677,GO:0003700,GO:0003899,GO:0006351,GO:0006352,GO:0006355,GO:0016740,GO:0016779,GO:0016987		K
FPS10_22825	hypothetical protein			TZDR
FPS10_22830	DUF983 domain-containing protein	GO:0016020,GO:0016021		S
FPS10_22835	NUDIX hydrolase			LR
FPS10_22840	EamA/RhaT family transporter	GO:0016020,GO:0016021		
FPS10_22845	hypothetical protein			
FPS10_22850	aldo/keto reductase			C
FPS10_22855	fumarylacetoacetase		K01555	Q
FPS10_22860	DUF1636 domain-containing protein			S
FPS10_22865	cobyric acid synthase	GO:0003824,GO:0006541,GO:0009236,GO:0015420,GO:0015889,GO:0035461	K02232	H
FPS10_22870	cob(I)yrinic acid a%2Cc-diamide adenosyltransferase	GO:0005524,GO:0008817,GO:0009236,GO:0016740	K19221	H
FPS10_22875	cobaltochelatase subunit CobN	GO:0009058,GO:0009236,GO:0051116	K02230	H
FPS10_22880	GNAT family N-acetyltransferase	GO:0008080,GO:0008152,GO:0016740,GO:0016746	K03829	R
FPS10_22885	cobalamin biosynthesis protein CobW	GO:0009236	K02234	R
FPS10_22890	cobalt-precorrin-6A reductase		K05895	H
FPS10_22895	cobalt-precorrin-5B (C(1))-methyltransferase	GO:0008168,GO:0009236,GO:0016740,GO:0032259,GO:0046140	K02188	H
FPS10_22900	uroporphyrinogen-III C-methyltransferase	GO:0006779,GO:0008152,GO:0008168,GO:0016740,GO:0032259,GO:0043115,GO:0055114	K02303	H
FPS10_22905	cobyrinate a%2Cc-diamide synthase	GO:0000166,GO:0003824,GO:0005524,GO:0006541,GO:0009236,GO:0016829,GO:0016874,GO:0042242,GO:0043802	K02224	H
FPS10_22910	lytic murein transglycosylase			M
FPS10_22915	chromosome segregation protein SMC	GO:0000166,GO:0003677,GO:0005524,GO:0005737,GO:0006260,GO:0007059,GO:0007062,GO:0030261	K03529	D
FPS10_22920	hypothetical protein	GO:0016020,GO:0016021		
FPS10_22925	formate transporter FocA	GO:0005215,GO:0006810,GO:0015499,GO:0015724,GO:0016020,GO:0016021	K06212	P
FPS10_22930	pyruvate formate lyase-activating protein	GO:0003824,GO:0005737,GO:0016491,GO:0016829,GO:0043365,GO:0046872,GO:0051536,GO:0051539,GO:0055114	K04069	O
FPS10_22935	formate C-acetyltransferase	GO:0003824,GO:0005737,GO:0005975,GO:0008152,GO:0008861,GO:0016740,GO:0016746	K00656	C
FPS10_22940				
FPS10_22945	transcriptional regulator		K07167	T
FPS10_22950	sigma-70 family RNA polymerase sigma factor	GO:0003677,GO:0003700,GO:0006351,GO:0006352,GO:0006355,GO:0016740,GO:0016779,GO:0016987		K
FPS10_22955	DUF3833 domain-containing protein			
FPS10_22960	DUF3833 domain-containing protein			
FPS10_22965	sodium:galactoside symporter	GO:0016020,GO:0016021		G
FPS10_22970	hypothetical protein			
FPS10_22975	class I SAM-dependent methyltransferase	GO:0008610	K00574	M
FPS10_22980	FAD-dependent oxidoreductase	GO:0016491,GO:0055114		R

FPS10_22985	DUF1365 domain-containing protein		K09701	S
FPS10_22990	SDR family NAD(P)-dependent oxidoreductase	GO:0008152,GO:0016491,GO:0055114		R
FPS10_22995	glutathione peroxidase	GO:0004601,GO:0004602,GO:0006979,GO:0016491,GO:0055114,GO:0098869	K00432	O
FPS10_23000	AraC family transcriptional regulator			K
FPS10_23005	FAD-binding protein	GO:0016491,GO:0055114		C
FPS10_23010	4Fe-4S dicluster domain-containing protein			C
FPS10_23015	oxidoreductase			P
FPS10_23020	D-galactonate dehydratase family protein	GO:0003824,GO:0008152,GO:0009063	K08323	MR
FPS10_23025	Gfo/Idh/MocA family oxidoreductase			R
FPS10_23030	GntR family transcriptional regulator		K22293	K
FPS10_23035	NAD(P)-dependent oxidoreductase			I
FPS10_23040	heme-binding protein			R
FPS10_23045	SDR family oxidoreductase	GO:0008152,GO:0016491,GO:0055114		IQR
FPS10_23050	cupin domain-containing protein			S
FPS10_23055	DUF4962 domain-containing protein			
FPS10_23060	extracellular solute-binding protein		K17318	G
FPS10_23065	carbohydrate ABC transporter permease	GO:0005886,GO:0006810,GO:0016020,GO:0016021	K17320	G
FPS10_23070	sugar ABC transporter permease		K17319	G
FPS10_23075	sn-glycerol-3-phosphate ABC transporter ATP-binding protein UgpC	GO:0000166,GO:0005215,GO:0005524,GO:0006810,GO:0008152,GO:0015430,GO:0015794,GO:0016787,GO:0016820,GO:0016887,GO:0043190,GO:0055085	K10112	G
FPS10_23080	GntR family transcriptional regulator		K22293	K
FPS10_23085	FadR family transcriptional regulator			K
FPS10_23090	hypothetical protein			V
FPS10_23095	malonyl-CoA synthase	GO:0001676,GO:0003824,GO:0004467,GO:0008152,GO:0016874	K18661	IQ
FPS10_23100	decarboxylase		K01578	
FPS10_23105	hypothetical protein		K09780	S
FPS10_23110	fumarylacetoacetate hydrolase family protein	GO:0003824,GO:0008152,GO:0016787		Q
FPS10_23115	malonic semialdehyde reductase	GO:0016491,GO:0055114	K09019	C
FPS10_23120	OsmC family protein	GO:0006979		O
FPS10_23125	C4-dicarboxylate ABC transporter			G
FPS10_23130	TRAP transporter small permease			Q
FPS10_23135	TRAP transporter large permease			G
FPS10_23140	hydroxyquinol 1%2C2-dioxygenase		K04098	Q
FPS10_23145	amidohydrolase family protein	GO:0008152,GO:0016787		R
FPS10_23150	GntR family transcriptional regulator	GO:0003677,GO:0003700,GO:0006351,GO:0006355		K
FPS10_23155	C4-dicarboxylate ABC transporter substrate-binding protein			Q
FPS10_23160	TRAP transporter small permease	GO:0016020,GO:0016021		G
FPS10_23165	TRAP transporter large permease	GO:0016020,GO:0016021		G
FPS10_23170	heme-binding protein			R
FPS10_23175	cupin domain-containing protein			S
FPS10_23180	hypothetical protein			
FPS10_23185	TAXI family TRAP transporter solute-binding subunit		K07080	R
FPS10_23190	TRAP transporter fused permease subunit	GO:0016020,GO:0016021		R
FPS10_23195	cupin domain-containing protein			
FPS10_23200	hypothetical protein			
FPS10_23205	DeoR/GlpR transcriptional regulator			KG
FPS10_23210	MFS transporter	GO:0016020,GO:0016021,GO:0055085		
FPS10_23215	HD domain-containing protein			TK
FPS10_23225	ABC transporter permease	GO:0005886,GO:0006810,GO:0016020,GO:0016021	K02033	EP
FPS10_23230	ABC transporter permease	GO:0005886,GO:0006810,GO:0016020,GO:0016021	K02034	EP
FPS10_23235	ABC transporter ATP-binding protein	GO:0000166,GO:0005524,GO:0006810,GO:0008152,GO:0015833,GO:0016887	K02031	R
FPS10_23240	PilZ domain-containing protein			
FPS10_23245	VWA domain-containing protein	GO:0008152,GO:0016851,GO:0016874	K07114	R
FPS10_23250	RNA polymerase sigma factor	GO:0003677,GO:0003700,GO:0006351,GO:0006352,GO:0006355,GO:0016987		K
FPS10_23255	ABC transporter substrate-binding protein	GO:0043190,GO:0055085	K02035	E
FPS10_23260	isopentenyl-diphosphate Delta-isomerase		K01823	I
FPS10_23265	DNA polymerase III subunit epsilon	GO:0003676,GO:0003677,GO:0003887,GO:0004527,GO:0006260,GO:0016020,GO:0016021,GO:0016740,GO:0016779,GO:0071897,GO:0090305	K02342	L
FPS10_23270	response regulator transcription factor	GO:0000160,GO:0005622		TK
FPS10_23275	hypothetical protein			
FPS10_23280	cyclic nucleotide-binding/CBS domain-containing protein		K07182	T
FPS10_23285	cation acetate symporter	GO:0005215,GO:0006810,GO:0016020,GO:0016021,GO:0055085	K14393	R

FPS10_23290	DUF4212 domain-containing protein	GO:0016020,GO:0016021		S
FPS10_23295	adenylate kinase	GO:0000166,GO:0004017,GO:0005524,GO:0005737,GO:0006139,GO:0016301,GO:0016310,GO:0016740,GO:0016776,GO:0019205,GO:0046939	K00939	F
FPS10_23300	acetate--CoA ligase	GO:0000166,GO:0003824,GO:0003987,GO:0005524,GO:0008152,GO:0016208,GO:0016874,GO:0019427,GO:0046872	K01895	I
FPS10_23305	alpha-hydroxy-acid oxidizing protein	GO:0003824,GO:0004460,GO:0010181,GO:0016491,GO:0055114	K00101	C
FPS10_23310	hypothetical protein			
FPS10_23315	hypothetical protein			
FPS10_23320	tryptophan synthase subunit alpha	GO:0000162,GO:0003824,GO:0004834,GO:0006568,GO:0008152,GO:0008652,GO:0009073,GO:0016829	K01695	E
FPS10_23325	cytochrome c family protein	GO:0009055,GO:0020037	K08738	C
FPS10_23330	D-ribose pyranase	GO:0005737,GO:0005975,GO:0005996,GO:0016853,GO:0016872,GO:0019303,GO:0048029	K06726	G
FPS10_23335	substrate-binding domain-containing protein	GO:0003677,GO:0003700,GO:0006351,GO:0006355	K02529	K
FPS10_23340	ribokinase	GO:0004747,GO:0006014,GO:0016301,GO:0016310,GO:0016740,GO:0016773,GO:0046835	K00852	G
FPS10_23345	tryptophan synthase subunit beta		K06001	E
FPS10_23350	hypothetical protein	GO:0016020,GO:0016021		
FPS10_23355	FAD-dependent oxidoreductase		K00315	E
FPS10_23360	pantoate--beta-alanine ligase	GO:0000166,GO:0004592,GO:0005524,GO:0005737,GO:0015940,GO:0016874	K01918	H
FPS10_23365	3-methyl-2-oxobutanoate hydroxymethyltransferase	GO:0003824,GO:0003864,GO:0005737,GO:0008168,GO:0015940,GO:0016740,GO:0032259,GO:0046872	K00606	H
FPS10_23370	hypothetical protein			
FPS10_23375	D-glycerate dehydrogenase	GO:0008152,GO:0016616,GO:0051287,GO:0055114	K00015	CHR
FPS10_23380	aspartyl-trna synthetase			S
FPS10_23385	redox-sensitive transcriptional activator SoxR	GO:0003677,GO:0006355,GO:0006979,GO:0051537	K13639	K
FPS10_23390	VOC family protein			E
FPS10_23395	M3 family metallopeptidase	GO:0004180,GO:0004222,GO:0006508,GO:0008233,GO:0008237,GO:0016787,GO:0046872	K01284	E
FPS10_23400	DUF4399 domain-containing protein			
FPS10_23405	hypothetical protein			
FPS10_23410	amino acid ABC transporter permease	GO:0005215,GO:0005886,GO:0006810,GO:0016020,GO:0016021	K02029	E
FPS10_23415	transporter substrate-binding domain-containing protein		K02030	ET
FPS10_23420	HesA/MoeB/ThiF family protein	GO:0008152,GO:0008641,GO:0016020,GO:0016021		H
FPS10_23425	BapA prefix-like domain-containing protein			
FPS10_23430	TolC family protein		K12543	MU
FPS10_23435	type I secretion system permease/ATPase		K12541	V
FPS10_23440	HlyD family efflux transporter periplasmic adaptor subunit	GO:0005623,GO:0008565,GO:0009306,GO:0016020,GO:0016021	K12542	V
FPS10_23445	S1 family peptidase			E
FPS10_23450	hypothetical protein			
FPS10_23455				
FPS10_23460	hypothetical protein			NU
FPS10_23465	rhomboid family intramembrane serine protease	GO:0004252,GO:0006508,GO:0016020,GO:0016021		R
FPS10_23470	hypothetical protein			
FPS10_23475	inositol monophosphatase	GO:0008934,GO:0046854,GO:0046855	K01092	G
FPS10_23480	LysR family transcriptional regulator	GO:0003677,GO:0003700,GO:0006351,GO:0006355	K03576	K
FPS10_23485	methylenetetrahydrofolate reductase [NAD(P)H]	GO:0004489,GO:0005829,GO:0006555,GO:0008652,GO:0009086,GO:0016491,GO:0035999,GO:0055114	K00297	E
FPS10_23490	hypothetical protein			
FPS10_23495	DUF2235 domain-containing protein			S
FPS10_23500	type III PLP-dependent enzyme	GO:0003824,GO:0004586,GO:0006596,GO:0016829	K01581	E
FPS10_23505	Lrp/AsnC family transcriptional regulator			K
FPS10_23510	acetoin utilization protein AcuC		K04768	BQ
FPS10_23515	NUDIX hydrolase	GO:0008152,GO:0016787		LR
FPS10_23520	DUF1178 family protein			S
FPS10_23525	ceramidase	GO:0006672,GO:0016020,GO:0016021,GO:0016811		
FPS10_23530	metal-dependent hydrolase	GO:0008152,GO:0016787		R
FPS10_23535	nucleoside deaminase	GO:0002100,GO:0003824,GO:0008033,GO:0008251,GO:0008270,GO:0016787,GO:0046872,GO:0052717	K11991	FJ
FPS10_23540	rRNA pseudouridine synthase	GO:0001522,GO:0003723,GO:0009451,GO:0009982,GO:0016853,GO:0016866	K06178	J
FPS10_23545	VCBS repeat-containing protein			
FPS10_23550	2Fe-2S iron-sulfur cluster binding domain-containing protein	GO:0009055,GO:0046872,GO:0051536,GO:0051537	K04755	C
FPS10_23555	hypothetical protein	GO:0003676,GO:0004386,GO:0008152		L
FPS10_23560	peptidoglycan-binding protein			M
FPS10_23565	hypothetical protein			

FPS10_23570	Do family serine endopeptidase		K04771	O
FPS10_23575	DUF2065 domain-containing protein	GO:0016020,GO:0016021	K09937	S
FPS10_23580	protease modulator HflC	GO:0016020,GO:0016021	K04087	O
FPS10_23585	FtsH protease activity modulator HflK	GO:0003674,GO:0016020,GO:0016021	K04088	O
FPS10_23590	glutathione-disulfide reductase	GO:0004362,GO:0005623,GO:0006749,GO:0016491,GO:0016668,GO:0045454,GO:0050660,GO:0050661,GO:0055114,GO:0098869	K00383	C
FPS10_23595	ion transporter		K10716	
FPS10_23605	SulP family inorganic anion transporter		K03321	P
FPS10_23610	FMN-dependent NADH-azoreductase	GO:0008752,GO:0009055,GO:0010181,GO:0016491,GO:0016652,GO:0016661,GO:0055114	K01118	I
FPS10_23615	signal recognition particle protein	GO:0000166,GO:0003723,GO:0003924,GO:0005525,GO:0005737,GO:0006612,GO:0006614,GO:0008152,GO:0008312,GO:0030529,GO:0048500	K03106	U
FPS10_23620	GNAT family N-acetyltransferase			
FPS10_23625	GNAT family N-acetyltransferase			J
FPS10_23630	GNAT family N-acetyltransferase	GO:0005840,GO:0008080,GO:0008152,GO:0016740		J
FPS10_23635	GNAT family N-acetyltransferase	GO:0008080,GO:0008152		J
FPS10_23640	chorismate mutase	GO:0004106,GO:0016853,GO:0046417	K04092	E
FPS10_23645	30S ribosomal protein S16		K02959	J
FPS10_23650	ribosome maturation factor RimM		K02860	J
FPS10_23655	hypothetical protein			
FPS10_23660	tRNA (guanosine(37)-N1)-methyltransferase TrmD	GO:0005737,GO:0006400,GO:0008033,GO:0008168,GO:0009019,GO:0016740,GO:0030488,GO:0032259,GO:0052906	K00554	J
FPS10_23665	EthD family reductase			
FPS10_23670	50S ribosomal protein L19		K02884	J
FPS10_23675	50S ribosomal protein L31	GO:0003735,GO:0005622,GO:0005840,GO:0006412,GO:0030529	K02909	J
FPS10_23680	M42 family peptidase			G
FPS10_23685	GNAT family N-acetyltransferase			V
FPS10_23690	AAA family ATPase		K03496	D
FPS10_23695	hypothetical protein			
FPS10_23700	Lrp/AsnC family transcriptional regulator	GO:0003677,GO:0003700,GO:0005622,GO:0006351,GO:0006355,GO:0043565		K
FPS10_23705	thioredoxin-disulfide reductase	GO:0004791,GO:0005737,GO:0016491,GO:0019430,GO:0055114	K00384	O
FPS10_23710	bifunctional sulfate adenylyltransferase/adenylylsulfate kinase	GO:0000103,GO:0004020,GO:0004781,GO:0005524,GO:0016301,GO:0016310,GO:0016740,GO:0016779	K00958	P
FPS10_23715	hypothetical protein			
FPS10_23720	DUF1150 family protein			S
FPS10_23725	Hsp20 family protein			O
FPS10_23730	DUF465 domain-containing protein			S
FPS10_23735	5-(carboxyamino)imidazole ribonucleotide mutase	GO:0006164,GO:0006189,GO:0016829,GO:0016853,GO:0034023	K01588	F
FPS10_23740	5-(carboxyamino)imidazole ribonucleotide synthase		K01589	F
FPS10_23745	DUF167 domain-containing protein		K09131	S
FPS10_23750	class II histone deacetylase			BQ
FPS10_23755	nitroreductase	GO:0016491,GO:0055114		C
FPS10_23760	hypothetical protein			E
FPS10_23765	DUF333 domain-containing protein		K09712	R
FPS10_23770	DUF1467 family protein	GO:0016020,GO:0016021		S
FPS10_23775	methylmalonyl-CoA epimerase		K05606	E
FPS10_23780	response regulator	GO:0000160,GO:0005622		T
FPS10_23785	aspartate--tRNA ligase	GO:0000166,GO:0003676,GO:0004812,GO:0004815,GO:0005524,GO:0005737,GO:0006412,GO:0006418,GO:0016874	K01876	J
FPS10_23790	SseB family protein			
FPS10_23795	uracil-DNA glycosylase family protein			L
FPS10_23800	helix-turn-helix transcriptional regulator			K
FPS10_23805	permease	GO:0016020,GO:0016021	K07089	R
FPS10_23810	ATP-binding cassette domain-containing protein	GO:0000166,GO:0005524,GO:0008152,GO:0015436,GO:0015776,GO:0016787,GO:0016887	K09689	GM
FPS10_23815	capsule biosynthesis protein	GO:0016020,GO:0016021	K10107	M
FPS10_23820	3-deoxy-8-phosphooctulonate synthase	GO:0003824,GO:0005737,GO:0008152,GO:0008676,GO:0009058,GO:0009103,GO:0016740,GO:0019294	K01627	M
FPS10_23825				
FPS10_23830	hypothetical protein			
FPS10_23835	hypothetical protein			R
FPS10_23840	DUF3604 domain-containing protein			
FPS10_23845	hypothetical protein			
FPS10_23850	hypothetical protein			
FPS10_23855	hypothetical protein			
FPS10_23860	DUF1214 domain-containing protein			S

FPS10_23865	RcnB family protein			S
FPS10_23870	alkaline phosphatase family protein	GO:0003824,GO:0008152		R
FPS10_23875	phospholipase D family protein	GO:0003824,GO:0008152	K06132	I
FPS10_23880	DUF2312 domain-containing protein			S
FPS10_23885	hypothetical protein	GO:0016020,GO:0016021		
FPS10_23890	hypothetical protein		K07516	I
FPS10_23895	TraR/DksA family transcriptional regulator	GO:0008270		T
FPS10_23900	TIGR01244 family phosphatase	GO:0008152,GO:0016787		S
FPS10_23905	hypothetical protein		K02416	N
FPS10_23910	sodium:solute symporter	GO:0000155,GO:0000160,GO:0000166,GO:0005215,GO:0005524,GO:0005622,GO:0006810,GO:0007165,GO:0016020,GO:0016021,GO:0016301,GO:0016310,GO:0016740,GO:0016772,GO:0023014,GO:0055085		ER
FPS10_23915	hypothetical protein	GO:0016020,GO:0016021		
FPS10_23920	response regulator	GO:0000160,GO:0005622		TK
FPS10_23925	helix-turn-helix domain-containing protein	GO:0003677,GO:0043565	K07110	K
FPS10_23930	ABC transporter substrate-binding protein			E
FPS10_23935	branched-chain amino acid ABC transporter permease			E
FPS10_23940	branched-chain amino acid ABC transporter permease	GO:0005215,GO:0005886,GO:0006810,GO:0016020,GO:0016021		E
FPS10_23945	ABC transporter ATP-binding protein	GO:0005524,GO:0008152,GO:0016887	K01995	E
FPS10_23950	ABC transporter ATP-binding protein		K01996	E
FPS10_23955	pyruvate kinase		K00873	G
FPS10_23960	zinc-binding dehydrogenase			CR
FPS10_23965	sulfide/dihydroorotate dehydrogenase-like FAD/NAD-binding protein	GO:0016491,GO:0051536,GO:0055114	K00266	ER
FPS10_23970	pyruvate-flavodoxin oxidoreductase	GO:0003824,GO:0008152,GO:0016491,GO:0016903,GO:0030976,GO:0055114	K03737	C
FPS10_23975	chromate efflux transporter		K07240	P
FPS10_23980	hypothetical protein			S
FPS10_23985	SDR family oxidoreductase	GO:0008152,GO:0008678,GO:0016491,GO:0055114	K00065	IQR
FPS10_23990	zinc-binding dehydrogenase			ER
FPS10_24000	FCD domain-containing protein		K22104	K
FPS10_24005	glucose-6-phosphate isomerase	GO:0004347,GO:0005737,GO:0006094,GO:0006096,GO:0016853	K01810	G
FPS10_24010	6-phosphogluconolactonase	GO:0005975,GO:0006098,GO:0016787,GO:0017057	K01057	G
FPS10_24015	glucose-6-phosphate dehydrogenase	GO:0004345,GO:0005975,GO:0006006,GO:0006098,GO:0016491,GO:0050661,GO:0055114	K00036	G
FPS10_24020	radical SAM protein			R
FPS10_24025	lytic transglycosylase domain-containing protein			M
FPS10_24030	trigger factor			
FPS10_24035	adenylosuccinate lyase	GO:0003824,GO:0004018,GO:0006164,GO:0006189,GO:0009152,GO:0016829,GO:0044208,GO:0070626	K01756	F
FPS10_24040	hypothetical protein			
FPS10_24045	hypothetical protein			
FPS10_24050	(Na+)-NQR maturation NqrM		K05952	S
FPS10_24055	FAD:protein FMN transferase		K03734	H
FPS10_24060	Na(+)-translocating NADH-quinone reductase subunit A		K00346	C
FPS10_24065	NADH:ubiquinone reductase (Na(+)-transporting) subunit B		K00347	C
FPS10_24070	Na(+)-translocating NADH-quinone reductase subunit C		K00348	C
FPS10_24075	NADH:ubiquinone reductase (Na(+)-transporting) subunit D	GO:0005886,GO:0006810,GO:0006811,GO:0006814,GO:0016020,GO:0016021,GO:0016491,GO:0016655,GO:0055114	K00349	C
FPS10_24080	NADH:ubiquinone reductase (Na(+)-transporting) subunit E		K00350	C
FPS10_24085	NADH:ubiquinone reductase (Na(+)-transporting) subunit F	GO:0005886,GO:0006810,GO:0006811,GO:0006814,GO:0009055,GO:0016020,GO:0016021,GO:0016491,GO:0016655,GO:0046872,GO:0051536,GO:0051537,GO:0055114	K00351	C
FPS10_24090	CBS domain-containing protein			T
FPS10_24095	NADH:flavin oxidoreductase	GO:0003824,GO:0008168,GO:0010181,GO:0016491,GO:0019504,GO:0032259,GO:0055114	K22551	R
FPS10_24100	TetR/AcrR family transcriptional regulator	GO:0003677,GO:0006351,GO:0006355		K
FPS10_24105	amidohydrolase			R
FPS10_24110	hypothetical protein			
FPS10_24115	ribulose-phosphate 3-epimerase	GO:0003824,GO:0004750,GO:0005975,GO:0006098,GO:0008152,GO:0016853,GO:0016857,GO:0046872	K01783	G
FPS10_24120	ABC transporter substrate-binding protein		K02051	P
FPS10_24125	ABC transporter ATP-binding protein	GO:0000166,GO:0005524,GO:0008152,GO:0016887	K02049	P
FPS10_24130	ABC transporter permease		K02050	P
FPS10_24135	type I glyceraldehyde-3-phosphate dehydrogenase	GO:0006006,GO:0016491,GO:0016620,GO:0050661,GO:0051287,GO:0055114	K00134	G

FPS10_24140	phasin family protein			
FPS10_24145	50S ribosomal protein L25/general stress protein Ctc		K02897	J
FPS10_24150	aminoacyl-tRNA hydrolase		K01056	J
FPS10_24155	DUF2237 domain-containing protein		K09966	S
FPS10_24160	helix-turn-helix transcriptional regulator			K
FPS10_24165	hypothetical protein			
FPS10_24170	hypothetical protein			
FPS10_24175	tryptophan synthase subunit beta	GO:0000162,GO:0004834,GO:0006568,GO:0008652,GO:0009073,GO:0016829	K01696	E
FPS10_24180	GNAT family N-acetyltransferase	GO:0008080,GO:0008152		R
FPS10_24185	phosphoribosylanthranilate isomerase	GO:0000162,GO:0003824,GO:0004640,GO:0006568,GO:0008152,GO:0008652,GO:0009073,GO:0016853	K01817	E
FPS10_24190	DUF1049 domain-containing protein	GO:0005887		
FPS10_24195	integration host factor subunit beta		K05788	L
FPS10_24200	30S ribosomal protein S1	GO:0003676,GO:0003723,GO:0003735,GO:0005840,GO:0006412,GO:0030529	K02945	J
FPS10_24205	aldo/keto reductase			C
FPS10_24210	(d)CMP kinase		K00945	F
FPS10_24215	3-phosphoshikimate 1-carboxyvinyltransferase	GO:0003824,GO:0003866,GO:0005737,GO:0008652,GO:0009073,GO:0009423,GO:0016740,GO:0016765	K00800	E
FPS10_24220	tRNA (guanosine(46)-N7)-methyltransferase TrmB	GO:0006400,GO:0008033,GO:0008168,GO:0008176,GO:0016740,GO:0032259,GO:0036265	K03439	R
FPS10_24225	methionine adenosyltransferase	GO:0000166,GO:0000287,GO:0004478,GO:0005524,GO:0005737,GO:0006556,GO:0006730,GO:0016740,GO:0046872	K00789	H
FPS10_24230	apolipoprotein N-acyltransferase	GO:0005886,GO:0006497,GO:0006807,GO:0016020,GO:0016021,GO:0016410,GO:0016740,GO:0016746,GO:0016787,GO:0016810,GO:0042158	K03820	M
FPS10_24235	HlyC/CorC family transporter	GO:0003824,GO:0016614,GO:0050660,GO:0055114	K06189	R
FPS10_24240	rRNA maturation RNase YbeY	GO:0004222,GO:0004518,GO:0004519,GO:0004521,GO:0005737,GO:0006364,GO:0006508,GO:0008270,GO:0016787,GO:0042254,GO:0046872,GO:0090305,GO:0090502	K07042	R
FPS10_24245	PhoH family protein	GO:0005524	K06217	T
FPS10_24250	hypothetical protein			
FPS10_24255	tRNA (N6-isopentenyl adenosine(37)-C2)-methylthiotransferase MiaB	GO:0003824,GO:0005506,GO:0005737,GO:0006400,GO:0008033,GO:0009451,GO:0016740,GO:0043412,GO:0046872,GO:0051536,GO:0051539	K06168	J
FPS10_24260	hypothetical protein			
FPS10_24265	hypothetical protein			
FPS10_24270	transcriptional repressor		K09826	P
FPS10_24275	bifunctional 3-hydroxydecanoyl-ACP dehydratase/trans-2-decenoyl-ACP isomerase	GO:0005737,GO:0006629,GO:0006631,GO:0006633,GO:0008693,GO:0016829,GO:0016853,GO:0019171,GO:0047451	K01716	I
FPS10_24280	beta-ketoacyl-ACP synthase I		K00647	IQ
FPS10_24285	SDR family oxidoreductase	GO:0004318,GO:0006633,GO:0016491,GO:0055114	K00208	I
FPS10_24290	aldo/keto reductase			C
FPS10_24295	glucose 1-dehydrogenase	GO:0008152,GO:0016491,GO:0055114	K00059	IQR
FPS10_24300	P1 family peptidase			EQ
FPS10_24305	hypothetical protein			
FPS10_24310	alpha/beta hydrolase			I
FPS10_24315	hypothetical protein			
FPS10_24320	esterase-like activity of phytase family protein			
FPS10_24325	haloacid dehalogenase type II	GO:0008152,GO:0016787,GO:0019120	K01560	R
FPS10_24330	alpha/beta hydrolase	GO:0008152,GO:0016787		R
FPS10_24335	threonine/serine dehydratase	GO:0006520,GO:0030170	K01754	E
FPS10_24340	3-oxoadipate enol-lactonase	GO:0042952,GO:0047570	K01055	R
FPS10_24345	glutathione-dependent disulfide-bond oxidoreductase	GO:0008152,GO:0016740	K11209	O
FPS10_24350	TCR/Tet family MFS transporter	GO:0005215,GO:0016020,GO:0016021,GO:0055085	K08151	G
FPS10_24355	Zn-dependent alcohol dehydrogenase	GO:0008270,GO:0016491,GO:0046872,GO:0055114		C
FPS10_24360	haloacid dehalogenase type II	GO:0008152,GO:0016787,GO:0019120	K01560	R
FPS10_24365	hypothetical protein			
FPS10_24370	nitrogen fixation protein			
FPS10_24375	mandelate racemase/muconate lactonizing enzyme family protein			MR
FPS10_24380	hypothetical protein			
FPS10_24385	hypothetical protein			
FPS10_24390	hypothetical protein			
FPS10_24395	hypothetical protein			
FPS10_24400	hypothetical protein			
FPS10_24405	alpha/beta fold hydrolase	GO:0003824,GO:0008152		R

FPS10_24410	hypothetical protein			
FPS10_24415	D-alanyl-D-alanine carboxypeptidase/D-alanyl-D-alanine-endopeptidase		K07259	M
FPS10_24420	2-dehydro-3-deoxyphosphooctonate aldolase			
FPS10_24425	lysine--tRNA ligase	GO:0000049,GO:0000166,GO:0004812,GO:0004824,GO:0005524,GO:0005737,GO:0006412,GO:0006418,GO:0006430,GO:0016874	K04566	J
FPS10_24430	GFA family protein	GO:0008152,GO:0016846		S
FPS10_24435	DUF4864 domain-containing protein			
FPS10_24440	SlyX family protein		K03745	
FPS10_24445	histidine--tRNA ligase	GO:0000166,GO:0004812,GO:0004821,GO:0005524,GO:0005737,GO:0006412,GO:0006418,GO:0006427,GO:0016874	K01892	J
FPS10_24450	ATP phosphoribosyltransferase regulatory subunit	GO:0003824,GO:0004812,GO:0005737,GO:0006418	K02502	E
FPS10_24455	ATP phosphoribosyltransferase	GO:0000105,GO:0000166,GO:0003879,GO:0005524,GO:0005737,GO:0008652,GO:0016740,GO:0016757	K00765	E
FPS10_24460	hypothetical protein			M
FPS10_24465	DUF1489 family protein			S
FPS10_24470	adenosylcobalamin-dependent ribonucleoside-diphosphate reductase	GO:0000166,GO:0004748,GO:0005524,GO:0006260,GO:0016491,GO:0031419,GO:0055114	K00525	F
FPS10_24475	hypothetical protein			
FPS10_24480	hypothetical protein			
FPS10_24485	transposase			
FPS10_24490	hypothetical protein		K02529	K
FPS10_24495	SulP family inorganic anion transporter	GO:0006810,GO:0008271,GO:0008272,GO:0015116,GO:0016020,GO:0016021,GO:0055085,GO:1902358	K03321	P
FPS10_24500	S-methyl-5'-thioadenosine phosphorylase		K00772	F
FPS10_24505	DedA family protein			S
FPS10_24510	hypothetical protein			
FPS10_24515	GMC family oxidoreductase	GO:0016491,GO:0016614,GO:0050660,GO:0055114		E
FPS10_24520	glycosyltransferase family 2 protein			
FPS10_24525	hypothetical protein			
FPS10_24530	SRPBCC domain-containing protein	GO:0006950		S
FPS10_24535	MmcB family DNA repair protein			S
FPS10_24540	hypothetical protein			
FPS10_24545	MarR family transcriptional regulator			M
FPS10_24550				
FPS10_24555	DUF3307 domain-containing protein	GO:0016020,GO:0016021		
FPS10_24560	adenylate/guanylate cyclase domain-containing protein		K01768	T
FPS10_24565	hypothetical protein	GO:0005509,GO:0007154,GO:0016021		S
FPS10_24570	cyclic nucleotide-binding domain-containing protein		K03321	P
FPS10_24575	alpha-amylase	GO:0003824,GO:0005975,GO:0005985,GO:0009018	K00690	G
FPS10_24580	hypothetical protein			
FPS10_24585	globin			C
FPS10_24590	thioredoxin TrxC	GO:0005623,GO:0006662,GO:0015035,GO:0045454,GO:0055114	K03672	O
FPS10_24595				
FPS10_24600	Asp/Glu/hydantoin racemase	GO:0006807,GO:0036361		
FPS10_24605	aldo/keto reductase			C
FPS10_24610	NAD(P)-dependent oxidoreductase			I
FPS10_24620	L-idonate 5-dehydrogenase	GO:0008270,GO:0016491,GO:0046183,GO:0046872,GO:0050572,GO:0055114	K00098	ER
FPS10_24625	TRAP transporter large permease	GO:0016020,GO:0016021		G
FPS10_24630	TRAP transporter small permease	GO:0016020,GO:0016021		G
FPS10_24635	TRAP transporter substrate-binding protein			G
FPS10_24640	GntR family transcriptional regulator		K22293	K
FPS10_24645	mannonate dehydratase		K01686	G
FPS10_24650	mannitol dehydrogenase family protein	GO:0003824,GO:0008152,GO:0008866,GO:0016491,GO:0019594,GO:0050662,GO:0055114	K00040	G
FPS10_24655	RNA polymerase factor sigma-32	GO:0003677,GO:0003700,GO:0006351,GO:0006352,GO:0006355,GO:0016987	K03089	K
FPS10_24660	fasciclin domain-containing protein			M
FPS10_24665	cupin domain-containing protein			K
FPS10_24670	DUF4389 domain-containing protein	GO:0016020,GO:0016021		
FPS10_24675	hypothetical protein			
FPS10_24680	Gfo/Idh/MocA family oxidoreductase			R
FPS10_24685	alpha/beta fold hydrolase		K03821	I
FPS10_24690	M3 family oligoendopeptidase	GO:0004222,GO:0006508	K08602	E
FPS10_24695	riboflavin synthase	GO:0004746,GO:0009231,GO:0016491,GO:0016740,GO:0055114	K00793	H
FPS10_24700	capsular biosynthesis protein	GO:0000271,GO:0015774	K07265	M
FPS10_24705	polysaccharide export protein		K01991	M

FPS10_24710	capsular polysaccharide biosynthesis protein	GO:0000271,GO:0015774	K07266	M
FPS10_24715	bifunctional diaminohydroxyphosphoribosylaminopyrimidine deaminase/5-amino-6-(5-phosphoribosylamino)uracil reductase RibD	GO:0003824,GO:0008270,GO:0008703,GO:0008835,GO:0009231,GO:0016491,GO:0016787,GO:0046872,GO:0050661,GO:0055114	K11752	H
FPS10_24720	transcriptional repressor NrdR	GO:0000166,GO:0003677,GO:0005524,GO:0006351,GO:0006355,GO:0008270,GO:0045892,GO:0046872	K07738	K
FPS10_24725	YjbQ family protein			S
FPS10_24730	hypothetical protein			
FPS10_24735	hypothetical protein			
FPS10_24740	hypothetical protein			S
FPS10_24745	RNA polymerase sigma factor RpoD	GO:0001123,GO:0003677,GO:0003700,GO:0005737,GO:0006351,GO:0006352,GO:0006355,GO:0010468,GO:0016987	K03086	K
FPS10_24750	DNA primase	GO:0000287,GO:0003677,GO:0003896,GO:0003899,GO:0006260,GO:0006269,GO:0008270,GO:0016740,GO:0016779,GO:0046872,GO:1990077	K02316	L
FPS10_24755	sarcosine oxidase subunit gamma		K00305	E
FPS10_24760	sarcosine oxidase subunit alpha family protein		K00302	E
FPS10_24765	sarcosine oxidase subunit delta	GO:0008115,GO:0016491,GO:0046653,GO:0055114	K00304	E
FPS10_24770	hypothetical protein			
FPS10_24775	sarcosine oxidase subunit beta family protein	GO:0008115,GO:0016491,GO:0046653,GO:0055114	K00303	E
FPS10_24780	SH3 domain-containing protein			S
FPS10_24785	hypothetical protein			U
FPS10_24790	hypothetical protein			U
FPS10_24795	pilus assembly protein			U
FPS10_24800	hypothetical protein			U
FPS10_24805	hypothetical protein			
FPS10_24810	pirin family protein		K06911	R
FPS10_24815	TetR/AcrR family transcriptional regulator			K
FPS10_24820	hypothetical protein			
FPS10_24825	homoserine dehydrogenase		K00003	E
FPS10_24830	aminodeoxychorismate synthase component I	GO:0008483,GO:0009058,GO:0009396,GO:0016740,GO:00468[illegible]0	K01665	EH
FPS10_24835	Dabb family protein			
FPS10_24840	class II fructose-bisphosphatase		K11532	G
FPS10_24845	single-stranded-DNA-specific exonuclease RecJ		K07462	L
FPS10_24850				
FPS10_24855	DUF1476 domain-containing protein			S
FPS10_24860	TetR/AcrR family transcriptional regulator			K
FPS10_24865	4Fe-4S dicluster domain-containing protein	GO:0009055,GO:0016491,GO:0046872,GO:0051536,GO:0051537,GO:0055114		C
FPS10_24870	hypothetical protein			
FPS10_24875	membrane dipeptidase	GO:0006508,GO:0016787,GO:0016805	K01273	E
FPS10_24880	hypothetical protein			
FPS10_24885	DMT family transporter			GER
FPS10_24890	methylmalonyl Co-A mutase-associated GTPase MeaB	GO:0003924,GO:0005525,GO:0008152	K07588	E
FPS10_24895	hypothetical protein			
FPS10_24900	DUF3108 domain-containing protein			
FPS10_24905	ATP-dependent helicase HrpB		K03579	L
FPS10_24910	ornithine carbamoyltransferase	GO:0004585,GO:0005737,GO:0006520,GO:0006526,GO:0006591,GO:0008652,GO:0016597,GO:0016740,GO:0016743	K00611	E
FPS10_24915	aspartate aminotransferase family protein	GO:0003824,GO:0003992,GO:0005737,GO:0006525,GO:0006526,GO:0008483,GO:0008652,GO:0016740,GO:0030170	K00821	E
FPS10_24920	multidrug ABC transporter permease		K01992	V
FPS10_24925	GcrA cell cycle regulator		K13583	S
FPS10_24930	ABC transporter ATP-binding protein	GO:0000166,GO:0005524,GO:0008152,GO:0016887	K01990	V
FPS10_24935	ABC transporter permease	GO:0005886,GO:0006810,GO:0016020,GO:0016021	K01992	V
FPS10_24940	DUF1127 domain-containing protein			S
FPS10_24945	tetratricopeptide repeat protein			R
FPS10_24950	30S ribosomal protein S12 methylthiotransferase RimO	GO:0003824,GO:0005506,GO:0005737,GO:0009451,GO:00167[illegible]0,GO:0018339,GO:0043412,GO:0046872,GO:0051536,GO:00515 39	K14441	J
FPS10_24955	hypothetical protein			
FPS10_24960	YigZ family protein			S
FPS10_24965	hypothetical protein			
FPS10_24970	hypothetical protein			
FPS10_24975	hypothetical protein			
FPS10_24980	NUDIX hydrolase	GO:0008152,GO:0016787		F
FPS10_24985	GGDEF domain-containing protein			T
FPS10_24990	chaperonin GroEL	GO:0000166,GO:0005524,GO:0005737,GO:0006457,GO:0042026,GO:0051082	K04077	O

FPS10_24995	co-chaperone GroES	GO:0005524,GO:0005737,GO:0006457	K04078	O
FPS10_25000	hypothetical protein			
FPS10_25005	class I SAM-dependent methyltransferase			H
FPS10_25010	manganese-dependent inorganic pyrophosphatase	GO:0004427,GO:0005737,GO:0008152,GO:0016462,GO:0016787,GO:0046872	K15986	C
FPS10_25015	coniferyl aldehyde dehydrogenase		K00154	C
FPS10_25020	TIGR01459 family HAD-type hydrolase	GO:0008152,GO:0016787		G
FPS10_25025	MaoC family dehydratase		K17865	I
FPS10_25030	bifunctional riboflavin kinase/FAD synthetase	GO:0000166,GO:0003919,GO:0005524,GO:0006747,GO:0008531,GO:0009231,GO:0009398,GO:0016301,GO:0016310,GO:0016740,GO:0016779	K11753	H
FPS10_25035	YcgN family cysteine cluster protein		K09160	S
FPS10_25040	low specificity L-threonine aldolase	GO:0003824,GO:0004793,GO:0006520,GO:0006567,GO:0016829	K01620	E
FPS10_25045	class I SAM-dependent methyltransferase	GO:0008168,GO:0016740,GO:0032259		H
FPS10_25050	alpha/beta hydrolase			R
FPS10_25055	2-hydroxychromene-2-carboxylate isomerase	GO:0008152,GO:0015035,GO:0016853,GO:0055114		Q
FPS10_25060	hypothetical protein			M
FPS10_25065	ribose-phosphate pyrophosphokinase	GO:0000166,GO:0000287,GO:0004749,GO:0005524,GO:0005737,GO:0006015,GO:0009156,GO:0009165,GO:0016301,GO:0016310,GO:0016740,GO:0044249,GO:0046872	K00948	FE
FPS10_25070	hypothetical protein	GO:0007155,GO:0030246		
FPS10_25075	alpha-hydroxy-acid oxidizing protein	GO:0003824,GO:0004460,GO:0010181,GO:0016491,GO:0055114	K00101	C
FPS10_25080	dihydroxy-acid dehydratase family protein	GO:0003824,GO:0008152,GO:0016829	K01687	EG
FPS10_25085	FadR family transcriptional regulator			K
FPS10_25090	glycolate oxidase subunit GlcF		K11473	C
FPS10_25095	FAD-binding protein	GO:0003824,GO:0016491,GO:0016614,GO:0050660,GO:0055114	K11472	C
FPS10_25100	FAD-binding protein	GO:0003824,GO:0016491,GO:0016614,GO:0050660,GO:0055114	K00104	C
FPS10_25105	DUF599 domain-containing protein	GO:0016020,GO:0016021		S
FPS10_25110	hypothetical protein			
FPS10_25115	VPLPA-CTERM sorting domain-containing protein			
FPS10_25120	hypothetical protein	GO:0016020,GO:0016021		S
FPS10_25125	DUF4159 domain-containing protein			
FPS10_25130	DUF58 domain-containing protein			R
FPS10_25135	MoxR family ATPase		K03924	R
FPS10_25140	DUF1285 domain-containing protein		K09986	S
FPS10_25145	DUF2798 domain-containing protein			
FPS10_25150	hypothetical protein			
FPS10_25155	NAD(P)-dependent oxidoreductase			I
FPS10_25160	efflux RND transporter periplasmic adaptor subunit	GO:0016020,GO:0055085		M
FPS10_25165	efflux RND transporter permease subunit	GO:0005215,GO:0006810,GO:0016020,GO:0016021		V
FPS10_25170	hypothetical protein			
FPS10_25175	hypothetical protein	GO:0016020,GO:0016021		
FPS10_25180	SPOR domain-containing protein			D
FPS10_25185	uracil phosphoribosyltransferase	GO:0000166,GO:0000287,GO:0003824,GO:0004845,GO:0005525,GO:0006223,GO:0008152,GO:0008655,GO:0016740,GO:0016757,GO:0044206	K00761	F
FPS10_25190	adenosine deaminase		K01488	F
FPS10_25195	3-hydroxyacyl-CoA dehydrogenase	GO:0003824,GO:0003857,GO:0006629,GO:0006631,GO:0006635,GO:0008152,GO:0016491,GO:0055114	K01782	I
FPS10_25200	acetyl-CoA C-acetyltransferase	GO:0003824,GO:0008152,GO:0016740,GO:0016746,GO:0016747	K00626	I
FPS10_25205	glutathione S-transferase	GO:0008152,GO:0016740	K00799	O
FPS10_25210	hypothetical protein			
FPS10_25215	acyl-CoA dehydrogenase			I
FPS10_25220	MerR family DNA-binding transcriptional regulator	GO:0003677,GO:0006355		K
FPS10_25225	MerR family DNA-binding transcriptional regulator	GO:0003677,GO:0006355		K
FPS10_25230	PaaI family thioesterase			Q
FPS10_25235	PaaI family thioesterase			Q
FPS10_25240	MATE family efflux transporter	GO:0006855,GO:0015238,GO:0015297,GO:0016020,GO:0016021,GO:0055085	K03327	V
FPS10_25245	quinone-dependent dihydroorotate dehydrogenase	GO:0003824,GO:0004152,GO:0005737,GO:0005886,GO:0006207,GO:0006221,GO:0006222,GO:0016020,GO:0016491,GO:0016627,GO:0044205,GO:0055114	K00254	F
FPS10_25250	DUF952 domain-containing protein			S
FPS10_25255	multifunctional 2'%2C3'-cyclic-nucleotide 2'-phosphodiesterase/5'-nucleotidase/3'-nucleotidase		K11751	F
FPS10_25260	class I SAM-dependent methyltransferase			
FPS10_25265	SOS response-associated peptidase	GO:0003674,GO:0005575,GO:0008150		S

FPS10_25270	sulfide/dihydroorotate dehydrogenase-like FAD/NAD-binding protein	GO:0006221,GO:0016491,GO:0050660,GO:0051537,GO:0055114	K00528	HC
FPS10_25275	NADPH-dependent glutamate synthase		K00266	ER
FPS10_25280	2-oxoacid:acceptor oxidoreductase subunit alpha		K00174	C
FPS10_25285	2-oxoglutarate oxidoreductase		K00175	C
FPS10_25290	hypothetical protein		K03737	C
FPS10_25295	phosphopentomutase	GO:0000287,GO:0003824,GO:0005737,GO:0006015,GO:0008152,GO:0008973,GO:0009117,GO:0009166,GO:0009264,GO:0016853,GO:0030145,GO:0043094,GO:0046872	K01839	G
FPS10_25300	thymidine phosphorylase	GO:0004645,GO:0006206,GO:0006213,GO:0008152,GO:0009032,GO:0016154,GO:0016740,GO:0016757,GO:0016763	K00758	F
FPS10_25305	cytidine deaminase		K01489	F
FPS10_25310	propionyl-CoA synthetase	GO:0003824,GO:0008152,GO:0016874	K01908	I
FPS10_25315	response regulator			T
FPS10_25320	sigma-54-dependent Fis family transcriptional regulator	GO:0000160,GO:0000166,GO:0003677,GO:0005524,GO:0005622,GO:0006351,GO:0006355,GO:0008134,GO:0043565		T
FPS10_25325	NAD kinase		K00858	G
FPS10_25330	serine hydroxymethyltransferase	GO:0003824,GO:0004372,GO:0005737,GO:0006544,GO:0006545,GO:0006563,GO:0006730,GO:0008652,GO:0016740,GO:0019264,GO:0030170,GO:0035999	K00600	E
FPS10_25335	hypothetical protein			
FPS10_25340	iron-containing alcohol dehydrogenase			C
FPS10_25345	hypothetical protein			
FPS10_25350	aldehyde dehydrogenase family protein	GO:0008152,GO:0016491,GO:0016620,GO:0055114	K00128	C
FPS10_25355	class I SAM-dependent methyltransferase			
FPS10_25360	hypothetical protein			
FPS10_25365	MFS transporter	GO:0005215,GO:0006810,GO:0016020,GO:0016021,GO:00550[illegible]5		G
FPS10_25370	hypothetical protein			
FPS10_25375	hypothetical protein			
FPS10_25380	succinylglutamate desuccinylase/aspartoacylase family protein	GO:0004181,GO:0006508,GO:0008152,GO:0008270,GO:0016787,GO:0016788,GO:0046872	K06987	R
FPS10_25385	30S ribosomal protein S6--L-glutamate ligase	GO:0000166,GO:0000287,GO:0003824,GO:0005524,GO:00064[illegible]2,GO:0006464,GO:0016874,GO:0016881,GO:0030145,GO:0046872	K05844	HJ
FPS10_25390	ATP-dependent zinc protease			O
FPS10_25395	magnesium transporter	GO:0005886,GO:0006810,GO:0006812,GO:0008324,GO:0015095,GO:0015693,GO:0016020,GO:0016021,GO:0046872,GO:0098655,GO:1903830	K06213	P
FPS10_25400	hypothetical protein			
FPS10_25405				
FPS10_25410	hypothetical protein			
FPS10_25415	UDP-N-acetylglucosamine 1-carboxyvinyltransferase	GO:0003824,GO:0005737,GO:0007049,GO:0008360,GO:0008760,GO:0009252,GO:0016740,GO:0016765,GO:0019277,GO:0051301,GO:0071555	K00790	M
FPS10_25420	DUF2948 family protein			
FPS10_25425	histidinol dehydrogenase	GO:0000105,GO:0004399,GO:0008152,GO:0008270,GO:0008652,GO:0016491,GO:0046872,GO:0051287,GO:0055114	K00013	E
FPS10_25430	UPF0262 family protein			S
FPS10_25435	low molecular weight phosphatase family protein			T
FPS10_25440	SHOCT domain-containing protein			
FPS10_25445	translation initiation factor IF-1		K02518	J
FPS10_25450	septum formation protein Maf		K06287	D
FPS10_25455	ribonuclease G	GO:0003723		J
FPS10_25460	DNA gyrase inhibitor YacG		K09862	S
FPS10_25465				
FPS10_25470	AAA family ATPase	GO:0005524		R
FPS10_25475	transposase		K07497	
FPS10_25480	transcriptional regulator/antitoxin MazE			
FPS10_25485	type II toxin-antitoxin system death-on-curing family toxin	GO:0016301,GO:0016310	K07341	R
FPS10_25495	hypothetical protein	GO:0003676,GO:0003677		
FPS10_25500	phosphomannomutase	GO:0000287,GO:0005975,GO:0016868,GO:0046872,GO:00717[illegible]4	K01840	G
FPS10_25505	mannose-1-phosphate guanylyltransferase/mannose-6-phosphate isomerase		K16011	G

Gene ID	Gene	Functions
FPS10_06860	*fliG*	flagellar motor switch protein FliG
FPS10_13635	*motA*	flagellar motor stator protein MotA
FPS10_13650	*fliL*	flagellar basal body protein FliL
FPS10_14440	*flgI*	flagellar basal body P-ring protein FlgI
FPS10_14450	*flgK*	flagellar hook-associated protein FlgK
FPS10_14455	*flgE*	flagellar hook-basal body complex protein
FPS10_15850	*flbT*	flagellar biosynthesis repressor FlbT
FPS10_15855	*flaF*	flagellar biosynthesis regulator FlaF
FPS10_15870	*flgJ*	flagellar biosynthesis protein FlgJ
FPS10_18395	*fliP*	flagellar type III secretion system pore protein FliP
FPS10_18400	*fliM/fliN*	FliM/FliN family flagellar motor switch protein
FPS10_18410	*fliF*	flagellar M-ring protein FliF
FPS10_19195	*fliL*	flagellar basal body-associated protein FliL
FPS10_19200	*flgH*	flagellar basal body L-ring protein FlgH
FPS10_19205	*flgA*	flagellar basal body P-ring formation protein FlgA
FPS10_19210	*flgG*	flagellar basal-body rod protein FlgG
FPS10_19215	*flgF*	flagellar hook-basal body complex protein
FPS10_19220	*fliQ*	flagellar biosynthetic protein FliQ
FPS10_19225	*fliE*	flagellar hook-basal body complex protein FliE
FPS10_19230	*flgC*	flagellar basal body rod protein FlgC
FPS10_19250	*flhB*	flagellar biosynthesis protein FlhB
FPS10_19260	*flhA*	flagellar biosynthesis protein FlhA

Supplementary Table 3. The flagellar related genes in *Pseudoruegeria* sp. M32A2M

Supplementary Table 4. Codon usage in *Pseudoruegeria* sp. M32A2M

	Codon	Amino acid	%	Ratio	Codon	Amino acid	%	Ratio	Codon	Amino acid	%	Ratio	Codon	Amino acid	%	Ratio	
	UUU	Phe (F)	0.79%	0.21	UCU	Ser (S)	0.29%	0.05	UAU	Tyr (Y)	1.06%	0.48	UGU	Cys (C)	0.20%	0.21	**U**
**U**	UUC	Phe (F)	3.04%	0.79	UCC	Ser (S)	1.60%	0.29	UAC	Tyr (Y)	1.16%	0.52	UGC	Cys (C)	0.75%	0.79	**C**
	UUA	Leu (L)	0.04%	0.00	UCA	Ser (S)	0.22%	0.04	UAA	STOP	0.05%	0.16	UGA	STOP	0.22%	0.70	**A**
	UUG	Leu (L)	1.00%	0.10	UCG	Ser (S)	1.78%	0.32	UAG	STOP	0.05%	0.15	UGG	Trp (W)	1.43%	1.00	**G**
	CUU	Leu (L)	1.33%	0.13	CCU	Pro (P)	0.35%	0.07	CAU	His (H)	1.00%	0.50	CGU	Arg (R)	0.81%	0.12	**U**
**C**	CUC	Leu (L)	2.72%	0.27	CCC	Pro (P)	1.60%	0.31	CAC	His (H)	1.02%	0.50	CGC	Arg (R)	3.17%	0.47	**C**
	CUA	Leu (L)	0.17%	0.02	CCA	Pro (P)	0.39%	0.08	CAA	Gln (Q)	0.69%	0.23	CGA	Arg (R)	0.40%	0.06	**A**
	CUG	Leu (L)	4.84%	0.48	CCG	Pro (P)	2.76%	0.54	CAG	Gln (Q)	2.36%	0.77	CGG	Arg (R)	1.91%	0.28	**G**
	AUU	Ile (I)	0.82%	0.16	ACU	Thr (T)	0.27%	0.05	AAU	Aln (N)	0.83%	0.31	AGU	Ser (S)	0.25%	0.04	**U**
**A**	AUC	Ile (I)	4.22%	0.81	ACC	Thr (T)	2.65%	0.50	AAC	Aln (N)	1.81%	0.69	AGC	Ser (S)	1.36%	0.25	**C**
	AUA	Ile (I)	0.19%	0.04	ACA	Thr (T)	0.51%	0.10	AAA	Lys (K)	0.77%	0.24	AGA	Arg (R)	0.15%	0.02	**A**
	AUG	Met (M)	2.44%	1.00	ACG	Thr (T)	1.88%	0.35	AAG	Lys (K)	2.40%	0.76	AGG	Arg (R)	0.31%	0.05	**G**
	GUU	Val (V)	1.04%	0.14	GCU	Ala (A)	0.80%	0.07	GAU	Asp (D)	2.28%	0.39	GGU	Gly (G)	1.14%	0.13	**U**
**G**	GUC	Val (V)	2.98%	0.42	GCC	Ala (A)	5.45%	0.46	GAC	Asp (D)	3.51%	0.61	GGC	Gly (G)	4.91%	0.57	**C**
	GUA	Val (V)	0.29%	0.04	GCA	Ala (A)	1.20%	0.10	GAA	Glu (E)	2.63%	0.41	GGA	Gly (G)	0.86%	0.10	**A**
	GUG	Val (V)	2.87%	0.40	GCG	Ala (A)	4.45%	0.37	GAG	Glu (E)	3.82%	0.59	GGG	Gly (G)	1.72%	0.20	**G**
	**U**				**C**				**A**				**G**				
